# Can we induce spermatogenesis in the domestic cat using an *in vitro* tissue culture approach?

**DOI:** 10.1371/journal.pone.0191912

**Published:** 2018-02-07

**Authors:** Andreia F. Silva, Sara Escada-Rebelo, Sandra Amaral, Renata S. Tavares, Stefan Schlatt, João Ramalho-Santos, Paula C. Mota

**Affiliations:** 1 Biology of Reproduction and Stem Cell Group, Center for Neuroscience and Cell Biology (CNC), University of Coimbra, Coimbra, Portugal; 2 Institute for Interdisciplinary Research (IIIUC), University of Coimbra, Coimbra, Portugal; 3 Centre of Reproductive Medicine and Andrology, Institute of Reproductive and Regenerative Biology, University of Münster, Münster, Germany; 4 Department of Life Sciences, University of Coimbra, Coimbra, Portugal; University of Hyderabad, INDIA

## Abstract

The reduced number of animals in most wild felid populations implies a loss of genetic diversity. The death of juveniles, prior to the production of mature sperm, represents a loss of potential genetic contribution to future populations. Since 2011 mouse testicular organ culture has introduced an alternative mechanism to produce sperm in vitro from immature tissue. However, extension of this technology to other species has remained limited. We have used the domestic cat (Felis catus) as a model for wild felids to investigate spermatogenesis initiation and regulation, with the mouse serving as a control species. Testicular tissue fragments were cultured in control medium or medium supplemented with knockout serum replacement (KSR), AlbuMax, beta-estradiol or AlbuMax plus beta-estradiol. Contrary to expectations, and unlike results obtained in mouse controls, no germ cell differentiation could be detected. The only germ cells observed after six weeks of culture were spermatogonia regardless of the initial stage of tubule development in the donor tissue. Moreover, the number of spermatogonia decreased with time in culture in all media tested, especially in the medium supplemented with KSR, while AlbuMax had a slight protective effect. The combination of AlbuMax and beta-estradiol led to an increase in the area occupied by seminiferous tubules, and thus to an increase in total number of spermatogonial cells. Considering all the media combinations tested the stimulus for felid germ cell differentiation in this type of system seems to be different from the mouse. Studies using other triggers of differentiation and tissue survival factors should be performed to pursue this technology for the genetic diversity preservation in wild felids.

## Introduction

Most wild felids are listed as threatened, vulnerable or endangered. The loss of genetic diversity is a significant problem in felids given that juvenile mortality of valuable individuals is usually high. As young animals do not produce mature sperm, the premature and unexpected death of juveniles is especially worrying because their genetic contribution will be lost [1].

Spermatogenesis is a complex process that takes place in the seminiferous epithelium, physical and physiologically regulated by Sertoli, Leydig, and peritubular cells. Importantly it involves a strict spatial organization, but also intricate metabolic reactions. In order to recreate this peculiar testicular environment there are currently both *in vivo* and *in vitro* models (reviewed by [2]) where sperm production is the main goal. Recently, Sato and colleagues [3, 4] reported successful *in vitro* sperm production after organ culture of immature and adult mouse testis. This culture system is based on a liquid-gas interphase method in which small fragments of mouse testis are plated on agarose gel stands half-soaked in serum-free medium and maintained for more than two months at low temperature (34°C) in a 5% CO_2_ atmosphere [5]. The organ culture method allows for the maintenance of the testicular architecture and microenvironment and this is particularly important since the contact between germ cells and somatic cells, Sertoli cells in particular, seems to be critical for the success of the spermatogenic process [6] as they need to be present in order to obtain spermatid-like cells in all the *in vitro* culture systems developed so far [7–9]. On the other hand, there is an effective disruption in blood supply that hampers oxygenation and nutrient diffusion, and eliminates the central endocrine control (reviewed by [10]). Therefore, it is important to supplement the culture medium with key factors necessary for normal spermatogenesis. There is a well-established endocrine contribution for the progression of *in vivo spermatogenesis*, namely by FSH (follicle-stimulating hormone), LH (luteinizing hormone) and ultimately, testosterone. Briefly, FSH will directly stimulate Sertoli cells to increase germ cell number and, in turn, Sertoli cells will stimulate Leydig cells. This effect, together with the action of LH on Leydig cells, will lead to the production of testosterone required for normal spermatogenesis [11]. Due to the importance of these hormones, several studies have reported their effects as culture media supplements. In one of the studies, FSH was shown to be crucial for the progression of type A spermatogonia up to pachytene spermatocytes, while LH and testosterone had no effects [12]. In an organotypic culture of human testicular tissue, addition of hCG (human Chorionic Gonadotropin) did not stimulate testosterone production, observed to decrease to 50% of the normal value after 2 to 4 days of culture, nor did it induce the differentiation of germ cells, even when combined with FSH [13]. Moreover, it also reported that FSH and testosterone had no effect in their organ culture experiments [3]. Furthermore, hormones such as estradiol and progesterone also influence male gametogenesis. Estradiol is a testosterone metabolite produced in Leydig cells and its absence results in progressive loss of fertility and disruption of spermatogenesis [14]. Also in Leydig cells, cholesterol leads to the production of progesterone, a metabolic precursor of both androgens and estrogens [15].

In terms of media for organ culture, knockout serum replacement (KSR), a well-defined supplement, has been widely used in studies involving *in vitro* spermatogenesis. In fact, several reports claimed an increment in spermatogenesis [16] and progression of meiosis [3] when KSR was present, compared to other media supplementation. Another factor commonly used in testis organ culture is AlbuMAX (lipid-rich bovine serum albumin), a component of KSR, and shown in some studies to be crucial for the induction of spermatogenesis [3].

In this study we used the protocol described by Sato and colleagues [5] to attempt to induce *in vitro* sperm production in testicular tissue of the domestic cat (*Felis catus*), the best animal model of feline reproduction and closer to other higher mammals in terms of spermatogenesis initiation and regulation. For this purpose and to provide more reliable comparisons, several supplements already used in mice (KSR and AlbuMax) and an additional supplement, 17β-estradiol, were tested and their effects on *in vitro* survival and development compared with a basic control medium (MEMα). To control for species variability we also carried out experiments in mouse tissue.

## Materials and methods

### Animals

#### Mice

Testicular tissue of mice was used to control for species-specific differences [5]. The results, described in S2 File, were obtained using testicular tissue of C57BL/6NCr mice (B6 mice) or 129S2/SvPasCrl mice (129 mice) pups at 5 days post partum (5 pups per experiment, n = 3 or n = 2 for each strain, respectively) produced in the Center for Neuroscience and Cell Biology—Faculty of Medicine animal facility. All procedures were approved by the National Veterinary Committee (DGAV) and the Ethics Committee of our institution (ORBEA_40_2013/25022013) and following the Directive 2010/ 63/EU (revising Directive 86/609/EEC) on the protection of animals used for scientific purposes. Animals were sacrificed by cervical dislocation.

The testes were decapsulated in control media [MEMα (ThermoFisher Scientific, USA) + 1% (v/v) Penicillin/Streptomycin (PS; Sigma-Aldrich, MO, USA); + 1% (v/v) Fungizone (FZ, ThermoFisher Scientific, USA)], divided in small fragments of about 1–2 mm^3^ and processed for organ culture as described below for the domestic cat testicular tissue.

#### Domestic cat

All results were obtained using testes of young cats (n = 6) that were kindly provided by Conceição Pereira and Ana Luísa Pinto, veterinarians at the VetCoimbra veterinarian clinic, after owner-requested routine castrations. Our previous work with this species (18) indicated that the age gives very little information on the status of spermatogenesis development and many of the owners do not know the correct age of their animals (mostly former stray cats). Therefore, we chose to use testes weighing less than 1g that present a wide range of development, from total immaturity in some animals to the presence of sperm in some seminiferous tubules in others. However, as spermatogenic development in the cat is asynchronous, even cats with sperm in some seminiferous tubules may have areas of less developed or even immature seminiferous epithelium.

The tissue was kept in ice-cold transport medium [DMEM/F12- with 1% (v/v) Fungizone (FZ) and 1.5% L-glutamine (v/v) (Thermofisher Scientific, USA) plus 1% (v/v) non-essential amino acids and 2% (v/v) Penicillin/Streptomycin (P/S) (Sigma-Aldrich, MO, USA)] until further manipulation (maximum transport time was 30 minutes). Upon arrival to the laboratory, testes were separated from the epididymis and weighed. Testes were then decapsulated in manipulation medium [DMEM/F12 with 1% (v/v) non-essential amino acids; 1%PS (v/v); 1% FZ (v/v); 1.5% L-glutamine (v/v)], gently divided in small fragments of about 1–2 mm^3^ and processed for organ culture.

### Organ culture

The agar gel blocks were produced following the protocol described by Sato and colleagues. Briefly, agar powder (Agar Bacto; BD Difco) was dissolved in culture medium [MEMα with 1% (v/v) PS and 1% (v/v) FZ] to a concentration of 1.5% (w/v) and autoclaved (121°C; 15min). The hot agar solution was aliquoted in 15ml sterile Falcons and stored at 4°C. On the day of organ culture initiation the agar was melted at 85°C and placed in sterile petri dishes. After solidification the gel was cut into 5-8mm squares (4-5mm thickness) and placed in six well plates [5].

The 1–2 mm^3^ mouse testicular tissue fragments were placed on the top of agar gel blocks that were nearly submerged in 3 different culture media: control medium—MEMα +1% (v/v) PS; 1% (v/v) FZ]; MEMα with 10% (v/v) FBS (ES Qualified; ThermoFisher Scientific, USA); MEMα with 10% (v/v) KSR (ThermoFisher Scientific, USA) and cultured at 35°C with 5% carbon dioxide. Tissue fragments were collected at different time points, ranging from 15–56 days, and fixed in Bouin’s solution (Sigma-Aldrich, MO, USA).

Domestic cat testicular tissue fragments were placed on the top of agar gel blocks, and as in the mouse system, the blocks were nearly submerged in: MEMα–(Control medium); MEMα with 10% (v/v) KSR; MEMα with 100nM 17β-estradiol (Sigma-Aldrich, MO, USA); MEMα with 4% (v/v) AlbuMax II (ThermoFisher Scientific, USA) and MEMα with 100nM 17β-estradiol + 4% (v/v) AlbuMax II. 1% (v/v) PS; 1% (v/v) FZ were added to all mediums. Testicular tissue was incubated at 35°C with 5% carbon dioxide and medium exchange every 3–4 days. The testicular tissue fragments were imaged, using a Leica camera (DFC480) coupled to a Leica Microscope (DM4000B) at 50x magnification in brightfield, every week after second week of culture and samples of tissue cultured in each medium retrieved and fixed in Bouin’s solution to evaluate spermatogenesis development. As the spermatogenic cycle takes around 42 days in the domestic cat [17] we maintained the cat organ culture for 6 weeks.

### Histology and immunohistochemistry

The histological and immunohistochemistry (IHC) analysis was performed on paraffin embedded samples that were sectioned to 5μm thickness. Random sections of testicular tissue from domestic cat fixed at Day 0 and of each condition were selected, deparaffinized and rehydrated in a graded series of ethanol. One section of each fragment was routinely stained with hematoxylin and Periodic acid/Shiff-reagent while others were used for immunohistochemistry (IHC). These cross-sections were immersed in Tris-EDTA buffer (10mM Tris, 1mM EDTA, pH = 9) and heated in an autoclave, 15 min at 121°C, for antigen retrieval. To inactivate endogenous peroxidase activity samples were incubated with hydrogen peroxide for 15 min at room temperature (RT) and protected from light. Blocking of unspecific antibody binding was accomplished by incubating the tissue sections with a solution containing 25% (v/v) goat serum in Tris buffer saline (TBS– 10mM Trizma Base + 150mM Nacl pH = 7,6 adjusted with HCl) with 0.5% (w/v) BSA for 30 minutes at RT. The slides were then incubated with primary rabbit anti-PGP9.5 antibody (1:100; Z511601-2—DAKO, Denmark, overnight at 8°C). This antibody labels all gonocytes, spermatogonia and pre-leptotene germ cells from the domestic cat [18, 19]. For nonspecific labeling controls, sections were incubated in blocking solution without primary antibody (negative control) or with rabbit IgG (I5006, Sigma-Aldrich; unspecific IgG labeling control). After three washes in TBS, sections were firstly incubated with biotinylated goat anti-rabbit antibody (1 hour at RT, A3687: Sigma-Aldrich, St-Luis, MO) and, after washing 3x in TBS, with streptavidin-horseradish peroxidase (ST-HRP—Sigma-Aldrich, MO, USA; 1:500, 30 min RT). After washing the slides, labeling was detected with 3,3-diaminobenzidine tetrahydrochloride (DAB—Sigma-Aldrich, MO, USA), until color developed as observed under the microscope. Positive staining appeared as a brown precipitate in the cells. After washing the slides in deionized water, samples were counterstained with hematoxylin for 5–10 minutes and washed in tap water for 15 minutes. After a quick step in distilled water, samples were immersed in increasing concentrations of ethanol and mounted with a xylene based permanent medium (Eukitt: Sigma-Aldrich, MO, USA).

### Histological and morphometric analysis

The histology of mouse testicular tissue fragments (S2 File, Panel A) was described and categorized according to the table in S2 File—Panel B and plotted in the graph (S2 File, Panel C).

Images from domestic cat testis sections of 6 animals and one hundred and fifty organ culture samples (n = 6 x 5 conditions x 5 time points) were taken with a Leica camera (DFC480) coupled to a Leica Microscope (DM4000B) at 200x magnification. After random selection of approximately 50% of the images taken from each sample, *Image J* software was used to quantify all PGP9.5 positive cells present in the image (corresponding to germ cell number, as all germ cells present in the tissue were labeled by PGP9.5) and the area (measured in arbitrary units) covered by seminiferous tubules, necrotic and interstitial tissue using a grid with 1.5 square inches. The area covered by the different types of tissue was represented as percentage of the total area measured (% of seminiferous tubules, % of necrotic tissue and % of interstitial tissue and the number of germ cells were normalized in terms of the area covered by seminiferous tubules (number of germ cells divided by area of seminiferous tubules).

### Statistical analysis

Statistical analysis, only assessed in domestic cat testicular tissue, was performed using SPSS (Statistical Package for the Social Sciences Program), version 21.00, software for Mac (SPSS Inc., Chicago, IL, USA). All variables were checked for normal distribution. To assess possible differences induced by medium supplementation in the medians of the parameters analyzed—percentage of seminiferous tubule area, interstitial and necrotic tissue as well as number of germ cells we used the Wilcoxon Signed Ranks Test. The same test was used to access differences between Day0 and the remaining time points for each medium. The differences between control culture medium and supplementations and between Day0 and other time points were considered statistically significant when P<0.05. All data collected and statistics test results are included in S1 File. Graphical representation was performed using Excel, version 14.4.1 for Mac.

## Results

As stated in the Material and Methods section, we decided to test our capacity to reproduce previous results [5] in our laboratory using mouse testicular tissue. The results, presented as supplemental data in Panels A, B and C of S2 File, confirm the ability of KSR to stimulate meiosis, with the observation of round spermatids after 21 days in the B6 mouse strain and 28 days in the 129 mouse strain. However, when longer culture times were used, we did not observe the presence of elongating spermatids detecting on the other hand atrophy of the seminiferous epithelium with decreased germ cell layers.

**Fig 1:**
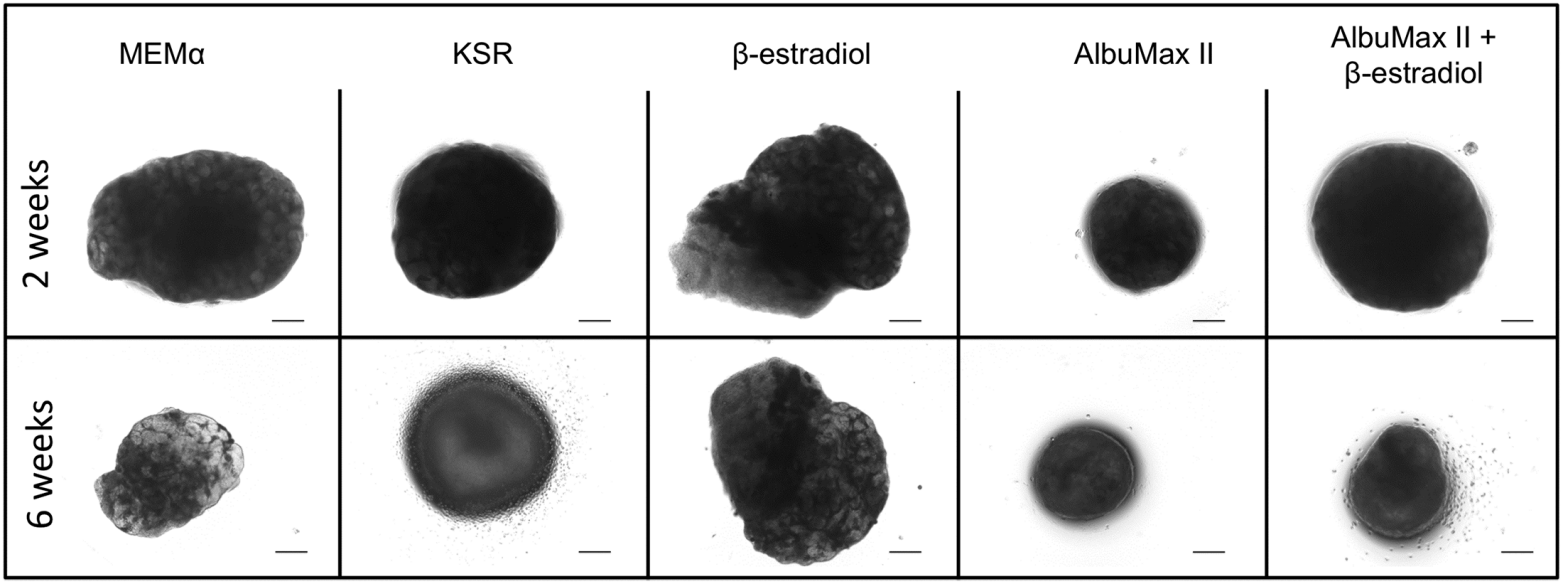
Domestic cat testicular tissue cultured for 2 and 6 weeks in the different types of media tested. Representative brightfield images of testicular tissue of the same animal cultured for 2 and 6 weeks. Scale bar: 100μm.

All data collected from the 6 experiments performed and corresponding statistics analysis may be found in S1 File. In the domestic cat, after the first 15 days in culture, testicular tissue fragments were observed weekly and images taken to register macroscopic alterations during the culture period. In Fig 1 a deterioration of tissue architecture throughout the culture period in visible, however the most striking observation occurs in the tissues in contact with KSR-containing medium and, to a lesser extent, those containing AlbuMax II. In fact, under these conditions, after 3 to 4 weeks in culture we could no longer distinguish the walls of the seminiferous tubules and the fragments, until this point flattened, acquired a spheroid shape.

**Fig 2:**
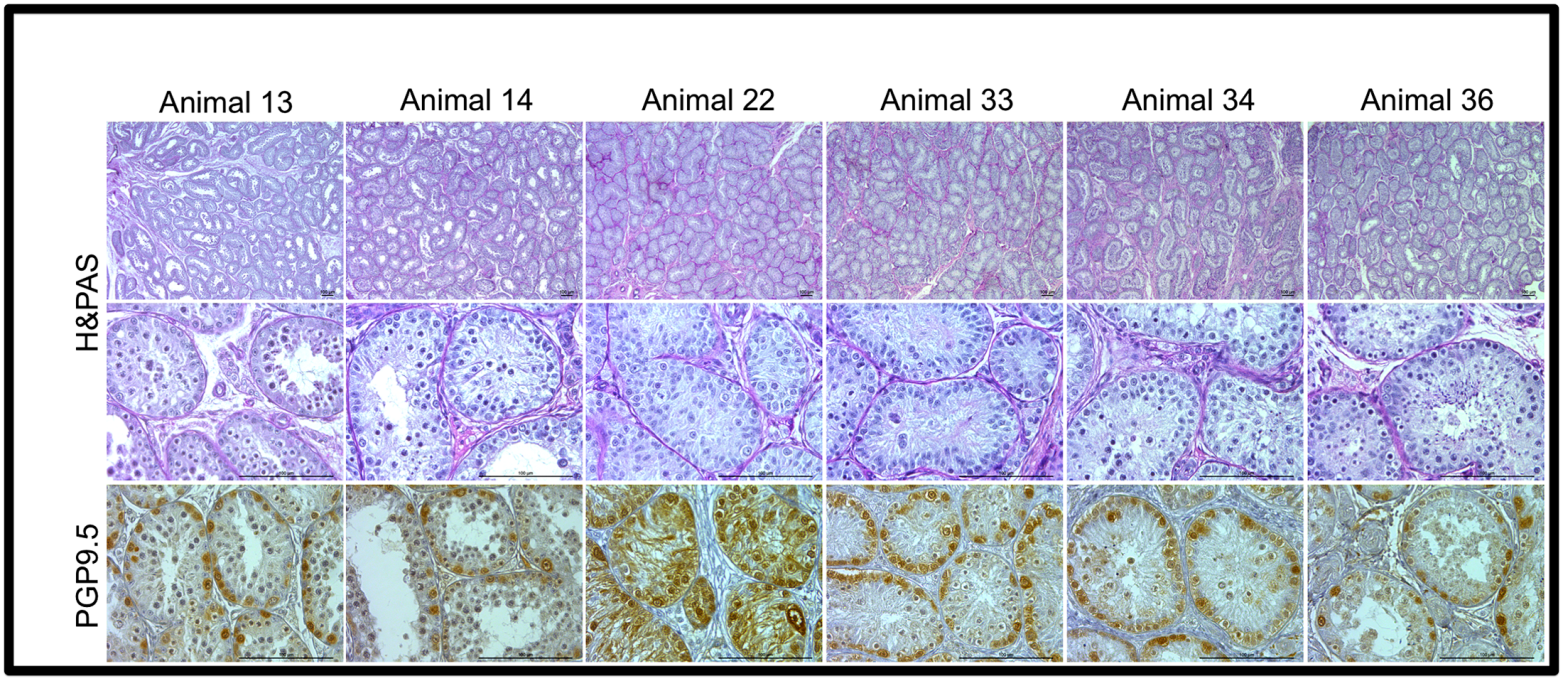
Histological and immunohistochemistry of domestic cat testicular tissue fixed at Day0. Immature and peri-pubertal testicular tissue collected and fixed at Day 0, before any culture, stained with H&PAS and labeled with anti-PGP9.5 antibody (labeled cells show a brown color stain). Scale bar: 100μm.

**Fig 3:**
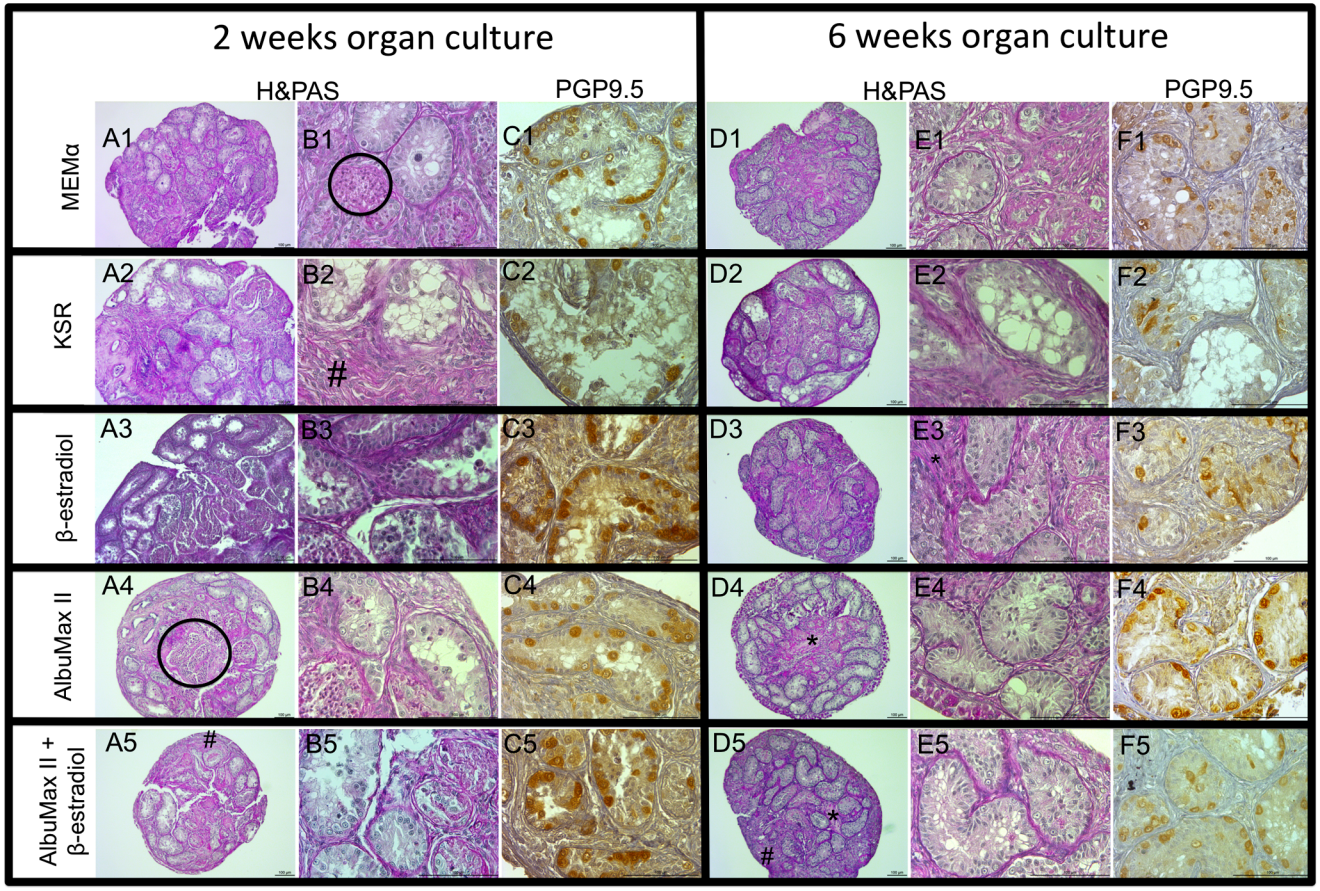
Histological and immunohistochemistry of domestic cat testicular fragments cultured *in vitro*. A, B represent histological and C PGP9.5 immunohistochemical images of fragments cultured for 2 weeks while D, E and F represent the same assays in tissue cultured for 6 weeks. Numerical categorization relates to culture medium with 1- MEMα, 2- MEMα with 10% KSR, 3—MEMα with 100nM 17β-estradiol, 4- MEMα with 4% AlbuMax II; and 5- MEMα with 100nM 17β-estradiol + 4% AlbuMax II. **#**—lax connective tissue, *—fibrotic tissue, circled area—necrotic tissue. Scale bar: 100μm.

Although we analyzed tissue fragments of all time points (Day 0, week 2, 3, 4, 5 and 6), we decided to present the histological and IHC appearance of the testicular tissue fragments at Day 0 (Fig 2) and recovered at week 2 and 6, which represent the beginning and end of tissue collection (Fig 3).

**Fig 4:**
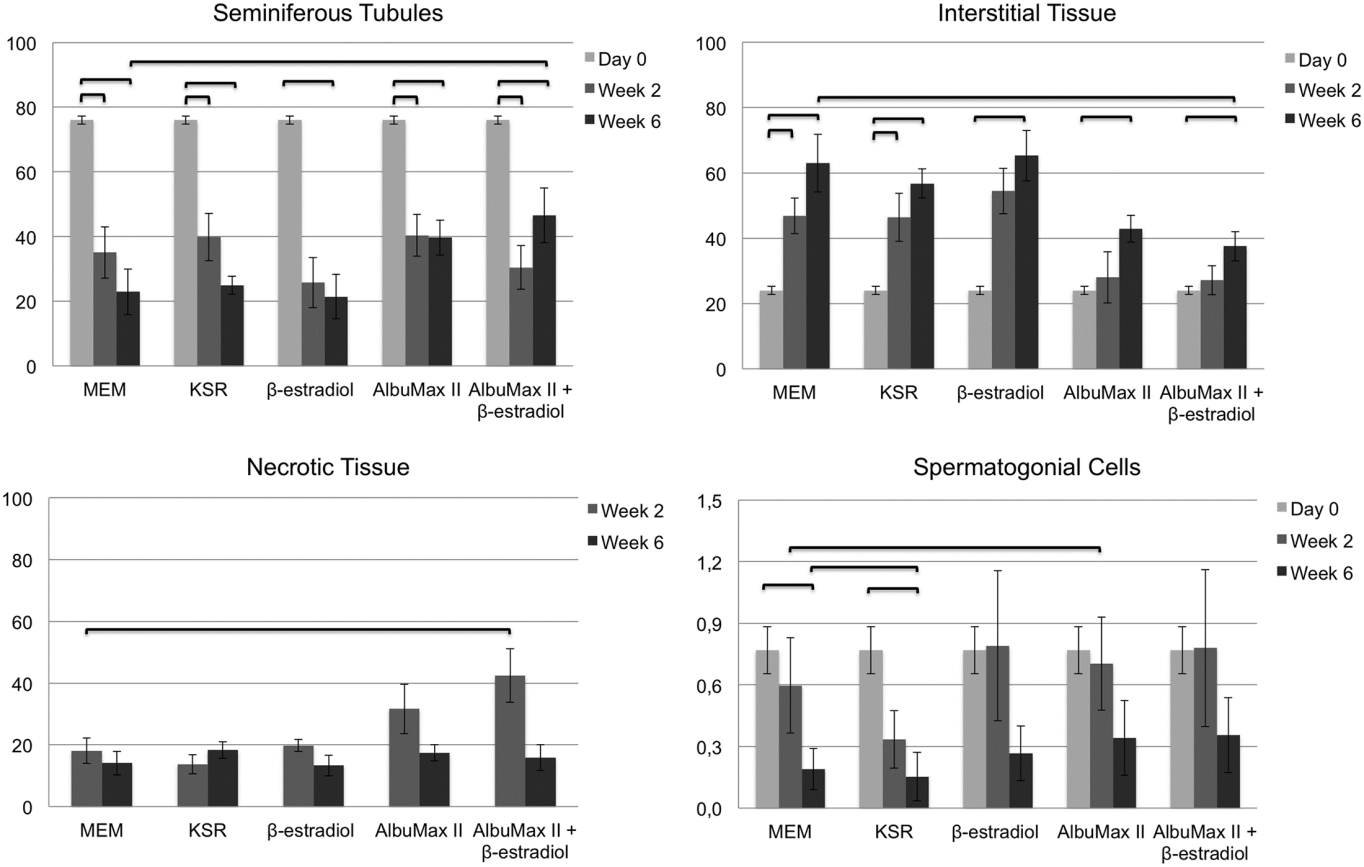
Graphical representation of the histomorphometric analysis of cat tissue performed using image J software. Percentage of the fragment area covered by Seminiferous Tubules, Interstitial Tissue and Necrotic Tissue. Number of spermatogonia per seminiferous tubule area—Spermatogonial Cells. Bars represent Mean±SEM. Straight parenthesis in the top of the column represent significant differences (P<0.05) relatively to Day 0 or control medium. N = 5 animals for the 2 week analysis and N = 6 animals for the 6 week analysis.

The morphometric analysis performed on cultured sections from all 6 animals, is shown graphically in Fig 4, and details of the Day 0 testicular tissue, in Table 1.

**Table 1:** Day 0 testicular tissue characterization.

Animal ID	Testes weight (in grams)	Percentage of area ST	Percentage of area interstitial tissue	Germ cells per ST area		
				Spermatogonia	Spermatocytes	Spermatids
13	0.91	80.99	19.01	131.92	34.87	66.28
14	0.89	73.20	26.80	79.92	28.44	37.47
22	0.43	72.95	27.05	62.61	5.75	0
33	0.76	77.87	22.13	63.30	14.58	8.02
34	0.72	75.35	24.65	55.68	10.48	21.72
36	0.92	75.68	24.32	67.63	32.24	70.64

Morphometric analysis was performed with H&PAS labeled cross-sections and Image J software.

The area of the tissue fragment occupied with seminiferous tubules decreased in relation to Day0 (MEMα, β-estradiol, AlbuMax II and β-estradiol plus AlbuMax II—P = 0,043, n = 5; KSR– 0.068, n = 4 at week 2 and MEMα, KSR, β-estradiol, AlbuMax II and β-estradiol plus AlbuMax II—P = 0.028, n = 6 at week 6) without any observable benefit from medium supplementation (Figs 2 and 3). At the end of the culture period (week 6) a slight protection conferred by supplementation with AlbuMax II plus β-estradiol relative to control medium (P = 0.046, n = 6; Fig 4) could be observed (Fig 3 D1 *vs* D5). The interstitial tissue, which varied from fibrotic (see * in Fig 3) to highly lax connective tissue (see # in Fig 3), increased, in relation to Day0, during the entire culture period in all media tested. This effect was more prominent in the tissue cultured in control medium, KSR and β-estradiol (MEMα and KSR—P = 0,043 at week 2 and P = 0,028 for MEMα, KSR, β-estradiol, AlbuMax II and β-estradiol plus AlbuMax II at week 6). Analysis of the effect of supplementation revealed that AlbuMax II plus β-estradiol (Fig 4) significantly decreased the area occupied by interstitial tissue in relation to control medium (P = 0.028, n = 6), after 6 weeks of culture.

On the other hand, the percentage of necrotic tissue, denoted by the higher PAS staining intensity (see circled area in Fig 3), was significantly higher (P = 0.043, n = 5) in tissue cultured with AlbuMax II plus β-estradiol than in control medium after 2 weeks in culture, with a subsequent decrease at week 6 (Fig 4).

Immunohistochemical labeling of the testicular tissue fragments with anti-PGP9.5 antibody, which labels all the germ cells until the pre-leptotene stage, allowed for the assessment of the number of spermatogonial cells per area of seminiferous tubules showing that, as already hinted in the histological assessment, there was no germ cell development beyond the spermatogonial stage (Fig 3, C1-5 and F1-5) as all cells inside the seminiferous tubules not labeled by brown precipitate were Sertoli cells, even in the animals that in Day0 tissue presented more developed germ cells (Fig 2, Table 1).

Image J software analysis showed that the number of spermatogonial cells per seminiferous tubule area decreased with time in culture when compared with Day0, however this effect only reached significant differences in tissue cultured in control or KSR supplemented media, especially at week 6 (P = 0.028, n = 6; P = 0,046, n = 6, respectively). There were no differences in the number of germ cells per area of seminiferous tubule in tissue cultured with AlbuMax, β-estradiol or both, relative to Day0. Supplementation of culture medium with KSR had a particular deleterious effect, inducing more germ cell loss than other media when compared to the control medium (2 and 4 weeks: P = 0.043; 3 and 5 weeks: P = 0.028, n = 6;Fig 4). On the other hand, medium supplemented with AlbuMax II presented a slight protective effect in the early phases of culture relative to control medium (P = 0.043, n = 5), with an increase in the number of spermatogonia per seminiferous tubule area (Fig 4).

## Discussion

The first report of *in vitro* spermatogenesis goes back to 1915 with the Goldschmidt study in moth pupa testis [20]. A few decades later Trowell developed a liquid-gas interphase organ culture method using different types of tissues, including testis. However, the testis culture was unsuccessful, with almost total tissue degeneration [21]. It was only in the 1960s that some valuable studies using the organ culture method began to emerge, with the differentiation of gonocytes/spermatogonia to meiotic cells [22–27]. However, the progression of the spermatogenic process up to haploid cells with an *in vitro* organ culture approach was only achieved a few years ago by Sato and colleagues, after performing some modifications to the previous culture protocols [4]. Although spermatid production was a rare event, overcoming the meiosis barrier was a critical step, with massive importance for the field, as was the report of live births resulting from the sperm produced *in vitro* using this system [3]. However, it has only been possible to overcome the meiotic barrier in other rodents species such as the rat (*Rattus norvegicus*) very recently [28], and to the best of our knowledge, no further studies using this methodology have reported similar results in other non-rodent mammalian species. In a recent report Perrard and colleagues introduced a new bioincubator that, according to the authors, may help maintain seminiferous tubule architecture, and facilitate sperm production in the rat and human seminiferous epithelium [29].

Considering that the number of endangered species is increasing, research to potentially help solve reproductive problems could aid the preservation of endangered populations. In the present study, we used the organ culture system [5] applied to the testicular tissue of immature and early pubertal domestic cats in an attempt to produce sperm and understand the progress of feline spermatogenesis *in vitro*. In mice, the entire process of spermatogenesis takes 35 days while in the domestic cat the spermatogenic cycle takes around 42 days [17] and for that reason we decided to culture the tissue for 6 weeks.

One of the most striking breakthroughs that led to the previously reported success in organ culture experiments was the addition of knockout serum replacement (KSR) or AlbuMax (one of the KSR constituents) to the medium, suggesting that both KSR and AlbuMax were effective in the induction and promotion of spermatogenesis in neonatal or pup mouse testis fragments [3]. In our mice experiments we report similar results with KSR supplementation, although we were not able to observe elongating spermatids, thus suggesting the need for further optimization of the system.

Conversely, in our cat organ culture system with KSR or AlbuMax supplementation we could no longer distinguish the walls of the seminiferous tubules macroscopically after 3 to 4 weeks, and the tissue adopted a spheroid appearance, contrarily to what was observed in the control medium (MEMα) or in medium supplemented with β-estradiol. Despite their similarity in terms of macroscopic evaluation, the microscopic analysis of testicular tissue cultured with either KSR and AlbuMax II showed clearly divergent results. After histological processing the fragments cultured with KSR showed large vacuoles, huge loss of germ cells, and after 6 weeks, even the number of Sertoli cells was low. More importantly there was no germ cell development beyond the spermatogonial stage. This last observation was confirmed after immunohistochemical labeling of the testicular tissue fragments with anti-PGP9.5 antibody. PGP9.5 (Protein gene product 9.5), also known as UCHL-1 (Ubiquitin carboxy-terminal hydrolase 1), which is expressed in spermatogonia in a variety of species, including mouse [30], monkey [31], human [32], bovine [33] and pig [34]. In the domestic cat, PGP9.5 labels gonocytes and spermatogonial stem cells in immature animals and all spermatogonia and early spermatocytes (pre-leptotene) in pubertal and adult animals [35]. In our cat organ culture the anti-PGP9.5 antibody labeled all the germ cells present indicating that no germ cell had crossed the meiotic barrier.

Throughout the culture period KSR supplementation led to significant germ cells loss, with higher deleterious effect compared to all other media. Although the beneficial effect of medium supplementation with KSR has been previously reported [3], there seems to be some controversy in the literature. Recently it was reported that KSR alters both somatic and germ cell differentiation in a fetal testis organ culture system [36] perhaps due to the fact that KSR tends to maintain cells in an undifferentiated and proliferative state, which prompted its use in embryonic stem cell cultures [37]. Curiously, media supplemented with one of the KSR constituents—AlbuMax—showed a protective effect, as observed by an increase in the number of spermatogonia, when compared to the control situation at 2 weeks of culture and no significant difference relative to Day0, even after 6 weeks of in vitro culture. There is no straightforward explanation for this divergent behavior between KSR and AlbuMax, nor to the divergent results in species tested in our lab in comparison with published literature. In fact, in a pilot study, after culturing mouse testicular tissue for 21–28 days in medium supplemented with KSR we observed differentiation of spermatogonia until round spermatids, a sign that the meiosis barrier had been surpassed. Furthermore, no deleterious effects, such as vacuolization of the seminiferous epithelium, were found in this case. This supports the idea that the differences observed between species are not due to culture conditions but rather to intrinsic differences in testicular tissue and spermatogenesis regulation.

Furthermore, we also decided to evaluate the effects of β-estradiol in spermatogenesis development, as it seems to be a potent germ cell survival factor, both in human [38, 39] and in rat testis [40]. Moreover, in teratospermic cats, which present increased sperm production due to a decreased apoptotic rate [41, 42], the testosterone to estradiol ratio is altered with an increase in the intratesticular levels of estradiol [43]. These experiments led us and other researchers [44] to hypothesize that β-estradiol might promote germ cell survival also in the domestic cat. Although we indeed observed an increase in the number of germ cells in the tissue cultured with β-estradiol when compared to the control medium, this effect did not reach statistical significance. However, as was the case for AlbuMax, β-estradiol was able to support germ cell survival, given that no significant differences relative to Day 0 were observed. When both supplements, AlbuMax and β-estradiol, were used a synergetic effect was observed after 6 weeks in culture, with fragments of cultured tissue presenting a significant increase in the area occupied by seminiferous tubules, a decrease in interstitial tissue, and higher germ cell numbers. This synergistic effect may be due to the regulation of poly-unsaturated fatty acids (PUFA), present in the lipid-rich bovine albumin (AlbuMax II) by estradiol, as already observed in other tissues and types of cells [45, 46]. Increasing evidence indicates that lipids, particularly (n-6) and (n-3) PUFA, play significant roles in cell signaling and gene expression, and are thereby linked to many physiological and pathological processes (reviewed by [47]). The high levels of PUFA present in testis may be an indicative of a role in stem cell niche maintenance, besides the already established role in spermatid differentiation [48].

In conclusion, the domestic cat testis organ culture did not follow the same pattern of development as reported in the mouse [3, 16], resulting in long term maintenance of some spermatogonia, possibly spermatogonial stem cells, but no further germ cell development. This was not only a problem of spermatogenesis initiation but also in its progression, since even tissue with more developed germ cells at Day 0 showed no further germ cell differentiation. Analysis of the existing literature reveals that spermatogenesis initiation and/or regulation in the domestic cat is likely more complex than in rodents. In fact, the overall rate of spermatogenesis is lower in the cat when compared with various rodents (rat, mouse, gerbil, capybara, paca, cutia, chinchilla) and several other mammals (bull, buffalo, ram, goat, boar, wild boar, peccary, dog, rabbit, marmoset) as is the meiotic index (reviewed by [49]). Furthermore, we and other researchers [17, 42] regularly observe seminiferous epithelium abnormalities in the cat.

Finally, we believe that, although no further germ cell development was obtained, the observation of some germ cell survival after 6 weeks of in vitro culture indicates that this approach may be viable in the cat. Our organ culture experiments provide some insights as to which steps and components of the system can be improved as well as which factors are necessary (or not) to induce and promote the spermatogenesis in this species. Our data also highlights that this culture system needs to be addressed in more detail, so results may be robustly reproducible in different species. In future studies, optimal culture conditions for felids must be established. Currently lacking information regarding the regulatory mechanisms of domestic cat spermatogenesis might also prove crucial.

## Supporting information

S1 File Data collection and statistical analysis. (PDF)

S2 File Mice testicular tissue organ culture. Histology, categorization and graphical representation (Panel A, B and C, respectively) of organ culture of B6 and 129 mouse strains testicular tissue using the protocol of Sato and colleagues [5].
(PDF)
