# Supplementary material for: Can we induce spermatogenesis in the domestic cat using an in vitro tissue culture approach?
Source: PLoS One. 2018 Feb 7;13(2):e0191912. doi: 10.1371/journal.pone.0191912 (PMC5802888; doi:10.1371/journal.pone.0191912)
Supplement: S1 File — (PDF) [file pone.0191912.s001.pdf]

| ID | Description |
|---|---|
| 2A | Week 2 - MEMα (control medium) |
| 2B | Week 2 - MEMα + 10%KSR |
| 2C | Week 2 - MEMα + 100nM 17β-estradiol |
| 2D | Week 2 - MEMα + 4% AlbuMax II |
| 2E | Week 2 - MEMα + 4% AlbuMax II + 100nM 17β-estradiol |
| 3A | Week 3 - MEMα (control medium) |
| 3B | Week 3 - MEMα + 10% KSR |
| 3C | Week 3 - MEMα + 100nM 17β-estradiol |
| 3D | Week 3 - MEMα + 4% AlbuMax II |
| 3E | Week 3 - MEMα + 4% AlbuMax II + 100nM 17β-estradiol |
| 4A | Week 4 - MEMα (control medium) |
| 4B | Week 4 - MEMα + 10% KSR |
| 4C | Week 4 - MEMα + 100nM 17β-estradiol |
| 4D | Week 4 - MEMα + 4% AlbuMax II |
| 4E | Week 4 - MEMα + 4% AlbuMax II + 100nM 17β-estradiol |
| 5A | Week 5 - MEMα (control medium) |
| 5B | Week 5 - MEMα + 10% KSR |
| 5C | Week 5 - MEMα + 100nM 17β-estradiol |
| 5D | Week 5 - MEMα + 4% AlbuMax II |
| 5E | Week 5 - MEMα + 4%AlbuMax II + 100nM 17β-estradiol |
| 6A | Week 6 - MEMα (control medium) |
| 6B | Week 6 - MEMα + 10% KSR |
| 6C | Week 6 - MEMα + 100nM 17β-estradiol |
| 6D | Week 6 - MEMα + 4% AlbuMax II |
| 6E | Week 6 - MEMα + 4% AlbuMax II + 100nM 17β-estradiol |

**Randomization of the images to analyse: aplication of the function RAND (returns a random number greater or equal to 0 and less than 1). Selection of 50% of images with smallest number.**

| | |
|---|---|
| **animal 13** | |
| 3A_20x.tif | 0,68203435 |
| 3A_20x1.tif | 0,08411901 |
| 3A_20x2.tif | 0,95904033 |
| 3A_20x3.tif | 0,64857639 |
| 3A_20x4.tif | 0,92878801 |
| 3A_20x5.tif | 0,08764249 |
| 3A_20x6.tif | 0,60941828 |
| 3A_20x7.tif | 0,84260112 |
| 3A_20x8.tif | 0,71667313 |
| 3A_20x9.tif | 0,87465947 |
| 3A_20x10.tif | 0,0504561 |
| 3A_20x11.tif | 0,27778603 |
| | |
| | |
| 3B_20x.tif | 0,76690103 |
| 3B_20x2.tif | 0,39970962 |
| 3B_20x3.tif | 0,37138548 |
| 3B_20x4.tif | 0,01935306 |
| 3B_20x5.tif | 0,36726933 |
| 3B_20x6.tif | 0,14941133 |
| 3B_20x7.tif | 0,10524539 |
| 3B_20x8.tif | 0,67605512 |
| | |
| | |
| 3C_20x.tif | 0,02302561 |
| 3C_20x1.tif | 0,5518913 |
| 3C_20x2.tif | 0,04108246 |
| 3C_20x3.tif | 0,47252507 |
| 3C_20x4.tif | 0,84476761 |
| 3C_20x5.tif | 0,87554635 |
| | |
| | |
| 3D_20x.tif | 0,88466572 |
| 3D_20x1.tif | 0,69471665 |
| 3D_20x2.tif | 0,67899237 |
| 3D_20x3.tif | 0,73687992 |
| | |
| | |
| 3E_20x.tif | 0,41809048 |
| 3E_20x2.tif | 0,08891265 |
| 3E_20x3.tif | 0,82193723 |
| 3E_20x4.tif | 0,97892526 |

| | |
|---|---|
| 3E_20x5.tif | 0,16105942 |
| 3E_20x6.tif | 0,74334896 |
| 3E_20x7.tif | 0,56826195 |
| 3E_20x9.tif | 0,74330531 |
| 3E_20x10.tif | 0,97852412 |

| | |
|---|---|
| 4A_20x.tif | 0,0186744 |
| 4A_20x1.tif | 0,17918411 |
| 4A_20x2.tif | 0,14472287 |
| 4A_20x3.tif | 0,86889767 |
| 4A_20x4.tif | 0,00764232 |
| 4A_20x5.tif | 0,03075895 |
| 4A_20x6.tif | 0,03337889 |
| 4A_20x7.tif | 0,62245493 |
| 4A_20x8.tif | 0,80921143 |
| 4A_20x9.tif | 0,42238088 |
| 4A_20x10.tif | 0,91509748 |
| 4A_20x11.tif | 0,42048459 |
| 4A_20x12.tif | 0,78338325 |

| | |
|---|---|
| 4B_20x.tif | 0,07794554 |
| 4B_20x1.tif | 0,2439805 |
| 4B_20x2.tif | 0,81138942 |
| 4B_20x3.tif | 0,8451465 |
| 4B_20x4.tif | 0,99079555 |
| 4B_20x5.tif | 0,84775098 |
| 4B_20x6.tif | 0,59211389 |
| 4B_20x7.tif | 0,88931089 |
| 4B_20x8.tif | 0,43032454 |

| | |
|---|---|
| 4C_20x.tif | 0,25760627 |
| 4C_20x1.tif | 0,58830075 |
| 4C_20x2.tif | 0,01430998 |
| 4C_20x3.tif | 0,48507411 |
| 4C_20x4.tif | 0,20846715 |

| | |
|---|---|
| 4D_20x.tif | 0,98398459 |
| 4D_20x1.tif | 0,75333874 |
| 4D_20x2.tif | 0,78901461 |
| 4D_20x3.tif | 0,06032509 |
| 4D_20x4.tif | 0,31945045 |

| | |
|---|---|
| 4E_20x.tif | 0,09879359 |
| 4E_20x1.tif | 0,2718875 |
| 4E_20x2.tif | 0,18565816 |
| 4E_20x3.tif | 0,16346407 |
| 4E_20x4.tif | 0,80842563 |
| 4E_20x5.tif | 0,91058514 |
| 4E_20x6.tif | 0,21421905 |
| 4E_20x7.tif | 0,88962743 |
| 4E_20x8.tif | 0,81874351 |

| | |
|---|---|
| 5A_20x.tif | 0,42197368 |
| 5A_20x1.tif | 0,50735386 |
| 5A_20x2.tif | 0,47547234 |
| 5A_20x3.tif | 0,2923641 |
| 5A_20x4.tif | 0,33667984 |
| 5A_20x5.tif | 0,10351004 |
| 5A_20x6.tif | 0,0808175 |
| 5A_20x7.tif | 0,81494768 |
| 5A_20x8.tif | 0,11581051 |
| 5A_20x9.tif | 0,01263715 |
| 5A_20x10.tif | 0,81087883 |
| 5A_20x11.tif | 0,34395413 |
| 5A_20x12.tif | 0,16824191 |
| 5A_20x13.tif | 0,71165469 |
| 5A_20x14.tif | 0,03745919 |
| 5A_20x15.tif | 0,92758771 |

| | |
|---|---|
| 5B_20x.tif | 0,14689157 |
| 5B_20x1.tif | 0,24107125 |
| 5B_20x2.tif | 0,84642152 |
| 5B_20x3.tif | 0,98361482 |
| 5B_20x4.tif | 0,22632832 |
| 5B_20x5.tif | 0,92166473 |
| 5B_20x6.tif | 0,65341043 |
| 5B_20x7.tif | 0,18155505 |
| 5B_20x8.tif | 0,0284159 |
| 5B_20x9.tif | 0,69871915 |
| 5B_20x10.tif | 0,3316659 |
| 5B_20x11.tif | 0,34299871 |
| 5B_20x12.tif | 0,06992288 |
| 5B_20x13.tif | 0,97661613 |
| 5B_20x14.tif | 0,66471346 |
| 5B_20x15.tif | 0,77882949 |

| | |
|---|---|
| 5C_20x.tif | 0,63050954 |
| 5C_20x1.tif | 0,46070848 |
| 5C_20x2.tif | 0,61121537 |
| 5C_20x4.tif | 0,67265615 |
| 5C_20x5.tif | 0,37136408 |
| 5C_20x6.tif | 0,90655312 |
| 5C_20x7.tif | 0,35226449 |
| 5C_20x8.tif | 0,82822243 |
| 5C_20x9.tif | 0,02003181 |
| 5C_20x11.tif | 0,52377653 |
| 5C_20x12.tif | 0,36882972 |
| 5C_20x13.tif | 0,25828465 |
| 5C_20x14.tif | 0,53587347 |
| 5C_20x15.tif | 0,42086509 |
| 5C_20x16.tif | 0,14716377 |
| | |
| | |
| 5D_20x.tif | 0,47131544 |
| 5D_20x2.tif | 0,9545121 |
| 5D_20x3.tif | 0,31729692 |
| 5D_20x4.tif | 0,35484128 |
| 5D_20x6.tif | 0,31377832 |
| | |
| | |
| 5E_20x.tif | 0,81578722 |
| 5E_20x1.tif | 0,89639699 |
| 5E_20x2.tif | 0,88473722 |
| | |
| | |
| 6A_20x.tif | 0,26216952 |
| 6A_20x1.tif | 0,26273823 |
| 6A_20x2.tif | 0,53130309 |
| 6A_20x3.tif | 0,50643723 |
| 6A_20x4.tif | 0,72589133 |
| 6A_20x5.tif | 0,47856394 |
| 6A_20x6.tif | 0,59121983 |
| 6A_20x7.tif | 0,06369688 |
| 6A_20x8.tif | 0,00824124 |
| 6A_20x9.tif | 0,97492826 |
| 6A_20x10.tif | 0,38019606 |
| 6A_20x11.tif | 0,36033069 |
| 6A_20x12.tif | 0,88871752 |
| 6A_20x13.tif | 0,43847743 |

| | |
|---|---|
| 6B_20x.tif | 0,81854777 |
| 6B_20x1.tif | 0,0673386 |
| 6B_20x2.tif | 0,21726905 |
| 6B_20x3.tif | 0,74324384 |
| 6B_20x4.tif | 0,46354797 |
| 6B_20x5.tif | 0,14007734 |
| 6B_20x6.tif | 0,37578745 |
| 6B_20x7.tif | 0,34445326 |
| 6B_20x8.tif | 0,93131325 |
| 6B_20x9.tif | 0,01089913 |
| 6B_20x10.tif | 0,66970445 |
| 6B_20x11.tif | 0,39120006 |
| 6B_20x12.tif | 0,55083176 |
| 6B_20x13.tif | 0,81073256 |

| | |
|---|---|
| 6C_20x.tif | 0,70639455 |
| 6C_20x1.tif | 0,2872259 |
| 6C_20x2.tif | 0,93969284 |
| 6C_20x3.tif | 0,15709575 |
| 6C_20x4.tif | 0,35864782 |
| 6C_20x5.tif | 0,24833074 |
| 6C_20x6.tif | 0,61233463 |

| | |
|---|---|
| 6D_20x.tif | 0,10169738 |
| 6D_20x1.tif | 0,0003347 |
| 6D_20x2.tif | 0,44649831 |
| 6D_20x3.tif | 0,97925418 |
| 6D_20x4.tif | 0,67618195 |

| | |
|---|---|
| 6E_20x.tif | 0,80228647 |
| 6E_20x1.tif | 0,9300843 |
| 6E_20x2.tif | 0,21284041 |
| 6E_20x3.tif | 0,58852477 |
| 6E_20x4.tif | 0,36431656 |

| **animal 14** | |
|---|---|
| 2A_20x.tif | 0,16232881 |
| 2A_20x1.tif | 0,06717446 |
| 2A_20x2.tif | 0,61945671 |
| 2A_20x3.tif | 0,42486215 |
| 2A_20x4.tif | 0,57906502 |

| | |
|---|---|
| 2A_20x5.tif | 0,58542923 |
| 2A_20x6.tif | 0,00830139 |
| 2A_20x7.tif | 0,60510028 |
| 2A_20x8.tif | 0,04930325 |
| 2A_20x9.tif | 0,33102703 |
| 2A_20x10.tif | 0,83757512 |
| 2A_20x11.tif | 0,26118688 |
| 2A_20x12.tif | 0,99738057 |
| 2A_20x13.tif | 0,77151721 |
| 2A_20x14.tif | 0,21394209 |
| | |
| 2B_20x.tif | 0,10648241 |
| 2B_20x1.tif | 0,13203338 |
| 2B_20x2.tif | 0,31099839 |
| 2B_20x3.tif | 0,05967627 |
| 2B_20x4.tif | 0,67719291 |
| 2B_20x5.tif | 0,40623534 |
| | |
| 2C_ (nd) | |
| | |
| 2D_20x.tif | 0,12455821 |
| 2D_20x1.tif | 0,15961694 |
| 2D_20x2.tif | 0,43129722 |
| 2D_20x3.tif | 0,19390329 |
| 2D_20x4.tif | 0,27472867 |
| 2D_20x5.tif | 0,43308241 |
| 2D_20x6.tif | 0,24180842 |
| 2D_20x7.tif | 0,76437167 |
| | |
| 2E_20x.tif | 0,842879 |
| 2E_20x1.tif | 0,76604552 |
| 2E_20x2.tif | 0,70954229 |
| 2E_20x3.tif | 0,48003008 |
| 2E_20x4.tif | 0,64736903 |
| 2E_20x5.tif | 0,62699543 |
| 2E_20x6.tif | 0,12833644 |
| 2E_20x7.tif | 0,93129023 |
| 2E_20x8.tif | 0,00382706 |
| | |
| 3A_20x.tif | 0,13989833 |

| | |
|---|---|
| 3A_20x1.tif | 0,82111553 |
| 3A_20x2.tif | 0,08137072 |
| 3A_20x3.tif | 0,56430139 |
| 3A_20x4.tif | 0,06630444 |
| 3A_20x5.tif | 0,68994894 |
| 3A_20x6.tif | 0,75150218 |
| 3A_20x7.tif | 0,4623615 |
| 3A_20x8.tif | 0,69669502 |
| 3A_20x9.tif | 0,96127908 |
| 3A_20x10.tif | 0,3133615 |
| 3A_20x11.tif | 0,23792714 |
| 3A_20x12.tif | 0,78871914 |
| 3A_20x13.tif | 0,60951539 |
| 3A_20x14.tif | 0,96648975 |
| | |
| | |
| 3B_20x.tif | 0,10804918 |
| 3B_20x1.tif | 0,94447023 |
| 3B_20x2.tif | 0,3176632 |
| 3B_20x3.tif | 0,71269168 |
| 3B_20x4.tif | 0,22274158 |
| 3B_20x5.tif | 0,92355894 |
| 3B_20x6.tif | 0,89854967 |
| 3B_20x7.tif | 0,47337728 |
| 3B_20x8.tif | 0,69307488 |
| 3B_20x9.tif | 0,85437073 |
| 3B_20x10.tif | 0,29612929 |
| | |
| | |
| 3C_20x.tif | 0,5004046 |
| 3C_20x1.tif | 0,90973722 |
| 3C_20x2.tif | 0,52935024 |
| 3C_20x3.tif | 0,79690566 |
| 3C_20x4.tif | 0,96434123 |
| 3C_20x5.tif | 0,8706673 |
| 3C_20x6.tif | 0,46155356 |
| 3C_20x7.tif | 0,44846202 |
| 3C_20x8.tif | 0,06550212 |
| 3C_20x9.tif | 0,69820041 |
| 3C_20x10.tif | 0,49202932 |
| 3C_20x11.tif | 0,58497878 |
| 3C_20x12.tif | 0,98488355 |
| 3C_20x13.tif | 0,09768685 |
| | |
| | |
| 3D_20x.tif | 0,01341641 |

| | |
|---|---|
| 3D_20x1.tif | 0,51472582 |
| 3D_20x2.tif | 0,56409866 |
| 3D_20x3.tif | 0,5982465 |
| 3D_20x4.tif | 0,01393484 |
| 3D_20x5.tif | 0,9681526 |
| 3D_20x6.tif | 0,54210951 |

| | |
|---|---|
| 3E_20x.tif | 0,26810988 |
| 3E_20x1.tif | 0,4330762 |
| 3E_20x2.tif | 0,80530023 |
| 3E_20x3.tif | 0,30557961 |
| 3E_20x4.tif | 0,29245001 |
| 3E_20x5.tif | 0,79303482 |
| 3E_20x6.tif | 0,67862573 |
| 3E_20x7.tif | 0,58702738 |
| 3E_20x8.tif | 0,9310836 |

| | |
|---|---|
| 4A_20x.tif | 0,48585936 |
| 4A_20x1.tif | 0,11025996 |
| 4A_20x2.tif | 0,86658687 |
| 4A_20x3.tif | 0,19964219 |
| 4A_20x4.tif | 0,12703686 |
| 4A_20x5.tif | 0,12396311 |
| 4A_20x6.tif | 0,52336273 |
| 4A_20x7.tif | 0,88728064 |
| 4A_20x8.tif | 0,73442406 |
| 4A_20x9.tif | 0,53810344 |
| 4A_20x10.tif | 0,91672628 |
| 4A_20x11.tif | 0,63195072 |
| 4A_20x12.tif | 0,84828005 |
| 4A_20x13.tif | 0,61998042 |
| 4A_20x14.tif | 0,29885547 |
| 4A_20x15.tif | 0,16930838 |
| 4A_20x16.tif | 0,36389911 |
| 4A_20x17.tif | 0,9544052 |
| 4A_20x18.tif | 0,01128225 |
| 4A_20x19.tif | 0,46568972 |
| 4A_20x20.tif | 0,23400782 |
| 4A_20x21.tif | 0,13610695 |
| 4A_20x22.tif | 0,36608561 |
| 4A_20x23.tif | 0,06749688 |
| 4A_20x24.tif | 0,53588348 |
| 4A_20x25.tif | 0,30694647 |
| 4A_20x26.tif | 0,58724116 |

| | |
|---|---|
| 4A_20x27.tif | 0,06770417 |
| 4A_20x28.tif | 0,65596624 |
| 4A_20x29.tif | 0,09532105 |
| 4A_20x30.tif | 0,28145673 |
| 4A_20x31.tif | 0,26020314 |
| | |
| 4B_20x.tif | 0,82111918 |
| 4B_20x1.tif | 0,40417188 |
| 4B_20x2.tif | 0,28112445 |
| 4B_20x3.tif | 0,98800742 |
| 4B_20x4.tif | 0,03698866 |
| 4B_20x5.tif | 0,2813826 |
| | |
| 4C_20x.tif | 0,52263978 |
| 4C_20x1.tif | 0,86011765 |
| 4C_20x2.tif | 0,61368257 |
| 4C_20x3.tif | 0,19452005 |
| 4C_20x4.tif | 0,96645473 |
| 4C_20x5.tif | 0,27801318 |
| 4C_20x6.tif | 0,68649591 |
| 4C_20x7.tif | 0,42361784 |
| 4C_20x8.tif | 0,35689753 |
| 4C_20x9.tif | 0,39254538 |
| 4C_20x10.tif | 0,34649311 |
| | |
| 4D_20x.tif | 0,56620694 |
| 4D_20x1.tif | 0,90610182 |
| 4D_20x2.tif | 0,46515961 |
| 4D_20x3.tif | 0,37509728 |
| 4D_20x4.tif | 0,54912539 |
| 4D_20x5.tif | 0,39024258 |
| 4D_20x6.tif | 0,29807597 |
| 4D_20x7.tif | 0,7952535 |
| | |
| 4E_20x.tif | 0,7145975 |
| 4E_20x1.tif | 0,7543811 |
| 4E_20x2.tif | 0,21042892 |
| 4E_20x3.tif | 0,02817851 |
| 4E_20x4.tif | 0,26298865 |
| 4E_20x5.tif | 0,03603144 |
| 4E_20x6.tif | 0,8915083 |
| 4E_20x7.tif | 0,49637058 |

| | |
|---|---|
| 5A_20x.tif | 0,01882461 |
| 5A_20x1.tif | 0,36390848 |
| 5A_20x2.tif | 0,56029276 |
| 5A_20x3.tif | 0,46619027 |
| 5A_20x4.tif | 0,24093773 |
| 5A_20x5.tif | 0,34055672 |
| 5A_20x6.tif | 0,28853902 |
| 5A_20x7.tif | 0,49895617 |
| 5A_20x8.tif | 0,76604141 |
| 5A_20x9.tif | 0,35210768 |
| 5A_20x10.tif | 0,57656559 |
| 5A_20x11.tif | 0,14383439 |
| 5A_20x12.tif | 0,30303609 |
| 5A_20x13.tif | 0,67340408 |
| 5A_20x14.tif | 0,39075427 |
| 5A_20x15.tif | 0,26742239 |
| 5A_20x16.tif | 0,31721776 |
| 5A_20x17.tif | 0,67431732 |
| 5A_20x18.tif | 0,96562202 |

| | |
|---|---|
| 5B_20x.tif | 0,51385225 |
| 5B_20x1.tif | 0,67114669 |
| 5B_20x2.tif | 0,53016678 |
| 5B_20x3.tif | 0,65303045 |
| 5B_20x4.tif | 0,97717258 |
| 5B_20x5.tif | 0,82535438 |
| 5B_20x6.tif | 0,43620348 |
| 5B_20x7.tif | 0,14989448 |
| 5B_20x8.tif | 0,16129659 |
| 5B_20x9.tif | 0,56937446 |
| 5B_20x10.tif | 0,38952023 |

| | |
|---|---|
| 5C_20x.tif | 0,21558998 |
| 5C_20x1.tif | 0,86035174 |
| 5C_20x2.tif | 0,52735971 |
| 5C_20x3.tif | 0,37654724 |
| 5C_20x4.tif | 0,76652379 |
| 5C_20x5.tif | 0,82420861 |
| 5C_20x6.tif | 0,63182754 |

| | |
|---|---|
| 5D_20x.tif | 0,73670066 |

| | |
|---|---|
| 5D_20x1.tif | 0,64899919 |
| 5D_20x2.tif | 0,54047201 |
| 5D_20x3.tif | 0,7291635 |
| 5D_20x4.tif | 0,26370003 |
| 5D_20x5.tif | 0,82965039 |
| 5D_20x6.tif | 0,92069324 |
| 5D_20x7.tif | 0,97718453 |
| 5D_20x8.tif | 0,67408761 |
| 5D_20x9.tif | 0,00791868 |
| 5D_20x10.tif | 0,77583902 |

| | |
|---|---|
| 5E_20x.tif | 0,83185096 |
| 5E_20x1.tif | 0,82001046 |
| 5E_20x2.tif | 0,12036502 |
| 5E_20x3.tif | 0,65943988 |
| 5E_20x4.tif | 0,3857058 |
| 5E_20x5.tif | 0,90648769 |
| 5E_20x6.tif | 0,86046364 |

| | |
|---|---|
| 6A_20x.tif | 0,41965766 |
| 6A_20x1.tif | 0,13225357 |
| 6A_20x2.tif | 0,21427655 |
| 6A_20x3.tif | 0,45583361 |
| 6A_20x4.tif | 0,12503813 |
| 6A_20x5.tif | 0,92265345 |
| 6A_20x6.tif | 0,55819341 |
| 6A_20x7.tif | 0,71177726 |
| 6A_20x8.tif | 0,46180287 |
| 6A_20x9.tif | 0,44889137 |
| 6A_20x10.tif | 0,97040706 |
| 6A_20x11.tif | 0,01726664 |
| 6A_20x12.tif | 0,78025819 |
| 6A_20x13.tif | 0,04302517 |
| 6A_20x14.tif | 0,69814362 |
| 6A_20x15.tif | 0,9917051 |
| 6A_20x16.tif | 0,97970382 |
| 6A_20x17.tif | 0,19233188 |
| 6A_20x18.tif | 0,58594042 |
| 6A_20x19.tif | 0,90587789 |
| 6A_20x20.tif | 0,9926591 |
| 6A_20x21.tif | 0,35348618 |
| 6A_20x22.tif | 0,36016071 |

| | |
|---|---|
| 6B_20x.tif | 0,36521892 |
| 6B_20x1.tif | 0,34898361 |
| 6B_20x2.tif | 0,6860269 |
| 6B_20x3.tif | 0,21286208 |
| 6B_20x4.tif | 0,6359093 |
| 6B_20x5.tif | 0,96372579 |
| 6B_20x6.tif | 0,96314749 |
| 6B_20x7.tif | 0,08891089 |

| | |
|---|---|
| 6C_20x.tif | 0,94638414 |
| 6C_20x1.tif | 0,38663983 |
| 6C_20x2.tif | 0,49897867 |
| 6C_20x3.tif | 0,8095262 |
| 6C_20x4.tif | 0,49613327 |
| 6C_20x5.tif | 0,94361475 |
| 6C_20x6.tif | 0,83779363 |
| 6C_20x7.tif | 0,43048587 |
| 6C_20x8.tif | 0,77975638 |
| 6C_20x9.tif | 0,53813997 |
| 6C_20x10.tif | 0,79095068 |
| 6C_20x11.tif | 0,70032397 |

| | |
|---|---|
| 6D_20x.tif | 0,60681756 |
| 6D_20x1.tif | 0,72507349 |
| 6D_20x2.tif | 0,82160331 |
| 6D_20x3.tif | 0,99749136 |
| 6D_20x4.tif | 0,74302165 |
| 6D_20x5.tif | 0,7874366 |
| 6D_20x6.tif | 0,4109383 |
| 6D_20x7.tif | 0,15519785 |
| 6D_20x8.tif | 0,44907085 |
| 6D_20x9.tif | 0,0205053 |
| 6D_20x10.tif | 0,79344439 |
| 6D_20x11.tif | 0,67561437 |

| | |
|---|---|
| 6E_20x.tif | 0,54887991 |
| 6E_20x1.tif | 0,38226786 |
| 6E_20x2.tif | 0,43041012 |
| 6E_20x3.tif | 0,41142004 |
| 6E_20x4.tif | 0,10910987 |
| 6E_20x5.tif | 0,46509493 |
| 6E_20x6.tif | 0,07912333 |
| 6E_20x7.tif | 0,67177611 |

| | |
|---|---|
| 6E_20x8.tif | 0,89639601 |
| 6E_20x9.tif | 0,13103436 |
| 6E_20x10.tif | 0,75340454 |

| **animal 22** | |
|---|---|
| 2A_20x.tif | 0,85256212 |
| 2A_20x1.tif | 0,26200674 |
| 2A_20x2.tif | 0,95540584 |
| 2A_20x3.tif | 0,12103969 |
| 2A_20x4.tif | 0,66639468 |
| 2A_20x5.tif | 0,81930346 |
| 2A_20x6.tif | 0,8406557 |
| 2A_20x7.tif | 0,31589844 |
| 2A_20x8.tif | 0,92568693 |
| 2A_20x9.tif | 0,44351847 |
| 2A_20x10.tif | 0,51443729 |
| 2A_20x11.tif | 0,75418184 |
| 2A_20x12.tif | 0,29108818 |
| 2A_20x13.tif | 0,36814581 |
| 2A_20x14.tif | 0,93844391 |
| 2A_20x15.tif | 0,83954822 |
| 2A_20x16.tif | 0,27574297 |
| 2A_20x17.tif | 0,66028843 |
| 2A_20x18.tif | 0,84660632 |
| 2A_20x19.tif | 0,95568428 |
| 2A_20x20.tif | 0,51470282 |
| 2A_20x21.tif | 0,71900007 |
| 2A_20x22.tif | 0,52774191 |
| 2A_20x23.tif | 0,32780571 |
| 2A_20x24.tif | 0,99228102 |

| | |
|---|---|
| 2B_20x.tif | 0,01000643 |
| 2B_20x1.tif | 0,36838764 |
| 2B_20x2.tif | 0,90038889 |
| 2B_20x3.tif | 0,19610025 |
| 2B_20x4.tif | 0,20699592 |
| 2B_20x5.tif | 0,05305463 |
| 2B_20x6.tif | 0,41156837 |
| 2B_20x7.tif | 0,55192827 |
| 2B_20x8.tif | 0,47109458 |
| 2B_20x9.tif | 0,87494333 |
| 2B_20x10.tif | 0,253085 |

| | |
|---|---|
| 2C_20x.tif | 0,59981836 |
| 2C_20x1.tif | 0,95851414 |
| 2C_20x2.tif | 0,51815114 |
| 2C_20x3.tif | 0,55329205 |
| 2C_20x4.tif | 0,65864656 |
| 2C_20x5.tif | 0,39152934 |
| 2C_20x6.tif | 0,72498634 |
| 2C_20x7.tif | 0,47759057 |
| 2C_20x8.tif | 0,40359914 |
| 2C_20x9.tif | 0,99156201 |
| 2C_20x10.tif | 0,55418093 |
| 2C_20x11.tif | 0,37111778 |
| 2C_20x12.tif | 0,91512304 |
| 2C_20x13.tif | 0,4713278 |
| 2C_20x14.tif | 0,19175743 |
| 2C_20x15.tif | 0,8498596 |

| | |
|---|---|
| 2D_20x.tif | 0,87261617 |
| 2D_20x1.tif | 0,0957797 |
| 2D_20x2.tif | 0,50801139 |
| 2D_20x3.tif | 0,47210445 |
| 2D_20x4.tif | 0,12704405 |
| 2D_20x5.tif | 0,13720814 |
| 2D_20x6.tif | 0,16872329 |
| 2D_20x7.tif | 0,16387818 |
| 2D_20x8.tif | 0,37442879 |
| 2D_20x9.tif | 0,15769278 |
| 2D_20x10.tif | 0,80940196 |

| | |
|---|---|
| 2E_20x.tif | 0,22939558 |
| 2E_20x1.tif | 0,87600078 |
| 2E_20x2.tif | 0,11767542 |
| 2E_20x3.tif | 0,79205756 |
| 2E_20x4.tif | 0,42046763 |
| 2E_20x5.tif | 0,7938069 |
| 2E_20x6.tif | 0,26183672 |
| 2E_20x7.tif | 0,80922154 |
| 2E_20x8.tif | 0,33275718 |
| 2E_20x9.tif | 0,33092047 |
| 2E_20x10.tif | 0,6068248 |
| 2E_20x11.tif | 0,62964745 |

| | |
|---|---|
| 3A_20x.tif | 0,32989511 |

| | |
|---|---|
| 3A_20x1.tif | 0,25263103 |
| 3A_20x2.tif | 0,38165891 |
| 3A_20x3.tif | 0,95644929 |
| 3A_20x4.tif | 0,54977222 |
| 3A_20x5.tif | 0,00912875 |
| 3A_20x6.tif | 0,08712594 |
| 3A_20x7.tif | 0,63335572 |
| 3A_20x8.tif | 0,50471446 |
| 3A_20x9.tif | 0,54861953 |
| 3A_20x10.tif | 0,38702763 |
| 3A_20x11.tif | 0,89407886 |
| 3A_20x12.tif | 0,27763958 |
| 3A_20x13.tif | 0,30209159 |
| 3A_20x14.tif | 0,09027121 |
| 3A_20x15.tif | 0,11131913 |
| 3A_20x16.tif | 0,93905667 |
| 3A_20x17.tif | 0,90343936 |
| 3A_20x18.tif | 0,46141376 |
| 3A_20x19.tif | 0,90118029 |
| 3A_20x20.tif | 0,60052633 |
| 3A_20x21.tif | 0,48967666 |
| 3A_20x22.tif | 0,27408627 |

| | |
|---|---|
| 3B_20x.tif | 0,62912418 |
| 3B_20x1.tif | 0,15556599 |
| 3B_20x2.tif | 0,57760584 |
| 3B_20x3.tif | 0,72234145 |
| 3B_20x4.tif | 0,46181442 |
| 3B_20x5.tif | 0,72489422 |
| 3B_20x6.tif | 0,25629863 |
| 3B_20x7.tif | 0,36343042 |
| 3B_20x8.tif | 0,65408165 |
| 3B_20x9.tif | 0,69740892 |
| 3B_20x10.tif | 0,026457 |
| 3B_20x11.tif | 0,65393466 |
| 3B_20x12.tif | 0,02125677 |
| 3B_20x13.tif | 0,62885838 |
| 3B_20x14.tif | 0,22517118 |
| 3B_20x15.tif | 0,36206244 |
| 3B_20x16.tif | 0,29734639 |
| 3B_20x17.tif | 0,02270917 |
| 3B_20x18.tif | 0,12639796 |

| | |
|---|---|
| 3C_20x.tif | 0,59656698 |

| | |
|---|---|
| 3C_20x1.tif | 0,8335249 |
| 3C_20x2.tif | 0,72056796 |
| 3C_20x3.tif | 0,86408354 |
| 3C_20x4.tif | 0,5050157 |
| 3C_20x5.tif | 0,27868644 |

| | |
|---|---|
| 3D_20x.tif | 0,70021153 |
| 3D_20x1.tif | 0,9079806 |
| 3D_20x2.tif | 0,00413849 |
| 3D_20x3.tif | 0,77062513 |
| 3D_20x4.tif | 0,70880354 |
| 3D_20x5.tif | 0,88258034 |
| 3D_20x6.tif | 0,04665058 |
| 3D_20x7.tif | 0,89514112 |
| 3D_20x8.tif | 0,05649545 |
| 3D_20x9.tif | 0,83843163 |
| 3D_20x10.tif | 0,64476483 |
| 3D_20x11.tif | 0,24163319 |
| 3D_20x12.tif | 0,01236251 |

| | |
|---|---|
| 3E_20x.tif | 0,30414387 |
| 3E_20x1.tif | 0,53373128 |
| 3E_20x2.tif | 0,72774523 |
| 3E_20x3.tif | 0,08718159 |
| 3E_20x4.tif | 0,845517 |
| 3E_20x5.tif | 0,12047458 |

| | |
|---|---|
| 4A_20x.tif | 0,49194891 |
| 4A_20x1.tif | 0,31932424 |
| 4A_20x2.tif | 0,12796069 |
| 4A_20x3.tif | 0,69038091 |
| 4A_20x4.tif | 0,06204868 |
| 4A_20x5.tif | 0,42405039 |
| 4A_20x6.tif | 0,09298485 |
| 4A_20x7.tif | 0,29487805 |
| 4A_20x8.tif | 0,6579647 |
| 4A_20x9.tif | 0,4301912 |
| 4A_20x10.tif | 0,92179116 |
| 4A_20x11.tif | 0,09842312 |
| 4A_20x12.tif | 0,53046747 |
| 4A_20x13.tif | 0,36586782 |
| 4A_20x14.tif | 0,54252669 |
| 4A_20x15.tif | 0,91103657 |

| | |
|---|---|
| 4A_20x16.tif | 0,88133137 |
| 4A_20x17.tif | 0,28777325 |
| 4A_20x18.tif | 0,09158731 |
| 4A_20x19.tif | 0,49132195 |
| 4A_20x20.tif | 0,7807704 |
| 4A_20x21.tif | 0,9130037 |
| 4A_20x22.tif | 0,43756221 |
| | |
| 4B_20x.tif | 0,70110356 |
| 4B_20x1.tif | 0,0302651 |
| 4B_20x2.tif | 0,40331821 |
| 4B_20x3.tif | 0,64784074 |
| 4B_20x4.tif | 0,01032904 |
| 4B_20x5.tif | 0,18998533 |
| 4B_20x6.tif | 0,78603064 |
| 4B_20x7.tif | 0,82063743 |
| 4B_20x8.tif | 0,59924927 |
| 4B_20x9.tif | 0,2199819 |
| 4B_20x10.tif | 0,49438523 |
| 4B_20x11.tif | 0,03977374 |
| 4B_20x12.tif | 0,69958499 |
| | |
| 4C_20x.tif | 0,70217088 |
| 4C_20x1.tif | 0,26544965 |
| 4C_20x2.tif | 0,87851566 |
| 4C_20x3.tif | 0,65939561 |
| 4C_20x4.tif | 0,25635024 |
| 4C_20x5.tif | 0,45561206 |
| 4C_20x6.tif | 0,68363336 |
| 4C_20x7.tif | 0,07687108 |
| | |
| 4D_20x.tif | 0,44175304 |
| 4D_20x1.tif | 0,4100458 |
| 4D_20x2.tif | 0,91478191 |
| 4D_20x3.tif | 0,3071812 |
| 4D_20x4.tif | 0,14823967 |
| 4D_20x5.tif | 0,52554265 |
| 4D_20x6.tif | 0,96684233 |
| 4D_20x7.tif | 0,36015765 |
| | |
| 4E_20x.tif | 0,46535217 |
| 4E_20x1.tif | 0,85719632 |

| | |
|---|---|
| 4E_20x2.tif | 0,57235255 |
| 4E_20x3.tif | 0,72374739 |
| 4E_20x4.tif | 0,84815543 |
| 4E_20x5.tif | 0,33212312 |
| 4E_20x6.tif | 0,44979715 |
| 4E_20x7.tif | 0,37112233 |
| 4E_20x8.tif | 0,95238364 |
| 4E_20x9.tif | 0,84859451 |
| 4E_20x10.tif | 0,87918374 |
| 4E_20x11.tif | 0,23294957 |
| 4E_20x12.tif | 0,29738425 |

| | |
|---|---|
| 5A_20x.tif | 0,08196076 |
| 5A_20x1.tif | 0,62797546 |
| 5A_20x2.tif | 0,10406123 |
| 5A_20x3.tif | 0,50048632 |
| 5A_20x4.tif | 0,11185782 |
| 5A_20x5.tif | 0,86458752 |
| 5A_20x6.tif | 0,49940491 |
| 5A_20x7.tif | 0,36921901 |
| 5A_20x8.tif | 0,67626414 |
| 5A_20x9.tif | 0,7369438 |
| 5A_20x10.tif | 0,2217224 |
| 5A_20x11.tif | 0,91926805 |
| 5A_20x12.tif | 0,62056002 |
| 5A_20x13.tif | 0,61090763 |
| 5A_20x14.tif | 0,59144471 |
| 5A_20x15.tif | 0,62164455 |
| 5A_20x16.tif | 0,60011115 |
| 5A_20x17.tif | 0,49646731 |
| 5A_20x18.tif | 0,10197584 |
| 5A_20x19.tif | 0,56212137 |
| 5A_20x20.tif | 0,72631075 |
| 5A_20x21.tif | 0,74651057 |

| | |
|---|---|
| 5B_20x.tif | 0,29375472 |
| 5B_20x1.tif | 0,25943536 |
| 5B_20x2.tif | 0,23980031 |
| 5B_20x3.tif | 0,25570203 |
| 5B_20x4.tif | 0,09725523 |
| 5B_20x5.tif | 0,19944853 |
| 5B_20x6.tif | 0,67240471 |
| 5B_20x7.tif | 0,85916483 |
| 5B_20x8.tif | 0,02683364 |

| | |
|---|---|
| 5B_20x9.tif | 0,28256101 |
| 5B_20x10.tif | 0,44772422 |
| 5B_20x11.tif | 0,35651509 |
| 5B_20x12.tif | 0,9340431 |
| 5B_20x13.tif | 0,56343473 |
| 5B_20x14.tif | 0,17545897 |
| 5B_20x15.tif | 0,55304845 |
| 5B_20x16.tif | 0,21963062 |
| 5B_20x17.tif | 0,47271724 |
| 5B_20x18.tif | 0,49723172 |
| | |
| 5C_20x.tif | 0,87489941 |
| 5C_20x1.tif | 0,80494794 |
| 5C_20x2.tif | 0,83034453 |
| 5C_20x3.tif | 0,93136377 |
| 5C_20x4.tif | 0,03238973 |
| 5C_20x5.tif | 0,99966998 |
| 5C_20x6.tif | 0,33918177 |
| 5C_20x7.tif | 0,20974778 |
| 5C_20x8.tif | 0,17318128 |
| | |
| 5D_20x.tif | 0,66903676 |
| 5D_20x1.tif | 0,07441113 |
| 5D_20x2.tif | 0,63327493 |
| 5D_20x3.tif | 0,72098686 |
| 5D_20x4.tif | 0,89423167 |
| 5D_20x5.tif | 0,63290084 |
| 5D_20x6.tif | 0,17730569 |
| 5D_20x7.tif | 0,68309998 |
| 5D_20x8.tif | 0,00455615 |
| 5D_20x9.tif | 0,58682204 |
| 5D_20x10.tif | 0,31493342 |
| 5D_20x11.tif | 0,94325116 |
| 5D_20x12.tif | 0,67719583 |
| | |
| 5E_20x.tif | 0,04586664 |
| 5E_20x1.tif | 0,24910595 |
| 5E_20x2.tif | 0,47912889 |
| 5E_20x3.tif | 0,95730218 |
| 5E_20x4.tif | 0,44152976 |
| 5E_20x5.tif | 0,27919172 |
| 5E_20x6.tif | 0,71697684 |
| 5E_20x7.tif | 0,68900192 |

| | |
|---|---|
| 5E_20x8.tif | 0,14319228 |
| 5E_20x9.tif | 0,8631861 |
| | |
| 6A_20x.tif | 0,54850514 |
| 6A_20x1.tif | 0,43743478 |
| 6A_20x2.tif | 0,25655915 |
| 6A_20x3.tif | 0,79131532 |
| 6A_20x4.tif | 0,83549334 |
| 6A_20x5.tif | 0,11124576 |
| 6A_20x6.tif | 0,74828471 |
| 6A_20x7.tif | 0,89638838 |
| 6A_20x8.tif | 0,81228832 |
| 6A_20x9.tif | 0,90298256 |
| 6A_20x10.tif | 0,75424834 |
| 6A_20x11.tif | 0,79756052 |
| 6A_20x12.tif | 0,824165 |
| 6A_20x13.tif | 0,85560929 |
| 6A_20x14.tif | 0,40241536 |
| 6A_20x15.tif | 0,9875193 |
| 6A_20x16.tif | 0,38343758 |
| 6A_20x17.tif | 0,6180925 |
| 6A_20x18.tif | 0,91081776 |
| | |
| 6B_20x.tif | 0,53805861 |
| 6B_20x1.tif | 0,27021691 |
| 6B_20x2.tif | 0,20419067 |
| 6B_20x3.tif | 0,54129279 |
| 6B_20x4.tif | 0,20291457 |
| 6B_20x5.tif | 0,49940334 |
| 6B_20x6.tif | 0,22379105 |
| 6B_20x7.tif | 0,9692505 |
| 6B_20x8.tif | 0,12177564 |
| 6B_20x9.tif | 0,39210107 |
| 6B_20x10.tif | 0,49618466 |
| 6B_20x11.tif | 0,85710575 |
| 6B_20x12.tif | 0,7589217 |
| 6B_20x13.tif | 0,9272263 |
| 6B_20x14.tif | 0,30839979 |
| 6B_20x15.tif | 0,1500004 |
| 6B_20x16.tif | 0,35374629 |
| 6B_20x17.tif | 0,71946155 |
| 6B_20x18.tif | 0,4291269 |
| 6B_20x19.tif | 0,52833119 |
| 6B_20x20.tif | 0,3525992 |

| | |
|---|---|
| 6B_20x21.tif | 0,24084304 |
| 6B_20x22.tif | 0,28103099 |
| | |
| 6C_20x.tif | 0,24416886 |
| 6C_20x1.tif | 0,27997345 |
| 6C_20x2.tif | 0,21074487 |
| 6C_20x3.tif | 0,05172532 |
| 6C_20x4.tif | 0,06207109 |
| 6C_20x5.tif | 0,93663902 |
| 6C_20x6.tif | 0,51906609 |
| 6C_20x7.tif | 0,18305921 |
| 6C_20x8.tif | 0,97433173 |
| 6C_20x9.tif | 0,94075496 |
| 6C_20x10.tif | 0,87509531 |
| | |
| 6D_20x.tif | 0,07428293 |
| 6D_20x1.tif | 0,51324193 |
| 6D_20x2.tif | 0,71843505 |
| 6D_20x3.tif | 0,35076097 |
| 6D_20x4.tif | 0,38804379 |
| 6D_20x5.tif | 0,80315012 |
| 6D_20x6.tif | 0,39908526 |
| 6D_20x7.tif | 0,76347306 |
| 6D_20x8.tif | 0,79127602 |
| 6D_20x9.tif | 0,0754658 |
| 6D_20x10.tif | 0,8225631 |
| | |
| 6E_20x.tif | 0,89183917 |
| 6E_20x1.tif | 0,75370304 |
| 6E_20x2.tif | 0,58569486 |
| 6E_20x3.tif | 0,26798074 |
| 6E_20x4.tif | 0,77329002 |
| 6E_20x5.tif | 0,57729839 |
| 6E_20x6.tif | 0,00927076 |
| 6E_20x7.tif | 0,63008141 |
| 6E_20x8.tif | 0,92337995 |
| 6E_20x9.tif | 0,98725 |
| 6E_20x10.tif | 0,17641052 |
| 6E_20x11.tif | 0,94631382 |
| | |
| **animal 33** | |
| 2A_20x.tif | 0,62259339 |

| | |
|---|---|
| 2A_20x1.tif | 0,3866612 |
| 2A_20x2.tif | 0,51453012 |
| 2A_20x3.tif | 0,8158849 |
| 2A_20x4.tif | 0,2953869 |

| | |
|---|---|
| 2B_20x.tif | 0,34951488 |
| 2B_20x1.tif | 0,28333709 |
| 2B_20x2.tif | 0,38519164 |
| 2B_20x3.tif | 0,85198384 |
| 2B_20x4.tif | 0,88132326 |
| 2B_20x5.tif | 0,09966457 |
| 2B_20x6.tif | 0,80724519 |
| 2B_20x7.tif | 0,53216144 |
| 2B_20x8.tif | 0,92599815 |
| 2B_20x9.tif | 0,18261274 |
| 2B_20x10.tif | 0,15225992 |
| 2B_20x11.tif | 0,8489563 |
| 2B_20x12.tif | 0,5084504 |
| 2B_20x13.tif | 0,46356842 |
| 2B_20x14.tif | 0,67049742 |
| 2B_20x15.tif | 0,28107304 |
| 2B_20x16.tif | 0,21087855 |
| 2B_20x17.tif | 0,4552293 |
| 2B_20x18.tif | 0,98319678 |
| 2B_20x19.tif | 0,5539091 |
| 2B_20x20.tif | 0,56902182 |
| 2B_20x21.tif | 0,87350339 |
| 2B_20x22.tif | 0,47386692 |
| 2B_20x23.tif | 0,38704455 |
| 2B_20x24.tif | 0,67250544 |

| | |
|---|---|
| 2C_20x.tif | 0,58447664 |
| 2C_20x1.tif | 0,43096849 |
| 2C_20x2.tif | 0,52461662 |
| 2C_20x3.tif | 0,42083506 |
| 2C_20x4.tif | 0,05003677 |
| 2C_20x5.tif | 0,71668498 |
| 2C_20x6.tif | 0,11799167 |
| 2C_20x7.tif | 0,95165994 |
| 2C_20x8.tif | 0,03421444 |
| 2C_20x9.tif | 0,98252992 |
| 2C_20x10.tif | 0,88738593 |

| | |
|---|---|
| 2D_20x.tif | 0,90041708 |
| 2D_20x1.tif | 0,02222034 |
| 2D_20x2.tif | 0,61918806 |
| 2D_20x3.tif | 0,48991917 |
| | |
| 2E_20x.tif | 0,22135769 |
| 2E_20x1.tif | 0,38424576 |
| 2E_20x2.tif | 0,76724045 |
| 2E_20x3.tif | 0,6964861 |
| 2E_20x4.tif | 0,03014897 |
| 2E_20x5.tif | 0,54512512 |
| 2E_20x6.tif | 0,90996501 |
| 2E_20x7.tif | 0,96830797 |
| | |
| 3A_20x.tif | 0,62747877 |
| 3A_20x1.tif | 0,63120117 |
| 3A_20x2.tif | 0,17924542 |
| 3A_20x3.tif | 0,92985949 |
| | |
| 3B_20x.tif | 0,17847272 |
| 3B_20x1.tif | 0,75390551 |
| 3B_20x2.tif | 0,78521788 |
| 3B_20x3.tif | 0,23503963 |
| 3B_20x4.tif | 0,06391869 |
| 3B_20x5.tif | 0,5372582 |
| 3B_20x6.tif | 0,54207169 |
| 3B_20x7.tif | 0,98381844 |
| 3B_20x8.tif | 0,35834439 |
| 3B_20x9.tif | 0,70209438 |
| | |
| 3C_20x.tif | 0,72530777 |
| 3C_20x1.tif | 0,26328186 |
| 3C_20x2.tif | 0,25232143 |
| 3C_20x3.tif | 0,152882 |
| 3C_20x4.tif | 0,77842302 |
| 3C_20x5.tif | 0,10545888 |
| 3C_20x6.tif | 0,30521897 |
| 3C_20x7.tif | 0,02021737 |
| 3C_20x8.tif | 0,43449929 |
| | |
| 3D_20x.tif | 0,80065904 |

| | |
|---|---|
| 3D_20x1.tif | 0,22875642 |
| 3D_20x2.tif | 0,32753928 |
| 3D_20x3.tif | 0,11224095 |
| 3D_20x4.tif | 0,54927387 |
| 3D_20x5.tif | 0,84345429 |
| 3D_20x6.tif | 0,89534466 |
| 3D_20x7.tif | 0,18009255 |
| 3D_20x8.tif | 0,30814627 |

| | |
|---|---|
| 3E_20x.tif | 0,40576971 |
| 3E_20x1.tif | 0,33042246 |
| 3E_20x2.tif | 0,74411016 |
| 3E_20x3.tif | 0,79075742 |
| 3E_20x4.tif | 0,95570748 |
| 3E_20x5.tif | 0,79757134 |
| 3E_20x6.tif | 0,34808073 |

| | |
|---|---|
| 4A_20x.tif | 0,31647314 |
| 4A_20x1.tif | 0,49666435 |
| 4A_20x2.tif | 0,56784696 |
| 4A_20x3.tif | 0,79491753 |
| 4A_20x4.tif | 0,88350546 |
| 4A_20x5.tif | 0,44601955 |
| 4A_20x6.tif | 0,16293706 |
| 4A_20x7.tif | 0,54073897 |
| 4A_20x8.tif | 0,75807536 |
| 4A_20x9.tif | 0,44214548 |
| 4A_20x10.tif | 0,47481882 |
| 4A_20x11.tif | 0,70839467 |
| 4A_20x12.tif | 0,24893608 |
| 4A_20x13.tif | 0,86184045 |
| 4A_20x14.tif | 0,39961574 |
| 4A_20x15.tif | 0,53996347 |
| 4A_20x16.tif | 0,65051163 |
| 4A_20x17.tif | 0,10012776 |
| 4A_20x18.tif | 0,33187812 |
| 4A_20x19.tif | 0,38167231 |
| 4A_20x20.tif | 0,57019285 |
| 4A_20x21.tif | 0,58709689 |
| 4A_20x22.tif | 0,59790597 |
| 4A_20x23.tif | 0,12055079 |
| 4A_20x24.tif | 0,26498957 |

4B_ (nd)

| | |
|---|---|
| 4C_20x.tif | 0,69456674 |
| 4C_20x1.tif | 0,8533721 |
| 4C_20x2.tif | 0,25795394 |
| 4C_20x3.tif | 0,75780493 |
| | |
| 4D_20x.tif | 0,29265475 |
| 4D_20x1.tif | 0,22866544 |
| 4D_20x2.tif | 0,64259507 |
| 4D_20x3.tif | 0,20545647 |
| 4D_20x4.tif | 0,31459374 |
| | |
| 4E_20x.tif | 0,43149691 |
| 4E_20x1.tif | 0,79858736 |
| 4E_20x2.tif | 0,02718561 |
| 4E_20x3.tif | 0,45784078 |
| 4E_20x4.tif | 0,60015486 |
| 4E_20x5.tif | 0,01636882 |
| 4E_20x6.tif | 0,41426366 |
| 4E_20x7.tif | 0,92185039 |
| | |
| 5A_20x.tif | 0,53523825 |
| 5A_20x1.tif | 0,10823442 |
| 5A_20x2.tif | 0,44107668 |
| 5A_20x3.tif | 0,84913719 |
| 5A_20x4.tif | 0,55301747 |
| 5A_20x5.tif | 0,08253637 |
| 5A_20x6.tif | 0,615596 |
| 5A_20x7.tif | 0,82795105 |
| 5A_20x8.tif | 0,18118777 |
| 5A_20x9.tif | 0,90989274 |
| 5A_20x10.tif | 0,17543581 |
| 5A_20x11.tif | 0,28826562 |
| 5A_20x12.tif | 0,06269983 |
| 5A_20x13.tif | 0,50449446 |
| | |
| 5B_20x.tif | 0,80897291 |
| 5B_20x1.tif | 0,70257121 |
| 5B_20x2.tif | 0,00951434 |

| | |
|---|---|
| 5C_20x.tif | 0,0348645 |
| 5C_20x1.tif | 0,30280729 |

| | |
|---|---|
| 5D_20x.tif | 0,1414877 |
| 5D_20x1.tif | 0,16156203 |
| 5D_20x2.tif | 0,01580895 |
| 5D_20x3.tif | 0,26253636 |

| | |
|---|---|
| 5E_20x.tif | 0,00493589 |
| 5E_20x1.tif | 0,53348936 |
| 5E_20x2.tif | 0,23300595 |
| 5E_20x3.tif | 0,77824375 |
| 5E_20x4.tif | 0,76058154 |
| 5E_20x5.tif | 0,97058071 |
| 5E_20x6.tif | 0,20760084 |

| | |
|---|---|
| 6A_20x.tif | 0,04437842 |
| 6A_20x1.tif | 0,41015095 |
| 6A_20x2.tif | 0,1329341 |
| 6A_20x3.tif | 0,42921093 |
| 6A_20x4.tif | 0,79076847 |
| 6A_20x5.tif | 0,4787452 |
| 6A_20x6.tif | 0,67204385 |
| 6A_20x7.tif | 0,60696934 |
| 6A_20x8.tif | 0,75687702 |

| | |
|---|---|
| 6B_20x.tif | 0,78471602 |
| 6B_20x1.tif | 0,60031491 |
| 6B_20x2.tif | 0,78818658 |
| 6B_20x3.tif | 0,54558662 |
| 6B_20x4.tif | 0,13016066 |
| 6B_20x5.tif | 0,87930918 |
| 6B_20x6.tif | 0,78873961 |
| 6B_20x7.tif | 0,60235296 |
| 6B_20x8.tif | 0,54339153 |
| 6B_20x9.tif | 0,92383299 |

| | |
|---|---|
| 6C_20x.tif | 0,1291511 |
| 6C_20x1.tif | 0,4293957 |
| 6C_20x2.tif | 0,63646025 |

| | |
|---|---|
| 6C_20x3.tif | 0,30062199 |
| 6C_20x4.tif | 0,66077421 |
| 6C_20x5.tif | 0,09269932 |
| | |
| 6D_20x.tif | 0,58815235 |
| 6D_20x1.tif | 0,97658635 |
| 6D_20x2.tif | 0,10217203 |
| 6D_20x3.tif | 0,95362175 |
| 6D_20x4.tif | 0,57076074 |
| 6D_20x5.tif | 0,50138425 |
| | |
| 6E_20x.tif | 0,0288182 |
| 6E_20x1.tif | 0,08291317 |
| 6E_20x2.tif | 0,50975333 |
| 6E_20x3.tif | 0,281384 |
| 6E_20x4.tif | 0,67798374 |
| 6E_20x5.tif | 0,58514751 |
| 6E_20x6.tif | 0,1439471 |
| | |
| **animal 34** | |
| 2A_20x.tif | 0,69819517 |
| 2A_20x1.tif | 0,15360959 |
| 2A_20x2.tif | 0,24026336 |
| 2A_20x3.tif | 0,45988318 |
| 2A_20x4.tif | 0,08574078 |
| 2A_20x5.tif | 0,81153236 |
| | |
| 2B_20x.tif | 0,45644672 |
| 2B_20x1.tif | 0,35307763 |
| 2B_20x2.tif | 0,23304289 |
| 2B_20x3.tif | 0,71254212 |
| 2B_20x4.tif | 0,44809643 |
| | |
| 2C_20x.tif | 0,67782578 |
| 2C_20x1.tif | 0,68888314 |
| | |
| 2D_20x.tif | 0,10858436 |
| 2D_20x1.tif | 0,13732172 |

| | |
|---|---|
| 2E_20x.tif | 0,73402903 |
| 2E_20x1.tif | 0,57186643 |
| 2E_20x2.tif | 0,15243146 |
| 2E_20x3.tif | 0,3518407 |
| 2E_20x4.tif | 0,58924034 |
| 2E_20x5.tif | 0,63552212 |
| 2E_20x6.tif | 0,11441074 |
| 2E_20x7.tif | 0,13077112 |
| 2E_20x8.tif | 0,51102634 |
| | |
| 3A_20x.tif | 0,42080164 |
| 3A_20x1.tif | 0,69915146 |
| 3A_20x2.tif | 0,28089545 |
| 3A_20x3.tif | 0,01602518 |
| 3A_20x4.tif | 0,32642849 |
| | |
| 3B_20x.tif | 0,35644728 |
| 3B_20x1.tif | 0,35045404 |
| 3B_20x2.tif | 0,51744002 |
| 3B_20x3.tif | 0,75903588 |
| | |
| 3C_20x.tif | 0,82187649 |
| 3C_20x1.tif | 0,68183429 |
| 3C_20x2.tif | 0,87999659 |
| 3C_20x3.tif | 0,51130437 |
| 3C_20x4.tif | 0,67576022 |
| 3C_20x5.tif | 0,3525648 |
| 3C_20x6.tif | 0,62787847 |
| 3C_20x7.tif | 0,40555101 |
| | |
| 3D_20x.tif | 0,10615549 |
| 3D_20x1.tif | 0,13130235 |
| | |
| 3E_20x.tif | 0,60817617 |
| | |
| 4A_20x.tif | 0,40952348 |
| 4A_20x1.tif | 0,62903904 |
| 4A_20x2.tif | 0,95297162 |
| 4A_20x3.tif | 0,81168671 |
| 4A_20x4.tif | 0,51466899 |

| | |
|---|---|
| 4A_20x5.tif | 0,29423061 |
| 4A_20x6.tif | 0,64474129 |
| 4A_20x7.tif | 0,46412145 |
| 4A_20x8.tif | 0,88267633 |
| | |
| 4B_20x.tif | 0,17251708 |
| 4B_20x1.tif | 0,98354362 |
| 4B_20x2.tif | 0,28162475 |
| 4B_20x3.tif | 0,43512545 |
| | |
| 4C_20x.tif | 0,87122772 |
| 4C_20x1.tif | 0,53508012 |
| | |
| 4D_20x.tif | 0,55940689 |
| | |
| 4E_20x.tif | 0,16144033 |
| 4E_20x1.tif | 0,16306787 |
| 4E_20x2.tif | 0,28307569 |
| 4E_20x3.tif | 0,88049277 |
| 4E_20x4.tif | 0,84384418 |
| | |
| 5A_20x.tif | 0,88307338 |
| 5A_20x1.tif | 0,94393594 |
| 5A_20x2.tif | 0,0372556 |
| 5A_20x3.tif | 0,04070731 |
| 5A_20x4.tif | 0,46850783 |
| 5A_20x5.tif | 0,48253393 |
| 5A_20x6.tif | 0,8233962 |
| 5A_20x7.tif | 0,74759683 |
| 5A_20x8.tif | 0,79117204 |
| 5A_20x9.tif | 0,96801037 |
| 5A_20x10.tif | 0,27278571 |
| 5A_20x11.tif | 0,0671523 |
| 5A_20x12.tif | 0,49044224 |
| 5A_20x13.tif | 0,58778464 |
| 5A_20x14.tif | 0,66263597 |
| 5A_20x15.tif | 0,95547049 |
| 5A_20x16.tif | 0,40122919 |
| 5A_20x17.tif | 0,11522632 |

| | |
|---|---|
| 5B_20x.tif | 0,26791536 |
| 5B_20x1.tif | 0,29909794 |
| 5B_20x2.tif | 0,50525837 |
| 5B_20x3.tif | 0,91429391 |
| 5B_20x4.tif | 0,34903048 |
| 5B_20x5.tif | 0,41652582 |
| 5B_20x6.tif | 0,39397155 |
| 5B_20x7.tif | 0,14719794 |
| | |
| 5C_20x.tif | 0,76778421 |
| 5C_20x1.tif | 0,65405336 |
| 5C_20x2.tif | 0,24900588 |
| 5C_20x3.tif | 0,68117986 |
| 5C_20x4.tif | 0,83717582 |
| 5C_20x5.tif | 0,56730909 |
| | |
| 5D_20x.tif | 0,42436742 |
| 5D_20x1.tif | 0,06896771 |
| 5D_20x2.tif | 0,70621716 |
| | |
| 5E_20x.tif | 0,3348882 |
| | |
| 6A_20x.tif | 0,01968846 |
| 6A_20x1.tif | 0,3784861 |
| 6A_20x2.tif | 0,38607344 |
| 6A_20x3.tif | 0,99298925 |
| 6A_20x4.tif | 0,17030639 |
| 6A_20x5.tif | 0,08565275 |
| 6A_20x6.tif | 0,90981541 |
| 6A_20x7.tif | 0,69133219 |
| 6A_20x8.tif | 0,35088174 |
| 6A_20x9.tif | 0,37591686 |
| 6A_20x10.tif | 0,08075453 |
| 6A_20x11.tif | 0,21308262 |
| 6A_20x12.tif | 0,1199813 |
| | |
| 6B_20x.tif | 0,30416734 |
| 6B_20x1.tif | 0,15807898 |
| 6B_20x2.tif | 0,15345389 |
| 6B_20x3.tif | 0,41163496 |
| 6B_20x4.tif | 0,61049708 |

| | |
|---|---|
| 6B_20x5.tif | 0,9279407 |
| 6B_20x6.tif | 0,25895512 |
| 6B_20x7.tif | 0,3725766 |
| | |
| | |
| 6C_20x.tif | 0,50185895 |
| 6C_20x1.tif | 0,8958717 |
| 6C_20x2.tif | 0,21521904 |
| 6C_20x3.tif | 0,50860941 |
| 6C_20x4.tif | 0,59103891 |
| 6C_20x5.tif | 0,21871309 |
| 6C_20x6.tif | 0,81378767 |
| | |
| | |
| 6D_20x.tif | 0,06718419 |
| | |
| | |
| 6E_20x.tif | 0,45850589 |
| 6E_20x1.tif | 0,91629355 |
| 6E_20x2.tif | 0,49877411 |
| 6E_20x3.tif | 0,0824787 |
| | |
| | |
| **animal 36** | |
| 2A_20x.tif | 0,88310489 |
| 2A_20x1.tif | 0,33144108 |
| 2A_20x2.tif | 0,88927268 |
| 2A_20x3.tif | 0,84776641 |
| | |
| | |
| 2B_20x.tif | 0,17009975 |
| 2B_20x1.tif | 0,63073485 |
| 2B_20x2.tif | 0,65669498 |
| 2B_20x3.tif | 0,18855646 |
| 2B_20x4.tif | 0,52402422 |
| 2B_20x5.tif | 0,71506684 |
| 2B_20x6.tif | 0,03329702 |
| 2B_20x7.tif | 0,82642699 |
| | |
| | |
| 2C_20x.tif | 0,82542391 |
| 2C_20x1.tif | 0,96713699 |
| 2C_20x2.tif | 0,4031316 |
| 2C_20x3.tif | 0,16530392 |
| 2C_20x4.tif | 0,88616679 |
| 2C_20x5.tif | 0,94734589 |

| | |
|---|---|
| 2C_20x6.tif | 0,46261087 |
| 2C_20x7.tif | 0,54318254 |
| | |
| 2D_20x.tif | 0,91017488 |
| 2D_20x1.tif | 0,35474665 |
| 2D_20x2.tif | 0,84431414 |
| 2D_20x3.tif | 0,63651136 |
| | |
| 2E_20x.tif | 0,03492028 |
| 2E_20x1.tif | 0,8812137 |
| 2E_20x2.tif | 0,63076274 |
| | |
| 3A_20x.tif | 0,59175791 |
| 3A_20x1.tif | 0,41700224 |
| 3A_20x2.tif | 0,12059787 |
| 3A_20x3.tif | 0,72050775 |
| 3A_20x4.tif | 0,09138671 |
| 3A_20x5.tif | 0,67525911 |
| 3A_20x6.tif | 0,40876504 |
| 3A_20x7.tif | 0,93561441 |
| 3A_20x8.tif | 0,66772871 |
| | |
| 3B_20x.tif | 0,44450643 |
| 3B_20x1.tif | 0,08370404 |
| 3B_20x2.tif | 0,87050982 |
| 3B_20x3.tif | 0,99889762 |
| 3B_20x4.tif | 0,3177642 |
| 3B_20x5.tif | 0,09766257 |
| 3B_20x6.tif | 0,70391174 |
| 3B_20x7.tif | 0,91070164 |
| 3B_20x8.tif | 0,33325635 |
| 3B_20x9.tif | 0,56423604 |
| | |
| 3C_20x.tif | 0,27873547 |
| 3C_20x1.tif | 0,71233782 |
| 3C_20x2.tif | 0,60682257 |
| 3C_20x3.tif | 0,91680763 |
| 3C_20x4.tif | 0,43935086 |
| 3C_20x5.tif | 0,70074007 |
| 3C_20x6.tif | 0,98639313 |
| 3C_20x7.tif | 0,37734353 |

| | |
|---|---|
| 3D_20x.tif | 0,11813105 |
| 3D_20x1.tif | 0,84465509 |
| 3D_20x2.tif | 0,27906488 |
| 3D_20x3.tif | 0,41510689 |
| 3D_20x4.tif | 0,76971662 |
| 3D_20x5.tif | 0,59290081 |
| 3D_20x6.tif | 0,84374314 |
| 3D_20x7.tif | 0,25411999 |

| | |
|---|---|
| 3E_20x.tif | 0,69562667 |
| 3E_20x1.tif | 0,88185189 |
| 3E_20x2.tif | 0,11059851 |
| 3E_20x3.tif | 0,07565457 |

| | |
|---|---|
| 4A_20x.tif | 0,90603129 |
| 4A_20x1.tif | 0,40555892 |
| 4A_20x2.tif | 0,8761565 |
| 4A_20x3.tif | 0,51623709 |
| 4A_20x4.tif | 0,22806314 |
| 4A_20x5.tif | 0,89836476 |
| 4A_20x6.tif | 0,55304785 |
| 4A_20x7.tif | 0,3890615 |
| 4A_20x8.tif | 0,20252603 |
| 4A_20x9.tif | 0,82671913 |
| 4A_20x10.tif | 0,02816504 |
| 4A_20x11.tif | 0,58663809 |

| | |
|---|---|
| 4B_20x.tif | 0,7347886 |
| 4B_20x1.tif | 0,82911794 |
| 4B_20x2.tif | 0,60772433 |
| 4B_20x3.tif | 0,38286298 |
| 4B_20x4.tif | 0,05669382 |

| | |
|---|---|
| 4C_20x.tif | 0,61747587 |
| 4C_20x1.tif | 0,48951195 |
| 4C_20x2.tif | 0,65983315 |
| 4C_20x3.tif | 0,25550704 |
| 4C_20x4.tif | 0,64771498 |
| 4C_20x5.tif | 0,06175536 |
| 4C_20x6.tif | 0,66882141 |

| | |
|---|---|
| 4C_20x7.tif | 0,73589609 |
| 4C_20x8.tif | 0,9563323 |
| 4C_20x9.tif | 0,2954388 |
| | |
| 4D_20x.tif | 0,65805596 |
| 4D_20x1.tif | 0,91470894 |
| 4D_20x2.tif | 0,01378721 |
| 4D_20x3.tif | 0,59377629 |
| 4D_20x4.tif | 0,37232013 |
| 4D_20x5.tif | 0,80785621 |
| | |
| 4E_20x.tif | 0,2124921 |
| 4E_20x1.tif | 0,03892546 |
| | |
| 5A_20x.tif | 0,82329259 |
| 5A_20x1.tif | 0,59589371 |
| 5A_20x2.tif | 0,79139661 |
| 5A_20x3.tif | 0,31863132 |
| 5A_20x4.tif | 0,71595829 |
| 5A_20x5.tif | 0,82345025 |
| 5A_20x6.tif | 0,44381822 |
| 5A_20x7.tif | 0,11025876 |
| 5A_20x8.tif | 0,97649125 |
| 5A_20x9.tif | 0,61791258 |
| 5A_20x10.tif | 0,18614716 |
| 5A_20x11.tif | 0,06235174 |
| 5A_20x12.tif | 0,35360924 |
| 5A_20x13.tif | 0,6943464 |
| 5A_20x14.tif | 0,10150989 |
| 5A_20x15.tif | 0,2974604 |
| 5A_20x16.tif | 0,23029129 |
| | |
| 5B_20x.tif | 0,02369083 |
| 5B_20x1.tif | 0,62338587 |
| 5B_20x2.tif | 0,1722499 |
| 5B_20x3.tif | 0,85259971 |
| 5B_20x4.tif | 0,6580062 |
| 5B_20x5.tif | 0,97530345 |
| 5B_20x6.tif | 0,1351595 |
| | |
| 5C_20x.tif | 0,39061977 |

| | |
|---|---|
| 5C_20x1.tif | 0,39732592 |
| 5C_20x2.tif | 0,1575187 |
| 5C_20x3.tif | 0,1862587 |
| | |
| | |
| 5D_20x.tif | 0,55329089 |
| | |
| | |
| 5E_20x.tif | 0,91798796 |
| 5E_20x1.tif | 0,89056495 |
| | |
| | |
| 6A_20x.tif | 0,51247613 |
| 6A_20x1.tif | 0,84659576 |
| 6A_20x2.tif | 0,21092445 |
| 6A_20x3.tif | 0,02798763 |
| 6A_20x4.tif | 0,81838936 |
| 6A_20x5.tif | 0,06146107 |
| 6A_20x6.tif | 0,23375821 |
| 6A_20x7.tif | 0,5048073 |
| 6A_20x8.tif | 0,80413686 |
| 6A_20x9.tif | 0,68900054 |
| 6A_20x10.tif | 0,01502225 |
| | |
| | |
| 6B_20x.tif | 0,7661083 |
| 6B_20x1.tif | 0,05103215 |
| 6B_20x2.tif | 0,25474079 |
| 6B_20x3.tif | 0,30411547 |
| 6B_20x4.tif | 0,52737876 |
| 6B_20x5.tif | 0,50088495 |
| 6B_20x6.tif | 0,39524369 |
| | |
| | |
| 6C_20x.tif | 0,22282987 |
| 6C_20x1.tif | 0,68895057 |
| 6C_20x2.tif | 0,26300705 |
| 6C_20x3.tif | 0,76556067 |
| 6C_20x4.tif | 0,22688256 |
| 6C_20x5.tif | 0,43263683 |
| 6C_20x6.tif | 0,90372048 |
| | |
| | |
| 6D_20x.tif | 0,81706022 |
| 6D_20x1.tif | 0,99929247 |

| | |
|---|---|
| 6E_20x.tif | 0,8483541 |
| 6E_20x1.tif | 0,6391733 |
| 6E_20x2.tif | 0,32080243 |
| 6E_20x3.tif | 0,33373019 |
| 6E_20x4.tif | 0,51740686 |
| 6E_20x5.tif | 0,21397342 |
| 6E_20x6.tif | 0,16220411 |
| 6E_20x7.tif | 0,9031918 |
| 6E_20x8.tif | 0,6139048 |

# Animal 13

Week: 3

Medium: A

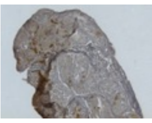
3A_20x1.tif

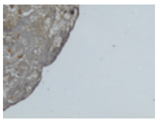
3A_20x3.tif

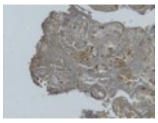
3A_20x5.tif

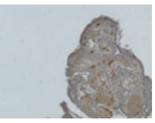
3A 20x6.tif

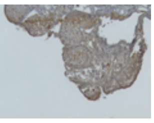
3A_20x10.tif

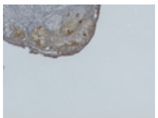
3A_20x11.tif

Medium: B

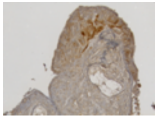
3B_20x4.tif

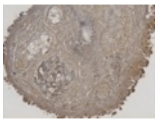
3B_20x5.tif

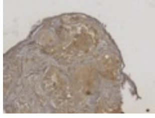
3B_20x6.tif

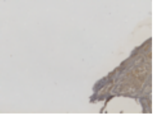
3B_20x7.tif

Medium: C

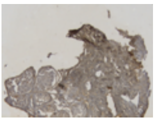
3C_20x.tif

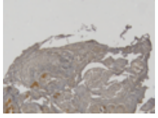
3C_20x2.tif

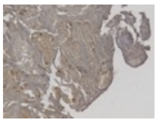
3C_20x3.tif

Medium: D

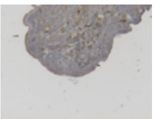
3D_20x1.tif

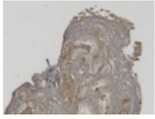
3D_20x2.tif

Medium: E

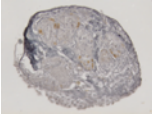
3E_20x.tif

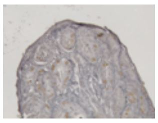
3E_20x2.tif

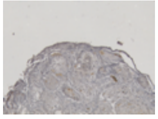
3E_20x5.tif

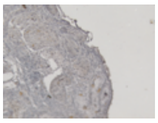
3E_20x7.tif

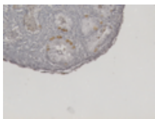
3E_20x9.tif

Week: 4

Medium: A

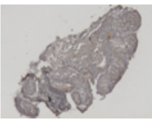
4A_20x.tif

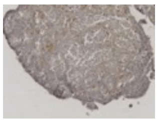
4A_20x1.tif

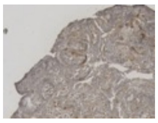
4A_20x2.tif

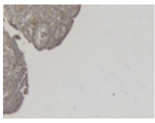
4A_20x4.tif

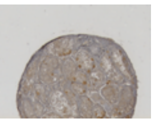
4A_20x5.tif

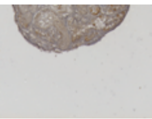
4A_20x6.tif

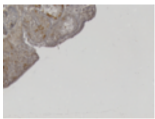
4A_20x11.tif

Medium: B

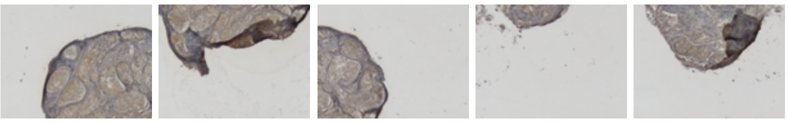

4B_20x.tif 4B_20x1.tif 4B_20x2.tif 4B_20x6.tif 4B_20x8.tif

Medium: C

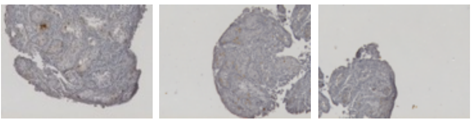

4C_20x.tif 4C_20x2.tif 4C_20x4.tif

Medium: D

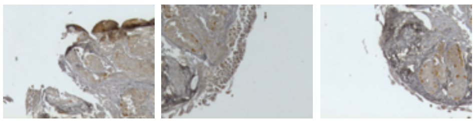

4D_20x1.tif 4D_20x3.tif 4D_20x4.tif

Medium: E

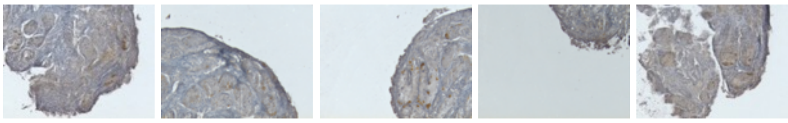

4E_20x.tif 4E_20x1.tif 4E_20x2.tif 4E_20x3.tif 4E_20x6.tif

<u>Week: 5</u>

Medium: A

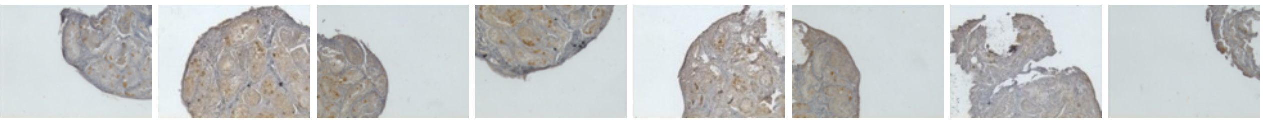

5A_20x3.tif 5A_20x4.tif 5A_20x5.tif 5A_20x6.tif 5A_20x8.tif 5A_20x9.tif 5A_20x12.tif 5A_20x14.tif

Medium: B

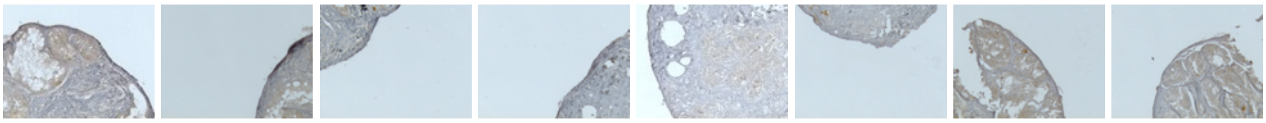

5B_20x.tif 5B_20x1.tif 5B_20x4.tif 5B_20x7.tif 5B_20x8.tif 5B_20x10.tif 5B_20x11.tif 5B_20x12.tif

Medium: C

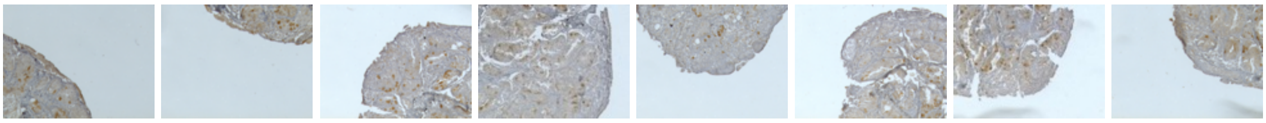

5C_20x1.tif 5C_20x5.tif 5C_20x7.tif 5C_20x9.tif 5C_20x12.tif 5C_20x13.tif 5C_20x15.tif 5C_20x16.tif

Medium: D

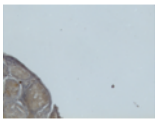
5D_20x3.tif

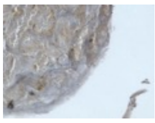
5D_20x4.tif

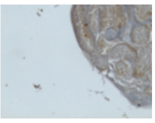
5D_20x6.tif

Medium: E

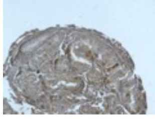
5E_20x.tif

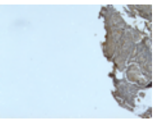
5E_20x2.tif

<u>Week: 6</u>

Medium: A

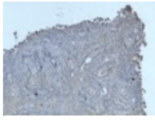
6A_20x.tif

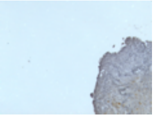
6A_20x1.tif

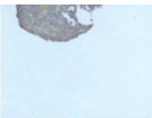
6A_20x7.tif

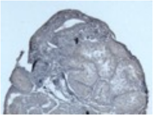
6A_20x8.tif

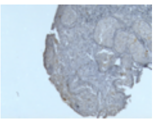
6A_20x10.tif

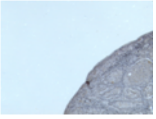
6A_20x11.tif

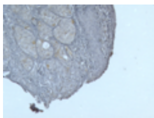
6A_20x13.tif

Medium: B

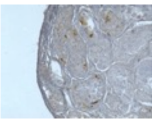
6B_20x1.tif

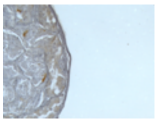
6B_20x2.tif

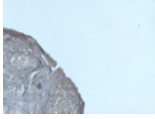
6B_20x5.tif

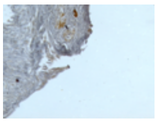
6B_20x6.tif

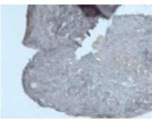
6B_20x7.tif

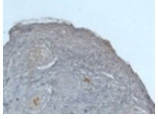
6B_20x9.tif

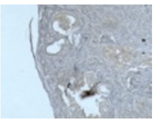
6B_20x11.tif

Medium: C

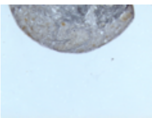
6C_20x1.tif

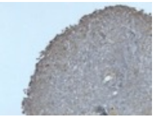
6C_20x3.tif

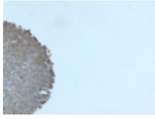
6C_20x4.tif

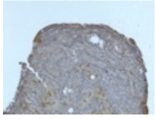
6C_20x5.tif

Medium: D

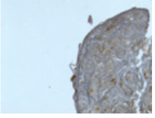
6D_20x.tif

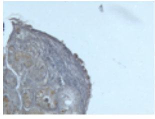
6D_20x1.tif

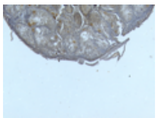
6D_20x2.tif

Medium: E

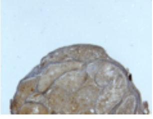
6E_20x2.tif

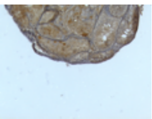
6E_20x3.tif

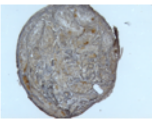
6E_20x4.tif

## Animal 14

<u>Week: 2</u>

Medium: A

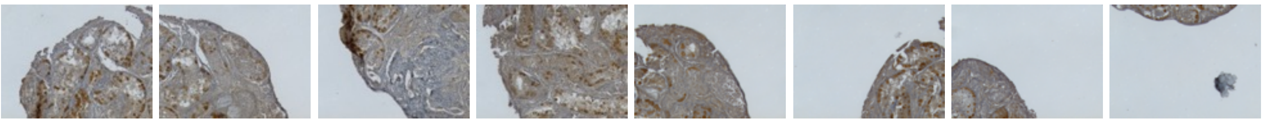

2A_20x.tif 2A_20x1.tif 2A_20x3.tif 2A_20x6.tif 2A_20x8.tif 2A_20x9.tif 2A_20x11.tif 2A_20x14.tif

Medium: B

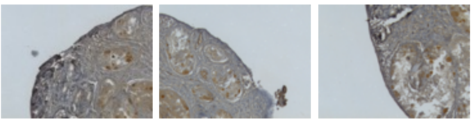

2B_20x.tif 2B_201x.tif 2B_20x3.tif

Medium: D

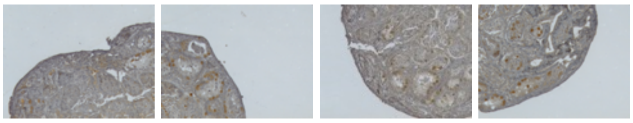

2D_20x.tif 2D_20x1.tif 2D_20x3.tif 2D_20x6.tif

Medium: E

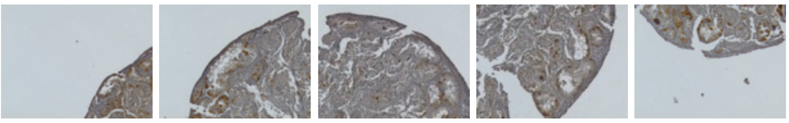

2E_20x3.tif 2E_20x4.tif 2E_20x5.tif 2E_20x6.tif 2E_20x8.tif

<u>Week: 3</u>

Medium: A

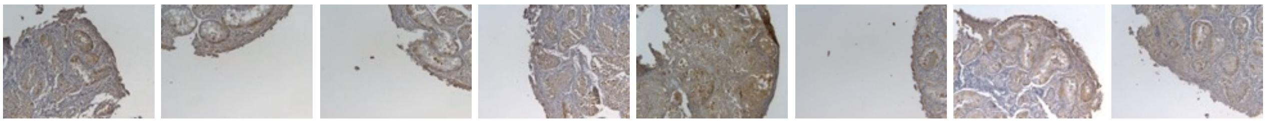

3A_20x.tif 3A_20x2.tif 3A_20x3.tif 3A_20x4.tif 3A_20x7.tif 3A_20x10.tif 3A_20x11.tif 3A_20x13.tif

Medium: B

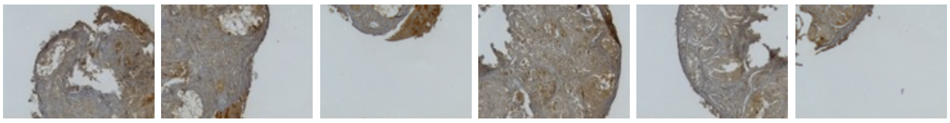

3B_20x.tif 3B_20x2.tif 3B_20x4.tif 3B_20x7.tif 3B_20x8.tif 3B_20x10.tif

Medium: C

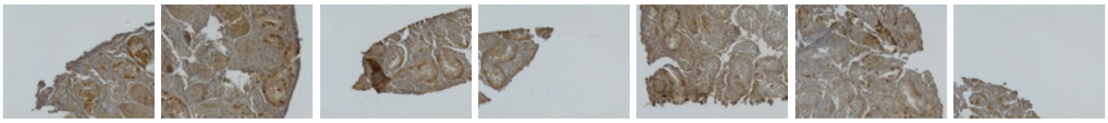

3C_20x.tif 3C_20x2.tif 3C_20x6.tif 3C_20x7.tif 3C_20x8.tif 3C_20x10.tif 3C_20x13.tif

Medium: D

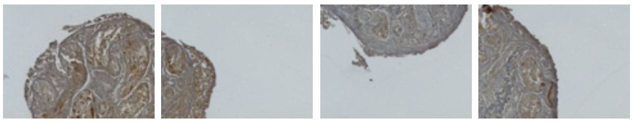

3D_20x.tif 3D_20x1.tif 3D_20x4.tif 3D_20x6.tif

Medium: E

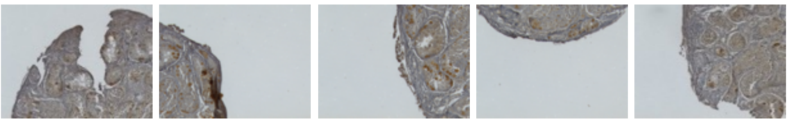

3E_20x.tif 3E_20x1.tif 3E_20x3.tif 3E_20x4.tif 3E_20x7.tif

## Week: 4

Medium: A

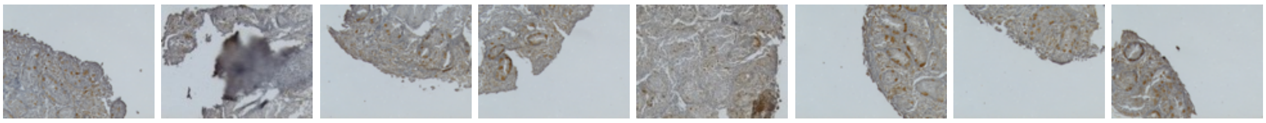

4A_20x1.tif 4A_20x3.tif 4A_20x4.tif 4A_20x5.tif 4A_20x14.tif 4A_20x15.tif 4A_20x16.tif 4A_20x18.tif

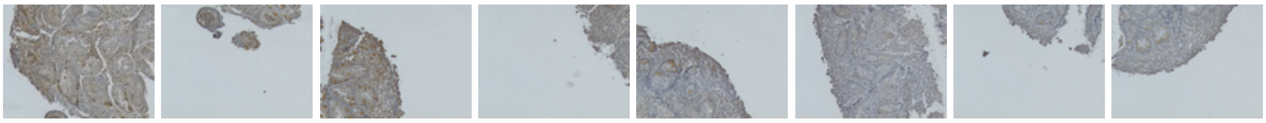

4A_20x20.tif 4A_20x21.tif 4A_20x23.tif 4A_20x25.tif 4A_20x27.tif 4A_20x29.tif 4A_20x30.tif 4A_20x31.tif

Medium: B

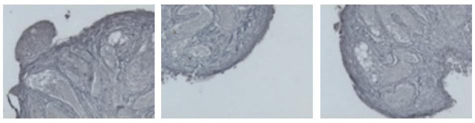

4B_20x2.tif 4B_20x4.tif 4B_20x5.tif

Medium: C

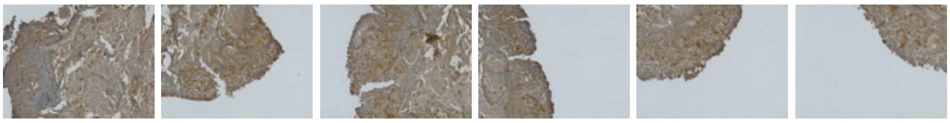

4C_20x3.tif 4C_20x5.tif 4C_20x7.tif 4C_20x8.tif 4C_20x9.tif 4C_20x10.tif

Medium: D

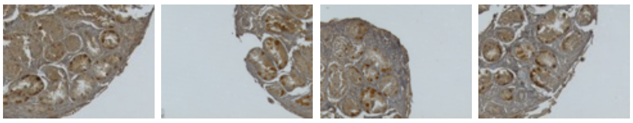

4D_20x2.tif 4D_20x3.tif 4D_20x5.tif 4D_20x6.tif

Medium: E

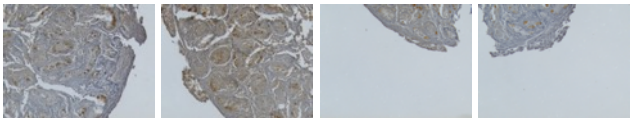

4E_20x2.tif 4E_20x3.tif 4E_20x4.tif 4E_20x5.tif

## Week: 5

### Medium: A

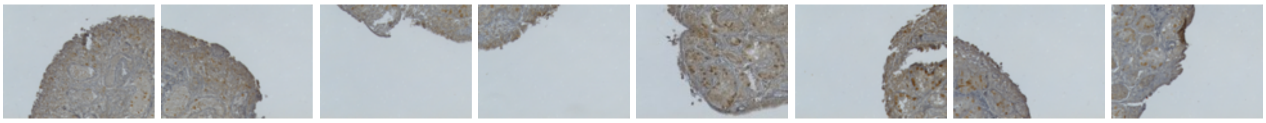

5A_20x.tif 5A_20x1.tif 5A_20x4.tif 5A_20x5.tif 5A_20x6.tif 5A_20x9.tif 5A_20x11.tif 5A_20x12.tif

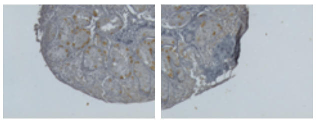

5A_20x15.tif 5A_20x16.tif

### Medium: B

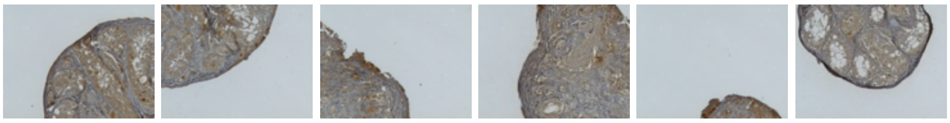

5B_20x.tif 5B_20x2.tif 5B_20x6.tif 5B_20x7.tif 5B_20x8.tif 5B_20x10.tif

### Medium: C

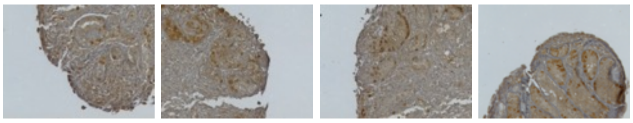

5C_20x.tif 5C_20x2.tif 5C_20x3.tif 5C_20x6.tif

### Medium: D

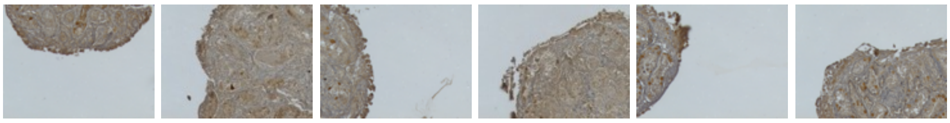

5D_20x.tif 5D_20x1.tif 5D_20x2.tif 5D_20x4.tif 5D_20x8.tif 5D_20x9.tif

### Medium: E

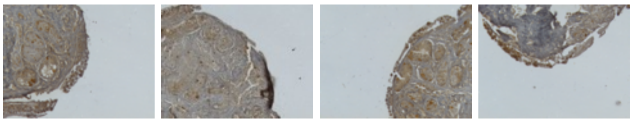

5E_20x1.tif 5E_20x2.tif 5E_20x3.tif 5E_20x4.tif

## Week: 6

### Medium: A

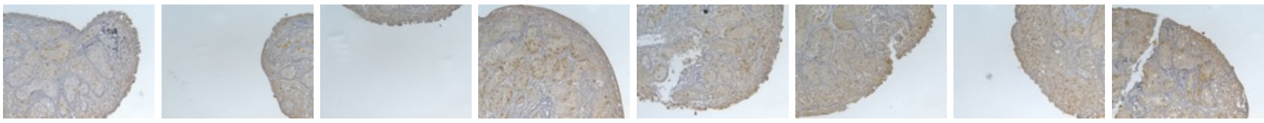

6A_20x.tif 6A_20x1.tif 6A_20x2.tif 6A_20x3.tif 6A_20x4.tif 6A_20x8.tif 6A_20x9.tif 6A_20x11.tif

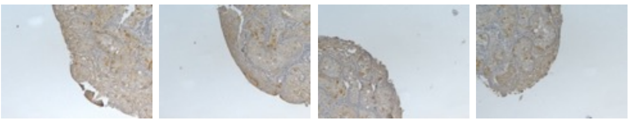

6A_20x13.tif 6A_20x17.tif 6A_20x21.tif 6A_20x22.tif

Medium: B

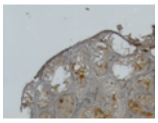
6B_20x.tif

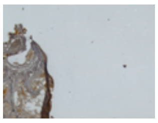
6B_20x1.tif

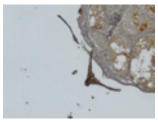
6B_20x3.tif

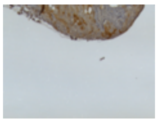
6B_20x7.tif

Medium: C

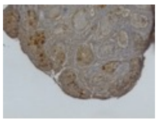
6C_20x1.tif

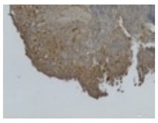
6C_20x2.tif

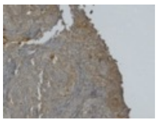
6C_20x4.tif

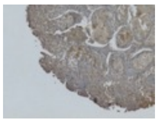
6C_20x7.tif

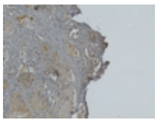
6C_20x9.tif

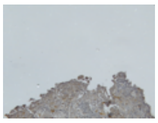
6C_20x11.tif

Medium: D

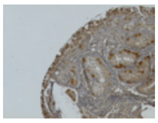
6D_20x.tif

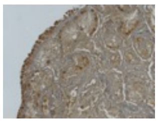
6D_20x6.tif

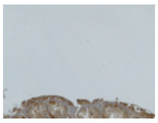
6D_20x7.tif

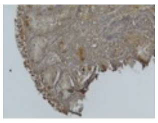
6D_20x8.tif

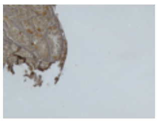
6D_20x9.tif

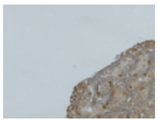
6D_20x11.tif

Medium: E

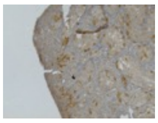
6E_20x1.tif

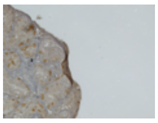
6E_20x2.tif

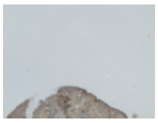
6E_20x3.tif

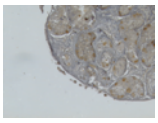
6E_20x4.tif

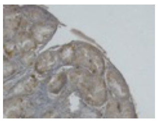
6E_20x6.tif

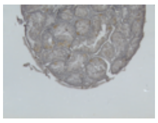
6E_20x9.tif

## Animal: 22

Week: 2

Medium: A

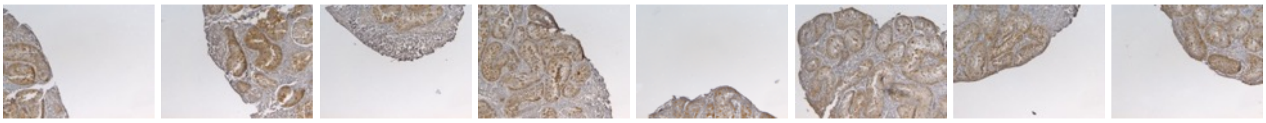

2A_20x1.tif 2A_20x3.tif 2A_20x4.tif 2A_20x7.tif 2A_20x9.tif 2A_20x10.tif 2A_20x12.tif 2A_20x13.tif

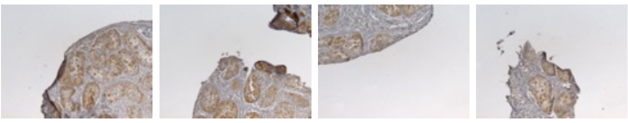

2A_20x17.tif 2A_20x20.tif 2A_20x22.tif 2A_20x23.tif

Medium: B

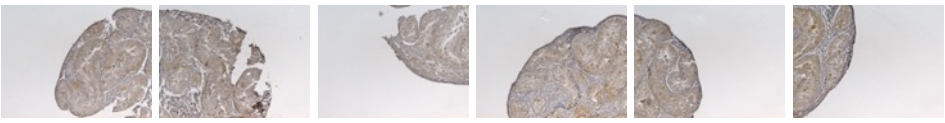

2B_20x.tif 2B_20x1.tif 2B_20x3.tif 2B_20x4.tif 2B_20x5.tif 2B_20x10.tif

Medium: C

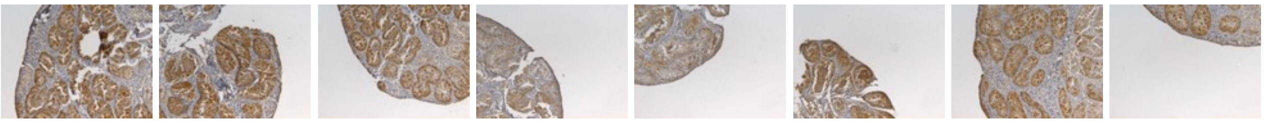

2C_20x2.tif 2C_20x3.tif 2C_20x5.tif 2C_20x7.tif 2C_20x8.tif 2C_20x11.tif 2C_20x13.tif 2C_20x14.tif

Medium: D

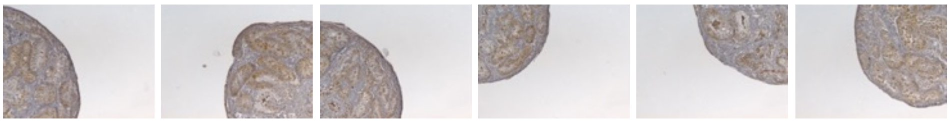

2D_20x1.tif 2D_20x4.tif 2D_20x5.tif 2D_20x6.tif 2D_20x7.tif 2D_20x9.tif

Medium: E

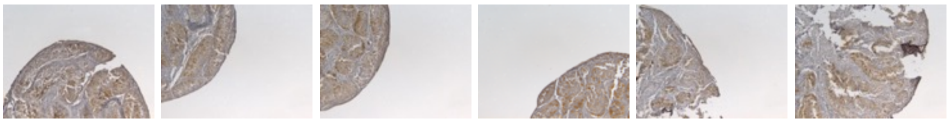

2E_20x.tif 2E_20x2.tif 2E_20x4.tif 2E_20x6.tif 2E_20x8.tif 2E_20x9.tif

Week: 3

Medium: A

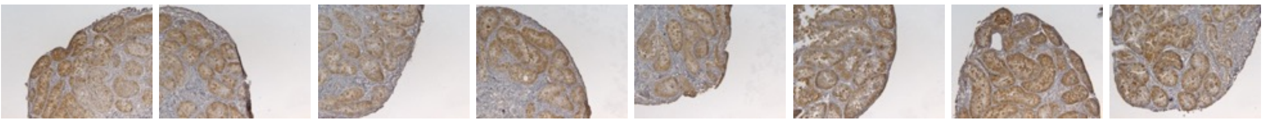

3A_20x.tif 3A_20x1.tif 3A_20x2.tif 3A_20x5.tif 3A_20x6.tif 3A_20x10.tif 3A_20x12.tif 3A_20x13.tif

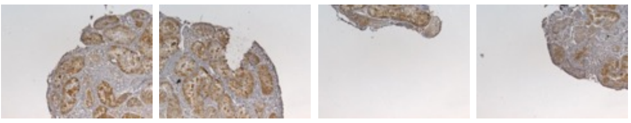

3A_20x14.tif 3A_20x15.tif 3A_20x18.tif 3A_20x22.tif

Medium: B

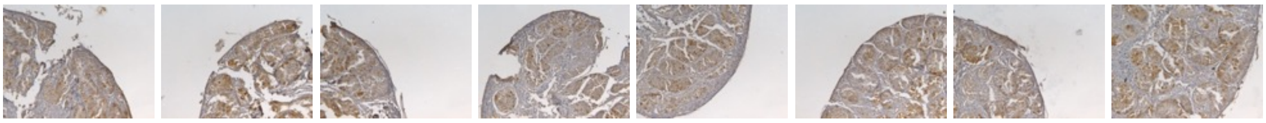

3B_20x1.tif 3B_20x6.tif 3B_20x7.tif 3B_20x10.tif 3B_20x12.tif 3B_20x14.tif 3B_20x15.tif 3B_20x16.tif

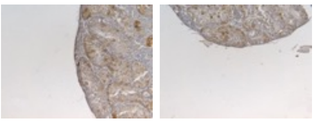

3B_20x17.tif 3B_20x18.tif

Medium: C

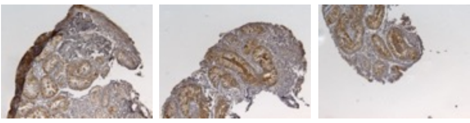

3C_20x.tif 3C_20x4.tif 3C_20x5.tif

Medium: D

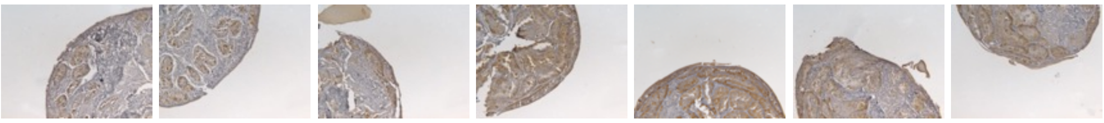

3D_20x.tif 3D_20x2.tif 3D_20x6.tif 3D_20x8.tif 3D_20x10.tif 3D_20x11.tif 3D_20x12.tif

Medium: E

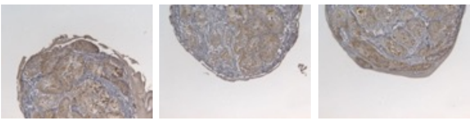

3E_20x.tif 3E_20x3.tif 3E_20x5.tif

<u>Week: 4</u>

Medium: A

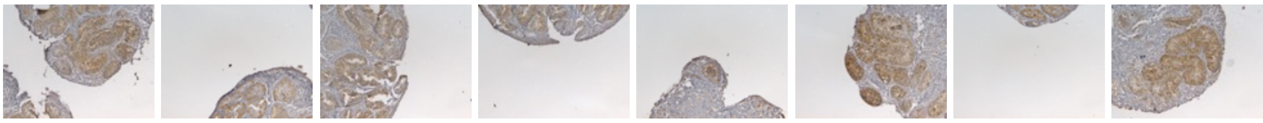

4A_20x1.tif 4A_20x2.tif 4A_20x4.tif 4A_20x5.tif 4A_20x6.tif 4A_20x7.tif 4A_20x9.tif 4A_20x11.tif

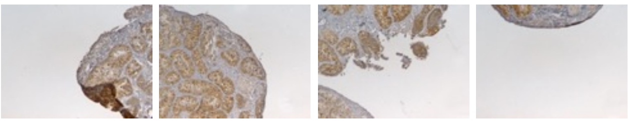

4A_20x13.tif 4A_20x17.tif 4A_20x18.tif 4A_20x22.tif

Medium: B

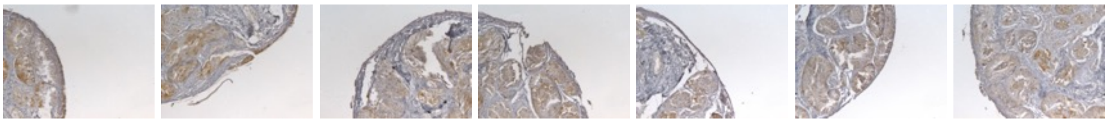

4B_20x1.tif 4B_20x2.tif 4B_20x4.tif 4B_20x5.tif 4B_20x9.tif 4B_20x10.tif 4B_20x11.tif

## Medium: C

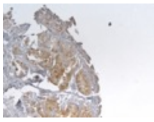
4C_20x1.tif

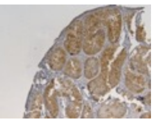
4C_20x4.tif

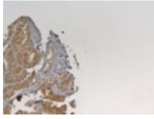
4C_20x5.tif

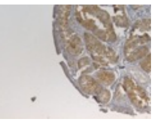
4C_20x7.tif

## Medium: D

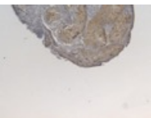
4D_20x1.tif

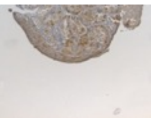
4D_20x3.tif

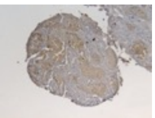
4D_20x4.tif

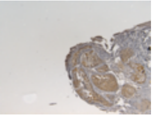
4D_20x7.tif

## Medium: E

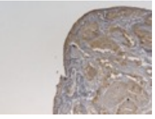
4E_20x.tif

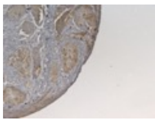
4E_20x2.tif

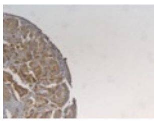
4E_20x5.tif

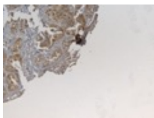
4E_20x6.tif

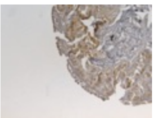
4E_20x7.tif

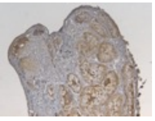
4E_20x11.tif

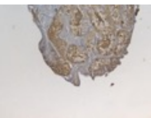
4E_20x12.tif

## <u>Week: 5</u>

## Medium: A

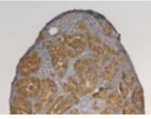
5A_20x.tif

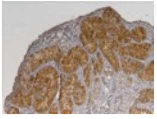
5A_20x2.tif

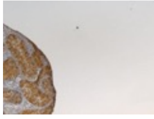
5A_20x3.tif

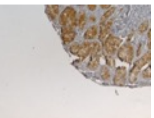
5A_20x4.tif

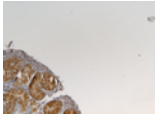
5A_20x6.tif

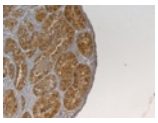
5A_20x7.tif

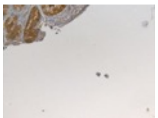
5A_20x10.tif

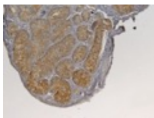
5A_20x14.tif

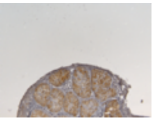
5A_20x17.tif

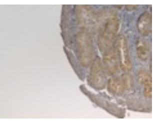
5A_20x18.tif

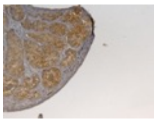
5A_20x19.tif

## Medium: B

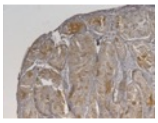
5B_20x.tif

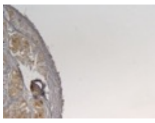
5B_20x1.tif

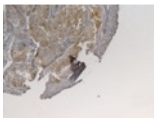
5B_20x2.tif

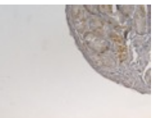
5B_20x3.tif

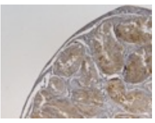
5B_20x4.tif

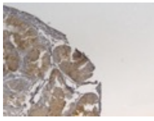
5B_20x5.tif

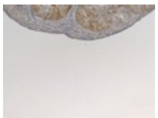
5B_20x8.tif

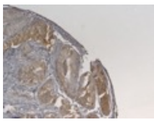
5B_20x14.tif

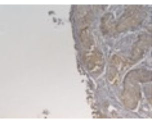
5B_20x16.tif

## Medium: C

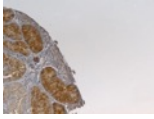
5C_20x1.tif

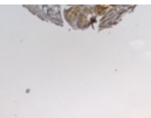
5C_20x4.tif

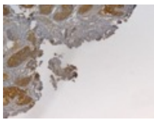
5C_20x6.tif

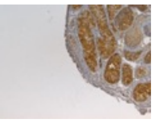
5C_20x7.tif

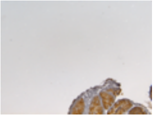
5C_20x8.tif

Medium: D

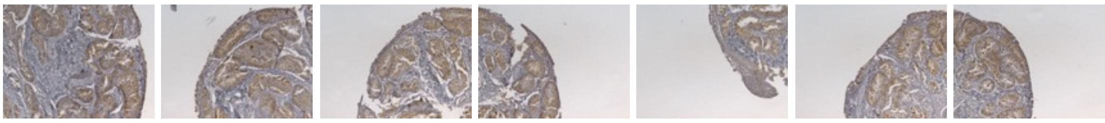

5D_20x1.tif 5D_20x2.tif 5D_20x5.tif 5D_20x6.tif 5D_20x8.tif 5D_20x9.tif 5D_20x10.tif

Medium: E

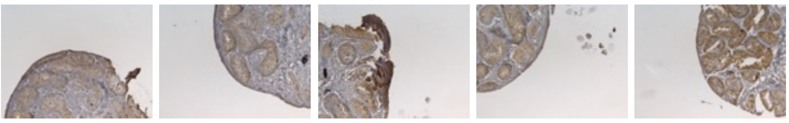

5E_20x.tif 5E_20x1.tif 5E_20x4.tif 5E_20x5.tif 5E_20x8.tif

<u>Week: 6</u>

Medium: A

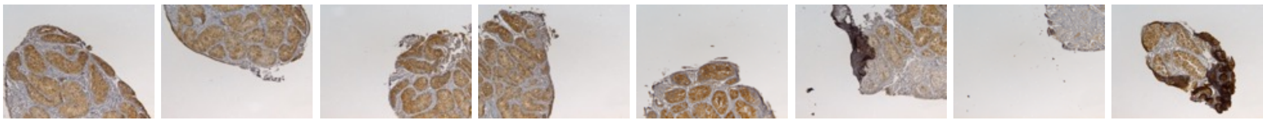

6A_20x.tif 6A_20x1.tif 6A_20x2.tif 6A_20x3.tif 6A_20x5.tif 6A_20x6.tif 6A_20x10.tif 6A_20x14.tif

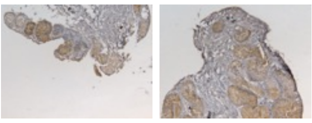

6A_20x16.tif 6A_20x17.tif

Medium: B

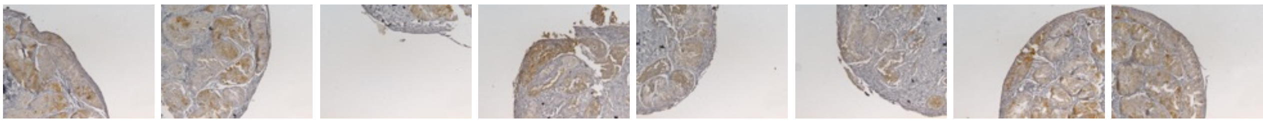

6B_20x1.tif 6B_20x2.tif 6B_20x4.tif 6B_20x6.tif 6B_20x8.tif 6B_20x9.tif 6B_20x14.tif 6B_20x15.tif

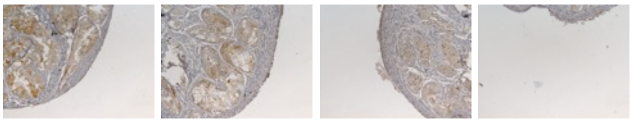

6B_20x16.tif 6B_20x20.tif 6B_20x21.tif 6B_20x22.tif

Medium: C

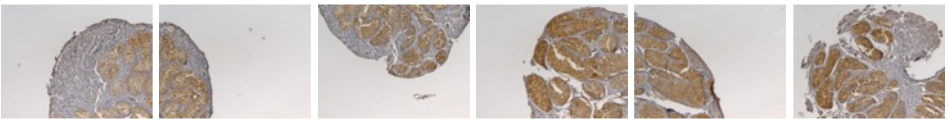

6C_20x.tif 6C_20x1.tif 6C_20x2.tif 6C_20x3.tif 6C_20x4.tif 6C_20x7.tif

Medium: D

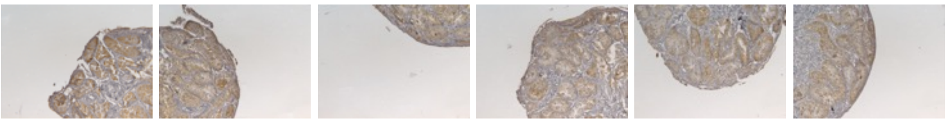

6D_20x.tif 6D_20x1.tif 6D_20x3.tif 6D_20x4.tif 6D_20x6.tif 6D_20x9.tif

Medium: E

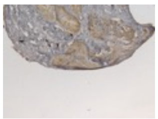
6E_20x2.tif

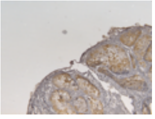
6E_20x3.tif

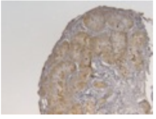
6E_20x5.tif

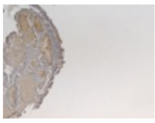
6E_20x6.tif

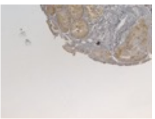
6E_20x7.tif

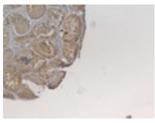
6E_20x10.tif

## Animal: 33

Week: 2

Medium: A

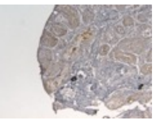 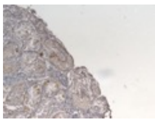 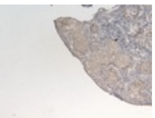

2A_20x1.tif 2A_20x2.tif 2A_20x4.tif

Medium: B

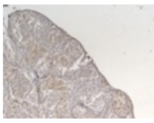 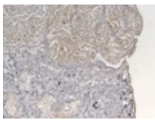 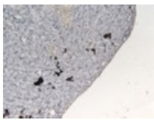 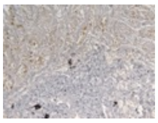 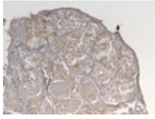 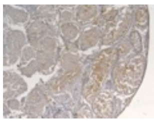 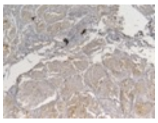 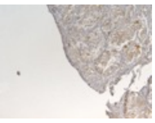

2B_20x.tif 2B_20x1.tif 2B_20x2.tif 2B_20x5.tif 2B_20x9.tif 2B_20x10.tif 2B_20x12.tif 2B_20x13.tif

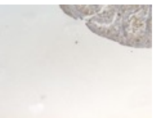 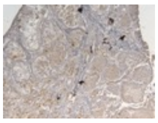 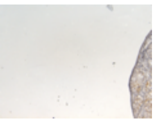 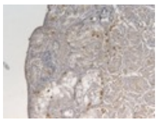 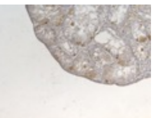

2B_20x15.tif 2B_20x16.tif 2B_20x17.tif 2B_20x22.tif 2B_20x23.tif

Medium: C

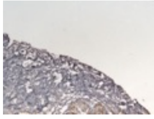 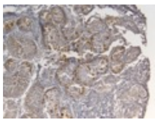 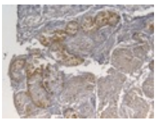 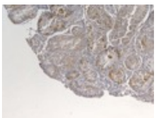 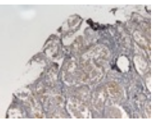 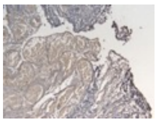

2C_20x1.tif 2C_20x2.tif 2C_20x3.tif 2C_20x4.tif 2C_20x6.tif 2C_20x8.tif

Medium: D

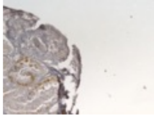 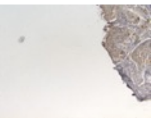

2D_20x1.tif 2D_20x3.tif

Medium: E

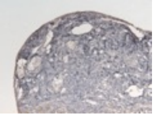 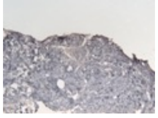 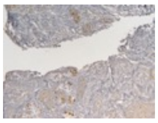 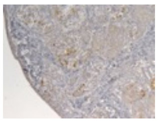

2E_20x.tif 2E_20x1.tif 2E_20x4.tif 2E_20x5.tif

Week: 3

Medium: A

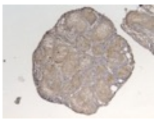 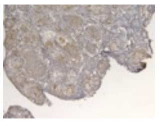

3A_20x.tif 3A_20x2.tif

Medium: B

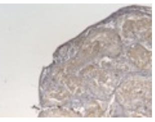
3B_20x.tif

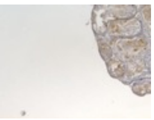
3B_20x3.tif

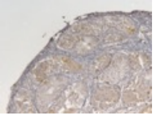
3B_20x4.tif

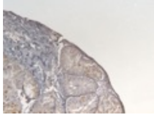
3B_20x5.tif

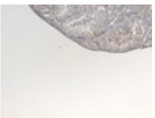
3B_20x8.tif

Medium: C

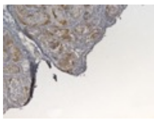
3C_20x1.tif

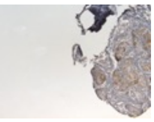
3C_20x2.tif

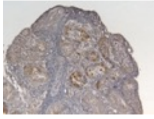
3C_20x3.tif

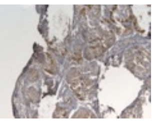
3C_20x5.tif

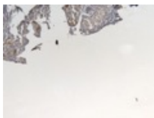
3C_20x7.tif

Medium: D

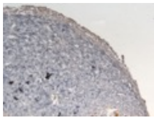
3D_20x1.tif

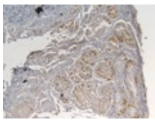
3D_20x2.tif

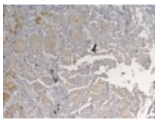
3D_20x3.tif

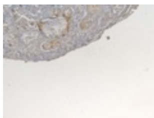
3D_20x7.tif

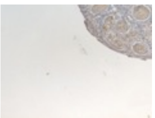
3D_20x8.tif

Medium: E

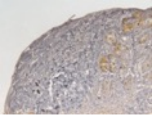
3E_20x.tif

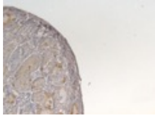
3E_20x1.tif

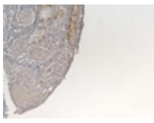
3E_20x2.tif

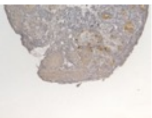
3E_20x6.tif

<u>Week: 4</u>

Medium: A

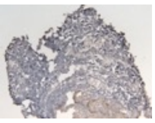
4A_20x.tif

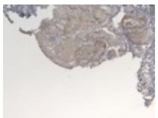
4A_20x1.tif

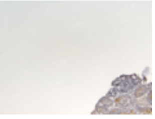
4A_20x5.tif

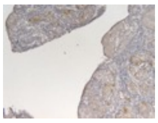
4A_20x6.tif

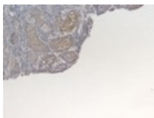
4A_20x9.tif

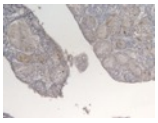
4A_20x10.tif

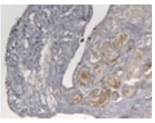
4A_20x12.tif

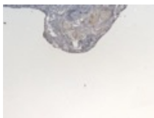
4A_20x14.tif

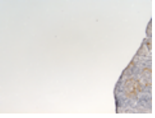
4A_20x17.tif

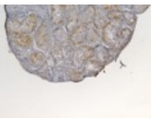
4A_20x18.tif

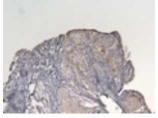
4A_20x19.tif

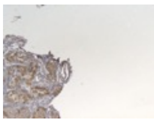
4A_20x23.tif

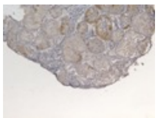
4A_20x24.tif

Medium: C

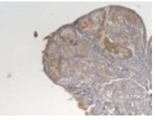
4C_20x.tif

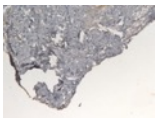
4C_20x2.tif

Medium: D

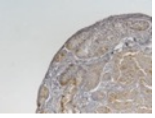
4D_20x.tif

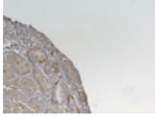
4D_20x1.tif

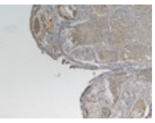
4D_20x3.tif

Medium: E

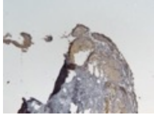
4E_20x.tif

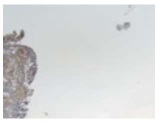
4E_20x2.tif

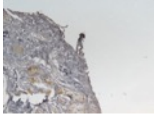
4E_20x5.tif

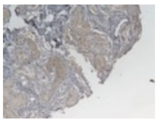
4E_20x6.tif

<u>Week: 5</u>

Medium: A

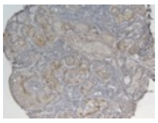
5A_20x1.tif

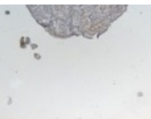
5A_20x2.tif

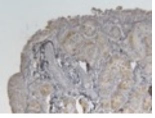
5A_20x5.tif

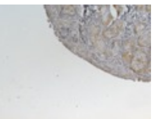
5A_20x8.tif

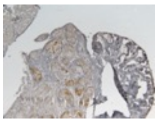
5A_20x10.tif

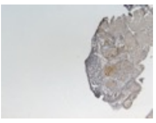
5A_20x11.tif

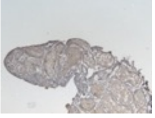
5A_20x12.tif

Medium: B

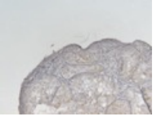
5B_20x1.tif

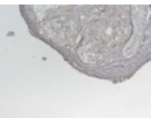
5B_20x2.tif

Medium: C

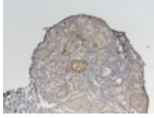
5C_20x0.tif

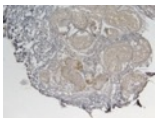
5C_20x1.tif

Medium: D

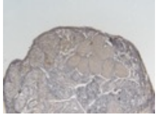
5D_20x.tif

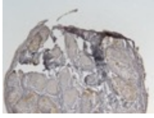
5D_20x2.tif

Medium: E

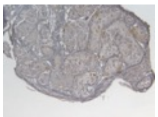
5E_20x.tif

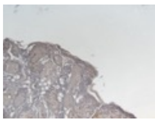
5E_20x1.tif

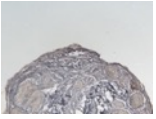
5E_20x2.tif

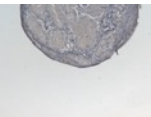
5E_20x6.tif

<u>Week: 6</u>

Medium: A

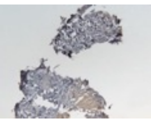
6A_20x.tif

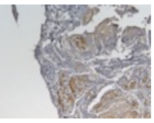
6A_20x1.tif

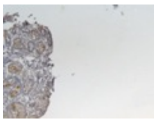
6A_20x2.tif

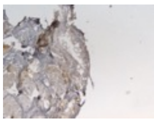
6A_20x3.tif

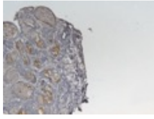
6A_20x5.tif

Medium: B

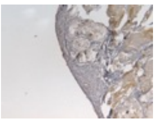
6B_20x1.tif

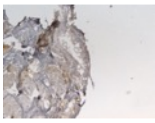
6B_20x3.tif

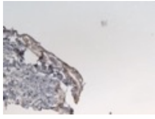
6B_20x4.tif

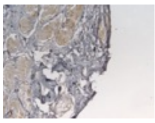
6B_20x7.tif

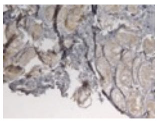
6B_20x8.tif

Medium: C

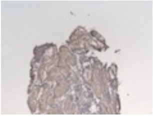
6C_20x.tif

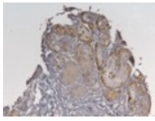
6C_20x3.tif

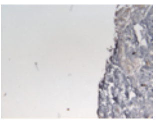
6C_20x5.tif

Medium: D

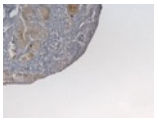
6D_20x2.tif

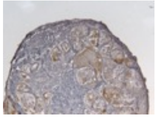
6D_20x4.tif

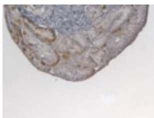
6D_20x5.tif

Medium: E

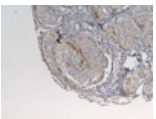
6E_20x.tif

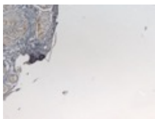
6E_20x1.tif

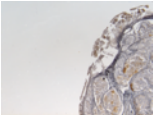
6E_20x3.tif

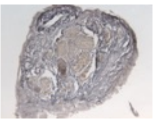
6E_20x6.tif

**Animal 34**

Week: 2

Medium: A

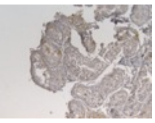 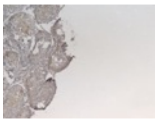 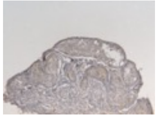

2A_20x1.tif 2A_20x2.tif 2A_20x4.tif

Medium: B

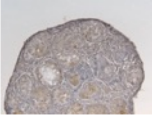 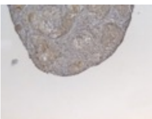 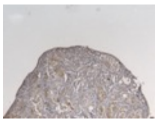

2B_20x0.tif 2B_20x1.tif 2B_20x2.tif

Medium: C

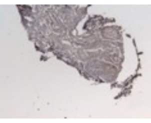 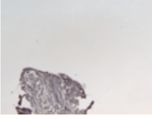

2C_20x.tif 2C_20x1.tif

Medium: D

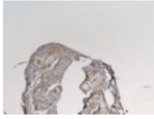 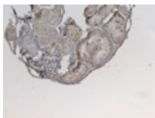

2D_20x.tif 2D_20x1.tif

Medium: E

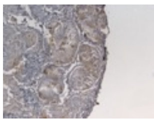 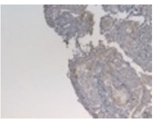 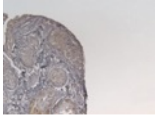 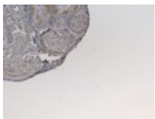 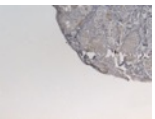

2E_20x2.tif 2E_20x3.tif 2E_20x6.tif 2E_20x7.tif 2E_20x8.tif

Week: 3

Medium: A

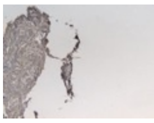 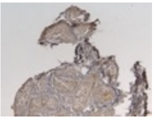 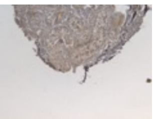

3A_20x2.tif 3A_20x3.tif 3A_20x4.tif

Medium: B

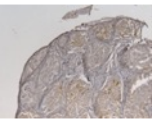 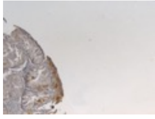

3B_20x.tif 3B_20x1.tif

Medium: C

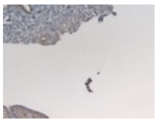
3C_20x3.tif

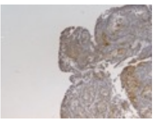
3C_20x5.tif

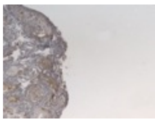
3C_20x6.tif

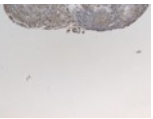
3C_20x7.tif

Medium: D

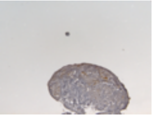
3D_20x.tif

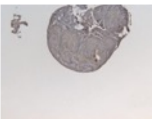
3D_20x1.tif

Medium: E

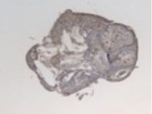
3E_20x.tif

Week: 4

Medium: A

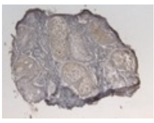
4A_20x.tif

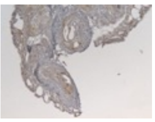
4A_20x1.tif

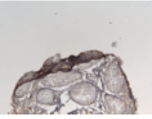
4A_20x4.tif

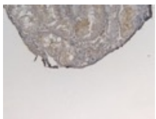
4A_20x5.tif

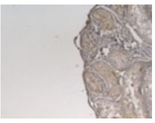
4A_20x7.tif

Medium: B

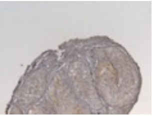
4B_20x.tif

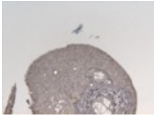
4B_20x2.tif

Medium: C

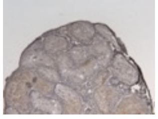
4C_20x.tif

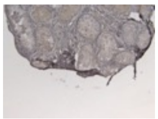
4C_20x1.tif

Medium: D

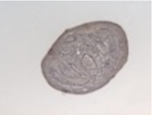
4D_20x.tif

Medium: E

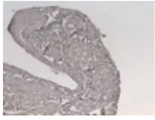
4E_20x.tif

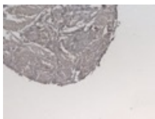
4E_20x1.tif

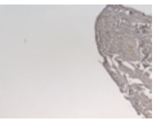
4E_20x2.tif

## Week: 5

### Medium: A

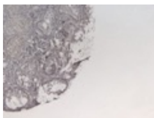
5A_20x2.tif

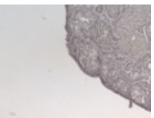
5A_20x3.tif

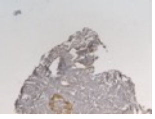
5A_20x4.tif

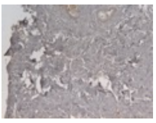
5A_20x5.tif

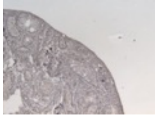
5A_20x10.tif

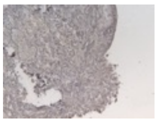
5A_20x11.tif

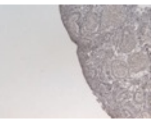
5A_20x12.tif

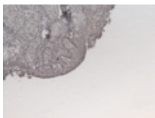
5A_20x16.tif

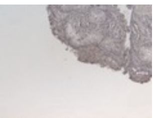
5A_20x17.tif

### Medium: B

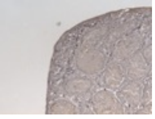
5B_20x.tif

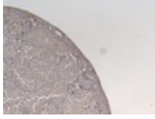
5B_20x1.tif

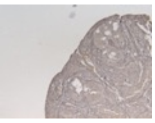
5B_20x4.tif

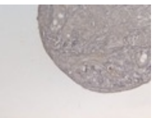
5B_20x7.tif

### Medium: C

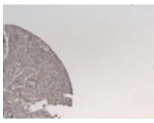
5C_20x1.tif

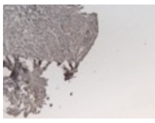
5C_20x2.tif

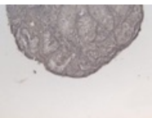
5C_20x5.tif

### Medium: D

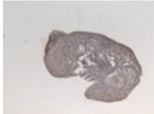
5D_20x.tif

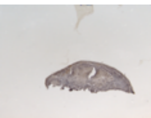
5D_20x1.tif

### Medium: E

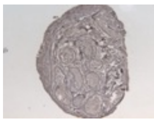
5E_20x.tif

## Week: 6

### Medium: A

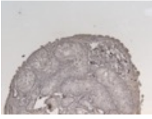
6A_20x.tif

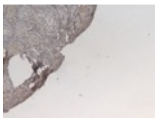
6A_20x4.tif

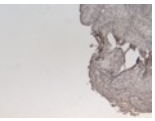
6A_20x5.tif

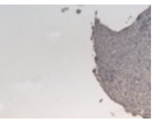
6A_20x8.tif

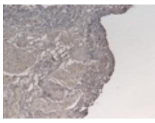
6A_20x10.tif

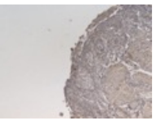
6A_20x11.tif

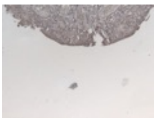
6A_20x12.tif

## Medium: B

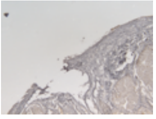
6B_20x.tif

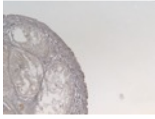
6B_20x1.tif

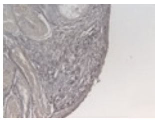
6B_20x2.tif

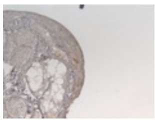
6B_20x6.tif

## Medium: C

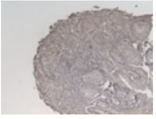
6C_20x.tif

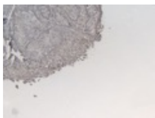
6C_20x2.tif

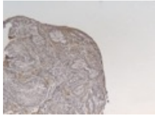
6C_20x3.tif

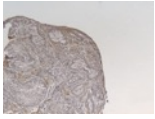
6C_20x5.tif

## Medium: D

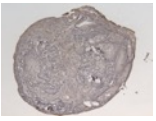
6D_20x.tif

## Medium: E

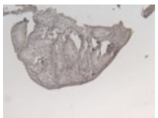
6E_20x.tif

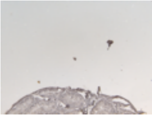
6E_20x3.tif

# Animal 36

## Week: 2

### Medium: A

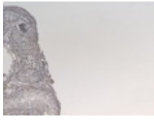 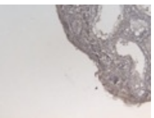

2A_20x1.tif 2A_20x3.tif

### Medium: B

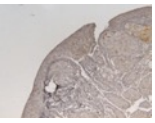 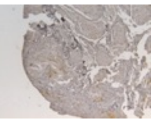 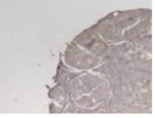 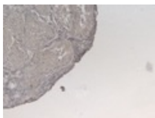

2B_20x.tif 2B_20x3.tif 2B_20x4.tif 2B_20x6.tif

### Medium: C

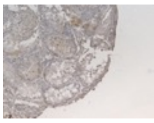 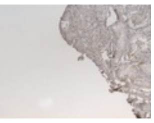 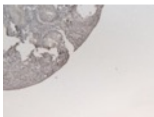 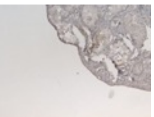

2C_20x2.tif 2C_20x3.tif 2C_20x6.tif 2C_20x7.tif

### Medium: D

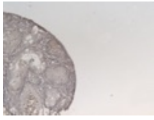 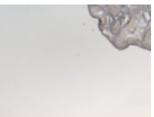

2D_20x1.tif 2D_20x3.tif

### Medium: E

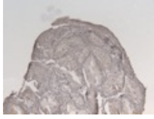 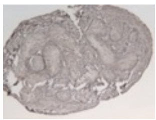

2E_20x.tif 2E_20x2.tif

## Week: 3

### Medium: A

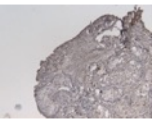 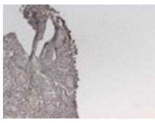 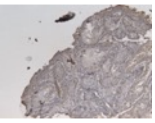 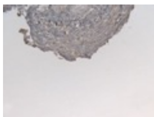

3A_20x.tif 3A_20x1.tif 3A_20x4.tif 3A_20x6.tif

## Medium: B

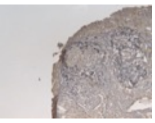
3B_20x.tif

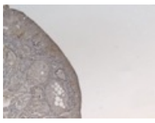
3B_20x1.tif

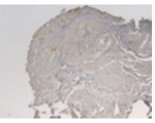
3B_20x4.tif

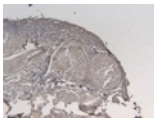
3B_20x5.tif

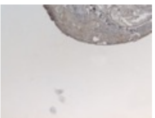
3B_20x8.tif

## Medium: C

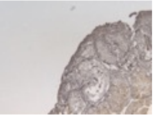
3C_20x.tif

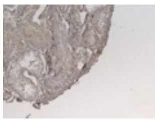
3C_20x2.tif

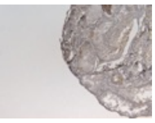
3C_20x4.tif

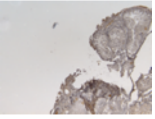
3C_20x7.tif

## Medium: D

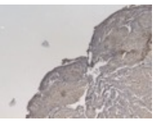
3D_20x.tif

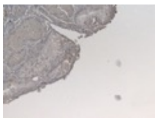
3D_20x2.tif

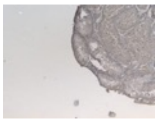
3D_20x3.tif

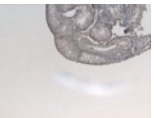
3D_20x7.tif

## Medium: E

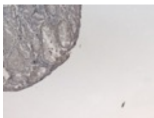
3E_20x2.tif

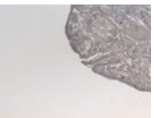
3E_20x3.tif

## <u>Week: 4</u>

## Medium: A

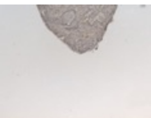
4A_20x1.tif

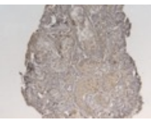
4A_20x3.tif

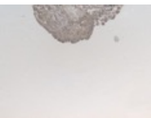
4A_20x4.tif

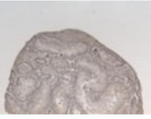
4A_20x7.tif

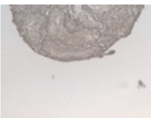
4A_20x8.tif

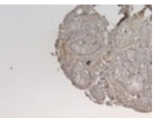
4A_20x10.tif

## Medium: B

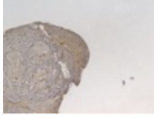
4B_20x2.tif

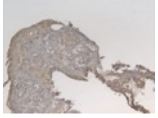
4B_20x3.tif

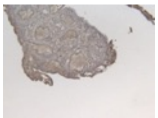
4B_20x4.tif

## Medium: C

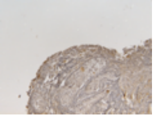
4C_20x.tif

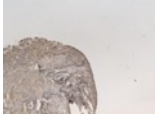
4C_20x1.tif

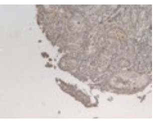
4C_20x3.tif

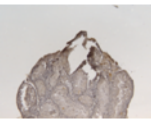
4C_20x5.tif

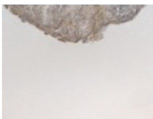
4C_20x9.tif

## Medium: D

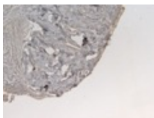
4D_20x2.tif

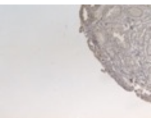
4D_20x3.tif

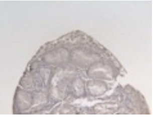
4D_20x4.tif

## Medium: E

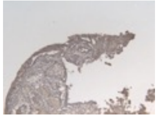
4E_20x.tif

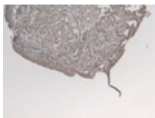
4E_20x1.tif

## Week: 5

## Medium: A

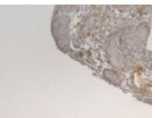
5A_20x3.tif

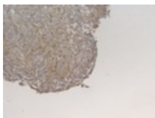
5A_20x6.tif

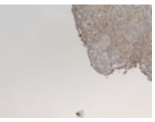
5A_20x7.tif

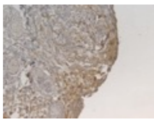
5A_20x10.tif

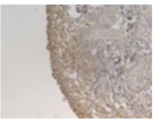
5A_20x11.tif

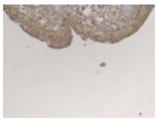
5A_20x12.tif

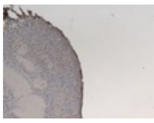
5A_20x14.tif

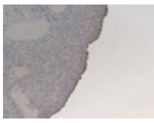
5A_20x15.tif

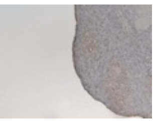
5A_20x16.tif

## Medium: B

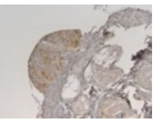
5B_20x.tif

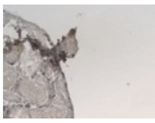
5B_20x1.tif

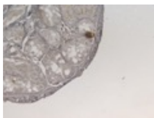
5B_20x2.tif

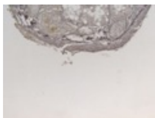
5B_20x6.tif

## Medium: C

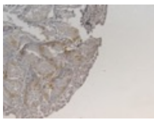
5C_20x2.tif

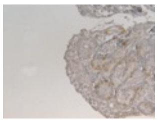
5C_20x3.tif

## Medium: D

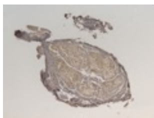
5D_20x.tif

## Medium: E

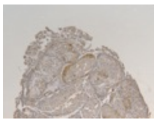
5E_20x.tif

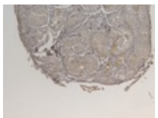
5E_20x1.tif

Week: 6

## Medium: A

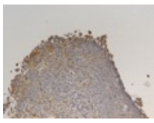
6A_20x2.tif

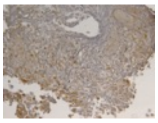
6A_20x3.tif

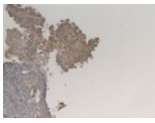
6A_20x5.tif

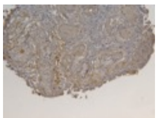
6A_20x6.tif

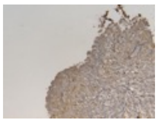
6A_20x7.tif

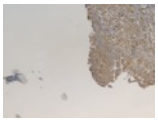
6A_20x10.tif

## Medium: B

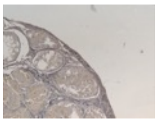
6B_20x1.tif

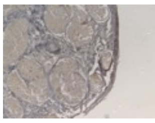
6B_20x2.tif

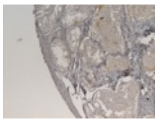
6B_20x3.tif

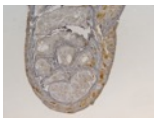
6B_20x6.tif

## Medium: C

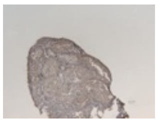
6C_20x.tif

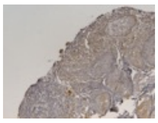
6C_20x2.tif

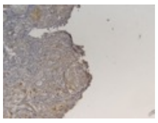
6C_20x4.tif

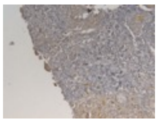
6C_20x5.tif

## Medium: D

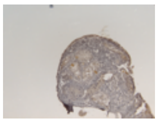
6D_20x.tif

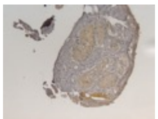
6D_20x1.tif

## Medium: E

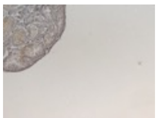
6E_20x2.tif

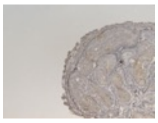
6E_20x3.tif

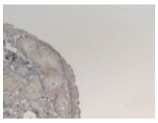
6E_20x4.tif

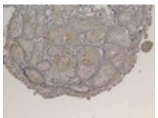
6E_20x5.tif

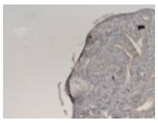
6E_20x6.tif

| D0 | Germ Cells | | | | | | | | | | |
|---|---|---|---|---|---|---|---|---|---|---|---|
| | Gonocytes and Spermatogonial cells (PGP9.5+ cells) | Spermatocytes | Spermatids | Total area | Seminiferous tubules area | Interstitial tissue area | % Seminiferous tubules | % Interstitial tissue | Normalized germ cell number | Normalized spermatogonial cell number |
| animal 13 | | | | | | | | | | |
| 13_20x.tif | 373 | 113 | 215 | 323 | 271 | 52 | 83,90092879 | 16,09907121 | | |
| 13_20x1.tif | 311 | 73 | 113 | 320 | 261 | 59 | 81,5625 | 18,4375 | | |
| 13_20x2.tif | 345 | 86 | 189 | 320 | 248 | 72 | 77,5 | 22,5 | | |
| | | | | | | Mean | 80,9878096 | 19,0121904 | 2,330769231 | 1,319230769 |
| animal 14 | | | | | | | | | | |
| 14_20x.tif | 210 | 80 | 91 | 343 | 240 | 103 | 69,97084548 | 30,02915452 | | |
| 14_20x1.tif | 177 | 56 | 84 | 335 | 262 | 73 | 78,20895522 | 21,79104478 | | |
| 14_20x2.tif | 206 | 75 | 103 | 336 | 240 | 96 | 71,42857143 | 28,57142857 | | |
| | | | | | | Mean | 73,20279071 | 26,79720929 | 1,458221024 | 0,799191375 |
| animal 22 | | | | | | | | | | |
| 22_20x.tif | 172 | 21 | 0 | 348 | 263 | 85 | 75,57471264 | 24,42528736 | | |
| 22_20x1.tif | 162 | 16 | 0 | 349 | 265 | 84 | 75,93123209 | 24,06876791 | | |
| 22_20x2.tif | 145 | 7 | 0 | 352 | 237 | 115 | 67,32954545 | 32,67045455 | | |
| | | | | | | Mean | 72,9451634 | 27,0548366 | 0,683660131 | 0,626143791 |
| animal 33 | | | | | | | | | | |
| 33_20x.tif | 154 | 29 | 19 | 352 | 282 | 70 | 80,11363636 | 19,88636364 | | |
| 33_20x1.tif | 178 | 41 | 20 | 353 | 263 | 90 | 74,50424929 | 25,49575071 | | |
| 33_20x2.tif | 189 | 50 | 27 | 352 | 278 | 74 | 78,97727273 | 21,02272727 | | |
| | | | | | | Mean | 77,86505279 | 22,13494721 | 0,859052248 | 0,633049818 |

| | | | | | | | | | | | |
|---|---|---|---|---|---|---|---|---|---|---|---|
| animal 34 | | | | | | | | | | | |
| 34_20x.tif | 161 | 20 | 57 | 352 | 278 | 74 | 78,97727273 | 21,02272727 | | | |
| 34_20x1.tif | 130 | 7 | 47 | 349 | 264 | 85 | 75,64469914 | 24,35530086 | | | |
| 34_20x2.tif | 150 | 56 | 68 | 350 | 250 | 100 | 71,42857143 | 28,57142857 | | | |
| | | | | | | Mean | 75,3501811 | 24,6498189 | | 0,878787879 | 0,556818182 |
| | | | | | | | | | | | |
| animal 36 | | | | | | | | | | | |
| 36_20x.tif | 221 | 90 | 231 | 338 | 282 | 56 | 83,43195266 | 16,56804734 | | | |
| 36_20x1.tif | 210 | 107 | 205 | 341 | 243 | 98 | 71,26099707 | 28,73900293 | | | |
| 36_20x2.tif | 85 | 49 | 103 | 329 | 238 | 91 | 72,34042553 | 27,65957447 | | | |
| | | | | | | Mean | 75,67779175 | 24,32220825 | | 1,705111402 | 0,676277851 |

| | Total area | Seminiferous tubules area | Interstitial tissue area | Necrotic tissue area | Number of germ cells | % Seminiferous tubules | % Interstitial tissue | % Necrotic tissue | Normalized germ cell number |
|---|---|---|---|---|---|---|---|---|---|
| **animal 13** | | | | | | | | | |
| 3A_20x1.tif | 106 | 98 | 0 | 8 | 23 | 92,45283019 | 0 | 7,54716981 | **Sum of the number of** |
| 3A_20x3.tif | 104 | 44 | 37 | 23 | 3 | 42,30769231 | 35,5769231 | 22,1153846 | **germ cells devided by** |
| 3A_20x5.tif | 206 | 90 | 93 | 23 | 7 | 43,68932039 | 45,1456311 | 11,1650485 | **the sum of area** |
| 3A_20x6.tif | 100 | 63 | 34 | 3 | 22 | 63 | 34 | 3 | **ocupied by** |
| 3A_20x10.tif | 143 | 2 | 23 | 118 | 4 | 1,398601399 | 16,0839161 | 82,5174825 | **seminiferous tubules** |
| 3A_20x11.tif | 78 | 32 | 16 | 30 | 6 | 41,02564103 | 20,5128205 | 38,4615385 | **of all images analysed** |
| | | | | | Mean | 47,31234755 | 25,2198818 | 27,4677707 | 0,197568389 |
| 3B_20x4.tif | 175 | 2 | 157 | 16 | 0 | 1,142857143 | 89,7142857 | 9,14285714 | |
| 3B_20x5.tif | 282 | 20 | 219 | 43 | 6 | 7,092198582 | 77,6595745 | 15,248227 | |
| 3B_20x6.tif | 194 | 50 | 82 | 62 | 9 | 25,77319588 | 42,2680412 | 31,9587629 | |
| 3B_20x7.tif | 52 | 14 | 34 | 4 | 0 | 26,92307692 | 65,3846154 | 7,69230769 | |
| | | | | | Mean | 15,23283213 | 68,7566292 | 16,0105387 | 0,174418605 |
| 3C_20x.tif | 161 | 37 | 68 | 56 | 2 | 22,98136646 | 42,2360248 | 34,7826087 | |
| 3C_20x2.tif | 164 | 46 | 80 | 38 | 8 | 28,04878049 | 48,7804878 | 23,1707317 | |
| 3C_20x3.tif | 221 | 73 | 102 | 46 | 10 | 33,03167421 | 46,1538462 | 20,8144796 | |
| | | | | | Mean | 28,02060705 | 45,7234529 | 26,25594 | 0,128205128 |
| 3D_20x1.tif | 124 | 0 | 78 | 46 | 0 | 0 | 62,9032258 | 37,0967742 | |
| 3D_20x2.tif | 178 | 47 | 100 | 31 | 11 | 26,40449438 | 56,1797753 | 17,4157303 | |

| | | | | | | | | | |
|---|---|---|---|---|---|---|---|---|---|
| | | | | | Mean | 13,20224719 | 59,5415005 | 27,2562523 | 0,234042553 |
| 3E_20x.tif | 203 | 60 | 143 | 0 | 18 | 29,55665025 | 70,4433498 | 0 | |
| 3E_20x2.tif | 207 | 116 | 91 | 0 | 39 | 56,03864734 | 43,9613527 | 0 | |
| 3E_20x5.tif | 166 | 53 | 54 | 59 | 4 | 31,92771084 | 32,5301205 | 35,5421687 | |
| 3E_20x7.tif | 154 | 41 | 51 | 62 | 5 | 26,62337662 | 33,1168831 | 40,2597403 | |
| 3E_20x9.tif | 142 | 50 | 74 | 18 | 14 | 35,21126761 | 52,1126761 | 12,6760563 | |
| | | | | | Mean | 35,87153053 | 46,4328764 | 17,6955931 | 0,25 |
| 4A_20x.tif | 149 | 59 | 66 | 24 | 6 | 39,59731544 | 44,295302 | 16,1073826 | |
| 4A_20x1.tif | 240 | 81 | 95 | 64 | 7 | 33,75 | 39,5833333 | 26,6666667 | |
| 4A_20x2.tif | 225 | 73 | 96 | 56 | 10 | 32,44444444 | 42,6666667 | 24,8888889 | |
| 4A_20x4.tif | 76 | 24 | 36 | 16 | 0 | 31,57894737 | 47,3684211 | 21,0526316 | |
| 4A_20x5.tif | 150 | 75 | 45 | 30 | 29 | 50 | 30 | 20 | |
| 4A_20x6.tif | 75 | 19 | 30 | 26 | 9 | 25,33333333 | 40 | 34,6666667 | |
| 4A_20x11.tif | 84 | 22 | 39 | 23 | 4 | 26,19047619 | 46,4285714 | 27,3809524 | |
| | | | | | Mean | 34,12778811 | 41,4774706 | 24,3947412 | 0,184135977 |
| 4B_20x.tif | 167 | 101 | 66 | 0 | 2 | 60,47904192 | 39,5209581 | 0 | |
| 4B_20x1.tif | 96 | 53 | 43 | 0 | 0 | 55,20833333 | 44,7916667 | 0 | |
| 4B_20x2.tif | 102 | 60 | 42 | 0 | 1 | 58,82352941 | 41,1764706 | 0 | |
| 4B_20x6.tif | 25 | 4 | 21 | 0 | 1 | 16 | 84 | 0 | |
| 4B_20x8.tif | 87 | 10 | 44 | 33 | 0 | 11,49425287 | 50,5747126 | 37,9310345 | |
| | | | | | Mean | 40,40103151 | 52,0127616 | 7,5862069 | 0,01754386 |

| | | | | | | | | | |
|---|---|---|---|---|---|---|---|---|---|
| 4C_20x.tif | 229 | 102 | 125 | 2 | 20 | 44,54148472 | 54,5851528 | 0,87336245 | |
| 4C_20x2.tif | 166 | 80 | 79 | 7 | 17 | 48,19277108 | 47,5903614 | 4,21686747 | |
| 4C_20x4.tif | 77 | 23 | 54 | 0 | 3 | 29,87012987 | 70,1298701 | 0 | |
| | | | | | Mean | 40,86812856 | 57,4351281 | 1,69674331 | 0,195121951 |
| 4D_20x1.tif | 150 | 57 | 83 | 10 | 10 | 38 | 55,3333333 | 6,66666667 | |
| 4D_20x3.tif | 128 | 28 | 88 | 12 | 12 | 21,875 | 68,75 | 9,375 | |
| 4D_20x4.tif | 159 | 37 | 122 | 0 | 27 | 23,27044025 | 76,7295597 | 0 | |
| | | | | | Mean | 27,71514675 | 66,937631 | 5,34722222 | 0,401639344 |
| 4E_20x.tif | 238 | 45 | 172 | 21 | 21 | 18,90756303 | 72,2689076 | 8,82352941 | |
| 4E_20x1.tif | 176 | 0 | 81 | 95 | 0 | 0 | 46,0227273 | 53,9772727 | |
| 4E_20x2.tif | 148 | 62 | 50 | 36 | 24 | 41,89189189 | 33,7837838 | 24,3243243 | |
| 4E_20x3.tif | 54 | 8 | 46 | 0 | 2 | 14,81481481 | 85,1851852 | 0 | |
| 4E_20x6.tif | 216 | 22 | 148 | 46 | 4 | 10,18518519 | 68,5185185 | 21,2962963 | |
| | | | | | Mean | 17,15989098 | 61,1558245 | 21,6842846 | 0,372262774 |
| 5A_20x3.tif | 149 | 80 | 61 | 8 | 10 | 53,69127517 | 40,9395973 | 5,36912752 | |
| 5A_20x4.tif | 245 | 71 | 117 | 57 | 26 | 28,97959184 | 47,755102 | 23,2653061 | |
| 5A_20x5.tif | 99 | 27 | 28 | 44 | 2 | 27,27272727 | 28,2828283 | 44,4444444 | |
| 5A_20x6.tif | 152 | 67 | 77 | 8 | 22 | 44,07894737 | 50,6578947 | 5,26315789 | |
| 5A_20x8.tif | 167 | 49 | 62 | 56 | 5 | 29,34131737 | 37,1257485 | 33,5329341 | |
| 5A_20x9.tif | 99 | 53 | 46 | 0 | 3 | 53,53535354 | 46,4646465 | 0 | |
| 5A_20x12.tif | 172 | 17 | 142 | 13 | 6 | 9,88372093 | 82,5581395 | 7,55813953 | |
| 5A_20x14.tif | 48 | 17 | 31 | 0 | 4 | 35,41666667 | 64,5833333 | 0 | |

| | | | | | | | | | |
|---|---|---|---|---|---|---|---|---|---|
| | | | | | Mean | 35,27495002 | 49,7959113 | 14,9291387 | 0,204724409 |
| 5B_20x.tif | 218 | 51 | 106 | 61 | 2 | 23,39449541 | 48,6238532 | 27,9816514 | |
| 5B_20x1.tif | 65 | 24 | 41 | 0 | 0 | 36,92307692 | 63,0769231 | 0 | |
| 5B_20x4.tif | 64 | 0 | 61 | 3 | 0 | 0 | 95,3125 | 4,6875 | |
| 5B_20x7.tif | 78 | 0 | 78 | 0 | 0 | 0 | 100 | 0 | |
| 5B_20x8.tif | 288 | 76 | 178 | 34 | 6 | 26,38888889 | 61,8055556 | 11,8055556 | |
| 5B_20x10.tif | 95 | 64 | 19 | 12 | 2 | 67,36842105 | 20 | 12,6315789 | |
| 5B_20x11.tif | 129 | 58 | 20 | 51 | 0 | 44,96124031 | 15,503876 | 39,5348837 | |
| 5B_20x12.tif | 133 | 82 | 24 | 27 | 4 | 61,65413534 | 18,0451128 | 20,3007519 | |
| | | | | | Mean | 32,58628224 | 52,7959776 | 14,6177402 | 0,03943662 |
| 5C_20x1.tif | 96 | 28 | 52 | 16 | 3 | 29,16666667 | 54,1666667 | 16,6666667 | |
| 5C_20x5.tif | 61 | 31 | 30 | 0 | 6 | 50,81967213 | 49,1803279 | 0 | |
| 5C_20x7.tif | 161 | 84 | 77 | 0 | 29 | 52,17391304 | 47,826087 | 0 | |
| 5C_20x9.tif | 275 | 158 | 111 | 6 | 26 | 57,45454545 | 40,3636364 | 2,18181818 | |
| 5C_20x12.tif | 157 | 90 | 67 | 0 | 17 | 57,32484076 | 42,6751592 | 0 | |
| 5C_20x13.tif | 188 | 101 | 72 | 15 | 7 | 53,72340426 | 38,2978723 | 7,9787234 | |
| 5C_20x15.tif | 186 | 134 | 52 | 0 | 6 | 72,04301075 | 27,9569892 | 0 | |
| 5C_20x16.tif | 123 | 76 | 47 | 0 | 9 | 61,78861789 | 38,2113821 | 0 | |
| | | | | | Mean | 54,31183387 | 42,3347651 | 3,35340103 | 0,146723647 |
| 5D_20x3.tif | 47 | 17 | 20 | 10 | 0 | 36,17021277 | 42,5531915 | 21,2765957 | |
| 5D_20x4.tif | 193 | 96 | 86 | 11 | 2 | 49,74093264 | 44,5595855 | 5,69948187 | |
| 5D_20x6.tif | 122 | 62 | 43 | 17 | 1 | 50,81967213 | 35,2459016 | 13,9344262 | |

| | | | | | | | | | | |
|---|---|---|---|---|---|---|---|---|---|---|
| | | | | | | Mean | 45,57693918 | 40,7862262 | 13,6368346 | 0,017142857 |
| 5E_20x.tif | 190 | 99 | 84 | 7 | 15 | | 52,10526316 | 44,2105263 | 3,68421053 | |
| 5E_20x2.tif | 78 | 29 | 49 | 0 | 3 | | 37,17948718 | 62,8205128 | 0 | |
| | | | | | | Mean | 44,64237517 | 53,5155196 | 1,84210526 | 0,140625 |
| 6A_20x.tif | 241 | 60 | 181 | 0 | 0 | | 24,89626556 | 75,1037344 | 0 | |
| 6A_20x1.tif | 77 | 39 | 38 | 0 | 0 | | 50,64935065 | 49,3506494 | 0 | |
| 6A_20x7.tif | 38 | 4 | 34 | 0 | 0 | | 10,52631579 | 89,4736842 | 0 | |
| 6A_20x8.tif | 201 | 103 | 98 | 0 | 6 | | 51,24378109 | 48,7562189 | 0 | |
| 6A_20x10.tif | 198 | 66 | 132 | 0 | 3 | | 33,33333333 | 66,6666667 | 0 | |
| 6A_20x11.tif | 95 | 31 | 64 | 0 | 2 | | 32,63157895 | 67,3684211 | 0 | |
| 6A_20x13.tif | 188 | 91 | 97 | 0 | 2 | | 48,40425532 | 51,5957447 | 0 | |
| | | | | | | Mean | 35,95498296 | 64,045017 | 0 | 0,032994924 |
| 6B_20x1.tif | 231 | 88 | 70 | 73 | 3 | | 38,0952381 | 30,3030303 | 31,6017316 | |
| 6B_20x2.tif | 139 | 54 | 41 | 44 | 3 | | 38,84892086 | 29,4964029 | 31,6546763 | |
| 6B_20x5.tif | 95 | 0 | 95 | 0 | 0 | | 0 | 100 | 0 | |
| 6B_20x6.tif | 126 | 12 | 114 | 0 | 2 | | 9,523809524 | 90,4761905 | 0 | |
| 6B_20x7.tif | 258 | 16 | 234 | 8 | 0 | | 6,201550388 | 90,6976744 | 3,10077519 | |
| 6B_20x9.tif | 221 | 26 | 195 | 0 | 0 | | 11,76470588 | 88,2352941 | 0 | |
| 6B_20x11.tif | 243 | 50 | 193 | 0 | 0 | | 20,57613169 | 79,4238683 | 0 | |
| | | | | | | Mean | 17,85862235 | 72,6617801 | 9,47959758 | 0,032520325 |

| | | | | | | | | | |
|---|---|---|---|---|---|---|---|---|---|
| 6C_20x1.tif | 95 | 67 | 28 | 0 | 1 | 70,52631579 | 29,4736842 | 0 | |
| 6C_20x3.tif | 229 | 8 | 221 | 0 | 0 | 3,493449782 | 96,5065502 | 0 | |
| 6C_20x4.tif | 76 | 5 | 71 | 0 | 2 | 6,578947368 | 93,4210526 | 0 | |
| 6C_20x5.tif | 207 | 70 | 133 | 4 | 5 | 33,81642512 | 64,2512077 | 1,93236715 | |
| | | | | | Mean | 28,60378452 | 70,9131237 | 0,48309179 | 0,053333333 |
| 6D_20x.tif | 134 | 58 | 53 | 23 | 14 | 43,28358209 | 39,5522388 | 17,1641791 | |
| 6D_20x1.tif | 124 | 47 | 69 | 8 | 2 | 37,90322581 | 55,6451613 | 6,4516129 | |
| 6D_20x2.tif | 124 | 80 | 34 | 10 | 7 | 64,51612903 | 27,4193548 | 8,06451613 | |
| | | | | | Mean | 48,56764564 | 40,8722516 | 10,5601027 | 0,124324324 |
| 6E_20x2.tif | 127 | 105 | 18 | 4 | 0 | 82,67716535 | 14,1732283 | 3,1496063 | |
| 6E_20x3.tif | 125 | 97 | 24 | 4 | 2 | 77,6 | 19,2 | 3,2 | |
| 6E_20x4.tif | 179 | 94 | 67 | 18 | 15 | 52,51396648 | 37,4301676 | 10,0558659 | |
| | | | | | Mean | 70,93037728 | 23,601132 | 5,46849074 | 0,057432432 |
| **animal 14** | | | | | | | | | |
| 2A_20x.tif | 218 | 159 | 56 | 3 | 108 | 72,93577982 | 25,6880734 | 1,37614679 | |
| 2A_20x1.tif | 183 | 121 | 44 | 18 | 72 | 66,12021858 | 24,0437158 | 9,83606557 | |
| 2A_20x3.tif | 236 | 65 | 127 | 44 | 27 | 27,54237288 | 53,8135593 | 18,6440678 | |
| 2A_20x6.tif | 285 | 176 | 78 | 31 | 127 | 61,75438596 | 27,3684211 | 10,877193 | |
| 2A_20x8.tif | 172 | 89 | 46 | 37 | 55 | 51,74418605 | 26,744186 | 21,5116279 | |
| 2A_20x9.tif | 96 | 67 | 25 | 4 | 60 | 69,79166667 | 26,0416667 | 4,16666667 | |
| 2A_20x11.tif | 66 | 24 | 26 | 16 | 15 | 36,36363636 | 39,3939394 | 24,2424242 | |
| 2A_20x14.tif | 32 | 28 | 4 | 0 | 31 | 87,5 | 12,5 | 0 | |
| | | | | | Mean | 59,21903079 | 29,4491952 | 11,331774 | 0,679012346 |

| | | | | | | | | | | |
|---|---|---|---|---|---|---|---|---|---|---|
| 2B_20x.tif | 193 | 63 | 107 | 23 | 25 | | 32,64248705 | 55,4404145 | 11,9170984 | |
| 2B_20x1.tif | 164 | 61 | 98 | 5 | 23 | | 37,19512195 | 59,7560976 | 3,04878049 | |
| 2B_20x3.tif | 191 | 95 | 58 | 38 | 22 | | 49,7382199 | 30,3664921 | 19,895288 | |
| | | | | | | Mean | 39,85860963 | 48,5210014 | 11,620389 | 0,319634703 |
| 2D_20x.tif | 198 | 18 | 110 | 70 | 12 | | 9,090909091 | 55,5555556 | 35,3535354 | |
| 2D_20x1.tif | 112 | 49 | 38 | 25 | 42 | | 43,75 | 33,9285714 | 22,3214286 | |
| 2D_20x3.tif | 258 | 47 | 151 | 60 | 23 | | 18,21705426 | 58,5271318 | 23,255814 | |
| 2D_20x6.tif | 202 | 29 | 124 | 49 | 27 | | 14,35643564 | 61,3861386 | 24,2574257 | |
| | | | | | | Mean | 23,68598778 | 49,3370863 | 26,976926 | 0,727272727 |
| 2E_20x3.tif | 60 | 17 | 29 | 14 | 6 | | 28,33333333 | 48,3333333 | 23,3333333 | |
| 2E_20x4.tif | 185 | 47 | 35 | 103 | 33 | | 25,40540541 | 18,9189189 | 55,6756757 | |
| 2E_20x5.tif | 252 | 46 | 62 | 144 | 31 | | 18,25396825 | 24,6031746 | 57,1428571 | |
| 2E_20x6.tif | 235 | 37 | 59 | 139 | 34 | | 15,74468085 | 25,106383 | 59,1489362 | |
| 2E_20x8.tif | 110 | 37 | 52 | 21 | 30 | | 33,63636364 | 47,2727273 | 19,0909091 | |
| | | | | | | Mean | 24,2747503 | 32,8469074 | 42,8783423 | 0,72826087 |
| 3A_20x.tif | 196 | 31 | 80 | 85 | 19 | | 15,81632653 | 15,8163265 | 43,3673469 | |
| 3A_20x2.tif | 94 | 42 | 38 | 14 | 15 | | 44,68085106 | 44,6808511 | 14,893617 | |
| 3A_20x3.tif | 93 | 42 | 29 | 22 | 21 | | 45,16129032 | 45,1612903 | 23,655914 | |
| 3A_20x4.tif | 205 | 31 | 55 | 119 | 4 | | 15,12195122 | 15,1219512 | 58,0487805 | |
| 3A_20x7.tif | 183 | 35 | 98 | 50 | 19 | | 19,12568306 | 19,1256831 | 27,3224044 | |

| | | | | | | | | | |
|---|---|---|---|---|---|---|---|---|---|
| 3A_20x10.tif | 69 | 8 | 54 | 7 | 1 | 11,5942029 | 11,5942029 | 10,1449275 | |
| 3A_20x11.tif | 237 | 99 | 93 | 45 | 60 | 41,7721519 | 41,7721519 | 18,9873418 | |
| 3A_20x13.tif | 209 | 56 | 114 | 39 | 46 | 26,79425837 | 26,7942584 | 18,6602871 | |
| | | | | | Mean | 27,50833942 | 27,5083394 | 26,8850774 | 0,537790698 |
| | | | | | | | | | |
| 3B_20x.tif | 180 | 14 | 139 | 27 | 6 | 7,777777778 | 77,2222222 | 15 | |
| 3B_20x2.tif | 212 | 21 | 137 | 54 | 7 | 9,905660377 | 64,6226415 | 25,4716981 | |
| 3B_20x4.tif | 54 | 4 | 48 | 2 | 0 | 7,407407407 | 88,8888889 | 3,7037037 | |
| 3B_20x7.tif | 252 | 41 | 50 | 161 | 1 | 16,26984127 | 19,8412698 | 63,8888889 | |
| 3B_20x8.tif | 193 | 89 | 69 | 35 | 3 | 46,11398964 | 35,7512953 | 18,134715 | |
| 3B_20x10.tif | 39 | 14 | 25 | 0 | 4 | 35,8974359 | 64,1025641 | 0 | |
| | | | | | Mean | 20,56201873 | 58,4048137 | 21,0331676 | 0,114754098 |
| | | | | | | | | | |
| 3C_20x.tif | 139 | 36 | 52 | 51 | 14 | 25,89928058 | 37,4100719 | 36,6906475 | |
| 3C_20x2.tif | 251 | 80 | 60 | 111 | 68 | 31,87250996 | 23,9043825 | 44,2231076 | |
| 3C_20x6.tif | 135 | 64 | 37 | 34 | 21 | 47,40740741 | 27,4074074 | 25,1851852 | |
| 3C_20x7.tif | 62 | 34 | 23 | 5 | 11 | 54,83870968 | 37,0967742 | 8,06451613 | |
| 3C_20x8.tif | 255 | 82 | 41 | 132 | 30 | 32,15686275 | 16,0784314 | 51,7647059 | |
| 3C_20x10.tif | 269 | 145 | 47 | 77 | 45 | 53,90334572 | 17,472119 | 28,6245353 | |
| 3C_20x13.tif | 40 | 20 | 20 | 0 | 8 | 50 | 50 | 0 | |
| | | | | | Mean | 42,29687373 | 29,9098838 | 27,7932425 | 0,427331887 |
| | | | | | | | | | |
| 3D_20x.tif | 237 | 60 | 126 | 51 | 16 | 25,3164557 | 53,164557 | 21,5189873 | |
| 3D_20x1.tif | 72 | 11 | 61 | 0 | 7 | 15,27777778 | 84,7222222 | 0 | |
| 3D_20x4.tif | 115 | 0 | 106 | 9 | 0 | 0 | 92,173913 | 7,82608696 | |
| 3D_20x6.tif | 144 | 19 | 110 | 15 | 7 | 13,19444444 | 76,3888889 | 10,4166667 | |

| | | | | | | | | | | |
|---|---|---|---|---|---|---|---|---|---|---|
| | | | | | | Mean | 13,44716948 | 76,6123953 | 9,94043524 | 0,333333333 |
| 3E_20x.tif | 209 | 52 | 102 | 55 | 28 | | 24,88038278 | 48,8038278 | 26,3157895 | |
| 3E_20x1.tif | 90 | 30 | 31 | 29 | 13 | | 33,33333333 | 34,4444444 | 32,2222222 | |
| 3E_20x3.tif | 151 | 39 | 73 | 39 | 32 | | 25,82781457 | 48,3443709 | 25,8278146 | |
| 3E_20x4.tif | 88 | 11 | 53 | 24 | 12 | | 12,5 | 60,2272727 | 27,2727273 | |
| 3E_20x7.tif | 216 | 30 | 117 | 69 | 15 | | 13,88888889 | 54,1666667 | 31,9444444 | |
| | | | | | | Mean | 22,08608391 | 49,1973165 | 28,7165996 | 0,617283951 |
| 4A_20x1.tif | 144 | 50 | 64 | 30 | 20 | | 34,72222222 | 44,4444444 | 20,8333333 | |
| 4A_20x3.tif | 77 | 21 | 23 | 33 | 7 | | 27,27272727 | 29,8701299 | 42,8571429 | |
| 4A_20x4.tif | 176 | 34 | 101 | 41 | 4 | | 19,31818182 | 57,3863636 | 23,2954545 | |
| 4A_20x5.tif | 125 | 42 | 57 | 26 | 16 | | 33,6 | 45,6 | 20,8 | |
| 4A_20x14.tif | 291 | 30 | 62 | 199 | 10 | | 10,30927835 | 21,3058419 | 68,3848797 | |
| 4A_20x15.tif | 170 | 78 | 65 | 27 | 41 | | 45,88235294 | 38,2352941 | 15,8823529 | |
| 4A_20x16.tif | 97 | 43 | 54 | 0 | 20 | | 44,32989691 | 55,6701031 | 0 | |
| 4A_20x18.tif | 100 | 54 | 39 | 7 | 21 | | 54 | 39 | 7 | |
| 4A_20x20.tif | 243 | 82 | 67 | 94 | 21 | | 33,74485597 | 27,5720165 | 38,6831276 | |
| 4A_20x21.tif | 42 | 15 | 25 | 2 | 1 | | 35,71428571 | 59,5238095 | 4,76190476 | |
| 4A_20x23.tif | 107 | 47 | 60 | 0 | 16 | | 43,92523364 | 56,0747664 | 0 | |
| 4A_20x25.tif | 44 | 6 | 38 | 0 | 0 | | 13,63636364 | 86,3636364 | 0 | |
| 4A_20x27.tif | 134 | 23 | 79 | 32 | 14 | | 17,1641791 | 58,9552239 | 23,880597 | |
| 4A_20x29.tif | 215 | 27 | 77 | 111 | 12 | | 12,55813953 | 35,8139535 | 51,627907 | |
| 4A_20x30.tif | 49 | 7 | 42 | 0 | 2 | | 14,28571429 | 85,7142857 | 0 | |
| 4A_20x31.tif | 126 | 26 | 90 | 10 | 12 | | 20,63492063 | 71,4285714 | 7,93650794 | |
| | | | | | | Mean | 28,818647 | 50,8099025 | 20,3714505 | 0,370940171 |

| | | | | | | | | | | |
|---|---|---|---|---|---|---|---|---|---|---|
| 4B_20x2.tif | 259 | 32 | 135 | 92 | 8 | | 12,35521236 | 52,1235521 | 35,5212355 | |
| 4B_20x4.tif | 130 | 9 | 96 | 25 | 1 | | 6,923076923 | 73,8461538 | 19,2307692 | |
| 4B_20x5.tif | 242 | 27 | 158 | 57 | 16 | | 11,15702479 | 65,2892562 | 23,553719 | |
| | | | | | | Mean | 10,14510469 | 63,7529874 | 26,1019079 | 0,367647059 |
| 4C_20x3.tif | 307 | 0 | 101 | 206 | 0 | | 0 | 32,8990228 | 67,1009772 | |
| 4C_20x5.tif | 181 | 0 | 87 | 94 | 0 | | 0 | 48,0662983 | 51,9337017 | |
| 4C_20x7.tif | 245 | 29 | 97 | 119 | 11 | | 11,83673469 | 39,5918367 | 48,5714286 | |
| 4C_20x8.tif | 131 | 24 | 73 | 34 | 10 | | 18,32061069 | 55,7251908 | 25,9541985 | |
| 4C_20x9.tif | 132 | 30 | 78 | 24 | 17 | | 22,72727273 | 59,0909091 | 18,1818182 | |
| 4C_20x10.tif | 75 | 26 | 46 | 3 | 15 | | 34,66666667 | 61,3333333 | 4 | |
| | | | | | | Mean | 14,5918808 | 49,4510985 | 35,9570207 | 0,486238532 |
| 4D_20x2.tif | 254 | 67 | 102 | 85 | 39 | | 26,37795276 | 40,1574803 | 33,4645669 | |
| 4D_20x3.tif | 125 | 31 | 53 | 41 | 23 | | 24,8 | 42,4 | 32,8 | |
| 4D_20x5.tif | 158 | 40 | 83 | 35 | 18 | | 25,3164557 | 52,5316456 | 22,1518987 | |
| 4D_20x6.tif | 206 | 44 | 123 | 39 | 44 | | 21,3592233 | 59,7087379 | 18,9320388 | |
| | | | | | | Mean | 24,46340794 | 48,6994659 | 26,8371261 | 0,681318681 |
| 4E_20x2.tif | 281 | 8 | 156 | 117 | 6 | | 2,846975089 | 55,5160142 | 41,6370107 | |
| 4E_20x3.tif | 290 | 17 | 150 | 123 | 9 | | 5,862068966 | 51,7241379 | 42,4137931 | |
| 4E_20x4.tif | 64 | 17 | 38 | 9 | 0 | | 26,5625 | 59,375 | 14,0625 | |
| 4E_20x5.tif | 78 | 7 | 55 | 16 | 4 | | 8,974358974 | 70,5128205 | 20,5128205 | |
| | | | | | | Mean | 11,06147576 | 59,2819932 | 29,6565311 | 0,387755102 |

| | | | | | | | | | |
|---|---|---|---|---|---|---|---|---|---|
| 5A_20x.tif | 195 | 16 | 158 | 21 | 0 | 8,205128205 | 81,025641 | 10,7692308 | |
| 5A_20x1.tif | 141 | 6 | 109 | 26 | 1 | 4,255319149 | 77,3049645 | 18,4397163 | |
| 5A_20x4.tif | 62 | 23 | 39 | 0 | 6 | 37,09677419 | 62,9032258 | 0 | |
| 5A_20x5.tif | 46 | 8 | 38 | 0 | 2 | 17,39130435 | 82,6086957 | 0 | |
| 5A_20x6.tif | 230 | 130 | 79 | 21 | 46 | 56,52173913 | 34,3478261 | 9,13043478 | |
| 5A_20x9.tif | 105 | 37 | 68 | 0 | 26 | 35,23809524 | 64,7619048 | 0 | |
| 5A_20x11.tif | 84 | 34 | 42 | 8 | 1 | 40,47619048 | 50 | 9,52380952 | |
| 5A_20x12.tif | 137 | 64 | 67 | 6 | 18 | 46,71532847 | 48,9051095 | 4,37956204 | |
| 5A_20x15.tif | 198 | 105 | 83 | 10 | 39 | 53,03030303 | 41,9191919 | 5,05050505 | |
| 5A_20x16.tif | 129 | 50 | 79 | 0 | 11 | 38,75968992 | 61,2403101 | 0 | |
| | | | | | Mean | 33,76898722 | 60,5016869 | 5,72932585 | 0,317124736 |

| | | | | | | | | | |
|---|---|---|---|---|---|---|---|---|---|
| 5B_20x.tif | 178 | 124 | 54 | 0 | 6 | 69,66292135 | 30,3370787 | 0 | |
| 5B_20x2.tif | 142 | 61 | 60 | 21 | 0 | 42,95774648 | 42,2535211 | 14,7887324 | |
| 5B_20x6.tif | 101 | 0 | 84 | 17 | 0 | 0 | 83,1683168 | 16,8316832 | |
| 5B_20x7.tif | 225 | 1 | 139 | 85 | 2 | 0,444444444 | 61,7777778 | 37,7777778 | |
| 5B_20x8.tif | 25 | 0 | 22 | 3 | 0 | 0 | 88 | 12 | |
| 5B_20x10.tif | 141 | 90 | 51 | 0 | 2 | 63,82978723 | 36,1702128 | 0 | |
| | | | | | Mean | 29,48248325 | 56,9511512 | 13,5663656 | 0,036231884 |

| | | | | | | | | | |
|---|---|---|---|---|---|---|---|---|---|
| 5C_20x.tif | 205 | 52 | 153 | 0 | 19 | 25,36585366 | 74,6341463 | 0 | |
| 5C_20x2.tif | 196 | 51 | 145 | 0 | 52 | 26,02040816 | 73,9795918 | 0 | |
| 5C_20x3.tif | 253 | 112 | 131 | 10 | 50 | 44,2687747 | 51,7786561 | 3,95256917 | |
| 5C_20x6.tif | 173 | 102 | 68 | 3 | 40 | 58,95953757 | 39,3063584 | 1,73410405 | |
| | | | | | Mean | 38,65364352 | 59,9246882 | 1,4216683 | 0,507886436 |

| | | | | | | | | | |
|---|---|---|---|---|---|---|---|---|---|
| 5D_20x.tif | 124 | 13 | 90 | 21 | 3 | 10,48387097 | 72,5806452 | 16,9354839 | |
| 5D_20x1.tif | 264 | 28 | 149 | 87 | 14 | 10,60606061 | 56,4393939 | 32,9545455 | |
| 5D_20x2.tif | 106 | 22 | 67 | 17 | 6 | 20,75471698 | 63,2075472 | 16,0377358 | |
| 5D_20x4.tif | 217 | 38 | 129 | 50 | 16 | 17,51152074 | 59,4470046 | 23,0414747 | |
| 5D_20x8.tif | 78 | 32 | 38 | 8 | 17 | 41,02564103 | 48,7179487 | 10,2564103 | |
| 5D_20x9.tif | 174 | 72 | 88 | 14 | 29 | 41,37931034 | 50,5747126 | 8,04597701 | |
| | | | | | Mean | 23,62685344 | 58,494542 | 17,8786045 | 0,414634146 |
| 5E_20x1.tif | 173 | 5 | 133 | 35 | 4 | 2,89017341 | 76,8786127 | 20,2312139 | |
| 5E_20x2.tif | 212 | 6 | 144 | 62 | 3 | 2,830188679 | 67,9245283 | 29,245283 | |
| 5E_20x3.tif | 151 | 19 | 96 | 36 | 13 | 12,58278146 | 63,5761589 | 23,8410596 | |
| 5E_20x4.tif | 122 | 0 | 111 | 11 | 0 | 0 | 90,9836066 | 9,01639344 | |
| | | | | | Mean | 4,575785887 | 74,8407266 | 20,5834875 | 0,666666667 |
| 6A_20x.tif | 255 | 79 | 120 | 56 | 3 | 30,98039216 | 47,0588235 | 21,9607843 | |
| 6A_20x1.tif | 91 | 37 | 54 | 0 | 0 | 40,65934066 | 59,3406593 | 0 | |
| 6A_20x2.tif | 32 | 4 | 28 | 0 | 1 | 12,5 | 87,5 | 0 | |
| 6A_20x3.tif | 265 | 41 | 49 | 175 | 2 | 15,47169811 | 18,490566 | 66,0377358 | |
| 6A_20x4.tif | 243 | 29 | 124 | 90 | 0 | 11,93415638 | 51,0288066 | 37,037037 | |
| 6A_20x8.tif | 221 | 28 | 140 | 53 | 1 | 12,66968326 | 63,3484163 | 23,9819005 | |
| 6A_20x9.tif | 179 | 28 | 131 | 20 | 3 | 15,6424581 | 73,1843575 | 11,1731844 | |
| 6A_20x11.tif | 216 | 81 | 94 | 41 | 12 | 37,5 | 43,5185185 | 18,9814815 | |
| 6A_20x13.tif | 173 | 95 | 54 | 24 | 16 | 54,9132948 | 31,2138728 | 13,8728324 | |
| 6A_20x17.tif | 142 | 43 | 81 | 18 | 7 | 30,28169014 | 57,0422535 | 12,6760563 | |
| 6A_20x21.tif | 106 | 64 | 34 | 8 | 7 | 60,37735849 | 32,0754717 | 7,54716981 | |
| 6A_20x22.tif | 134 | 56 | 51 | 27 | 7 | 41,79104478 | 38,0597015 | 20,1492537 | |

| | | | | | | | | | |
|---|---|---|---|---|---|---|---|---|---|
| | | | | | Mean | 30,39342641 | 50,1551206 | 19,451453 | 0,100854701 |
| 6B_20x.tif | 160 | 31 | 64 | 65 | 2 | 19,375 | 40 | 40,625 | |
| 6B_20x1.tif | 46 | 15 | 26 | 5 | 1 | 32,60869565 | 56,5217391 | 10,8695652 | |
| 6B_20x3.tif | 87 | 40 | 27 | 20 | 4 | 45,97701149 | 31,0344828 | 22,9885057 | |
| 6B_20x7.tif | 60 | 0 | 60 | 0 | 0 | 0 | 100 | 0 | |
| | | | | | Mean | 24,49017679 | 56,8890555 | 18,6207677 | 0,081395349 |
| 6C_20x1.tif | 219 | 104 | 99 | 16 | 21 | 47,48858447 | 45,2054795 | 7,30593607 | |
| 6C_20x2.tif | 206 | 14 | 178 | 14 | 3 | 6,796116505 | 86,407767 | 6,7961165 | |
| 6C_20x4.tif | 221 | 40 | 175 | 6 | 6 | 18,09954751 | 79,1855204 | 2,71493213 | |
| 6C_20x7.tif | 210 | 7 | 153 | 50 | 1 | 3,333333333 | 72,8571429 | 23,8095238 | |
| 6C_20x9.tif | 200 | 14 | 120 | 66 | 8 | 7 | 60 | 33 | |
| 6C_20x11.tif | 75 | 4 | 62 | 9 | 2 | 5,333333333 | 82,6666667 | 12 | |
| | | | | | Mean | 14,67515253 | 71,0537627 | 14,2710848 | 0,224043716 |
| 6D_20x.tif | 201 | 65 | 118 | 18 | 49 | 32,33830846 | 58,7064677 | 8,95522388 | |
| 6D_20x6.tif | 285 | 56 | 156 | 73 | 26 | 19,64912281 | 54,7368421 | 25,6140351 | |
| 6D_20x7.tif | 40 | 18 | 20 | 2 | 10 | 45 | 50 | 5 | |
| 6D_20x8.tif | 225 | 64 | 126 | 35 | 23 | 28,44444444 | 56 | 15,5555556 | |
| 6D_20x9.tif | 79 | 27 | 43 | 9 | 15 | 34,17721519 | 54,4303797 | 11,3924051 | |
| 6D_20x11.tif | 101 | 50 | 43 | 8 | 22 | 49,5049505 | 42,5742574 | 7,92079208 | |
| | | | | | Mean | 34,85234023 | 52,7413245 | 12,4063353 | 0,517857143 |

| | | | | | | | | | |
|---|---|---|---|---|---|---|---|---|---|
| 6E_20x1.tif | 259 | 92 | 113 | 54 | 63 | 35,52123552 | 43,6293436 | 20,8494208 | |
| 6E_20x2.tif | 138 | 65 | 48 | 25 | 44 | 47,10144928 | 34,7826087 | 18,115942 | |
| 6E_20x3.tif | 38 | 13 | 18 | 7 | 3 | 34,21052632 | 47,3684211 | 18,4210526 | |
| 6E_20x4.tif | 185 | 66 | 87 | 32 | 42 | 35,67567568 | 47,027027 | 17,2972973 | |
| 6E_20x6.tif | 224 | 90 | 90 | 44 | 72 | 40,17857143 | 40,1785714 | 19,6428571 | |
| 6E_20x9.tif | 155 | 70 | 45 | 40 | 20 | 45,16129032 | 29,0322581 | 25,8064516 | |
| | | | | | Mean | 39,64145809 | 40,3363716 | 20,0221703 | 0,616161616 |
| **animal 22** | | | | | | | | | |
| 2A_20x1.tif | 87 | 42 | 38 | 7 | 47 | 48,27586207 | 43,6781609 | 8,04597701 | |
| 2A_20x3.tif | 201 | 81 | 103 | 17 | 133 | 40,29850746 | 51,2437811 | 8,45771144 | |
| 2A_20x4.tif | 111 | 27 | 82 | 2 | 27 | 24,32432432 | 73,8738739 | 1,8018018 | |
| 2A_20x7.tif | 247 | 134 | 87 | 26 | 194 | 54,25101215 | 35,2226721 | 10,5263158 | |
| 2A_20x9.tif | 42 | 30 | 12 | 0 | 41 | 71,42857143 | 28,5714286 | 0 | |
| 2A_20x10.tif | 293 | 188 | 88 | 17 | 184 | 64,16382253 | 30,0341297 | 5,80204778 | |
| 2A_20x12.tif | 145 | 93 | 52 | 0 | 135 | 64,13793103 | 35,862069 | 0 | |
| 2A_20x13.tif | 130 | 86 | 42 | 2 | 123 | 66,15384615 | 32,3076923 | 1,53846154 | |
| 2A_20x16.tif | 90 | 22 | 36 | 32 | 22 | 24,44444444 | 40 | 35,5555556 | |
| 2A_20x17.tif | 176 | 84 | 75 | 17 | 154 | 47,72727273 | 42,6136364 | 9,65909091 | |
| 2A_20x20.tif | 108 | 29 | 51 | 28 | 103 | 26,85185185 | 47,2222222 | 25,9259259 | |
| 2A_20x22.tif | 106 | 37 | 66 | 3 | 57 | 34,90566038 | 62,2641509 | 2,83018868 | |
| 2A_20x23.tif | 80 | 32 | 46 | 2 | 66 | 40 | 57,5 | 2,5 | |
| | | | | | Mean | 46,68946973 | 44,6456782 | 8,66485203 | 1,453107345 |
| 2B_20x.tif | 169 | 120 | 49 | 0 | 82 | 71,00591716 | 28,9940828 | 0 | |
| 2B_20x1.tif | 192 | 131 | 61 | 0 | 88 | 68,22916667 | 31,7708333 | 0 | |
| 2B_20x3.tif | 100 | 58 | 26 | 16 | 29 | 58 | 26 | 16 | |
| 2B_20x4.tif | 200 | 116 | 79 | 5 | 116 | 58 | 39,5 | 2,5 | |
| 2B_20x5.tif | 113 | 95 | 15 | 3 | 116 | 84,07079646 | 13,2743363 | 2,65486726 | |

| | | | | | | | | | |
|---|---|---|---|---|---|---|---|---|---|
| 2B_20x10.tif | 117 | 71 | 43 | 3 | 75 | 60,68376068 | 36,7521368 | 2,56410256 | |
| | | | | | Mean | 66,66494016 | 29,3818982 | 3,95316164 | 0,856175973 |
| | | | | | | | | | |
| 2C_20x2.tif | 283 | 112 | 120 | 51 | 225 | 39,57597173 | 42,4028269 | 18,0212014 | |
| 2C_20x3.tif | 235 | 104 | 107 | 24 | 206 | 44,25531915 | 45,5319149 | 10,212766 | |
| 2C_20x5.tif | 225 | 121 | 77 | 27 | 250 | 53,77777778 | 34,2222222 | 12 | |
| 2C_20x7.tif | 116 | 62 | 37 | 17 | 103 | 53,44827586 | 31,8965517 | 14,6551724 | |
| 2C_20x8.tif | 122 | 70 | 35 | 17 | 85 | 57,37704918 | 28,6885246 | 13,9344262 | |
| 2C_20x11.tif | 102 | 44 | 34 | 24 | 93 | 43,1372549 | 33,3333333 | 23,5294118 | |
| 2C_20x13.tif | 289 | 129 | 105 | 55 | 201 | 44,6366782 | 36,3321799 | 19,0311419 | |
| 2C_20x14.tif | 78 | 26 | 40 | 12 | 53 | 33,33333333 | 51,2820513 | 15,3846154 | |
| | | | | | Mean | 46,19270752 | 37,9612006 | 15,8460919 | 1,820359281 |
| | | | | | | | | | |
| 2D_20x1.tif | 136 | 78 | 46 | 12 | 112 | 57,35294118 | 33,8235294 | 8,82352941 | |
| 2D_20x4.tif | 164 | 77 | 65 | 22 | 154 | 46,95121951 | 39,6341463 | 13,4146341 | |
| 2D_20x5.tif | 125 | 64 | 48 | 13 | 77 | 51,2 | 38,4 | 10,4 | |
| 2D_20x6.tif | 96 | 42 | 39 | 15 | 81 | 43,75 | 40,625 | 15,625 | |
| 2D_20x7.tif | 126 | 32 | 61 | 33 | 21 | 25,3968254 | 48,4126984 | 26,1904762 | |
| 2D_20x9.tif | 175 | 69 | 66 | 40 | 119 | 39,42857143 | 37,7142857 | 22,8571429 | |
| | | | | | Mean | 44,01325959 | 39,7682766 | 16,2184638 | 1,55801105 |
| | | | | | | | | | |
| 2E_20x.tif | 161 | 39 | 48 | 74 | 76 | 24,22360248 | 29,8136646 | 45,9627329 | |
| 2E_20x2.tif | 121 | 50 | 49 | 22 | 110 | 41,32231405 | 40,4958678 | 18,1818182 | |
| 2E_20x4.tif | 123 | 42 | 29 | 52 | 94 | 34,14634146 | 23,5772358 | 42,2764228 | |
| 2E_20x6.tif | 100 | 46 | 22 | 32 | 98 | 46 | 22 | 32 | |
| 2E_20x8.tif | 130 | 29 | 74 | 27 | 78 | 22,30769231 | 56,9230769 | 20,7692308 | |

| | | | | | | | | | |
|---|---|---|---|---|---|---|---|---|---|
| 2E_20x9.tif | 246 | 51 | 137 | 58 | 124 | 20,73170732 | 55,6910569 | 23,5772358 | |
| | | | | | Mean | 31,45527627 | 38,0834837 | 30,4612401 | 2,256809339 |
| 3A_20x.tif | 234 | 87 | 66 | 81 | 149 | 37,17948718 | 28,2051282 | 34,6153846 | |
| 3A_20x1.tif | 176 | 69 | 72 | 35 | 134 | 39,20454545 | 40,9090909 | 19,8863636 | |
| 3A_20x2.tif | 215 | 90 | 88 | 37 | 100 | 41,86046512 | 40,9302326 | 17,2093023 | |
| 3A_20x5.tif | 219 | 97 | 103 | 19 | 192 | 44,29223744 | 47,0319635 | 8,67579909 | |
| 3A_20x6.tif | 208 | 84 | 102 | 22 | 130 | 40,38461538 | 49,0384615 | 10,5769231 | |
| 3A_20x10.tif | 227 | 134 | 53 | 40 | 268 | 59,030837 | 23,3480176 | 17,6211454 | |
| 3A_20x12.tif | 271 | 149 | 94 | 28 | 178 | 54,98154982 | 34,6863469 | 10,3321033 | |
| 3A_20x13.tif | 287 | 124 | 105 | 58 | 167 | 43,20557491 | 36,5853659 | 20,2090592 | |
| 3A_20x14.tif | 206 | 61 | 99 | 46 | 79 | 29,61165049 | 48,0582524 | 22,3300971 | |
| 3A_20x15.tif | 210 | 104 | 81 | 25 | 154 | 49,52380952 | 38,5714286 | 11,9047619 | |
| 3A_20x18.tif | 55 | 11 | 24 | 20 | 13 | 20 | 43,6363636 | 36,3636364 | |
| 3A_20x22.tif | 137 | 35 | 82 | 20 | 47 | 25,54744526 | 59,8540146 | 14,5985401 | |
| | | | | | Mean | 40,40185146 | 40,9045555 | 18,693593 | 1,541626794 |
| 3B_20x1.tif | 188 | 92 | 72 | 24 | 39 | 48,93617021 | 38,2978723 | 12,7659574 | |
| 3B_20x6.tif | 197 | 85 | 90 | 22 | 49 | 43,14720812 | 45,6852792 | 11,1675127 | |
| 3B_20x7.tif | 127 | 81 | 46 | 0 | 58 | 63,77952756 | 36,2204724 | 0 | |
| 3B_20x10.tif | 220 | 13 | 108 | 99 | 19 | 5,909090909 | 49,0909091 | 45 | |
| 3B_20x12.tif | 226 | 17 | 91 | 118 | 13 | 7,522123894 | 40,2654867 | 52,2123894 | |
| 3B_20x14.tif | 196 | 111 | 60 | 25 | 74 | 56,63265306 | 30,6122449 | 12,755102 | |
| 3B_20x15.tif | 142 | 76 | 50 | 16 | 39 | 53,52112676 | 35,2112676 | 11,2676056 | |
| 3B_20x16.tif | 307 | 121 | 131 | 55 | 133 | 39,41368078 | 42,6710098 | 17,9153094 | |
| 3B_20x17.tif | 181 | 113 | 48 | 20 | 54 | 62,43093923 | 26,519337 | 11,0497238 | |
| 3B_20x18.tif | 94 | 78 | 16 | 0 | 33 | 82,9787234 | 17,0212766 | 0 | |
| | | | | | Mean | 46,42712439 | 36,1595156 | 17,41336 | 0,649301144 |

| | | | | | | | | | |
|---|---|---|---|---|---|---|---|---|---|
| 3C_20x.tif | 241 | 71 | 162 | 8 | 91 | 29,46058091 | 67,219917 | 3,31950207 | |
| 3C_20x4.tif | 190 | 30 | 116 | 44 | 62 | 15,78947368 | 61,0526316 | 23,1578947 | |
| 3C_20x5.tif | 122 | 37 | 62 | 23 | 55 | 30,32786885 | 50,8196721 | 18,852459 | |
| | | | | | Mean | 25,19264115 | 59,6974069 | 15,1099519 | 1,507246377 |
| 3D_20x.tif | 204 | 8 | 165 | 31 | 18 | 3,921568627 | 80,8823529 | 15,1960784 | |
| 3D_20x2.tif | 161 | 6 | 91 | 64 | 8 | 3,726708075 | 56,5217391 | 39,7515528 | |
| 3D_20x6.tif | 111 | 5 | 85 | 21 | 19 | 4,504504505 | 76,5765766 | 18,9189189 | |
| 3D_20x8.tif | 178 | 104 | 36 | 38 | 259 | 58,42696629 | 20,2247191 | 21,3483146 | |
| 3D_20x10.tif | 123 | 63 | 34 | 26 | 157 | 51,2195122 | 27,6422764 | 21,1382114 | |
| 3D_20x11.tif | 175 | 38 | 91 | 46 | 92 | 21,71428571 | 52 | 26,2857143 | |
| 3D_20x12.tif | 157 | 42 | 80 | 35 | 111 | 26,75159236 | 50,955414 | 22,2929936 | |
| | | | | | Mean | 24,32359111 | 52,1147255 | 23,5616834 | 2,496240602 |
| 3E_20x.tif | 168 | 54 | 68 | 46 | 86 | 32,14285714 | 40,4761905 | 27,3809524 | |
| 3E_20x3.tif | 190 | 41 | 115 | 34 | 58 | 21,57894737 | 60,5263158 | 17,8947368 | |
| 3E_20x5.tif | 181 | 33 | 90 | 58 | 85 | 18,2320442 | 49,7237569 | 32,0441989 | |
| | | | | | Mean | 23,98461624 | 50,2420877 | 25,773296 | 1,7890625 |
| 4A_20x1.tif | 192 | 48 | 97 | 47 | 88 | 25 | 50,5208333 | 24,4791667 | |
| 4A_20x2.tif | 81 | 28 | 43 | 10 | 52 | 34,56790123 | 53,0864198 | 12,345679 | |
| 4A_20x4.tif | 186 | 64 | 76 | 46 | 105 | 34,40860215 | 40,8602151 | 24,7311828 | |
| 4A_20x5.tif | 89 | 39 | 44 | 6 | 67 | 43,82022472 | 49,4382022 | 6,74157303 | |

| | | | | | | | | | |
|---|---|---|---|---|---|---|---|---|---|
| 4A_20x6.tif | 85 | 0 | 79 | 6 | 0 | 0 | 92,9411765 | 7,05882353 | |
| 4A_20x7.tif | 210 | 75 | 108 | 27 | 145 | 35,71428571 | 51,4285714 | 12,8571429 | |
| 4A_20x9.tif | 27 | 3 | 17 | 7 | 17 | 11,11111111 | 62,962963 | 25,9259259 | |
| 4A_20x11.tif | 227 | 90 | 110 | 27 | 128 | 39,64757709 | 48,4581498 | 11,8942731 | |
| 4A_20x13.tif | 116 | 30 | 61 | 25 | 62 | 25,86206897 | 52,5862069 | 21,5517241 | |
| 4A_20x17.tif | 203 | 112 | 62 | 29 | 162 | 55,17241379 | 30,5418719 | 14,2857143 | |
| 4A_20x18.tif | 215 | 36 | 131 | 48 | 50 | 16,74418605 | 60,9302326 | 22,3255814 | |
| 4A_20x22.tif | 40 | 4 | 31 | 5 | 9 | 10 | 77,5 | 12,5 | |
| | | | | | Mean | 27,67069757 | 55,9379035 | 16,3913989 | 1,672967864 |
| 4B_20x1.tif | 106 | 60 | 41 | 5 | 49 | 56,60377358 | 38,6792453 | 4,71698113 | |
| 4B_20x2.tif | 177 | 63 | 96 | 18 | 53 | 35,59322034 | 54,2372881 | 10,1694915 | |
| 4B_20x4.tif | 193 | 104 | 86 | 3 | 56 | 53,88601036 | 44,5595855 | 1,55440415 | |
| 4B_20x5.tif | 158 | 50 | 62 | 46 | 65 | 31,64556962 | 39,2405063 | 29,1139241 | |
| 4B_20x9.tif | 130 | 19 | 93 | 18 | 11 | 14,61538462 | 71,5384615 | 13,8461538 | |
| 4B_20x10.tif | 185 | 89 | 66 | 30 | 68 | 48,10810811 | 35,6756757 | 16,2162162 | |
| 4B_20x11.tif | 273 | 109 | 110 | 54 | 65 | 39,92673993 | 40,2930403 | 19,7802198 | |
| | | | | | Mean | 40,05411522 | 46,3176861 | 13,6281987 | 0,74291498 |
| 4C_20x1.tif | 229 | 54 | 138 | 37 | 67 | 23,58078603 | 60,2620087 | 16,1572052 | |
| 4C_20x4.tif | 226 | 116 | 74 | 36 | 204 | 51,32743363 | 32,7433628 | 15,9292035 | |
| 4C_20x5.tif | 102 | 57 | 33 | 12 | 91 | 55,88235294 | 32,3529412 | 11,7647059 | |
| 4C_20x7.tif | 215 | 82 | 95 | 38 | 100 | 38,13953488 | 44,1860465 | 17,6744186 | |
| | | | | | Mean | 42,23252687 | 42,3860898 | 15,3813833 | 1,495145631 |
| 4D_20x1.tif | 114 | 31 | 60 | 23 | 54 | 27,19298246 | 52,6315789 | 20,1754386 | |

| | | | | | | | | | |
|---|---|---|---|---|---|---|---|---|---|
| 4D_20x3.tif | 109 | 23 | 59 | 27 | 45 | 21,10091743 | 54,1284404 | 24,7706422 | |
| 4D_20x4.tif | 195 | 8 | 121 | 66 | 29 | 4,102564103 | 62,0512821 | 33,8461538 | |
| 4D_20x7.tif | 124 | 5 | 73 | 46 | 14 | 4,032258065 | 58,8709677 | 37,0967742 | |
| | | | | | Mean | 14,10718051 | 56,9205673 | 28,9722522 | 2,119402985 |
| 4E_20x.tif | 176 | 29 | 83 | 64 | 75 | 16,47727273 | 47,1590909 | 36,3636364 | |
| 4E_20x2.tif | 196 | 15 | 121 | 60 | 32 | 7,653061224 | 61,7346939 | 30,6122449 | |
| 4E_20x5.tif | 118 | 36 | 31 | 51 | 92 | 30,50847458 | 26,2711864 | 43,220339 | |
| 4E_20x6.tif | 134 | 18 | 74 | 42 | 54 | 13,43283582 | 55,2238806 | 31,3432836 | |
| 4E_20x7.tif | 208 | 41 | 116 | 51 | 68 | 19,71153846 | 55,7692308 | 24,5192308 | |
| 4E_20x11.tif | 253 | 35 | 134 | 84 | 104 | 13,83399209 | 52,9644269 | 33,201581 | |
| 4E_20x12.tif | 133 | 23 | 45 | 65 | 54 | 17,29323308 | 33,8345865 | 48,8721805 | |
| | | | | | Mean | 16,98720114 | 47,5652994 | 35,4474994 | 2,431472081 |
| 5A_20x.tif | 242 | 123 | 103 | 16 | 118 | 50,82644628 | 42,5619835 | 6,61157025 | |
| 5A_20x2.tif | 287 | 163 | 113 | 11 | 168 | 56,79442509 | 39,3728223 | 3,83275261 | |
| 5A_20x3.tif | 78 | 54 | 24 | 0 | 65 | 69,23076923 | 30,7692308 | 0 | |
| 5A_20x4.tif | 156 | 73 | 78 | 5 | 99 | 46,79487179 | 50 | 3,20512821 | |
| 5A_20x6.tif | 64 | 31 | 24 | 9 | 47 | 48,4375 | 37,5 | 14,0625 | |
| 5A_20x7.tif | 213 | 132 | 68 | 13 | 175 | 61,97183099 | 31,9248826 | 6,10328638 | |
| 5A_20x10.tif | 50 | 10 | 26 | 14 | 13 | 20 | 52 | 28 | |
| 5A_20x14.tif | 222 | 140 | 78 | 4 | 152 | 63,06306306 | 35,1351351 | 1,8018018 | |
| 5A_20x17.tif | 98 | 52 | 39 | 7 | 49 | 53,06122449 | 39,7959184 | 7,14285714 | |
| 5A_20x18.tif | 197 | 78 | 108 | 11 | 89 | 39,59390863 | 54,822335 | 5,58375635 | |
| 5A_20x19.tif | 171 | 89 | 63 | 19 | 92 | 52,04678363 | 36,8421053 | 11,1111111 | |
| | | | | | Mean | 51,07462029 | 40,9749466 | 7,95043308 | 1,129100529 |

| | | | | | | | | | | |
|---|---|---|---|---|---|---|---|---|---|---|
| 5B_20x.tif | 264 | 165 | 58 | 41 | 81 | | 62,5 | 21,969697 | 15,530303 | |
| 5B_20x1.tif | 85 | 40 | 38 | 7 | 22 | | 47,05882353 | 44,7058824 | 8,23529412 | |
| 5B_20x2.tif | 152 | 91 | 42 | 19 | 43 | | 59,86842105 | 27,6315789 | 12,5 | |
| 5B_20x3.tif | 114 | 69 | 45 | 0 | 44 | | 60,52631579 | 39,4736842 | 0 | |
| 5B_20x4.tif | 199 | 127 | 55 | 17 | 27 | | 63,81909548 | 27,638191 | 8,54271357 | |
| 5B_20x5.tif | 130 | 67 | 30 | 33 | 35 | | 51,53846154 | 23,0769231 | 25,3846154 | |
| 5B_20x8.tif | 77 | 48 | 29 | 0 | 26 | | 62,33766234 | 37,6623377 | 0 | |
| 5B_20x14.tif | 165 | 73 | 62 | 30 | 28 | | 44,24242424 | 37,5757576 | 18,1818182 | |
| 5B_20x16.tif | 160 | 35 | 72 | 53 | 14 | | 21,875 | 45 | 33,125 | |
| | | | | | | Mean | 52,64068933 | 33,8593391 | 13,4999716 | 0,447552448 |
| 5C_20x1.tif | 117 | 56 | 45 | 16 | 74 | | 47,86324786 | 38,4615385 | 13,6752137 | |
| 5C_20x4.tif | 31 | 13 | 10 | 8 | 11 | | 41,93548387 | 32,2580645 | 25,8064516 | |
| 5C_20x6.tif | 124 | 1 | 71 | 52 | 7 | | 0,806451613 | 57,2580645 | 41,9354839 | |
| 5C_20x7.tif | 148 | 42 | 61 | 45 | 41 | | 28,37837838 | 41,2162162 | 30,4054054 | |
| 5C_20x8.tif | 29 | 14 | 11 | 4 | 21 | | 48,27586207 | 37,9310345 | 13,7931034 | |
| | | | | | | Mean | 33,45188476 | 41,4249836 | 25,1231316 | 1,222222222 |
| 5D_20x1.tif | 315 | 92 | 149 | 74 | 165 | | 29,20634921 | 47,3015873 | 23,4920635 | |
| 5D_20x2.tif | 232 | 75 | 104 | 53 | 107 | | 32,32758621 | 44,8275862 | 22,8448276 | |
| 5D_20x5.tif | 194 | 62 | 98 | 34 | 105 | | 31,95876289 | 50,5154639 | 17,5257732 | |
| 5D_20x6.tif | 129 | 46 | 69 | 14 | 50 | | 35,65891473 | 53,4883721 | 10,8527132 | |
| 5D_20x8.tif | 111 | 30 | 74 | 7 | 46 | | 27,02702703 | 66,6666667 | 6,30630631 | |
| 5D_20x9.tif | 168 | 69 | 83 | 16 | 94 | | 41,07142857 | 49,4047619 | 9,52380952 | |
| 5D_20x10.tif | 148 | 66 | 71 | 11 | 105 | | 44,59459459 | 47,972973 | 7,43243243 | |
| | | | | | | Mean | 34,5492376 | 51,4539159 | 13,9968465 | 1,527272727 |

| | | | | | | | | | |
|---|---|---|---|---|---|---|---|---|---|
| 5E_20x.tif | 163 | 56 | 75 | 32 | 86 | 34,35582822 | 46,0122699 | 19,6319018 | |
| 5E_20x1.tif | 192 | 49 | 124 | 19 | 77 | 25,52083333 | 64,5833333 | 9,89583333 | |
| 5E_20x4.tif | 123 | 23 | 65 | 35 | 58 | 18,69918699 | 52,8455285 | 28,4552846 | |
| 5E_20x5.tif | 109 | 45 | 53 | 11 | 94 | 41,28440367 | 48,6238532 | 10,0917431 | |
| 5E_20x8.tif | 182 | 104 | 64 | 14 | 185 | 57,14285714 | 35,1648352 | 7,69230769 | |
| | | | | | Mean | 35,40062187 | 49,445964 | 15,1534141 | 1,805054152 |

| | | | | | | | | | |
|---|---|---|---|---|---|---|---|---|---|
| 6A_20x.tif | 179 | 83 | 66 | 30 | 84 | 46,36871508 | 36,8715084 | 16,7597765 | |
| 6A_20x1.tif | 149 | 89 | 32 | 28 | 39 | 59,73154362 | 21,4765101 | 18,7919463 | |
| 6A_20x2.tif | 110 | 68 | 23 | 19 | 43 | 61,81818182 | 20,9090909 | 17,2727273 | |
| 6A_20x3.tif | 139 | 81 | 31 | 27 | 32 | 58,27338129 | 22,3021583 | 19,4244604 | |
| 6A_20x5.tif | 101 | 53 | 24 | 24 | 20 | 52,47524752 | 23,7623762 | 23,7623762 | |
| 6A_20x6.tif | 139 | 75 | 23 | 41 | 18 | 53,95683453 | 16,5467626 | 29,4964029 | |
| 6A_20x10.tif | 51 | 0 | 21 | 30 | 0 | 0 | 41,1764706 | 58,8235294 | |
| 6A_20x14.tif | 58 | 47 | 11 | 0 | 3 | 81,03448276 | 18,9655172 | 0 | |
| 6A_20x16.tif | 93 | 3 | 39 | 51 | 0 | 3,225806452 | 41,9354839 | 54,8387097 | |
| 6A_20x17.tif | 197 | 85 | 101 | 11 | 52 | 43,14720812 | 51,2690355 | 5,58375635 | |
| | | | | | Mean | 46,00314012 | 29,5214914 | 24,4753685 | 0,498287671 |

| | | | | | | | | |
|---|---|---|---|---|---|---|---|---|
| 6B_20x1.tif | 154 | 95 | 26 | 33 | 36 | 61,68831169 | 16,8831169 | 21,4285714 |
| 6B_20x2.tif | 220 | 59 | 62 | 99 | 51 | 26,81818182 | 28,1818182 | 45 |
| 6B_20x4.tif | 44 | 17 | 27 | 0 | 10 | 38,63636364 | 61,3636364 | 0 |
| 6B_20x6.tif | 178 | 35 | 77 | 66 | 28 | 19,66292135 | 43,258427 | 37,0786517 |
| 6B_20x8.tif | 144 | 41 | 27 | 76 | 34 | 28,47222222 | 18,75 | 52,7777778 |
| 6B_20x9.tif | 206 | 43 | 130 | 33 | 26 | 20,87378641 | 63,1067961 | 16,0194175 |
| 6B_20x14.tif | 172 | 92 | 64 | 16 | 89 | 53,48837209 | 37,2093023 | 9,30232558 |
| 6B_20x15.tif | 171 | 138 | 26 | 7 | 124 | 80,70175439 | 15,2046784 | 4,09356725 |

| | | | | | | | | | | |
|---|---|---|---|---|---|---|---|---|---|---|
| 6B_20x16.tif | 162 | 91 | 52 | 19 | 76 | | 56,17283951 | 32,0987654 | 11,7283951 | |
| 6B_20x20.tif | 225 | 90 | 81 | 54 | 71 | | 40 | 36 | 24 | |
| 6B_20x21.tif | 179 | 45 | 94 | 40 | 6 | | 25,1396648 | 52,5139665 | 22,3463687 | |
| 6B_20x22.tif | 20 | 0 | 18 | 2 | 1 | | 0 | 90 | 10 | |
| | | | | | | Mean | 37,63786816 | 41,2142089 | 21,1479229 | 0,739946381 |
| 6C_20x.tif | 179 | 55 | 72 | 52 | 59 | | 30,72625698 | 40,2234637 | 29,0502793 | |
| 6C_20x1.tif | 85 | 54 | 20 | 11 | 23 | | 63,52941176 | 23,5294118 | 12,9411765 | |
| 6C_20x2.tif | 151 | 51 | 52 | 48 | 52 | | 33,77483444 | 34,4370861 | 31,7880795 | |
| 6C_20x3.tif | 181 | 130 | 32 | 19 | 108 | | 71,82320442 | 17,679558 | 10,4972376 | |
| 6C_20x4.tif | 65 | 52 | 8 | 5 | 40 | | 80 | 12,3076923 | 7,69230769 | |
| 6C_20x7.tif | 215 | 71 | 80 | 64 | 64 | | 33,02325581 | 37,2093023 | 29,7674419 | |
| | | | | | | Mean | 52,14616057 | 27,564419 | 20,2894204 | 0,837772397 |
| 6D_20x.tif | 160 | 52 | 80 | 28 | 82 | | 32,5 | 50 | 17,5 | |
| 6D_20x1.tif | 157 | 70 | 61 | 26 | 91 | | 44,58598726 | 38,8535032 | 16,5605096 | |
| 6D_20x3.tif | 66 | 35 | 21 | 10 | 39 | | 53,03030303 | 31,8181818 | 15,1515152 | |
| 6D_20x4.tif | 230 | 73 | 114 | 43 | 84 | | 31,73913043 | 49,5652174 | 18,6956522 | |
| 6D_20x6.tif | 207 | 87 | 88 | 32 | 86 | | 42,02898551 | 42,5120773 | 15,4589372 | |
| 6D_20x9.tif | 176 | 57 | 78 | 41 | 57 | | 32,38636364 | 44,3181818 | 23,2954545 | |
| | | | | | | Mean | 39,37846164 | 42,8445269 | 17,7770114 | 1,173796791 |
| 6E_20x2.tif | 159 | 31 | 97 | 31 | 37 | | 19,49685535 | 61,0062893 | 19,4968553 | |
| 6E_20x3.tif | 155 | 48 | 76 | 31 | 45 | | 30,96774194 | 49,0322581 | 20 | |
| 6E_20x5.tif | 213 | 81 | 100 | 32 | 96 | | 38,02816901 | 46,9483568 | 15,0234742 | |
| 6E_20x6.tif | 108 | 11 | 56 | 41 | 12 | | 10,18518519 | 51,8518519 | 37,962963 | |

| | | | | | | | | | |
|---|---|---|---|---|---|---|---|---|---|
| 6E_20x7.tif | 113 | 41 | 63 | 9 | 62 | 36,28318584 | 55,7522124 | 7,96460177 | |
| 6E_20x10.tif | 142 | 59 | 40 | 43 | 60 | 41,54929577 | 28,1690141 | 30,2816901 | |
| | | | | | Mean | 29,41840552 | 48,7933304 | 21,7882641 | 1,151291513 |
| **animal 33** | | | | | | | | | |
| 2A_20x1.tif | 242 | 32 | 159 | 51 | 12 | 13,2231405 | 65,7024793 | 21,0743802 | |
| 2A_20x2.tif | 169 | 44 | 87 | 38 | 15 | 26,03550296 | 51,4792899 | 22,4852071 | |
| 2A_20x4.tif | 139 | 5 | 77 | 57 | 4 | 3,597122302 | 55,3956835 | 41,0071942 | |
| | | | | | Mean | 14,28525525 | 57,5258176 | 28,1889272 | 0,382716049 |
| 2B_20x.tif | 194 | 109 | 60 | 25 | 0 | 56,18556701 | 30,9278351 | 12,8865979 | |
| 2B_20x1.tif | 301 | 104 | 182 | 15 | 1 | 34,55149502 | 60,4651163 | 4,9833887 | |
| 2B_20x2.tif | 242 | 13 | 229 | 0 | 0 | 5,371900826 | 94,6280992 | 0 | |
| 2B_20x5.tif | 358 | 44 | 234 | 80 | 13 | 12,29050279 | 65,3631285 | 22,3463687 | |
| 2B_20x9.tif | 246 | 120 | 84 | 42 | 4 | 48,7804878 | 34,1463415 | 17,0731707 | |
| 2B_20x10.tif | 336 | 94 | 121 | 121 | 0 | 27,97619048 | 36,0119048 | 36,0119048 | |
| 2B_20x12.tif | 311 | 67 | 142 | 102 | 4 | 21,54340836 | 45,659164 | 32,7974277 | |
| 2B_20x13.tif | 159 | 62 | 71 | 26 | 5 | 38,99371069 | 44,6540881 | 16,3522013 | |
| 2B_20x15.tif | 55 | 38 | 17 | 0 | 7 | 69,09090909 | 30,9090909 | 0 | |
| 2B_20x16.tif | 344 | 111 | 172 | 61 | 8 | 32,26744186 | 50 | 17,7325581 | |
| 2B_20x17.tif | 31 | 18 | 13 | 0 | 2 | 58,06451613 | 41,9354839 | 0 | |
| 2B_20x22.tif | 305 | 109 | 101 | 95 | 31 | 35,73770492 | 33,1147541 | 31,147541 | |
| 2B_20x23.tif | 149 | 95 | 39 | 15 | 33 | 63,75838926 | 26,1744966 | 10,0671141 | |
| | | | | | Mean | 38,81632494 | 45,6915002 | 15,4921748 | 0,109756098 |
| 2C_20x1.tif | 141 | 7 | 134 | 0 | 6 | 4,964539007 | 95,035461 | 0 | |

| | | | | | | | | | | |
|---|---|---|---|---|---|---|---|---|---|---|
| 2C_20x2.tif | 315 | 107 | 143 | 65 | 123 | | 33,96825397 | 45,3968254 | 20,6349206 | |
| 2C_20x3.tif | 319 | 96 | 141 | 82 | 111 | | 30,09404389 | 44,200627 | 25,7053292 | |
| 2C_20x4.tif | 236 | 87 | 97 | 52 | 43 | | 36,86440678 | 41,1016949 | 22,0338983 | |
| 2C_20x6.tif | 250 | 83 | 149 | 18 | 21 | | 33,2 | 59,6 | 7,2 | |
| 2C_20x8.tif | 299 | 2 | 199 | 98 | 2 | | 0,668896321 | 66,5551839 | 32,7759197 | |
| | | | | | | Mean | 23,29335666 | 58,6482987 | 18,0583446 | 0,631399317 |
| 2D_20x1.tif | 110 | 32 | 7 | 71 | 21 | | 29,09090909 | 6,36363636 | 64,5454545 | |
| 2D_20x3.tif | 75 | 19 | 22 | 34 | 5 | | 25,33333333 | 29,3333333 | 45,3333333 | |
| | | | | | | Mean | 27,21212121 | 17,8484848 | 54,9393939 | 0,509803922 |
| 2E_20x.tif | 233 | 2 | 0 | 231 | 1 | | 0,858369099 | 0 | 99,1416309 | |
| 2E_20x1.tif | 198 | 0 | 0 | 198 | 0 | | 0 | 0 | 100 | |
| 2E_20x4.tif | 235 | 38 | 159 | 38 | 11 | | 16,17021277 | 67,6595745 | 16,1702128 | |
| 2E_20x5.tif | 297 | 79 | 65 | 153 | 32 | | 26,5993266 | 21,8855219 | 51,5151515 | |
| | | | | | | Mean | 10,90697712 | 22,3862741 | 66,7067488 | 0,369747899 |
| 3A_20x.tif | 196 | 27 | 102 | 67 | 4 | | 13,7755102 | 52,0408163 | 34,1836735 | |
| 3A_20x2.tif | 263 | 93 | 145 | 25 | 23 | | 35,36121673 | 55,1330798 | 9,50570342 | |
| | | | | | | Mean | 24,56836347 | 53,5869481 | 21,8446884 | 0,225 |
| 3B_20x.tif | 210 | 112 | 79 | 19 | 4 | | 53,33333333 | 37,6190476 | 9,04761905 | |
| 3B_20x3.tif | 90 | 43 | 34 | 13 | 14 | | 47,77777778 | 37,7777778 | 14,4444444 | |

| | | | | | | | | | |
|---|---|---|---|---|---|---|---|---|---|
| 3B_20x4.tif | 245 | 129 | 98 | 18 | 6 | 52,65306122 | 40 | 7,34693878 | |
| 3B_20x5.tif | 170 | 73 | 91 | 6 | 2 | 42,94117647 | 53,5294118 | 3,52941176 | |
| 3B_20x8.tif | 93 | 57 | 34 | 2 | 2 | 61,29032258 | 36,5591398 | 2,15053763 | |
| | | | | | Mean | 51,59913428 | 41,0970754 | 7,30379033 | 0,06763285 |
| 3C_20x1.tif | 159 | 61 | 86 | 12 | 27 | 38,36477987 | 54,0880503 | 7,54716981 | |
| 3C_20x2.tif | 134 | 30 | 82 | 22 | 7 | 22,3880597 | 61,1940299 | 16,4179104 | |
| 3C_20x3.tif | 271 | 52 | 156 | 63 | 28 | 19,18819188 | 57,5645756 | 23,2472325 | |
| 3C_20x5.tif | 219 | 53 | 112 | 54 | 29 | 24,20091324 | 51,1415525 | 24,6575342 | |
| 3C_20x7.tif | 59 | 19 | 28 | 12 | 10 | 32,20338983 | 47,4576271 | 20,3389831 | |
| | | | | | Mean | 27,26906691 | 54,2891671 | 18,441766 | 0,469767442 |
| 3D_20x1.tif | 241 | 3 | 224 | 14 | 0 | 1,244813278 | 92,9460581 | 5,80912863 | |
| 3D_20x2.tif | 272 | 56 | 38 | 178 | 37 | 20,58823529 | 13,9705882 | 65,4411765 | |
| 3D_20x3.tif | 276 | 24 | 39 | 213 | 18 | 8,695652174 | 14,1304348 | 77,173913 | |
| 3D_20x7.tif | 112 | 40 | 54 | 18 | 12 | 35,71428571 | 48,2142857 | 16,0714286 | |
| 3D_20x8.tif | 62 | 4 | 12 | 46 | 1 | 6,451612903 | 19,3548387 | 74,1935484 | |
| | | | | | Mean | 14,53891987 | 37,7232411 | 47,737839 | 0,535433071 |
| 3E_20x.tif | 254 | 37 | 157 | 60 | 23 | 14,56692913 | 61,8110236 | 23,6220472 | |
| 3E_20x1.tif | 133 | 60 | 37 | 36 | 25 | 45,11278195 | 27,8195489 | 27,0676692 | |
| 3E_20x2.tif | 129 | 50 | 30 | 49 | 16 | 38,75968992 | 23,255814 | 37,9844961 | |
| 3E_20x6.tif | 161 | 52 | 50 | 59 | 18 | 32,29813665 | 31,0559006 | 36,6459627 | |
| | | | | | Mean | 32,68438441 | 35,9855718 | 31,3300438 | 0,412060302 |

| | | | | | | | | | |
|---|---|---|---|---|---|---|---|---|---|
| 4A_20x.tif | 233 | 3 | 214 | 16 | 3 | 1,287553648 | 91,8454936 | 6,86695279 | |
| 4A_20x1.tif | 159 | 16 | 102 | 41 | 10 | 10,06289308 | 64,1509434 | 25,7861635 | |
| 4A_20x5.tif | 37 | 6 | 29 | 2 | 4 | 16,21621622 | 78,3783784 | 5,40540541 | |
| 4A_20x6.tif | 195 | 47 | 118 | 30 | 15 | 24,1025641 | 60,5128205 | 15,3846154 | |
| 4A_20x9.tif | 124 | 8 | 85 | 31 | 11 | 6,451612903 | 68,5483871 | 25 | |
| 4A_20x10.tif | 198 | 24 | 119 | 55 | 7 | 12,12121212 | 60,1010101 | 27,7777778 | |
| 4A_20x12.tif | 290 | 37 | 225 | 28 | 18 | 12,75862069 | 77,5862069 | 9,65517241 | |
| 4A_20x14.tif | 61 | 0 | 52 | 9 | 0 | 0 | 85,2459016 | 14,7540984 | |
| 4A_20x17.tif | 46 | 11 | 25 | 10 | 12 | 23,91304348 | 54,3478261 | 21,7391304 | |
| 4A_20x18.tif | 172 | 93 | 60 | 19 | 87 | 54,06976744 | 34,8837209 | 11,0465116 | |
| 4A_20x19.tif | 184 | 24 | 142 | 18 | 13 | 13,04347826 | 77,173913 | 9,7826087 | |
| 4A_20x23.tif | 83 | 30 | 45 | 8 | 63 | 36,14457831 | 54,2168675 | 9,63855422 | |
| 4A_20x24.tif | 205 | 21 | 91 | 93 | 28 | 10,24390244 | 44,3902439 | 45,3658537 | |
| | | | | | Mean | 16,95503405 | 65,490901 | 17,5540649 | 0,846875 |
| 4C_20x.tif | 195 | 41 | 126 | 28 | 26 | 21,02564103 | 64,6153846 | 14,3589744 | |
| 4C_20x2.tif | 226 | 0 | 226 | 0 | 0 | 0 | 100 | 0 | |
| | | | | | Mean | 10,51282051 | 82,3076923 | 7,17948718 | 0,634146341 |
| 4D_20x.tif | 188 | 63 | 35 | 90 | 20 | 33,5106383 | 18,6170213 | 47,8723404 | |
| 4D_20x1.tif | 114 | 21 | 35 | 58 | 10 | 18,42105263 | 30,7017544 | 50,877193 | |
| 4D_20x3.tif | 206 | 61 | 56 | 89 | 16 | 29,61165049 | 27,184466 | 43,2038835 | |
| | | | | | Mean | 27,1811138 | 25,5010806 | 47,3178056 | 0,317241379 |

| | | | | | | | | | | |
|---|---|---|---|---|---|---|---|---|---|---|
| 4E_20x.tif | 93 | 14 | 42 | 37 | 0 | | 15,05376344 | 45,1612903 | 39,7849462 | |
| 4E_20x2.tif | 45 | 10 | 12 | 23 | 0 | | 22,22222222 | 26,6666667 | 51,1111111 | |
| 4E_20x5.tif | 124 | 32 | 86 | 6 | 7 | | 25,80645161 | 69,3548387 | 4,83870968 | |
| 4E_20x6.tif | 227 | 50 | 75 | 102 | 3 | | 22,02643172 | 33,0396476 | 44,9339207 | |
| | | | | | | Mean | 21,27721725 | 43,5556108 | 35,1671719 | 0,094339623 |
| | | | | | | | | | | |
| 5A_20x1.tif | 320 | 33 | 203 | 84 | 29 | | 10,3125 | 63,4375 | 26,25 | |
| 5A_20x2.tif | 53 | 12 | 35 | 6 | 1 | | 22,64150943 | 66,0377358 | 11,3207547 | |
| 5A_20x5.tif | 263 | 87 | 170 | 6 | 52 | | 33,07984791 | 64,6387833 | 2,28136882 | |
| 5A_20x8.tif | 137 | 36 | 93 | 8 | 46 | | 26,27737226 | 67,8832117 | 5,83941606 | |
| 5A_20x10.tif | 188 | 26 | 136 | 26 | 29 | | 13,82978723 | 72,3404255 | 13,8297872 | |
| 5A_20x11.tif | 104 | 8 | 46 | 50 | 14 | | 7,692307692 | 44,2307692 | 48,0769231 | |
| 5A_20x12.tif | 146 | 0 | 80 | 66 | 0 | | 0 | 54,7945205 | 45,2054795 | |
| | | | | | | Mean | 16,2619035 | 61,9089923 | 21,8291042 | 0,846534653 |
| | | | | | | | | | | |
| 5B_20x1.tif | 171 | 117 | 54 | 0 | 9 | | 68,42105263 | 31,5789474 | 0 | |
| 5B_20x2.tif | 156 | 90 | 60 | 6 | 3 | | 57,69230769 | 38,4615385 | 3,84615385 | |
| | | | | | | Mean | 63,05668016 | 35,0202429 | 1,92307692 | 0,057971014 |
| | | | | | | | | | | |
| 5C_20x.tif | 215 | 22 | 155 | 38 | 46 | | 10,23255814 | 72,0930233 | 17,6744186 | |
| 5C_20x1.tif | 269 | 6 | 189 | 74 | 7 | | 2,230483271 | 70,260223 | 27,5092937 | |
| | | | | | | Mean | 6,231520705 | 71,1766232 | 22,5918561 | 1,892857143 |

| | | | | | | | | | |
|---|---|---|---|---|---|---|---|---|---|
| 5D_20x.tif | 203 | 50 | 57 | 96 | 5 | 24,63054187 | 28,0788177 | 47,2906404 | |
| 5D_20x2.tif | 169 | 67 | 27 | 75 | 1 | 39,64497041 | 15,9763314 | 44,3786982 | |
| | | | | | Mean | 32,13775614 | 22,0275745 | 45,8346693 | 0,051282051 |
| 5E_20x.tif | 234 | 130 | 37 | 67 | 15 | 55,55555556 | 15,8119658 | 28,6324786 | |
| 5E_20x1.tif | 131 | 15 | 21 | 95 | 2 | 11,45038168 | 16,0305344 | 72,519084 | |
| 5E_20x2.tif | 141 | 9 | 44 | 88 | 3 | 6,382978723 | 31,2056738 | 62,4113475 | |
| 5E_20x6.tif | 117 | 5 | 36 | 76 | 1 | 4,273504274 | 30,7692308 | 64,957265 | |
| | | | | | Mean | 19,41560506 | 23,4543512 | 57,1300438 | 0,132075472 |
| 6A_20x.tif | 116 | 0 | 110 | 6 | 0 | 0 | 94,8275862 | 5,17241379 | |
| 6A_20x1.tif | 210 | 26 | 156 | 28 | 12 | 12,38095238 | 74,2857143 | 13,3333333 | |
| 6A_20x2.tif | 91 | 10 | 70 | 11 | 4 | 10,98901099 | 76,9230769 | 12,0879121 | |
| 6A_20x3.tif | 47 | 0 | 27 | 20 | 0 | 0 | 57,4468085 | 42,5531915 | |
| 6A_20x5.tif | 164 | 13 | 121 | 30 | 9 | 7,926829268 | 73,7804878 | 18,2926829 | |
| | | | | | Mean | 6,259358528 | 75,4527347 | 18,2879067 | 0,510204082 |
| 6B_20x1.tif | 178 | 41 | 82 | 55 | 1 | 23,03370787 | 46,0674157 | 30,8988764 | |
| 6B_20x3.tif | 146 | 72 | 57 | 17 | 2 | 49,31506849 | 39,0410959 | 11,6438356 | |
| 6B_20x4.tif | 92 | 13 | 75 | 4 | 1 | 14,13043478 | 81,5217391 | 4,34782609 | |
| 6B_20x7.tif | 209 | 47 | 142 | 20 | 1 | 22,48803828 | 67,9425837 | 9,56937799 | |
| 6B_20x8.tif | 281 | 70 | 160 | 51 | 1 | 24,91103203 | 56,9395018 | 18,1494662 | |
| | | | | | Mean | 26,77565629 | 58,3024673 | 14,9218765 | 0,024691358 |

| | | | | | | | | | | |
|---|---|---|---|---|---|---|---|---|---|---|
| 6C_20x.tif | 125 | 0 | 70 | 55 | 0 | | 0 | 56 | 44 | |
| 6C_20x3.tif | 205 | 60 | 116 | 29 | 27 | | 29,26829268 | 56,5853659 | 14,1463415 | |
| 6C_20x5.tif | 105 | 0 | 97 | 8 | 0 | | 0 | 92,3809524 | 7,61904762 | |
| | | | | | | Mean | 9,756097561 | 68,3221061 | 21,9217964 | 0,45 |
| 6D_20x2.tif | 124 | 6 | 72 | 46 | 0 | | 4,838709677 | 58,0645161 | 37,0967742 | |
| 6D_20x4.tif | 221 | 87 | 106 | 28 | 17 | | 39,36651584 | 47,9638009 | 12,6696833 | |
| 6D_20x5.tif | 169 | 136 | 33 | 0 | 23 | | 80,47337278 | 19,5266272 | 0 | |
| | | | | | | Mean | 41,55953277 | 41,8516481 | 16,5888192 | 0,174672489 |
| 6E_20x.tif | 189 | 90 | 68 | 31 | 17 | | 47,61904762 | 35,978836 | 16,4021164 | |
| 6E_20x1.tif | 56 | 6 | 32 | 18 | 2 | | 10,71428571 | 57,1428571 | 32,1428571 | |
| 6E_20x3.tif | 104 | 50 | 54 | 0 | 6 | | 48,07692308 | 51,9230769 | 0 | |
| 6E_20x6.tif | 210 | 2 | 107 | 101 | 1 | | 0,952380952 | 50,952381 | 48,0952381 | |
| | | | | | | Mean | 26,84065934 | 48,9992877 | 24,1600529 | 0,175675676 |
| **animal 34** | | | | | | | | | | |
| 2A_20x1.tif | 239 | 56 | 92 | 91 | 17 | | 23,43096234 | 38,4937238 | 38,0753138 | |
| 2A_20x2.tif | 122 | 24 | 44 | 54 | 14 | | 19,67213115 | 36,0655738 | 44,2622951 | |
| 2A_20x4.tif | 172 | 78 | 94 | 0 | 24 | | 45,34883721 | 54,6511628 | 0 | |
| | | | | | | Mean | 29,4839769 | 43,0701535 | 27,4458696 | 0,348101266 |

| | | | | | | | | | |
|---|---|---|---|---|---|---|---|---|---|
| 2B_20x.tif | 218 | 75 | 131 | 12 | 24 | 34,40366972 | 60,0917431 | 5,50458716 | |
| 2B_20x1.tif | 148 | 45 | 70 | 33 | 6 | 30,40540541 | 47,2972973 | 22,2972973 | |
| 2B_20x2.tif | 155 | 42 | 89 | 24 | 22 | 27,09677419 | 57,4193548 | 15,483871 | |
| | | | | | Mean | 30,63528311 | 54,9361318 | 14,4285851 | 0,320987654 |
| 2C_20x.tif | 128 | 9 | 101 | 18 | 4 | 7,03125 | 78,90625 | 14,0625 | |
| 2C_20x1.tif | 49 | 5 | 31 | 13 | 0 | 10,20408163 | 63,2653061 | 26,5306122 | |
| | | | | | Mean | 8,617665816 | 71,0857781 | 20,2965561 | 0,285714286 |
| 2D_20x.tif | 82 | 41 | 8 | 33 | 20 | 50 | 9,75609756 | 40,2439024 | |
| 2D_20x1.tif | 128 | 63 | 0 | 65 | 30 | 49,21875 | 0 | 50,78125 | |
| | | | | | Mean | 49,609375 | 4,87804878 | 45,5125762 | 0,480769231 |
| 2E_20x2.tif | 194 | 90 | 15 | 89 | 41 | 46,39175258 | 7,73195876 | 45,8762887 | |
| 2E_20x3.tif | 159 | 25 | 23 | 111 | 14 | 15,72327044 | 14,4654088 | 69,8113208 | |
| 2E_20x6.tif | 148 | 32 | 27 | 89 | 15 | 21,62162162 | 18,2432432 | 60,1351351 | |
| 2E_20x7.tif | 102 | 46 | 12 | 44 | 20 | 45,09803922 | 11,7647059 | 43,1372549 | |
| 2E_20x8.tif | 99 | 35 | 10 | 54 | 12 | 35,35353535 | 10,1010101 | 54,5454545 | |
| | | | | | Mean | 32,83764384 | 12,4612654 | 54,7010908 | 0,447368421 |
| 3A_20x2.tif | 90 | 0 | 73 | 17 | 0 | 0 | 81,1111111 | 18,8888889 | |
| 3A_20x3.tif | 158 | 9 | 80 | 69 | 1 | 5,696202532 | 50,6329114 | 43,6708861 | |

| | | | | | | | | | | |
|---|---|---|---|---|---|---|---|---|---|---|
| 3A_20x4.tif | 133 | 12 | 65 | 56 | 0 | | 9,022556391 | 48,8721805 | 42,1052632 | |
| | | | | | | Mean | 4,906252974 | 60,205401 | 34,888346 | 0,047619048 |
| 3B_20x.tif | 229 | 93 | 87 | 49 | 0 | | 40,61135371 | 37,9912664 | 21,3973799 | |
| 3B_20x1.tif | 83 | 51 | 29 | 3 | 0 | | 61,44578313 | 34,939759 | 3,61445783 | |
| | | | | | | Mean | 51,02856842 | 36,4655127 | 12,5059189 | 0 |
| 3C_20x3.tif | 72 | 6 | 66 | 0 | 1 | | 8,333333333 | 91,6666667 | 0 | |
| 3C_20x5.tif | 188 | 45 | 141 | 2 | 8 | | 23,93617021 | 75 | 1,06382979 | |
| 3C_20x6.tif | 128 | 45 | 80 | 3 | 0 | | 35,15625 | 62,5 | 2,34375 | |
| 3C_20x7.tif | 62 | 9 | 51 | 2 | 1 | | 14,51612903 | 82,2580645 | 3,22580645 | |
| | | | | | | Mean | 20,48547064 | 77,8561828 | 1,65834656 | 0,095238095 |
| 3D_20x.tif | 58 | 3 | 39 | 16 | 1 | | 5,172413793 | 67,2413793 | 27,5862069 | |
| 3D_20x1.tif | 80 | 6 | 4 | 70 | 1 | | 7,5 | 5 | 87,5 | |
| | | | | | | Mean | 6,336206897 | 36,1206897 | 57,5431034 | 0,222222222 |
| 3E_20x.tif | 78 | 42 | 36 | 0 | 2 | | 53,84615385 | 46,1538462 | 0 | |
| | | | | | | Mean | 53,84615385 | 46,1538462 | 0 | 0,047619048 |

| | | | | | | | | | | |
|---|---|---|---|---|---|---|---|---|---|---|
| 4A_20x.tif | 192 | 45 | 101 | 46 | 3 | | 23,4375 | 52,6041667 | 23,9583333 | |
| 4A_20x1.tif | 170 | 36 | 127 | 7 | 9 | | 21,17647059 | 74,7058824 | 4,11764706 | |
| 4A_20x4.tif | 96 | 49 | 46 | 1 | 3 | | 51,04166667 | 47,9166667 | 1,04166667 | |
| 4A_20x5.tif | 152 | 64 | 83 | 5 | 8 | | 42,10526316 | 54,6052632 | 3,28947368 | |
| 4A_20x7.tif | 150 | 55 | 74 | 21 | 3 | | 36,66666667 | 49,3333333 | 14 | |
| | | | | | | Mean | 34,88551342 | 55,8330624 | 9,28142415 | 0,104417671 |
| 4B_20x.tif | 163 | 74 | 71 | 18 | 5 | | 45,39877301 | 43,5582822 | 11,0429448 | |
| 4B_20x2.tif | 131 | 9 | 122 | 0 | 0 | | 6,870229008 | 93,129771 | 0 | |
| | | | | | | Mean | 26,13450101 | 68,3440266 | 5,52147239 | 0,060240964 |
| 4C_20x.tif | 216 | 109 | 76 | 31 | 6 | | 50,46296296 | 35,1851852 | 14,3518519 | |
| 4C_20x1.tif | 197 | 77 | 109 | 11 | 3 | | 39,08629442 | 55,3299492 | 5,58375635 | |
| | | | | | | Mean | 44,77462869 | 45,2575672 | 9,9678041 | 0,048387097 |
| 4D_20x.tif | 99 | 19 | 80 | 0 | 0 | | 19,19191919 | 80,8080808 | 0 | |
| | | | | | | Mean | 19,19191919 | 80,8080808 | 0 | 0 |
| 4E_20x.tif | 173 | 0 | 0 | 173 | 0 | | 0 | 0 | 100 | |
| 4E_20x1.tif | 130 | 0 | 0 | 130 | 0 | | 0 | 0 | 100 | |
| 4E_20x2.tif | 87 | 0 | 0 | 87 | 0 | | 0 | 0 | 100 | |
| | | | | | | Mean | 0 | 0 | 100 | 0 |

| | | | | | | | | | |
|---|---|---|---|---|---|---|---|---|---|
| 5A_20x2.tif | 138 | 28 | 89 | 21 | 0 | 20,28985507 | 64,4927536 | 15,2173913 | |
| 5A_20x3.tif | 150 | 42 | 76 | 32 | 0 | 28 | 50,6666667 | 21,3333333 | |
| 5A_20x4.tif | 139 | 5 | 134 | 0 | 8 | 3,597122302 | 96,4028777 | 0 | |
| 5A_20x5.tif | 317 | 6 | 311 | 0 | 0 | 1,892744479 | 98,1072555 | 0 | |
| 5A_20x10.tif | 179 | 33 | 138 | 8 | 2 | 18,43575419 | 77,0949721 | 4,46927374 | |
| 5A_20x11.tif | 235 | 22 | 198 | 15 | 0 | 9,361702128 | 84,2553191 | 6,38297872 | |
| 5A_20x12.tif | 192 | 0 | 151 | 41 | 0 | 0 | 78,6458333 | 21,3541667 | |
| 5A_20x16.tif | 133 | 37 | 82 | 14 | 0 | 27,81954887 | 61,6541353 | 10,5263158 | |
| 5A_20x17.tif | 121 | 39 | 75 | 7 | 0 | 32,23140496 | 61,9834711 | 5,78512397 | |
| | | | | | Mean | 15,73645911 | 74,8114761 | 9,45206484 | 0,047169811 |
| 5B_20x.tif | 201 | 11 | 113 | 77 | 0 | 5,472636816 | 56,2189055 | 38,3084577 | |
| 5B_20x1.tif | 168 | 7 | 75 | 86 | 0 | 4,166666667 | 44,6428571 | 51,1904762 | |
| 5B_20x4.tif | 177 | 39 | 98 | 40 | 1 | 22,03389831 | 55,3672316 | 22,5988701 | |
| 5B_20x7.tif | 180 | 56 | 90 | 34 | 1 | 31,11111111 | 50 | 18,8888889 | |
| | | | | | Mean | 15,69607822 | 51,5572486 | 32,7466732 | 0,017699115 |
| 5C_20x1.tif | 100 | 3 | 97 | 0 | 0 | 3 | 97 | 0 | |
| 5C_20x2.tif | 153 | 4 | 144 | 5 | 0 | 2,614379085 | 94,1176471 | 3,26797386 | |
| 5C_20x5.tif | 166 | 62 | 95 | 9 | 0 | 37,34939759 | 57,2289157 | 5,42168675 | |
| | | | | | Mean | 14,32125889 | 82,7821876 | 2,89655353 | 0 |
| 5D_20x.tif | 98 | 12 | 86 | 0 | 0 | 12,24489796 | 87,755102 | 0 | |

| | | | | | | | | | |
|---|---|---|---|---|---|---|---|---|---|
| 5D_20x1.tif | 36 | 15 | 21 | 0 | 0 | 41,66666667 | 58,3333333 | 0 | |
| | | | | | Mean | 26,95578231 | 73,0442177 | 0 | 0 |
| | | | | | | | | | |
| 5E_20x.tif | 163 | 18 | 60 | 85 | 0 | 11,04294479 | 36,809816 | 52,1472393 | |
| | | | | | Mean | 11,04294479 | 36,809816 | 52,1472393 | 0 |
| | | | | | | | | | |
| 6A_20x.tif | 185 | 87 | 90 | 8 | 0 | 47,02702703 | 48,6486486 | 4,32432432 | |
| 6A_20x4.tif | 117 | 28 | 74 | 15 | 0 | 23,93162393 | 63,2478632 | 12,8205128 | |
| 6A_20x5.tif | 122 | 41 | 41 | 40 | 0 | 33,60655738 | 33,6065574 | 32,7868852 | |
| 6A_20x8.tif | 102 | 0 | 102 | 0 | 0 | 0 | 100 | 0 | |
| 6A_20x10.tif | 232 | 0 | 163 | 69 | 0 | 0 | 70,2586207 | 29,7413793 | |
| 6A_20x11.tif | 173 | 8 | 114 | 51 | 0 | 4,624277457 | 65,8959538 | 29,4797688 | |
| 6A_20x12.tif | 97 | 8 | 79 | 10 | 0 | 8,24742268 | 81,443299 | 10,3092784 | |
| | | | | | Mean | 16,77670121 | 66,1572775 | 17,0660213 | 0 |
| | | | | | | | | | |
| 6B_20x.tif | 149 | 6 | 108 | 35 | 0 | 4,026845638 | 72,4832215 | 23,4899329 | |
| 6B_20x1.tif | 115 | 54 | 53 | 8 | 0 | 46,95652174 | 46,0869565 | 6,95652174 | |
| 6B_20x2.tif | 213 | 29 | 152 | 32 | 0 | 13,61502347 | 71,3615023 | 15,0234742 | |
| 6B_20x6.tif | 132 | 26 | 79 | 27 | 0 | 19,6969697 | 59,8484848 | 20,4545455 | |
| | | | | | Mean | 21,07384014 | 62,4450413 | 16,4811186 | 0 |
| | | | | | | | | | |
| 6C_20x.tif | 225 | 23 | 192 | 10 | 0 | 10,22222222 | 85,3333333 | 4,44444444 | |
| 6C_20x2.tif | 126 | 30 | 85 | 11 | 0 | 23,80952381 | 67,4603175 | 8,73015873 | |

| | | | | | | | | | | |
|---|---|---|---|---|---|---|---|---|---|---|
| 6C_20x3.tif | 91 | 8 | 79 | 4 | 0 | | 8,791208791 | 86,8131868 | 4,3956044 | |
| 6C_20x5.tif | 177 | 37 | 124 | 16 | 0 | | 20,9039548 | 70,0564972 | 9,03954802 | |
| | | | | | | Mean | 15,93172741 | 77,4158337 | 6,6524389 | 0 |
| 6D_20x.tif | 203 | 114 | 52 | 37 | 1 | | 56,15763547 | 25,6157635 | 18,226601 | |
| | | | | | | Mean | 56,15763547 | 25,6157635 | 18,226601 | 0,00877193 |
| 6E_20x.tif | 121 | 91 | 30 | 0 | 1 | | 75,20661157 | 24,7933884 | 0 | |
| 6E_20x3.tif | 45 | 33 | 12 | 0 | 0 | | 73,33333333 | 26,6666667 | 0 | |
| | | | | | | Mean | 74,26997245 | 25,7300275 | 0 | 0,008064516 |
| **animal 36** | | | | | | | | | | |
| 2A_20x1.tif | 81 | 26 | 42 | 13 | 4 | | 32,09876543 | 51,8518519 | 16,0493827 | |
| 2A_20x3.tif | 119 | 23 | 80 | 16 | 2 | | 19,32773109 | 67,2268908 | 13,4453782 | |
| | | | | | | Mean | 25,71324826 | 59,5393713 | 14,7473804 | 0,12244898 |
| 2B_20x.tif | 209 | 26 | 141 | 42 | 5 | | 12,44019139 | 67,4641148 | 20,0956938 | |
| 2B_20x3.tif | 256 | 36 | 159 | 61 | 3 | | 14,0625 | 62,109375 | 23,828125 | |
| 2B_20x4.tif | 176 | 83 | 76 | 17 | 3 | | 47,15909091 | 43,1818182 | 9,65909091 | |
| 2B_20x6.tif | 148 | 29 | 61 | 58 | 1 | | 19,59459459 | 41,2162162 | 39,1891892 | |
| | | | | | | Mean | 23,31409422 | 53,4928811 | 23,1930247 | 0,068965517 |

| | | | | | | | | | |
|---|---|---|---|---|---|---|---|---|---|
| 2C_20x2.tif | 207 | 77 | 70 | 60 | 23 | 37,19806763 | 33,8164251 | 28,9855072 | |
| 2C_20x3.tif | 130 | 26 | 67 | 37 | 7 | 20 | 51,5384615 | 28,4615385 | |
| 2C_20x6.tif | 103 | 19 | 74 | 10 | 2 | 18,44660194 | 71,8446602 | 9,70873786 | |
| 2C_20x7.tif | 128 | 30 | 56 | 42 | 7 | 23,4375 | 43,75 | 32,8125 | |
| | | | | | Mean | 24,77054239 | 50,2373867 | 24,9920709 | 0,256578947 |
| 2D_20x1.tif | 116 | 63 | 40 | 13 | 13 | 54,31034483 | 34,4827586 | 11,2068966 | |
| 2D_20x3.tif | 45 | 27 | 10 | 8 | 9 | 60 | 22,2222222 | 17,7777778 | |
| | | | | | Mean | 57,15517241 | 28,3524904 | 14,4923372 | 0,244444444 |
| 2E_20x.tif | 172 | 84 | 52 | 36 | 9 | 48,8372093 | 30,2325581 | 20,9302326 | |
| 2E_20x2.tif | 226 | 127 | 67 | 32 | 11 | 56,19469027 | 29,6460177 | 14,159292 | |
| | | | | | Mean | 52,51594978 | 29,9392879 | 17,5447623 | 0,09478673 |
| 3A_20x.tif | 212 | 19 | 139 | 54 | 4 | 8,962264151 | 65,5660377 | 25,4716981 | |
| 3A_20x1.tif | 138 | 18 | 79 | 41 | 2 | 13,04347826 | 57,2463768 | 29,7101449 | |
| 3A_20x2.tif | 103 | 12 | 70 | 21 | 2 | 11,65048544 | 67,961165 | 20,3883495 | |
| 3A_20x4.tif | 223 | 27 | 168 | 28 | 3 | 12,10762332 | 75,3363229 | 12,5560538 | |
| 3A_20x6.tif | 104 | 2 | 102 | 0 | 0 | 1,923076923 | 98,0769231 | 0 | |
| | | | | | Mean | 9,537385618 | 72,8373651 | 17,6252493 | 0,141025641 |

| | | | | | | | | | |
|---|---|---|---|---|---|---|---|---|---|
| 3B_20x.tif | 199 | 3 | 187 | 9 | 0 | 1,507537688 | 93,9698492 | 4,52261307 | |
| 3B_20x1.tif | 130 | 13 | 96 | 21 | 0 | 10 | 73,8461538 | 16,1538462 | |
| 3B_20x4.tif | 238 | 28 | 129 | 81 | 0 | 11,76470588 | 54,2016807 | 34,0336134 | |
| 3B_20x5.tif | 194 | 50 | 111 | 33 | 3 | 25,77319588 | 57,2164948 | 17,0103093 | |
| 3B_20x8.tif | 74 | 3 | 60 | 11 | 0 | 4,054054054 | 81,0810811 | 14,8648649 | |
| | | | | | Mean | 10,6198987 | 72,0630519 | 17,3170494 | 0,030927835 |
| 3C_20x.tif | 153 | 17 | 86 | 50 | 1 | 11,11111111 | 56,2091503 | 32,6797386 | |
| 3C_20x2.tif | 175 | 28 | 84 | 63 | 3 | 16 | 48 | 36 | |
| 3C_20x4.tif | 168 | 49 | 84 | 35 | 10 | 29,16666667 | 50 | 20,8333333 | |
| 3C_20x7.tif | 130 | 30 | 64 | 36 | 4 | 23,07692308 | 49,2307692 | 27,6923077 | |
| | | | | | Mean | 19,83867521 | 50,8599799 | 29,3013449 | 0,14516129 |
| 3D_20x.tif | 161 | 8 | 26 | 127 | 1 | 4,968944099 | 16,1490683 | 78,8819876 | |
| 3D_20x2.tif | 137 | 53 | 25 | 59 | 2 | 38,68613139 | 18,2481752 | 43,0656934 | |
| 3D_20x3.tif | 132 | 53 | 13 | 66 | 4 | 40,15151515 | 9,84848485 | 50 | |
| 3D_20x7.tif | 95 | 67 | 28 | 0 | 6 | 70,52631579 | 29,4736842 | 0 | |
| | | | | | Mean | 38,58322661 | 18,4298531 | 42,9869203 | 0,071823204 |
| 3E_20x2.tif | 104 | 40 | 42 | 22 | 0 | 38,46153846 | 40,3846154 | 21,1538462 | |
| 3E_20x3.tif | 110 | 27 | 27 | 56 | 1 | 24,54545455 | 24,5454545 | 50,9090909 | |
| | | | | | Mean | 31,5034965 | 32,465035 | 36,0314685 | 0,014925373 |

| | | | | | | | | | |
|---|---|---|---|---|---|---|---|---|---|
| 4A_20x1.tif | 55 | 4 | 46 | 5 | 3 | 7,272727273 | 83,6363636 | 9,09090909 | |
| 4A_20x3.tif | 236 | 32 | 163 | 41 | 3 | 13,55932203 | 69,0677966 | 17,3728814 | |
| 4A_20x4.tif | 48 | 5 | 43 | 0 | 0 | 10,41666667 | 89,5833333 | 0 | |
| 4A_20x7.tif | 183 | 52 | 81 | 50 | 1 | 28,41530055 | 44,2622951 | 27,3224044 | |
| 4A_20x8.tif | 116 | 50 | 57 | 9 | 0 | 43,10344828 | 49,137931 | 7,75862069 | |
| 4A_20x10.tif | 171 | 34 | 120 | 17 | 1 | 19,88304094 | 70,1754386 | 9,94152047 | |
| | | | | | Mean | 20,44175096 | 67,6438597 | 11,9143893 | 0,04519774 |
| 4B_20x2.tif | 126 | 17 | 96 | 13 | 0 | 13,49206349 | 76,1904762 | 10,3174603 | |
| 4B_20x3.tif | 160 | 0 | 149 | 11 | 0 | 0 | 93,125 | 6,875 | |
| 4B_20x4.tif | 125 | 9 | 108 | 8 | 0 | 7,2 | 86,4 | 6,4 | |
| | | | | | Mean | 6,897354497 | 85,2384921 | 7,86415344 | 0 |
| 4C_20x.tif | 158 | 22 | 119 | 17 | 2 | 13,92405063 | 75,3164557 | 10,7594937 | |
| 4C_20x1.tif | 125 | 14 | 107 | 4 | 1 | 11,2 | 85,6 | 3,2 | |
| 4C_20x3.tif | 186 | 33 | 123 | 30 | 0 | 17,74193548 | 66,1290323 | 16,1290323 | |
| 4C_20x5.tif | 133 | 43 | 53 | 37 | 2 | 32,33082707 | 39,8496241 | 27,8195489 | |
| 4C_20x9.tif | 70 | 7 | 50 | 13 | 2 | 10 | 71,4285714 | 18,5714286 | |
| | | | | | Mean | 17,03936264 | 67,6647367 | 15,2959007 | 0,058823529 |
| 4D_20x2.tif | 167 | 2 | 138 | 27 | 0 | 1,19760479 | 82,6347305 | 16,1676647 | |
| 4D_20x3.tif | 97 | 32 | 30 | 35 | 4 | 32,98969072 | 30,9278351 | 36,0824742 | |
| 4D_20x4.tif | 139 | 86 | 39 | 14 | 4 | 61,8705036 | 28,057554 | 10,0719424 | |
| | | | | | Mean | 32,01926637 | 47,2067065 | 20,7740271 | 0,066666667 |

| | | | | | | | | | |
|---|---|---|---|---|---|---|---|---|---|
| 4E_20x.tif | 102 | 18 | 59 | 25 | 1 | 17,64705882 | 57,8431373 | 24,5098039 | |
| 4E_20x1.tif | 144 | 25 | 83 | 36 | 2 | 17,36111111 | 57,6388889 | 25 | |
| | | | | | Mean | 17,50408497 | 57,7410131 | 24,754902 | 0,069767442 |
| 5A_20x3.tif | 144 | 20 | 103 | 21 | 3 | 13,88888889 | 71,5277778 | 14,5833333 | |
| 5A_20x6.tif | 157 | 22 | 126 | 9 | 0 | 14,01273885 | 80,2547771 | 5,73248408 | |
| 5A_20x7.tif | 97 | 5 | 76 | 16 | 0 | 5,154639175 | 78,3505155 | 16,4948454 | |
| 5A_20x10.tif | 229 | 9 | 210 | 10 | 1 | 3,930131004 | 91,7030568 | 4,36681223 | |
| 5A_20x11.tif | 225 | 0 | 188 | 37 | 0 | 0 | 83,5555556 | 16,4444444 | |
| 5A_20x12.tif | 97 | 4 | 93 | 0 | 0 | 4,12371134 | 95,8762887 | 0 | |
| 5A_20x14.tif | 150 | 0 | 111 | 39 | 0 | 0 | 74 | 26 | |
| 5A_20x15.tif | 191 | 0 | 155 | 36 | 0 | 0 | 81,1518325 | 18,8481675 | |
| 5A_20x16.tif | 166 | 0 | 159 | 7 | 1 | 0 | 95,7831325 | 4,21686747 | |
| | | | | | Mean | 4,567789918 | 83,578104 | 11,8541061 | 0,083333333 |
| 5B_20x.tif | 185 | 35 | 135 | 15 | 1 | 18,91891892 | 72,972973 | 8,10810811 | |
| 5B_20x1.tif | 113 | 27 | 58 | 28 | 0 | 23,89380531 | 51,3274336 | 24,7787611 | |
| 5B_20x2.tif | 156 | 52 | 65 | 39 | 0 | 33,33333333 | 41,6666667 | 25 | |
| 5B_20x6.tif | 104 | 38 | 58 | 8 | 1 | 36,53846154 | 55,7692308 | 7,69230769 | |
| | | | | | Mean | 28,17112978 | 55,434076 | 16,3947942 | 0,013157895 |
| 5C_20x2.tif | 171 | 75 | 77 | 19 | 59 | 43,85964912 | 45,0292398 | 11,1111111 | |
| 5C_20x3.tif | 171 | 68 | 103 | 0 | 33 | 39,76608187 | 60,2339181 | 0 | |

| | | | | | | | | | | |
|---|---|---|---|---|---|---|---|---|---|---|
| | | | | | Mean | 41,8128655 | 52,6315789 | 5,55555556 | | 0,643356643 |
| 5D_20x.tif | 106 | 0 | 0 | 106 | 0 | 0 | 0 | 100 | | |
| | | | | | Mean | 0 | 0 | 100 | | 0 |
| 5E_20x.tif | 172 | 91 | 40 | 41 | 43 | 52,90697674 | 23,255814 | 23,8372093 | | |
| 5E_20x1.tif | 168 | 60 | 43 | 65 | 22 | 35,71428571 | 25,5952381 | 38,6904762 | | |
| | | | | | Mean | 44,31063123 | 24,425526 | 31,2638427 | | 0,430463576 |
| 6A_20x2.tif | 176 | 0 | 176 | 0 | 0 | 0 | 100 | 0 | | |
| 6A_20x3.tif | 298 | 10 | 274 | 14 | 0 | 3,355704698 | 91,9463087 | 4,69798658 | | |
| 6A_20x5.tif | 128 | 0 | 124 | 4 | 0 | 0 | 96,875 | 3,125 | | |
| 6A_20x6.tif | 248 | 10 | 182 | 56 | 0 | 4,032258065 | 73,3870968 | 22,5806452 | | |
| 6A_20x7.tif | 176 | 9 | 166 | 1 | 0 | 5,113636364 | 94,3181818 | 0,56818182 | | |
| 6A_20x10.tif | 108 | 0 | 108 | 0 | 0 | 0 | 100 | 0 | | |
| | | | | | Mean | 2,083599854 | 92,7544312 | 5,16196893 | | 0 |
| 6B_20x1.tif | 145 | 34 | 65 | 46 | 2 | 23,44827586 | 44,8275862 | 31,7241379 | | |
| 6B_20x2.tif | 245 | 45 | 129 | 71 | 1 | 18,36734694 | 52,6530612 | 28,9795918 | | |
| 6B_20x3.tif | 226 | 62 | 113 | 51 | 0 | 27,43362832 | 50 | 22,5663717 | | |
| 6B_20x6.tif | 217 | 40 | 106 | 71 | 4 | 18,43317972 | 48,8479263 | 32,718894 | | |
| | | | | | Mean | 21,92060771 | 49,0821434 | 28,9972489 | | 0,038674033 |

| | | | | | | | | | |
|---|---|---|---|---|---|---|---|---|---|
| 6C_20x.tif | 122 | 8 | 62 | 52 | 0 | 6,557377049 | 50,8196721 | 42,6229508 | |
| 6C_20x2.tif | 221 | 31 | 150 | 40 | 2 | 14,02714932 | 67,8733032 | 18,0995475 | |
| 6C_20x4.tif | 176 | 14 | 155 | 7 | 0 | 7,954545455 | 88,0681818 | 3,97727273 | |
| 6C_20x5.tif | 259 | 2 | 257 | 0 | 0 | 0,772200772 | 99,2277992 | 0 | |
| | | | | | Mean | 7,327818149 | 76,4972391 | 16,1749428 | 0,036363636 |
| 6D_20x.tif | 105 | 28 | 54 | 23 | 2 | 26,66666667 | 51,4285714 | 21,9047619 | |
| 6D_20x1.tif | 135 | 11 | 75 | 49 | 0 | 8,148148148 | 55,5555556 | 36,2962963 | |
| | | | | | Mean | 17,40740741 | 53,4920635 | 29,1005291 | 0,051282051 |
| 6E_20x2.tif | 70 | 38 | 19 | 13 | 5 | 54,28571429 | 27,1428571 | 18,5714286 | |
| 6E_20x3.tif | 147 | 56 | 31 | 60 | 6 | 38,0952381 | 21,0884354 | 40,8163265 | |
| 6E_20x4.tif | 108 | 52 | 27 | 29 | 7 | 48,14814815 | 25 | 26,8518519 | |
| 6E_20x5.tif | 215 | 95 | 64 | 56 | 13 | 44,18604651 | 29,7674419 | 26,0465116 | |
| 6E_20x6.tif | 144 | 11 | 124 | 9 | 0 | 7,638888889 | 86,1111111 | 6,25 | |
| | | | | | Mean | 38,47080719 | 37,8219691 | 23,7072237 | 0,123015873 |

| | % Seminiferous tubules | % Interstitial tissue | % Necrotic Tissue | Normalized spermatogonial cell number |
|---|---|---|---|---|
| **D0** | | | | |
| animal 13 | 80,9878096 | 19,0121904 | | 1,319230769 |
| animal 14 | 73,20279071 | 26,79720929 | | 0,799191375 |
| animal 22 | 72,9451634 | 27,0548366 | | 0,626143791 |
| animal 33 | 77,86505279 | 22,13494721 | | 0,633049818 |
| animal 34 | 75,3501811 | 24,6498189 | | 0,556818182 |
| animal 36 | 75,67779175 | 24,32220825 | | 0,676277851 |
| | | | | |
| **animal 13** | | | | |
| 3A | 47,31234755 | 25,21988179 | 27,46777066 | 0,197568389 |
| 3B | 15,23283213 | 68,7566292 | 16,01053867 | 0,174418605 |
| 3C | 28,02060705 | 45,72345293 | 26,25594001 | 0,128205128 |
| 3D | 13,20224719 | 59,54150054 | 27,25625227 | 0,234042553 |
| 3E | 35,87153053 | 46,43287641 | 17,69559305 | 0,25 |
| 4A | 34,12778811 | 41,47747064 | 24,39474125 | 0,184135977 |
| 4B | 40,40103151 | 52,0127616 | 7,586206897 | 0,01754386 |
| 4C | 40,86812856 | 57,43512814 | 1,696743305 | 0,195121951 |
| 4D | 27,71514675 | 66,93763103 | 5,347222222 | 0,401639344 |
| 4E | 17,15989098 | 61,15582446 | 21,68428455 | 0,372262774 |
| 5A | 35,27495002 | 49,79591128 | 14,92913871 | 0,204724409 |
| 5B | 32,58628224 | 52,79597757 | 14,61774018 | 0,03943662 |
| 5C | 54,31183387 | 42,3347651 | 3,353401032 | 0,146723647 |
| 5D | 45,57693918 | 40,78622621 | 13,63683461 | 0,017142857 |
| 5E | 44,64237517 | 53,51551957 | 1,842105263 | 0,140625 |
| 6A | 35,95498296 | 64,04501704 | 0 | 0,032994924 |
| 6B | 17,85862235 | 72,66178007 | 9,479597579 | 0,032520325 |
| 6C | 28,60378452 | 70,9131237 | 0,483091787 | 0,053333333 |
| 6D | 48,56764564 | 40,87225165 | 10,56010271 | 0,124324324 |
| 6E | 70,93037728 | 23,60113198 | 5,46849074 | 0,057432432 |
| | | | | |
| **animal 14** | | | | |
| 2A | 59,21903079 | 29,44919522 | 11,33177399 | 0,679012346 |
| 2B | 39,85860963 | 48,52100141 | 11,62038896 | 0,319634703 |
| 2C (nd) | | | | |
| 2D | 23,68598778 | 49,33708626 | 26,97692596 | 0,727272727 |
| 2E | 24,2747503 | 32,84690742 | 42,87834228 | 0,72826087 |
| 3A | 27,50833942 | 27,50833942 | 26,8850774 | 0,537790698 |
| 3B | 20,56201873 | 58,40481365 | 21,03316762 | 0,114754098 |
| 3C | 42,29687373 | 29,90988376 | 27,79324251 | 0,427331887 |
| 3D | 13,44716948 | 76,61239528 | 9,940435241 | 0,333333333 |
| 3E | 22,08608391 | 49,19731649 | 28,7165996 | 0,617283951 |
| 4A | 28,818647 | 50,80990252 | 20,37145048 | 0,370940171 |
| 4B | 10,14510469 | 63,75298739 | 26,10190792 | 0,367647059 |
| 4C | 14,5918808 | 49,45109852 | 35,95702068 | 0,486238532 |

| | | | | |
|---|---|---|---|---|
| 4D | 24,46340794 | 48,69946594 | 26,83712612 | 0,681318681 |
| 4E | 11,06147576 | 59,28199317 | 29,65653107 | 0,387755102 |
| 5A | 33,76898722 | 60,50168694 | 5,729325848 | 0,317124736 |
| 5B | 29,48248325 | 56,95115119 | 13,56636556 | 0,036231884 |
| 5C | 38,65364352 | 59,92468817 | 1,421668304 | 0,507886435 |
| 5D | 23,62685344 | 58,49454204 | 17,87860452 | 0,414634146 |
| 5E | 4,575785887 | 74,84072663 | 20,58348748 | 0,666666667 |
| 6A | 30,39342641 | 50,15512062 | 19,45145298 | 0,100854701 |
| 6B | 24,49017679 | 56,88905547 | 18,62076774 | 0,081395349 |
| 6C | 14,67515253 | 71,05376272 | 14,27108475 | 0,224043716 |
| 6D | 34,85234023 | 52,74132449 | 12,40633528 | 0,517857143 |
| 6E | 39,64145809 | 40,33637165 | 20,02217026 | 0,616161616 |
| | | | | |
| **animal 22** | | | | |
| 2A | 46,68946973 | 44,64567823 | 8,664852033 | 1,453107345 |
| 2B | 66,66494016 | 29,3818982 | 3,953161637 | 0,856175973 |
| 2C | 46,19270752 | 37,9612006 | 15,84609188 | 1,820359281 |
| 2D | 44,01325959 | 39,76827665 | 16,21846377 | 1,55801105 |
| 2E | 31,45527627 | 38,08348366 | 30,46124007 | 2,256809339 |
| 3A | 40,40185146 | 40,90455552 | 18,69359301 | 1,541626794 |
| 3B | 46,42712439 | 36,15951557 | 17,41336004 | 0,649301144 |
| 3C | 25,19264115 | 59,69740691 | 15,10995194 | 1,507246377 |
| 3D | 24,32359111 | 52,11472545 | 23,56168344 | 2,496240602 |
| 3E | 23,98461624 | 50,24208772 | 25,77329604 | 1,7890625 |
| 4A | 27,67069757 | 55,93790353 | 16,3913989 | 1,672967864 |
| 4B | 40,05411522 | 46,31768611 | 13,62819867 | 0,74291498 |
| 4C | 42,23252687 | 42,38608981 | 15,38138332 | 1,495145631 |
| 4D | 14,10718051 | 56,92056728 | 28,97225221 | 2,119402985 |
| 4E | 16,98720114 | 47,56529942 | 35,44749944 | 2,431472081 |
| 5A | 51,07462029 | 40,97494663 | 7,950433077 | 1,129100529 |
| 5B | 52,64068933 | 33,85933908 | 13,49997159 | 0,334265734 |
| 5C | 33,45188476 | 41,42498364 | 25,1231316 | 1,222222222 |
| 5D | 34,5492376 | 51,45391587 | 13,99684653 | 1,527272727 |
| 5E | 35,40062187 | 49,44596402 | 15,15341411 | 1,805054152 |
| 6A | 46,00314012 | 29,52149137 | 24,47536851 | 0,498287671 |
| 6B | 37,63786816 | 41,21420893 | 21,14792291 | 0,739946381 |
| 6C | 52,14616057 | 27,56441903 | 20,2894204 | 0,837772397 |
| 6D | 39,37846164 | 42,84452692 | 17,77701144 | 1,173796791 |
| 6E | 29,41840552 | 48,79333042 | 21,78826407 | 1,151291513 |
| | | | | |
| **animal 33** | | | | |
| 2A | 14,28525525 | 57,52581758 | 28,18892717 | 0,382716049 |
| 2B | 38,81632494 | 45,69150021 | 15,49217484 | 0,109756098 |
| 2C | 23,29335666 | 58,6482987 | 18,05834464 | 0,80104712 |
| 2D | 27,21212121 | 17,84848485 | 54,93939394 | 0,509803922 |
| 2E | 10,90697712 | 22,38627409 | 66,7067488 | 0,369747899 |
| 3A | 24,56836347 | 53,58694809 | 21,84468845 | 0,225 |
| 3B | 51,59913428 | 41,09707539 | 7,303790333 | 0,06763285 |

| | | | | |
|---|---|---|---|---|
| 3C | 27,26906691 | 54,28916709 | 18,44176601 | 0,469767442 |
| 3D | 14,53891987 | 37,72324111 | 47,73783902 | 0,535433071 |
| 3E | 32,68438441 | 35,98557177 | 31,33004382 | 0,412060302 |
| 4A | 16,95503405 | 65,490901 | 17,55406495 | 0,846875 |
| 4B (nd) | | | | |
| 4C | 10,51282051 | 82,30769231 | 7,179487179 | 0,634146341 |
| 4D | 27,1811138 | 25,50108056 | 47,31780563 | 0,317241379 |
| 4E | 21,27721725 | 43,55561082 | 35,16717193 | 0,094339623 |
| 5A | 16,2619035 | 61,9089923 | 21,82910419 | 0,846534653 |
| 5B | 63,05668016 | 35,02024291 | 1,923076923 | 0,057971014 |
| 5C | 6,231520705 | 71,17662315 | 22,59185614 | 1,892857143 |
| 5D | 32,13775614 | 22,02757455 | 45,83466931 | 0,051282051 |
| 5E | 19,41560506 | 23,45435117 | 57,13004377 | 0,132075472 |
| 6A | 6,259358528 | 75,45273475 | 18,28790673 | 0,510204082 |
| 6B | 26,77565629 | 58,30246725 | 14,92187646 | 0,024691358 |
| 6C | 9,756097561 | 68,32210608 | 21,92179636 | 0,45 |
| 6D | 41,55953277 | 41,85164808 | 16,58881915 | 0,174672489 |
| 6E | 26,84065934 | 48,99928775 | 24,16005291 | 0,175675676 |
| | | | | |
| **animal 34** | | | | |
| 2A | 29,4839769 | 43,07015347 | 27,44586963 | 0,348101266 |
| 2B | 30,63528311 | 54,93613175 | 14,42858514 | 0,320987654 |
| 2C | 8,617665816 | 71,08577806 | 20,29655612 | 0,285714286 |
| 2D | 49,609375 | 4,87804878 | 45,51257622 | 0,480769231 |
| 2E | 32,83764384 | 12,46126536 | 54,7010908 | 0,447368421 |
| 3A | 4,906252974 | 60,20540098 | 34,88834604 | 0,047619048 |
| 3B | 51,02856842 | 36,46551271 | 12,50591887 | 0 |
| 3C | 20,48547064 | 77,8561828 | 1,65834656 | 0,095238095 |
| 3D | 6,336206897 | 36,12068966 | 57,54310345 | 0,222222222 |
| 3E | 53,84615385 | 46,15384615 | 0 | 0,047619048 |
| 4A | 34,88551342 | 55,83306244 | 9,281424149 | 0,104417671 |
| 4B | 26,13450101 | 68,3440266 | 5,521472393 | 0,060240964 |
| 4C | 44,77462869 | 45,25756721 | 9,967804099 | 0,048387097 |
| 4D | 19,19191919 | 80,80808081 | 0 | 0 |
| 4E | 0 | 0 | 100 | 0 |
| 5A | 15,73645911 | 74,81147605 | 9,452064836 | 0,047169811 |
| 5B | 15,69607822 | 51,55724856 | 32,74667321 | 0,017699115 |
| 5C | 14,32125889 | 82,78218757 | 2,896553534 | 0 |
| 5D | 26,95578231 | 73,04421769 | 0 | 0 |
| 5E | 11,04294479 | 36,80981595 | 52,14723926 | 0 |
| 6A | 16,77670121 | 66,15727753 | 17,06602126 | 0 |
| 6B | 21,07384014 | 62,4450413 | 16,48111856 | 0 |
| 6C | 15,93172741 | 77,4158337 | 6,652438898 | 0 |
| 6D | 56,15763547 | 25,61576355 | 18,22660099 | 0,00877193 |
| 6E | 74,26997245 | 25,73002755 | 0 | 0,008064516 |
| | | | | |
| **animal 36** | | | | |
| 2A | 25,71324826 | 59,5393713 | 14,74738043 | 0,12244898 |

| | | | | |
|---|---|---|---|---|
| 2B | 23,31409422 | 53,49288106 | 23,19302472 | 0,068965517 |
| 2C | 24,77054239 | 50,23738671 | 24,99207089 | 0,256578947 |
| 2D | 57,15517241 | 28,35249042 | 14,49233716 | 0,244444444 |
| 2E | 52,51594978 | 29,93928792 | 17,5447623 | 0,09478673 |
| 3A | 9,537385618 | 72,83736511 | 17,62524927 | 0,141025641 |
| 3B | 10,6198987 | 72,06305194 | 17,31704936 | 0,030927835 |
| 3C | 19,83867521 | 50,85997989 | 29,3013449 | 0,14516129 |
| 3D | 38,58322661 | 18,42985314 | 42,98692025 | 0,071823204 |
| 3E | 31,5034965 | 32,46503497 | 36,03146853 | 0,014925373 |
| 4A | 20,44175096 | 67,64385972 | 11,91438933 | 0,04519774 |
| 4B | 6,897354497 | 85,23849206 | 7,864153439 | 0 |
| 4C | 17,03936264 | 67,66473669 | 15,29590067 | 0,058823529 |
| 4D | 32,01926637 | 47,20670652 | 20,77402711 | 0,066666667 |
| 4E | 17,50408497 | 57,74101307 | 24,75490196 | 0,069767442 |
| 5A | 4,567789918 | 83,57810403 | 11,85410605 | 0,083333333 |
| 5B | 28,17112978 | 55,43407601 | 16,39479422 | 0,013157895 |
| 5C | 41,8128655 | 52,63157895 | 5,555555556 | 0,643356643 |
| 5D | 0 | 0 | 100 | 0 |
| 5E | 44,31063123 | 24,42552602 | 31,26384275 | 0,430463576 |
| 6A | 2,083599854 | 92,75443122 | 5,161968926 | 0 |
| 6B | 21,92060771 | 49,08214342 | 28,99724886 | 0,038674033 |
| 6C | 7,327818149 | 76,49723909 | 16,17494276 | 0,036363636 |
| 6D | 17,40740741 | 53,49206349 | 29,1005291 | 0,051282051 |
| 6E | 38,47080719 | 37,8219691 | 23,70722372 | 0,123015873 |

| **Animal_ID** | Animal 13 | Animal 14 | Animal 22 | Animal 33 | Animal 34 | Animal 36 | Means | SEM | MEM |
|---|---|---|---|---|---|---|---|---|---|
| Perc_ST_0 | 80,988 | 73,203 | 72,945 | 77,865 | 75,350 | 75,678 | 76,005 | 1,2388034 | KSR |
| Perc_Int_0 | 19,012 | 26,797 | 27,055 | 22,135 | 24,650 | 24,322 | 23,995 | 1,2388034 | β-estradiol |
| Perc_Nec_0 | | | | | | | | | AlbuMax II |
| SpermatogonialCells_0 | 1,319 | 0,799 | 0,626 | 0,633 | 0,557 | 0,676 | 0,768 | 0,1149163 | AlbuMax II + β-estradiol |
| MEM_Perc_ST_W2 | | 59,220 | 46,690 | 14,290 | 29,480 | 25,710 | 35,078 | 7,9652768 | |
| MEM_Perc_Int_W2 | | 29,450 | 44,650 | 57,530 | 43,070 | 59,540 | 46,848 | 5,4634553 | |
| MEM_Perc_Nec_W2 | | 11,330 | 8,660 | 28,190 | 27,450 | 14,750 | 18,076 | 4,095099 | |
| MEM_GermCells_W2 | | 0,679 | 1,453 | 0,383 | 0,348 | 0,122 | 0,597 | 0,2315979 | |
| MEM_Perc_ST_W3 | 47,310 | 27,510 | 40,400 | 24,570 | 4,910 | 9,540 | 25,707 | 6,784798 | |
| MEM_Perc_Int_W3 | 25,220 | 27,510 | 40,900 | 53,590 | 60,210 | 72,840 | 46,712 | 7,6954044 | |
| MEM_Perc_Nec_W3 | 27,470 | 26,890 | 18,690 | 21,840 | 34,890 | 17,630 | 24,568 | 2,6488091 | |
| MEM_GermCells_W3 | 0,198 | 0,538 | 1,542 | 0,225 | 0,048 | 0,141 | 0,448 | 0,2288393 | |
| MEM_Perc_ST_W4 | 34,130 | 28,820 | 27,670 | 16,960 | 34,890 | 20,440 | 27,152 | 2,9470182 | |
| MEM_Perc_Int_W4 | 41,480 | 50,810 | 55,940 | 65,490 | 55,830 | 67,640 | 56,198 | 3,9290062 | |
| MEM_Perc_Nec_W4 | 24,390 | 20,370 | 16,390 | 17,550 | 9,280 | 11,910 | 16,648 | 2,2465387 | |
| MEM_GermCells_W4 | 0,184 | 0,371 | 1,673 | 0,847 | 0,104 | 0,045 | 0,537 | 0,2561035 | |
| MEM_Perc_ST_W5 | 35,270 | 33,770 | 51,070 | 16,260 | 15,740 | 4,570 | 26,113 | 6,9140286 | |
| MEM_Perc_Int_W5 | 49,800 | 60,500 | 40,970 | 61,910 | 74,810 | 83,580 | 61,928 | 6,385193 | |
| MEM_Perc_Nec_W5 | 14,930 | 5,730 | 7,950 | 21,830 | 9,450 | 11,850 | 11,957 | 2,3625053 | |
| MEM_GermCells_W5 | 0,205 | 0,317 | 1,129 | 0,847 | 0,047 | 0,083 | 0,438 | 0,1818492 | |
| MEM_Perc_ST_W6 | 35,950 | 30,390 | 46,000 | 6,260 | 16,780 | 2,080 | 22,910 | 7,0894955 | |
| MEM_Perc_Int_W6 | 64,050 | 50,160 | 29,520 | 75,450 | 66,160 | 92,750 | 63,015 | 8,8259612 | |
| MEM_Perc_Nec_W6 | 0,000 | 19,450 | 24,480 | 18,290 | 17,070 | 5,160 | 14,075 | 3,8361167 | |
| MEM_GermCells_W6 | 0,033 | 0,101 | 0,498 | 0,510 | 0,000 | 0,000 | 0,190 | 0,1003932 | |
| KSR_Perc_ST_W2 | | 39,860 | 66,660 | 38,820 | 30,640 | 23,310 | 39,858 | 7,3424535 | |
| KSR_Perc_Int_W2 | | 48,520 | 29,380 | 45,690 | 54,940 | 53,490 | 46,404 | 4,5709874 | |
| KSR_Perc_Nec_W2 | | 11,620 | 3,950 | 15,490 | 14,430 | 23,190 | 13,736 | 3,1078893 | |
| KSR_GermCells_W2 | | 0,320 | 0,856 | 0,110 | 0,321 | 0,069 | 0,335 | 0,1402771 | |
| KSR_Perc_ST_W3 | 15,230 | 20,560 | 46,430 | 51,600 | 51,030 | 10,620 | 32,578 | 7,7925817 | |
| KSR_Perc_Int_W3 | 68,760 | 58,400 | 36,160 | 41,100 | 36,470 | 72,060 | 52,158 | 6,6708502 | |
| KSR_Perc_Nec_W3 | 16,010 | 21,030 | 17,410 | 7,300 | 12,510 | 17,320 | 15,263 | 1,9463259 | |
| KSR_GermCells_W3 | 0,174 | 0,115 | 0,649 | 0,068 | 0,000 | 0,031 | 0,173 | 0,09857 | |
| KSR_Perc_ST_W4 | 40,400 | 10,150 | 40,050 | | 26,130 | 6,900 | 24,726 | 7,1158152 | |
| KSR_Perc_Int_W4 | 52,010 | 63,750 | 46,320 | | 68,340 | 85,240 | 63,132 | 6,7919477 | |
| KSR_Perc_Nec_W4 | 7,590 | 26,100 | 13,630 | | 5,520 | 7,860 | 12,140 | 3,7413032 | |
| KSR_GermCells_W4 | 0,018 | 0,368 | 0,743 | | 0,060 | 0,000 | 0,238 | 0,142933 | |
| KSR_Perc_ST_W5 | 32,590 | 29,480 | 52,640 | 63,060 | 15,700 | 28,170 | 36,940 | 7,1446227 | |
| KSR_Perc_Int_W5 | 52,800 | 56,950 | 33,860 | 35,020 | 51,560 | 55,430 | 47,603 | 4,236732 | |
| KSR_Perc_Nec_W5 | 14,620 | 13,570 | 13,500 | 1,920 | 32,750 | 16,390 | 15,458 | 4,0466442 | |
| KSR_GermCells_W5 | 0,039 | 0,036 | 0,334 | 0,058 | 0,018 | 0,013 | 0,083 | 0,0506636 | |
| KSR_Perc_ST_W6 | 17,860 | 24,490 | 37,640 | 26,780 | 21,070 | 21,920 | 24,960 | 2,8231153 | |
| KSR_Perc_Int_W6 | 72,660 | 56,890 | 41,210 | 58,300 | 62,450 | 49,080 | 56,765 | 4,4285413 | |
| KSR_Perc_Nec_W6 | 9,480 | 18,620 | 21,150 | 14,920 | 16,480 | 29,000 | 18,275 | 2,678559 | |
| KSR_GermCells_W6 | 0,033 | 0,081 | 0,740 | 0,025 | 0,000 | 0,039 | 0,153 | 0,1179029 | |
| EST_Perc_ST_W2 | | | 46,190 | 23,290 | 8,620 | 24,770 | 25,718 | 7,7364843 | |
| EST_Perc_Int_W2 | | | 37,960 | 58,650 | 71,090 | 50,240 | 54,485 | 6,9771586 | |
| EST_Perc_Nec_W2 | | | 15,850 | 18,060 | 20,300 | 24,990 | 19,800 | 1,9539746 | |
| EST_GermCells_W2 | | | 1,820 | 0,801 | 0,286 | 0,257 | 0,791 | 0,3652255 | |
| EST_Perc_ST_W3 | 28,020 | 42,300 | 25,190 | 27,270 | 20,490 | 19,840 | 27,185 | 3,3262579 | |
| EST_Perc_Int_W3 | 45,720 | 29,910 | 59,700 | 54,290 | 77,860 | 50,860 | 53,057 | 6,4685922 | |
| EST_Perc_Nec_W3 | 26,260 | 27,790 | 15,110 | 18,440 | 1,660 | 29,300 | 19,760 | 4,2767488 | |
| EST_GermCells_W3 | 0,128 | 0,427 | 1,507 | 0,470 | 0,095 | 0,145 | 0,462 | 0,2190917 | |
| EST_Perc_ST_W4 | 40,870 | 14,590 | 42,230 | 10,510 | 44,770 | 17,040 | 28,335 | 6,4666817 | |
| EST_Perc_Int_W4 | 57,440 | 49,450 | 42,390 | 82,310 | 45,260 | 67,660 | 57,418 | 6,2226936 | |
| EST_Perc_Nec_W4 | 1,700 | 35,960 | 15,380 | 7,180 | 9,970 | 15,300 | 14,248 | 4,8287655 | |
| EST_GermCells_W4 | 0,195 | 0,486 | 1,495 | 0,634 | 0,048 | 0,059 | 0,486 | 0,2235198 | |
| EST_Perc_ST_W5 | 54,310 | 38,650 | 33,450 | 6,230 | 14,320 | 41,810 | 31,462 | 7,337651 | |
| EST_Perc_Int_W5 | 42,330 | 59,920 | 41,420 | 71,180 | 82,780 | 52,630 | 58,377 | 6,6830086 | |
| EST_Perc_Nec_W5 | 3,350 | 1,420 | 25,120 | 22,590 | 2,900 | 5,560 | 10,157 | 4,3777381 | |
| EST_GermCells_W5 | 0,147 | 0,508 | 1,222 | 1,893 | 0,000 | 0,643 | 0,736 | 0,2900794 | |
| EST_Perc_ST_W6 | 28,600 | 14,680 | 52,150 | 9,760 | 15,930 | 7,330 | 21,408 | 6,8457673 | |
| EST_Perc_Int_W6 | 70,910 | 71,050 | 27,560 | 68,320 | 77,420 | 76,500 | 65,293 | 7,6817896 | |
| EST_Perc_Nec_W6 | 0,480 | 14,270 | 20,290 | 21,920 | 6,650 | 16,170 | 13,297 | 3,3710391 | |
| EST_GermCells_W6 | 0,053 | 0,224 | 0,838 | 0,450 | 0,000 | 0,036 | 0,267 | 0,1330769 | |
| ALB_Perc_ST_W2 | | 23,690 | 44,010 | 27,210 | 49,610 | 57,160 | 40,336 | 6,4495485 | |
| ALB_Perc_Int_W2 | | 49,340 | 39,770 | 17,850 | 4,880 | 28,350 | 28,038 | 7,8472947 | |
| ALB_Perc_Nec_W2 | | 26,980 | 16,220 | 54,940 | 45,510 | 14,490 | 31,628 | 8,0277042 | |
| ALB_GermCells_W2 | | 0,727 | 1,558 | 0,510 | 0,481 | 0,244 | 0,704 | 0,2267844 | |
| ALB_Perc_ST_W3 | 13,200 | 13,450 | 24,320 | 14,540 | 6,340 | 38,580 | 18,405 | 4,6696336 | |
| ALB_Perc_Int_W3 | 59,540 | 76,610 | 52,110 | 37,720 | 36,120 | 18,430 | 46,755 | 8,3276514 | |
| ALB_Perc_Nec_W3 | 27,260 | 9,940 | 23,560 | 47,740 | 57,540 | 42,990 | 34,838 | 7,1945055 | |
| ALB_GermCells_W3 | 0,234 | 0,333 | 2,496 | 0,535 | 0,222 | 0,072 | 0,649 | 0,3747115 | |
| ALB_Perc_ST_W4 | 27,720 | 24,460 | 14,110 | 27,180 | 19,190 | 32,020 | 24,113 | 2,6416783 | |
| ALB_Perc_Int_W4 | 66,940 | 48,700 | 56,920 | 25,500 | 80,810 | 47,210 | 54,347 | 7,7038283 | |
| ALB_Perc_Nec_W4 | 5,350 | 26,840 | 28,970 | 47,320 | 0,000 | 20,770 | 21,542 | 7,0109055 | |

| | | | | | | | | |
|---|---|---|---|---|---|---|---|---|
| ALB_GermCells_W4 | 0,402 | 0,681 | 2,119 | 0,317 | 0,000 | 0,067 | 0,598 | 0,3203593 |
| ALB_Perc_ST_W5 | 45,580 | 23,630 | 34,550 | 32,140 | 26,960 | 0,000 | 27,143 | 6,2419915 |
| ALB_Perc_Int_W5 | 40,790 | 58,490 | 51,450 | 22,030 | 73,040 | 0,000 | 40,967 | 10,774294 |
| ALB_Perc_Nec_W5 | 13,640 | 17,880 | 14,000 | 45,830 | 0,000 | 100,000 | 31,892 | 14,943965 |
| ALB_GermCells_W5 | 0,017 | 0,415 | 0,000 | 0,051 | 0,000 | 0,000 | 0,081 | 0,0673141 |
| ALB_Perc_ST_W6 | 48,570 | 34,850 | 39,380 | 41,560 | 56,160 | 17,410 | 39,655 | 5,3960632 |
| ALB_Perc_Int_W6 | 40,870 | 52,740 | 42,840 | 41,850 | 25,620 | 53,490 | 42,902 | 4,1344048 |
| ALB_Perc_Nec_W6 | 10,560 | 12,410 | 17,780 | 16,590 | 18,230 | 29,100 | 17,445 | 2,6460848 |
| ALB_GermCells_W6 | 0,124 | 0,518 | 1,174 | 0,175 | 0,009 | 0,051 | 0,342 | 0,1820021 |
| ALB_EST_Perc_ST_W2 | | 24,270 | 31,460 | 10,910 | 32,840 | 52,520 | 30,400 | 6,7594697 |
| ALB_EST_Perc_Int_W2 | | 32,850 | 38,080 | 22,390 | 12,460 | 29,940 | 27,144 | 4,4620204 |
| ALB_EST_Perc_Nec_W2 | | 42,880 | 30,460 | 66,710 | 54,700 | 17,540 | 42,458 | 8,6690913 |
| ALB_EST_GermCells_W2 | | 0,728 | 2,257 | 0,370 | 0,447 | 0,095 | 0,779 | 0,3828888 |
| ALB_EST_Perc_ST_W3 | 35,870 | 22,090 | 23,980 | 32,680 | 53,850 | 31,500 | 33,328 | 4,6370015 |
| ALB_EST_Perc_Int_W3 | 46,430 | 49,200 | 50,240 | 35,990 | 46,150 | 32,470 | 43,413 | 3,0085486 |
| ALB_EST_Perc_Nec_W3 | 17,700 | 28,720 | 25,770 | 31,330 | 0,000 | 36,030 | 23,258 | 5,2796802 |
| ALB_EST_GermCells_W3 | 0,250 | 0,617 | 1,789 | 0,412 | 0,048 | 0,015 | 0,522 | 0,2697463 |
| ALB_EST_Perc_ST_W4 | 17,160 | 11,060 | 16,990 | 21,280 | 0,000 | 17,500 | 13,998 | 3,1027783 |
| ALB_EST_Perc_Int_W4 | 61,156 | 59,280 | 47,570 | 43,560 | 0,000 | 57,740 | 44,884 | 9,4177755 |
| ALB_EST_Perc_Nec_W4 | 21,684 | 29,660 | 35,450 | 35,170 | 100,000 | 24,750 | 41,119 | 11,988135 |
| ALB_EST_GermCells_W4 | 0,372 | 0,388 | 2,432 | 0,094 | 0,000 | 0,070 | 0,559 | 0,3802709 |
| ALB_EST_Perc_ST_W5 | 44,640 | 4,580 | 35,400 | 19,420 | 11,040 | 44,310 | 26,565 | 7,0594257 |
| ALB_EST_Perc_Int_W5 | 53,520 | 74,840 | 49,450 | 23,450 | 36,810 | 24,430 | 43,750 | 8,0167471 |
| ALB_EST_Perc_Nec_W5 | 1,840 | 20,580 | 15,150 | 57,130 | 52,150 | 31,260 | 29,685 | 8,812673 |
| ALB_EST_GermCells_W5 | 0,141 | 0,667 | 1,805 | 0,132 | 0,000 | 0,431 | 0,529 | 0,2735763 |
| ALB_EST_Perc_ST_W6 | 70,930 | 39,640 | 29,418 | 26,840 | 74,270 | 38,470 | 46,595 | 8,4809216 |
| ALB_EST_Perc_Int_W6 | 23,600 | 40,340 | 48,793 | 49,000 | 25,730 | 37,820 | 37,547 | 4,4709687 |
| ALB_EST_Perc_Nec_W6 | 5,470 | 20,020 | 21,788 | 24,160 | 0,000 | 23,710 | 15,858 | 4,2522703 |
| ALB_EST_GermCells_W6 | 0,057 | 0,616 | 1,151 | 0,176 | 0,008 | 0,123 | 0,355 | 0,1823168 |

```
 EXAMINE VARIABLES=Perc_ST_0 Perc_Int_0 GermCells_0 MEM_Perc_ST_W2 MEM_Perc_Int_W2 MEM_Perc_Nec
KSR_Perc_ST_W4 KSR_Perc_Int_W4 KSR_Perc_Nec_W4 KSR_GermCells_W4 KSR_Perc_ST_W5 KSR_Perc_Int_W5
ALB_Perc_ST_W2 ALB_Perc_Int_W2 ALB_Perc_Nec_W2 ALB_GermCells_W2 ALB_Perc_ST_W3 ALB_Perc_Int_W3
ALB_EST_Perc_Int_W4 ALB_EST_Perc_Nec_W4 ALB_EST_GermCells_W4 ALB_EST_Perc_ST_W5 ALB_EST_Perc_In
  /PLOT BOXPLOT NPPLOT
  /COMPARE GROUPS
  /STATISTICS DESCRIPTIVES
  /CINTERVAL 95
  /MISSING PAIRWISE
  /NOTOTAL.
```

## Explore

**Notes**

| | | |
|---|---|---|
| Output Created | | 10-AUG-2017 17:29:00 |
| Comments | | |
| Input | Data | /Users/pmota/Desktop/Organ Culture_treatment and ... |
| | Active Dataset | DataSet4 |
| | Filter | <none> |
| | Weight | <none> |
| | Split File | <none> |
| | N of Rows in Working Data File | 6 |
| Missing Value Handling | Definition of Missing | User-defined missing values for dependent variables are treated as missing. |
| | Cases Used | Statistics are based on cases with no missing values for the dependent variable or factor(s) being analyzed. |

**Notes**

| | |
|---|---|
| **Syntax** | EXAMINE<br>VARIABLES=Perc_ST_0<br>Perc_Int_0 GermCells_0<br>MEM_Perc_ST_W2<br>MEM_Perc_Int_W2<br>MEM_Perc_Nec_W2<br>MEM_GermCells_W2<br>MEM_Perc_ST_W3<br>MEM_Perc_Int_W3<br>MEM_Perc_Nec_W3<br>MEM_GermCells_W3<br>MEM_Perc_ST_W4<br>MEM_Perc_Int_W4<br>MEM_Perc_Nec_W4<br>MEM_GermCells_W4<br>MEM_Perc_ST_W5<br>MEM_Perc_Int_W5<br>MEM_Perc_Nec_W5<br>MEM_GermCells_W5<br>MEM_Perc_ST_W6<br>MEM_Perc_Int_W6<br>MEM_Perc_Nec_W6<br>MEM_GermCells_W6<br>KSR_Perc_ST_W2<br>KSR_Perc_Int_W2<br>KSR_Perc_Nec_W2<br>KSR_GermCells_W2<br>KSR_Perc_ST_W3<br>KSR_Perc_Int_W3<br>KSR_Perc_Nec_W3<br>KSR_GermCells_W3<br>KSR_Perc_ST_W4<br>KSR_Perc_Int_W4<br>KSR_Perc_Nec_W4<br>KSR_GermCells_W4<br>KSR_Perc_ST_W5<br>KSR_Perc_Int_W5<br>KSR_Perc_Nec_W5<br>KSR_GermCells_W5<br>KSR_Perc_ST_W6<br>KSR_Perc_Int_W6<br>KSR_Perc_Nec_W6<br>KSR_GermCells_W6<br>EST_Perc_ST_W2<br>EST_Perc_Int_W2<br>EST_Perc_Nec_W2<br>EST_GermCells_W2<br>EST_Perc_ST_W3<br>EST_Perc_Int_W3<br>EST_Perc_Nec_W3<br>EST_GermCells_W3<br>EST_Perc_ST_W4<br>EST_Perc_Int_W4<br>EST_Perc_Nec_W4<br>EST_GermCells_W4<br>EST_Perc_ST_W5<br>EST_Perc_Int_W5<br>EST_Perc_Nec_W5<br>EST_GermCells_W5<br>EST_Perc_ST_W6<br>EST_Perc_Int_W6<br>EST_Perc_Nec_W6<br>EST_GermCells_W6<br>ALB_Perc_ST_W2<br>ALB_Perc_Int_W2<br>ALB_Perc_Nec_W2<br>ALB_GermCells_W2<br>ALB_Perc_ST_W3<br>ALB_Perc_Int_W3<br>ALB_Perc_Nec_W3<br>ALB_GermCells_W3<br>ALB_Perc_ST_W4<br>ALB_Perc_Int_W4<br>ALB_Perc_Nec_W4<br>ALB_GermCells_W4<br>ALB_Perc_ST_W5<br>ALB_Perc_Int_W5 |

**Notes**

| | | |
|---|---|---|
| Resources | Processor Time | 00:00:45,63 |
| | Elapsed Time | 00:01:03,00 |

```
[DataSet4] /Users/pmota/Desktop/Organ Culture_treatment and week.sav
```

**Case Processing Summary**

| | Cases | | | | | |
|---|---|---|---|---|---|---|
| | Valid | | Missing | | Total | |
| | N | Percent | N | Percent | N | Percent |
| Perc_ST_0 | 6 | 100,0% | 0 | 0,0% | 6 | 100,0% |
| Perc_Int_0 | 6 | 100,0% | 0 | 0,0% | 6 | 100,0% |
| GermCells_0 | 6 | 100,0% | 0 | 0,0% | 6 | 100,0% |
| MEM_Perc_ST_W2 | 5 | 83,3% | 1 | 16,7% | 6 | 100,0% |
| MEM_Perc_Int_W2 | 5 | 83,3% | 1 | 16,7% | 6 | 100,0% |
| MEM_Perc_Nec_W2 | 5 | 83,3% | 1 | 16,7% | 6 | 100,0% |
| MEM_GermCells_W2 | 5 | 83,3% | 1 | 16,7% | 6 | 100,0% |
| MEM_Perc_ST_W3 | 6 | 100,0% | 0 | 0,0% | 6 | 100,0% |
| MEM_Perc_Int_W3 | 6 | 100,0% | 0 | 0,0% | 6 | 100,0% |
| MEM_Perc_Nec_W3 | 6 | 100,0% | 0 | 0,0% | 6 | 100,0% |
| MEM_GermCells_W3 | 6 | 100,0% | 0 | 0,0% | 6 | 100,0% |
| MEM_Perc_ST_W4 | 6 | 100,0% | 0 | 0,0% | 6 | 100,0% |
| MEM_Perc_Int_W4 | 6 | 100,0% | 0 | 0,0% | 6 | 100,0% |
| MEM_Perc_Nec_W4 | 6 | 100,0% | 0 | 0,0% | 6 | 100,0% |
| MEM_GermCells_W4 | 6 | 100,0% | 0 | 0,0% | 6 | 100,0% |
| MEM_Perc_ST_W5 | 6 | 100,0% | 0 | 0,0% | 6 | 100,0% |
| MEM_Perc_Int_W5 | 6 | 100,0% | 0 | 0,0% | 6 | 100,0% |
| MEM_Perc_Nec_W5 | 6 | 100,0% | 0 | 0,0% | 6 | 100,0% |
| MEM_GermCells_W5 | 6 | 100,0% | 0 | 0,0% | 6 | 100,0% |
| MEM_Perc_ST_W6 | 6 | 100,0% | 0 | 0,0% | 6 | 100,0% |
| MEM_Perc_Int_W6 | 6 | 100,0% | 0 | 0,0% | 6 | 100,0% |
| MEM_Perc_Nec_W6 | 6 | 100,0% | 0 | 0,0% | 6 | 100,0% |
| MEM_GermCells_W6 | 6 | 100,0% | 0 | 0,0% | 6 | 100,0% |
| KSR_Perc_ST_W2 | 5 | 83,3% | 1 | 16,7% | 6 | 100,0% |
| KSR_Perc_Int_W2 | 5 | 83,3% | 1 | 16,7% | 6 | 100,0% |
| KSR_Perc_Nec_W2 | 5 | 83,3% | 1 | 16,7% | 6 | 100,0% |
| KSR_GermCells_W2 | 5 | 83,3% | 1 | 16,7% | 6 | 100,0% |
| KSR_Perc_ST_W3 | 6 | 100,0% | 0 | 0,0% | 6 | 100,0% |
| KSR_Perc_Int_W3 | 6 | 100,0% | 0 | 0,0% | 6 | 100,0% |
| KSR_Perc_Nec_W3 | 6 | 100,0% | 0 | 0,0% | 6 | 100,0% |
| KSR_GermCells_W3 | 6 | 100,0% | 0 | 0,0% | 6 | 100,0% |
| KSR_Perc_ST_W4 | 5 | 83,3% | 1 | 16,7% | 6 | 100,0% |
| KSR_Perc_Int_W4 | 5 | 83,3% | 1 | 16,7% | 6 | 100,0% |
| KSR_Perc_Nec_W4 | 5 | 83,3% | 1 | 16,7% | 6 | 100,0% |
| KSR_GermCells_W4 | 5 | 83,3% | 1 | 16,7% | 6 | 100,0% |
| KSR_Perc_ST_W5 | 6 | 100,0% | 0 | 0,0% | 6 | 100,0% |
| KSR_Perc_Int_W5 | 6 | 100,0% | 0 | 0,0% | 6 | 100,0% |
| KSR_Perc_Nec_W5 | 6 | 100,0% | 0 | 0,0% | 6 | 100,0% |
| KSR_GermCells_W5 | 6 | 100,0% | 0 | 0,0% | 6 | 100,0% |
| KSR_Perc_ST_W6 | 6 | 100,0% | 0 | 0,0% | 6 | 100,0% |
| KSR_Perc_Int_W6 | 6 | 100,0% | 0 | 0,0% | 6 | 100,0% |
| KSR_Perc_Nec_W6 | 6 | 100,0% | 0 | 0,0% | 6 | 100,0% |
| KSR_GermCells_W6 | 6 | 100,0% | 0 | 0,0% | 6 | 100,0% |

Case Processing Summary

| | Cases | | | | | |
|---|---|---|---|---|---|---|
| | Valid | | Missing | | Total | |
| | N | Percent | N | Percent | N | Percent |
| EST_Perc_ST_W2 | 4 | 66,7% | 2 | 33,3% | 6 | 100,0% |
| EST_Perc_Int_W2 | 4 | 66,7% | 2 | 33,3% | 6 | 100,0% |
| EST_Perc_Nec_W2 | 4 | 66,7% | 2 | 33,3% | 6 | 100,0% |
| EST_GermCells_W2 | 4 | 66,7% | 2 | 33,3% | 6 | 100,0% |
| EST_Perc_ST_W3 | 6 | 100,0% | 0 | 0,0% | 6 | 100,0% |
| EST_Perc_Int_W3 | 6 | 100,0% | 0 | 0,0% | 6 | 100,0% |
| EST_Perc_Nec_W3 | 6 | 100,0% | 0 | 0,0% | 6 | 100,0% |
| EST_GermCells_W3 | 6 | 100,0% | 0 | 0,0% | 6 | 100,0% |
| EST_Perc_ST_W4 | 6 | 100,0% | 0 | 0,0% | 6 | 100,0% |
| EST_Perc_Int_W4 | 6 | 100,0% | 0 | 0,0% | 6 | 100,0% |
| EST_Perc_Nec_W4 | 6 | 100,0% | 0 | 0,0% | 6 | 100,0% |
| EST_GermCells_W4 | 6 | 100,0% | 0 | 0,0% | 6 | 100,0% |
| EST_Perc_ST_W5 | 6 | 100,0% | 0 | 0,0% | 6 | 100,0% |
| EST_Perc_Int_W5 | 6 | 100,0% | 0 | 0,0% | 6 | 100,0% |
| EST_Perc_Nec_W5 | 6 | 100,0% | 0 | 0,0% | 6 | 100,0% |
| EST_GermCells_W5 | 6 | 100,0% | 0 | 0,0% | 6 | 100,0% |
| EST_Perc_ST_W6 | 6 | 100,0% | 0 | 0,0% | 6 | 100,0% |
| EST_Perc_Int_W6 | 6 | 100,0% | 0 | 0,0% | 6 | 100,0% |
| EST_Perc_Nec_W6 | 6 | 100,0% | 0 | 0,0% | 6 | 100,0% |
| EST_GermCells_W6 | 6 | 100,0% | 0 | 0,0% | 6 | 100,0% |
| ALB_Perc_ST_W2 | 5 | 83,3% | 1 | 16,7% | 6 | 100,0% |
| ALB_Perc_Int_W2 | 5 | 83,3% | 1 | 16,7% | 6 | 100,0% |
| ALB_Perc_Nec_W2 | 5 | 83,3% | 1 | 16,7% | 6 | 100,0% |
| ALB_GermCells_W2 | 5 | 83,3% | 1 | 16,7% | 6 | 100,0% |
| ALB_Perc_ST_W3 | 6 | 100,0% | 0 | 0,0% | 6 | 100,0% |
| ALB_Perc_Int_W3 | 6 | 100,0% | 0 | 0,0% | 6 | 100,0% |
| ALB_Perc_Nec_W3 | 6 | 100,0% | 0 | 0,0% | 6 | 100,0% |
| ALB_GermCells_W3 | 6 | 100,0% | 0 | 0,0% | 6 | 100,0% |
| ALB_Perc_ST_W4 | 6 | 100,0% | 0 | 0,0% | 6 | 100,0% |
| ALB_Perc_Int_W4 | 6 | 100,0% | 0 | 0,0% | 6 | 100,0% |
| ALB_Perc_Nec_W4 | 6 | 100,0% | 0 | 0,0% | 6 | 100,0% |
| ALB_GermCells_W4 | 6 | 100,0% | 0 | 0,0% | 6 | 100,0% |
| ALB_Perc_ST_W5 | 6 | 100,0% | 0 | 0,0% | 6 | 100,0% |
| ALB_Perc_Int_W5 | 6 | 100,0% | 0 | 0,0% | 6 | 100,0% |
| ALB_Perc_Nec_W5 | 6 | 100,0% | 0 | 0,0% | 6 | 100,0% |
| ALB_GermCells_W5 | 6 | 100,0% | 0 | 0,0% | 6 | 100,0% |
| ALB_Perc_ST_W6 | 6 | 100,0% | 0 | 0,0% | 6 | 100,0% |
| ALB_Perc_Int_W6 | 6 | 100,0% | 0 | 0,0% | 6 | 100,0% |
| ALB_Perc_Nec_W6 | 6 | 100,0% | 0 | 0,0% | 6 | 100,0% |
| ALB_GermCells_W6 | 6 | 100,0% | 0 | 0,0% | 6 | 100,0% |
| ALB_EST_Perc_ST_W2 | 5 | 83,3% | 1 | 16,7% | 6 | 100,0% |
| ALB_EST_Perc_Int_W2 | 5 | 83,3% | 1 | 16,7% | 6 | 100,0% |
| ALB_EST_Perc_Nec_W2 | 5 | 83,3% | 1 | 16,7% | 6 | 100,0% |
| ALB_EST_GermCells_W2 | 5 | 83,3% | 1 | 16,7% | 6 | 100,0% |
| ALB_EST_Perc_ST_W3 | 6 | 100,0% | 0 | 0,0% | 6 | 100,0% |
| ALB_EST_Perc_Int_W3 | 6 | 100,0% | 0 | 0,0% | 6 | 100,0% |
| ALB_EST_Perc_Nec_W3 | 6 | 100,0% | 0 | 0,0% | 6 | 100,0% |
| ALB_EST_GermCells_W3 | 6 | 100,0% | 0 | 0,0% | 6 | 100,0% |

**Case Processing Summary**

| | Cases | | | | | |
|---|---|---|---|---|---|---|
| | Valid | | Missing | | Total | |
| | N | Percent | N | Percent | N | Percent |
| ALB_EST_Perc_ST_W4 | 6 | 100,0% | 0 | 0,0% | 6 | 100,0% |
| ALB_EST_Perc_Int_W4 | 6 | 100,0% | 0 | 0,0% | 6 | 100,0% |
| ALB_EST_Perc_Nec_W4 | 6 | 100,0% | 0 | 0,0% | 6 | 100,0% |
| ALB_EST_GermCells_W4 | 6 | 100,0% | 0 | 0,0% | 6 | 100,0% |
| ALB_EST_Perc_ST_W5 | 6 | 100,0% | 0 | 0,0% | 6 | 100,0% |
| ALB_EST_Perc_Int_W5 | 6 | 100,0% | 0 | 0,0% | 6 | 100,0% |
| ALB_EST_Perc_Nec_W5 | 6 | 100,0% | 0 | 0,0% | 6 | 100,0% |
| ALB_EST_GermCells_W5 | 6 | 100,0% | 0 | 0,0% | 6 | 100,0% |
| ALB_EST_Perc_ST_W6 | 6 | 100,0% | 0 | 0,0% | 6 | 100,0% |
| ALB_EST_Perc_Int_W6 | 6 | 100,0% | 0 | 0,0% | 6 | 100,0% |
| ALB_EST_Perc_Nec_W6 | 6 | 100,0% | 0 | 0,0% | 6 | 100,0% |
| ALB_EST_GermCells_W6 | 6 | 100,0% | 0 | 0,0% | 6 | 100,0% |

**Descriptives**

| | | | Statistic | Std. Error |
|---|---|---|---|---|
| Perc_ST_0 | Mean | | 76,0048 | 1,23880 |
| | 95% Confidence Interval for Mean | Lower Bound | 72,8204 | |
| | | Upper Bound | 79,1892 | |
| | 5% Trimmed Mean | | 75,8979 | |
| | Median | | 75,5140 | |
| | Variance | | 9,208 | |
| | Std. Deviation | | 3,03444 | |
| | Minimum | | 72,95 | |
| | Maximum | | 80,99 | |
| | Range | | 8,04 | |
| | Interquartile Range | | 5,51 | |
| | Skewness | | ,850 | ,845 |
| | Kurtosis | | ,173 | 1,741 |
| Perc_Int_0 | Mean | | 23,9952 | 1,23880 |
| | 95% Confidence Interval for Mean | Lower Bound | 20,8108 | |
| | | Upper Bound | 27,1796 | |
| | 5% Trimmed Mean | | 24,1021 | |
| | Median | | 24,4860 | |
| | Variance | | 9,208 | |
| | Std. Deviation | | 3,03444 | |
| | Minimum | | 19,01 | |
| | Maximum | | 27,05 | |
| | Range | | 8,04 | |
| | Interquartile Range | | 5,51 | |
| | Skewness | | -,850 | ,845 |
| | Kurtosis | | ,173 | 1,741 |
| GermCells_0 | Mean | | ,7685 | ,11492 |
| | 95% Confidence Interval for Mean | Lower Bound | ,4731 | |
| | | Upper Bound | 1,0639 | |
| | 5% Trimmed Mean | | ,7496 | |
| | Median | | ,6547 | |
| | Variance | | ,079 | |
| | Std. Deviation | | ,28149 | |
| | Minimum | | ,56 | |

**Descriptives**

| | | | Statistic | Std. Error |
|---|---|---|---|---|
| MEM_Perc_ST_W2 | Maximum | | 1,32 | |
| | Range | | ,76 | |
| | Interquartile Range | | ,32 | |
| | Skewness | | 2,038 | ,845 |
| | Kurtosis | | 4,326 | 1,741 |
| | Mean | | 35,0780 | 7,96528 |
| | 95% Confidence Interval for Mean | Lower Bound | 12,9628 | |
| | | Upper Bound | 57,1932 | |
| | 5% Trimmed Mean | | 34,8917 | |
| | Median | | 29,4800 | |
| | Variance | | 317,228 | |
| | Std. Deviation | | 17,81090 | |
| | Minimum | | 14,29 | |
| | Maximum | | 59,22 | |
| | Range | | 44,93 | |
| | Interquartile Range | | 32,96 | |
| | Skewness | | ,417 | ,913 |
| | Kurtosis | | -1,127 | 2,000 |
| MEM_Perc_Int_W2 | Mean | | 46,8480 | 5,46346 |
| | 95% Confidence Interval for Mean | Lower Bound | 31,6790 | |
| | | Upper Bound | 62,0170 | |
| | 5% Trimmed Mean | | 47,1094 | |
| | Median | | 44,6500 | |
| | Variance | | 149,247 | |
| | Std. Deviation | | 12,21666 | |
| | Minimum | | 29,45 | |
| | Maximum | | 59,54 | |
| | Range | | 30,09 | |
| | Interquartile Range | | 22,28 | |
| | Skewness | | -,472 | ,913 |
| | Kurtosis | | -,659 | 2,000 |
| MEM_Perc_Nec_W2 | Mean | | 18,0760 | 4,09510 |
| | 95% Confidence Interval for Mean | Lower Bound | 6,7062 | |
| | | Upper Bound | 29,4458 | |
| | 5% Trimmed Mean | | 18,0372 | |
| | Median | | 14,7500 | |
| | Variance | | 83,849 | |
| | Std. Deviation | | 9,15692 | |
| | Minimum | | 8,66 | |
| | Maximum | | 28,19 | |
| | Range | | 19,53 | |
| | Interquartile Range | | 17,83 | |
| | Skewness | | ,369 | ,913 |
| | Kurtosis | | -2,979 | 2,000 |
| MEM_GermCells_W2 | Mean | | ,5971 | ,23160 |
| | 95% Confidence Interval for Mean | Lower Bound | -,0460 | |
| | | Upper Bound | 1,2401 | |
| | 5% Trimmed Mean | | ,5759 | |
| | Median | | ,3827 | |
| | Variance | | ,268 | |
| | Std. Deviation | | ,51787 | |

## Descriptives

| | | | Statistic | Std. Error |
|---|---|---|---|---|
| | Minimum | | ,12 | |
| | Maximum | | 1,45 | |
| | Range | | 1,33 | |
| | Interquartile Range | | ,83 | |
| | Skewness | | 1,487 | ,913 |
| | Kurtosis | | 2,319 | 2,000 |
| MEM_Perc_ST_W3 | Mean | | 25,7067 | 6,78480 |
| | 95% Confidence Interval for Mean | Lower Bound | 8,2658 | |
| | | Upper Bound | 43,1475 | |
| | 5% Trimmed Mean | | 25,6619 | |
| | Median | | 26,0400 | |
| | Variance | | 276,201 | |
| | Std. Deviation | | 16,61929 | |
| | Minimum | | 4,91 | |
| | Maximum | | 47,31 | |
| | Range | | 42,40 | |
| | Interquartile Range | | 33,75 | |
| | Skewness | | ,003 | ,845 |
| | Kurtosis | | -1,480 | 1,741 |
| MEM_Perc_Int_W3 | Mean | | 46,7117 | 7,69540 |
| | 95% Confidence Interval for Mean | Lower Bound | 26,9300 | |
| | | Upper Bound | 66,4933 | |
| | 5% Trimmed Mean | | 46,4541 | |
| | Median | | 47,2450 | |
| | Variance | | 355,315 | |
| | Std. Deviation | | 18,84981 | |
| | Minimum | | 25,22 | |
| | Maximum | | 72,84 | |
| | Range | | 47,62 | |
| | Interquartile Range | | 36,43 | |
| | Skewness | | ,153 | ,845 |
| | Kurtosis | | -1,526 | 1,741 |
| MEM_Perc_Nec_W3 | Mean | | 24,5683 | 2,64881 |
| | 95% Confidence Interval for Mean | Lower Bound | 17,7594 | |
| | | Upper Bound | 31,3773 | |
| | 5% Trimmed Mean | | 24,3804 | |
| | Median | | 24,3650 | |
| | Variance | | 42,097 | |
| | Std. Deviation | | 6,48823 | |
| | Minimum | | 17,63 | |
| | Maximum | | 34,89 | |
| | Range | | 17,26 | |
| | Interquartile Range | | 10,90 | |
| | Skewness | | ,636 | ,845 |
| | Kurtosis | | -,318 | 1,741 |
| MEM_GermCells_W3 | Mean | | ,4484 | ,22884 |
| | 95% Confidence Interval for Mean | Lower Bound | -,1398 | |
| | | Upper Bound | 1,0367 | |
| | 5% Trimmed Mean | | ,4100 | |
| | Median | | ,2113 | |
| | Variance | | ,314 | |

## Descriptives

| | | | Statistic | Std. Error |
|---|---|---|---|---|
| | Std. Deviation | | ,56054 | |
| | Minimum | | ,05 | |
| | Maximum | | 1,54 | |
| | Range | | 1,49 | |
| | Interquartile Range | | ,67 | |
| | Skewness | | 2,021 | ,845 |
| | Kurtosis | | 4,168 | 1,741 |
| MEM_Perc_ST_W4 | Mean | | 27,1517 | 2,94702 |
| | 95% Confidence Interval for Mean | Lower Bound | 19,5761 | |
| | | Upper Bound | 34,7272 | |
| | 5% Trimmed Mean | | 27,2880 | |
| | Median | | 28,2450 | |
| | Variance | | 52,109 | |
| | Std. Deviation | | 7,21869 | |
| | Minimum | | 16,96 | |
| | Maximum | | 34,89 | |
| | Range | | 17,93 | |
| | Interquartile Range | | 14,75 | |
| | Skewness | | -,441 | ,845 |
| | Kurtosis | | -1,408 | 1,741 |
| MEM_Perc_Int_W4 | Mean | | 56,1983 | 3,92901 |
| | 95% Confidence Interval for Mean | Lower Bound | 46,0985 | |
| | | Upper Bound | 66,2982 | |
| | 5% Trimmed Mean | | 56,3804 | |
| | Median | | 55,8850 | |
| | Variance | | 92,623 | |
| | Std. Deviation | | 9,62406 | |
| | Minimum | | 41,48 | |
| | Maximum | | 67,64 | |
| | Range | | 26,16 | |
| | Interquartile Range | | 17,55 | |
| | Skewness | | -,352 | ,845 |
| | Kurtosis | | -,346 | 1,741 |
| MEM_Perc_Nec_W4 | Mean | | 16,6483 | 2,24654 |
| | 95% Confidence Interval for Mean | Lower Bound | 10,8734 | |
| | | Upper Bound | 22,4232 | |
| | 5% Trimmed Mean | | 16,6276 | |
| | Median | | 16,9700 | |
| | Variance | | 30,282 | |
| | Std. Deviation | | 5,50287 | |
| | Minimum | | 9,28 | |
| | Maximum | | 24,39 | |
| | Range | | 15,11 | |
| | Interquartile Range | | 10,12 | |
| | Skewness | | ,018 | ,845 |
| | Kurtosis | | -,726 | 1,741 |
| MEM_GermCells_W4 | Mean | | ,5374 | ,25610 |
| | 95% Confidence Interval for Mean | Lower Bound | -,1209 | |
| | | Upper Bound | 1,1958 | |
| | 5% Trimmed Mean | | ,5017 | |
| | Median | | ,2775 | |

**Descriptives**

| | | | Statistic | Std. Error |
|---|---|---|---|---|
| | Variance | | ,394 | |
| | Std. Deviation | | ,62732 | |
| | Minimum | | ,05 | |
| | Maximum | | 1,67 | |
| | Range | | 1,63 | |
| | Interquartile Range | | ,96 | |
| | Skewness | | 1,513 | ,845 |
| | Kurtosis | | 1,806 | 1,741 |
| MEM_Perc_ST_W5 | Mean | | 26,1133 | 6,91403 |
| | 95% Confidence Interval for Mean | Lower Bound | 8,3403 | |
| | | Upper Bound | 43,8864 | |
| | 5% Trimmed Mean | | 25,9237 | |
| | Median | | 25,0150 | |
| | Variance | | 286,823 | |
| | Std. Deviation | | 16,93584 | |
| | Minimum | | 4,57 | |
| | Maximum | | 51,07 | |
| | Range | | 46,50 | |
| | Interquartile Range | | 26,27 | |
| | Skewness | | ,290 | ,845 |
| | Kurtosis | | -,849 | 1,741 |
| MEM_Perc_Int_W5 | Mean | | 61,9283 | 6,38519 |
| | 95% Confidence Interval for Mean | Lower Bound | 45,5147 | |
| | | Upper Bound | 78,3420 | |
| | 5% Trimmed Mean | | 61,8898 | |
| | Median | | 61,2050 | |
| | Variance | | 244,624 | |
| | Std. Deviation | | 15,64046 | |
| | Minimum | | 40,97 | |
| | Maximum | | 83,58 | |
| | Range | | 42,61 | |
| | Interquartile Range | | 29,41 | |
| | Skewness | | ,102 | ,845 |
| | Kurtosis | | -,847 | 1,741 |
| MEM_Perc_Nec_W5 | Mean | | 11,9567 | 2,36251 |
| | 95% Confidence Interval for Mean | Lower Bound | 5,8837 | |
| | | Upper Bound | 18,0297 | |
| | 5% Trimmed Mean | | 11,7541 | |
| | Median | | 10,6500 | |
| | Variance | | 33,489 | |
| | Std. Deviation | | 5,78693 | |
| | Minimum | | 5,73 | |
| | Maximum | | 21,83 | |
| | Range | | 16,10 | |
| | Interquartile Range | | 9,26 | |
| | Skewness | | 1,033 | ,845 |
| | Kurtosis | | ,854 | 1,741 |
| MEM_GermCells_W5 | Mean | | ,4380 | ,18185 |
| | 95% Confidence Interval for Mean | Lower Bound | -,0295 | |
| | | Upper Bound | ,9054 | |
| | 5% Trimmed Mean | | ,4213 | |

## Descriptives

| | | | Statistic | Std. Error |
|---|---|---|---|---|
| | Median | | ,2609 | |
| | Variance | | ,198 | |
| | Std. Deviation | | ,44544 | |
| | Minimum | | ,05 | |
| | Maximum | | 1,13 | |
| | Range | | 1,08 | |
| | Interquartile Range | | ,84 | |
| | Skewness | | ,949 | ,845 |
| | Kurtosis | | -,946 | 1,741 |
| MEM_Perc_ST_W6 | Mean | | 22,9100 | 7,08950 |
| | 95% Confidence Interval for Mean | Lower Bound | 4,6859 | |
| | | Upper Bound | 41,1341 | |
| | 5% Trimmed Mean | | 22,7844 | |
| | Median | | 23,5850 | |
| | Variance | | 301,566 | |
| | Std. Deviation | | 17,36565 | |
| | Minimum | | 2,08 | |
| | Maximum | | 46,00 | |
| | Range | | 43,92 | |
| | Interquartile Range | | 33,25 | |
| | Skewness | | ,061 | ,845 |
| | Kurtosis | | -1,764 | 1,741 |
| MEM_Perc_Int_W6 | Mean | | 63,0150 | 8,82596 |
| | 95% Confidence Interval for Mean | Lower Bound | 40,3271 | |
| | | Upper Bound | 85,7029 | |
| | 5% Trimmed Mean | | 63,2239 | |
| | Median | | 65,1050 | |
| | Variance | | 467,386 | |
| | Std. Deviation | | 21,61910 | |
| | Minimum | | 29,52 | |
| | Maximum | | 92,75 | |
| | Range | | 63,23 | |
| | Interquartile Range | | 34,78 | |
| | Skewness | | -,340 | ,845 |
| | Kurtosis | | ,453 | 1,741 |
| MEM_Perc_Nec_W6 | Mean | | 14,0750 | 3,83612 |
| | 95% Confidence Interval for Mean | Lower Bound | 4,2139 | |
| | | Upper Bound | 23,9361 | |
| | 5% Trimmed Mean | | 14,2789 | |
| | Median | | 17,6800 | |
| | Variance | | 88,295 | |
| | Std. Deviation | | 9,39653 | |
| | Minimum | | ,00 | |
| | Maximum | | 24,48 | |
| | Range | | 24,48 | |
| | Interquartile Range | | 16,84 | |
| | Skewness | | -,764 | ,845 |
| | Kurtosis | | -,996 | 1,741 |
| MEM_GermCells_W6 | Mean | | ,1904 | ,10039 |
| | 95% Confidence Interval for Mean | Lower Bound | -,0677 | |
| | | Upper Bound | ,4485 | |

## Descriptives

| | | | Statistic | Std. Error |
|---|---|---|---|---|
| | 5% Trimmed Mean | | ,1832 | |
| | Median | | ,0670 | |
| | Variance | | ,060 | |
| | Std. Deviation | | ,24591 | |
| | Minimum | | ,00 | |
| | Maximum | | ,51 | |
| | Range | | ,51 | |
| | Interquartile Range | | ,50 | |
| | Skewness | | ,877 | ,845 |
| | Kurtosis | | -1,895 | 1,741 |
| KSR_Perc_ST_W2 | Mean | | 39,8580 | 7,34245 |
| | 95% Confidence Interval for Mean | Lower Bound | 19,4721 | |
| | | Upper Bound | 60,2439 | |
| | 5% Trimmed Mean | | 39,2883 | |
| | Median | | 38,8200 | |
| | Variance | | 269,558 | |
| | Std. Deviation | | 16,41823 | |
| | Minimum | | 23,31 | |
| | Maximum | | 66,66 | |
| | Range | | 43,35 | |
| | Interquartile Range | | 26,29 | |
| | Skewness | | 1,312 | ,913 |
| | Kurtosis | | 2,291 | 2,000 |
| KSR_Perc_Int_W2 | Mean | | 46,4040 | 4,57099 |
| | 95% Confidence Interval for Mean | Lower Bound | 33,7129 | |
| | | Upper Bound | 59,0951 | |
| | 5% Trimmed Mean | | 46,8756 | |
| | Median | | 48,5200 | |
| | Variance | | 104,470 | |
| | Std. Deviation | | 10,22104 | |
| | Minimum | | 29,38 | |
| | Maximum | | 54,94 | |
| | Range | | 25,56 | |
| | Interquartile Range | | 16,68 | |
| | Skewness | | -1,540 | ,913 |
| | Kurtosis | | 2,519 | 2,000 |
| KSR_Perc_Nec_W2 | Mean | | 13,7360 | 3,10789 |
| | 95% Confidence Interval for Mean | Lower Bound | 5,1071 | |
| | | Upper Bound | 22,3649 | |
| | 5% Trimmed Mean | | 13,7544 | |
| | Median | | 14,4300 | |
| | Variance | | 48,295 | |
| | Std. Deviation | | 6,94945 | |
| | Minimum | | 3,95 | |
| | Maximum | | 23,19 | |
| | Range | | 19,24 | |
| | Interquartile Range | | 11,56 | |
| | Skewness | | -,119 | ,913 |
| | Kurtosis | | 1,212 | 2,000 |
| KSR_GermCells_W2 | Mean | | ,3351 | ,14028 |

**Descriptives**

| | | | Statistic | Std. Error |
|---|---|---|---|---|
| | 95% Confidence Interval for Mean | Lower Bound | -,0544 | |
| | | Upper Bound | ,7246 | |
| | 5% Trimmed Mean | | ,3210 | |
| | Median | | ,3196 | |
| | Variance | | ,098 | |
| | Std. Deviation | | ,31367 | |
| | Minimum | | ,07 | |
| | Maximum | | ,86 | |
| | Range | | ,79 | |
| | Interquartile Range | | ,50 | |
| | Skewness | | 1,501 | ,913 |
| | Kurtosis | | 2,501 | 2,000 |
| KSR_Perc_ST_W3 | Mean | | 32,5783 | 7,79258 |
| | 95% Confidence Interval for Mean | Lower Bound | 12,5469 | |
| | | Upper Bound | 52,6098 | |
| | 5% Trimmed Mean | | 32,7415 | |
| | Median | | 33,4950 | |
| | Variance | | 364,346 | |
| | Std. Deviation | | 19,08785 | |
| | Minimum | | 10,62 | |
| | Maximum | | 51,60 | |
| | Range | | 40,98 | |
| | Interquartile Range | | 37,10 | |
| | Skewness | | -,074 | ,845 |
| | Kurtosis | | -2,941 | 1,741 |
| KSR_Perc_Int_W3 | Mean | | 52,1583 | 6,67085 |
| | 95% Confidence Interval for Mean | Lower Bound | 35,0104 | |
| | | Upper Bound | 69,3063 | |
| | 5% Trimmed Mean | | 51,9415 | |
| | Median | | 49,7500 | |
| | Variance | | 267,001 | |
| | Std. Deviation | | 16,34018 | |
| | Minimum | | 36,16 | |
| | Maximum | | 72,06 | |
| | Range | | 35,90 | |
| | Interquartile Range | | 33,19 | |
| | Skewness | | ,233 | ,845 |
| | Kurtosis | | -2,564 | 1,741 |
| KSR_Perc_Nec_W3 | Mean | | 15,2633 | 1,94633 |
| | 95% Confidence Interval for Mean | Lower Bound | 10,2601 | |
| | | Upper Bound | 20,2665 | |
| | 5% Trimmed Mean | | 15,3854 | |
| | Median | | 16,6650 | |
| | Variance | | 22,729 | |
| | Std. Deviation | | 4,76751 | |
| | Minimum | | 7,30 | |
| | Maximum | | 21,03 | |
| | Range | | 13,73 | |
| | Interquartile Range | | 7,11 | |
| | Skewness | | -,872 | ,845 |
| | Kurtosis | | ,829 | 1,741 |

**Descriptives**

| | | | Statistic | Std. Error |
|---|---|---|---|---|
| KSR_GermCells_W3 | Mean | | ,1728 | ,09857 |
| | 95% Confidence Interval for Mean | Lower Bound | -,0805 | |
| | | Upper Bound | ,4262 | |
| | 5% Trimmed Mean | | ,1560 | |
| | Median | | ,0912 | |
| | Variance | | ,058 | |
| | Std. Deviation | | ,24145 | |
| | Minimum | | ,00 | |
| | Maximum | | ,65 | |
| | Range | | ,65 | |
| | Interquartile Range | | ,27 | |
| | Skewness | | 2,105 | ,845 |
| | Kurtosis | | 4,661 | 1,741 |
| KSR_Perc_ST_W4 | Mean | | 24,7260 | 7,11582 |
| | 95% Confidence Interval for Mean | Lower Bound | 4,9693 | |
| | | Upper Bound | 44,4827 | |
| | 5% Trimmed Mean | | 24,8456 | |
| | Median | | 26,1300 | |
| | Variance | | 253,174 | |
| | Std. Deviation | | 15,91145 | |
| | Minimum | | 6,90 | |
| | Maximum | | 40,40 | |
| | Range | | 33,50 | |
| | Interquartile Range | | 31,70 | |
| | Skewness | | -,135 | ,913 |
| | Kurtosis | | -2,898 | 2,000 |
| KSR_Perc_Int_W4 | Mean | | 63,1320 | 6,79195 |
| | 95% Confidence Interval for Mean | Lower Bound | 44,2745 | |
| | | Upper Bound | 81,9895 | |
| | 5% Trimmed Mean | | 62,8378 | |
| | Median | | 63,7500 | |
| | Variance | | 230,653 | |
| | Std. Deviation | | 15,18726 | |
| | Minimum | | 46,32 | |
| | Maximum | | 85,24 | |
| | Range | | 38,92 | |
| | Interquartile Range | | 27,63 | |
| | Skewness | | ,573 | ,913 |
| | Kurtosis | | -,133 | 2,000 |
| KSR_Perc_Nec_W4 | Mean | | 12,1400 | 3,74130 |
| | 95% Confidence Interval for Mean | Lower Bound | 1,7525 | |
| | | Upper Bound | 22,5275 | |
| | 5% Trimmed Mean | | 11,7322 | |
| | Median | | 7,8600 | |
| | Variance | | 69,987 | |
| | Std. Deviation | | 8,36581 | |
| | Minimum | | 5,52 | |
| | Maximum | | 26,10 | |
| | Range | | 20,58 | |
| | Interquartile Range | | 13,31 | |
| | Skewness | | 1,609 | ,913 |

### Descriptives

| | | | Statistic | Std. Error |
|---|---|---|---|---|
| | Kurtosis | | 2,379 | 2,000 |
| KSR_GermCells_W4 | Mean | | ,2376 | ,14293 |
| | 95% Confidence Interval for Mean | Lower Bound | -,1592 | |
| | | Upper Bound | ,6345 | |
| | 5% Trimmed Mean | | ,2228 | |
| | Median | | ,0602 | |
| | Variance | | ,102 | |
| | Std. Deviation | | ,31961 | |
| | Minimum | | ,00 | |
| | Maximum | | ,74 | |
| | Range | | ,74 | |
| | Interquartile Range | | ,55 | |
| | Skewness | | 1,295 | ,913 |
| | Kurtosis | | ,624 | 2,000 |
| KSR_Perc_ST_W5 | Mean | | 36,9400 | 7,14462 |
| | 95% Confidence Interval for Mean | Lower Bound | 18,5742 | |
| | | Upper Bound | 55,3058 | |
| | 5% Trimmed Mean | | 36,6689 | |
| | Median | | 31,0350 | |
| | Variance | | 306,274 | |
| | Std. Deviation | | 17,50068 | |
| | Minimum | | 15,70 | |
| | Maximum | | 63,06 | |
| | Range | | 47,36 | |
| | Interquartile Range | | 30,19 | |
| | Skewness | | ,612 | ,845 |
| | Kurtosis | | -,734 | 1,741 |
| KSR_Perc_Int_W5 | Mean | | 47,6033 | 4,23673 |
| | 95% Confidence Interval for Mean | Lower Bound | 36,7125 | |
| | | Upper Bound | 58,4942 | |
| | 5% Trimmed Mean | | 47,8476 | |
| | Median | | 52,1800 | |
| | Variance | | 107,699 | |
| | Std. Deviation | | 10,37783 | |
| | Minimum | | 33,86 | |
| | Maximum | | 56,95 | |
| | Range | | 23,09 | |
| | Interquartile Range | | 21,08 | |
| | Skewness | | -,829 | ,845 |
| | Kurtosis | | -1,838 | 1,741 |
| KSR_Perc_Nec_W5 | Mean | | 15,4583 | 4,04664 |
| | 95% Confidence Interval for Mean | Lower Bound | 5,0561 | |
| | | Upper Bound | 25,8606 | |
| | 5% Trimmed Mean | | 15,2498 | |
| | Median | | 14,0950 | |
| | Variance | | 98,252 | |
| | Std. Deviation | | 9,91221 | |
| | Minimum | | 1,92 | |
| | Maximum | | 32,75 | |
| | Range | | 30,83 | |
| | Interquartile Range | | 9,88 | |

Descriptives

| | | | Statistic | Std. Error |
|---|---|---|---|---|
| | Skewness | | ,824 | ,845 |
| | Kurtosis | | 2,671 | 1,741 |
| KSR_GermCells_W5 | Mean | | ,0831 | ,05066 |
| | 95% Confidence Interval for Mean | Lower Bound | -,0471 | |
| | | Upper Bound | ,2134 | |
| | 5% Trimmed Mean | | ,0731 | |
| | Median | | ,0378 | |
| | Variance | | ,015 | |
| | Std. Deviation | | ,12410 | |
| | Minimum | | ,01 | |
| | Maximum | | ,33 | |
| | Range | | ,32 | |
| | Interquartile Range | | ,11 | |
| | Skewness | | 2,358 | ,845 |
| | Kurtosis | | 5,646 | 1,741 |
| KSR_Perc_ST_W6 | Mean | | 24,9600 | 2,82312 |
| | 95% Confidence Interval for Mean | Lower Bound | 17,7030 | |
| | | Upper Bound | 32,2170 | |
| | 5% Trimmed Mean | | 24,6500 | |
| | Median | | 23,2050 | |
| | Variance | | 47,820 | |
| | Std. Deviation | | 6,91519 | |
| | Minimum | | 17,86 | |
| | Maximum | | 37,64 | |
| | Range | | 19,78 | |
| | Interquartile Range | | 9,23 | |
| | Skewness | | 1,451 | ,845 |
| | Kurtosis | | 2,541 | 1,741 |
| KSR_Perc_Int_W6 | Mean | | 56,7650 | 4,42854 |
| | 95% Confidence Interval for Mean | Lower Bound | 45,3811 | |
| | | Upper Bound | 68,1489 | |
| | 5% Trimmed Mean | | 56,7461 | |
| | Median | | 57,5950 | |
| | Variance | | 117,672 | |
| | Std. Deviation | | 10,84767 | |
| | Minimum | | 41,21 | |
| | Maximum | | 72,66 | |
| | Range | | 31,45 | |
| | Interquartile Range | | 17,89 | |
| | Skewness | | -,003 | ,845 |
| | Kurtosis | | ,166 | 1,741 |
| KSR_Perc_Nec_W6 | Mean | | 18,2750 | 2,67856 |
| | 95% Confidence Interval for Mean | Lower Bound | 11,3895 | |
| | | Upper Bound | 25,1605 | |
| | 5% Trimmed Mean | | 18,1678 | |
| | Median | | 17,5500 | |
| | Variance | | 43,048 | |
| | Std. Deviation | | 6,56110 | |
| | Minimum | | 9,48 | |
| | Maximum | | 29,00 | |
| | Range | | 19,52 | |

## Descriptives

| | | | Statistic | Std. Error |
|---|---|---|---|---|
| | Interquartile Range | | 9,55 | |
| | Skewness | | ,567 | ,845 |
| | Kurtosis | | 1,086 | 1,741 |
| KSR_GermCells_W6 | Mean | | ,1529 | ,11790 |
| | 95% Confidence Interval for Mean | Lower Bound | -,1502 | |
| | | Upper Bound | ,4559 | |
| | 5% Trimmed Mean | | ,1287 | |
| | Median | | ,0356 | |
| | Variance | | ,083 | |
| | Std. Deviation | | ,28880 | |
| | Minimum | | ,00 | |
| | Maximum | | ,74 | |
| | Range | | ,74 | |
| | Interquartile Range | | ,23 | |
| | Skewness | | 2,404 | ,845 |
| | Kurtosis | | 5,822 | 1,741 |
| EST_Perc_ST_W2 | Mean | | 25,7175 | 7,73648 |
| | 95% Confidence Interval for Mean | Lower Bound | 1,0966 | |
| | | Upper Bound | 50,3384 | |
| | 5% Trimmed Mean | | 25,5300 | |
| | Median | | 24,0300 | |
| | Variance | | 239,413 | |
| | Std. Deviation | | 15,47297 | |
| | Minimum | | 8,62 | |
| | Maximum | | 46,19 | |
| | Range | | 37,57 | |
| | Interquartile Range | | 28,55 | |
| | Skewness | | ,642 | 1,014 |
| | Kurtosis | | 1,687 | 2,619 |
| EST_Perc_Int_W2 | Mean | | 54,4850 | 6,97716 |
| | 95% Confidence Interval for Mean | Lower Bound | 32,2806 | |
| | | Upper Bound | 76,6894 | |
| | 5% Trimmed Mean | | 54,4806 | |
| | Median | | 54,4450 | |
| | Variance | | 194,723 | |
| | Std. Deviation | | 13,95432 | |
| | Minimum | | 37,96 | |
| | Maximum | | 71,09 | |
| | Range | | 33,13 | |
| | Interquartile Range | | 26,95 | |
| | Skewness | | ,015 | 1,014 |
| | Kurtosis | | -,206 | 2,619 |
| EST_Perc_Nec_W2 | Mean | | 19,8000 | 1,95397 |
| | 95% Confidence Interval for Mean | Lower Bound | 13,5816 | |
| | | Upper Bound | 26,0184 | |
| | 5% Trimmed Mean | | 19,7311 | |
| | Median | | 19,1800 | |
| | Variance | | 15,272 | |
| | Std. Deviation | | 3,90795 | |
| | Minimum | | 15,85 | |
| | Maximum | | 24,99 | |

**Descriptives**

| | | | Statistic | Std. Error |
|---|---|---|---|---|
| | Range | | 9,14 | |
| | Interquartile Range | | 7,42 | |
| | Skewness | | ,816 | 1,014 |
| | Kurtosis | | ,480 | 2,619 |
| EST_GermCells_W2 | Mean | | ,7909 | ,36523 |
| | 95% Confidence Interval for Mean | Lower Bound | -,3714 | |
| | | Upper Bound | 1,9532 | |
| | 5% Trimmed Mean | | ,7634 | |
| | Median | | ,5434 | |
| | Variance | | ,534 | |
| | Std. Deviation | | ,73045 | |
| | Minimum | | ,26 | |
| | Maximum | | 1,82 | |
| | Range | | 1,56 | |
| | Interquartile Range | | 1,30 | |
| | Skewness | | 1,385 | 1,014 |
| | Kurtosis | | 1,369 | 2,619 |
| EST_Perc_ST_W3 | Mean | | 27,1850 | 3,32626 |
| | 95% Confidence Interval for Mean | Lower Bound | 18,6346 | |
| | | Upper Bound | 35,7354 | |
| | 5% Trimmed Mean | | 26,7533 | |
| | Median | | 26,2300 | |
| | Variance | | 66,384 | |
| | Std. Deviation | | 8,14763 | |
| | Minimum | | 19,84 | |
| | Maximum | | 42,30 | |
| | Range | | 22,46 | |
| | Interquartile Range | | 11,26 | |
| | Skewness | | 1,525 | ,845 |
| | Kurtosis | | 2,825 | 1,741 |
| EST_Perc_Int_W3 | Mean | | 53,0567 | 6,46859 |
| | 95% Confidence Interval for Mean | Lower Bound | 36,4286 | |
| | | Upper Bound | 69,6847 | |
| | 5% Trimmed Mean | | 52,9646 | |
| | Median | | 52,5750 | |
| | Variance | | 251,056 | |
| | Std. Deviation | | 15,84475 | |
| | Minimum | | 29,91 | |
| | Maximum | | 77,86 | |
| | Range | | 47,95 | |
| | Interquartile Range | | 22,47 | |
| | Skewness | | ,207 | ,845 |
| | Kurtosis | | 1,195 | 1,741 |
| EST_Perc_Nec_W3 | Mean | | 19,7600 | 4,27675 |
| | 95% Confidence Interval for Mean | Lower Bound | 8,7663 | |
| | | Upper Bound | 30,7537 | |
| | 5% Trimmed Mean | | 20,2356 | |
| | Median | | 22,3500 | |
| | Variance | | 109,743 | |
| | Std. Deviation | | 10,47585 | |
| | Minimum | | 1,66 | |

Descriptives

| | | | Statistic | Std. Error |
|---|---|---|---|---|
| | Maximum | | 29,30 | |
| | Range | | 27,64 | |
| | Interquartile Range | | 16,42 | |
| | Skewness | | -1,141 | ,845 |
| | Kurtosis | | ,842 | 1,741 |
| EST_GermCells_W3 | Mean | | ,4622 | ,21909 |
| | 95% Confidence Interval for Mean | Lower Bound | -,1010 | |
| | | Upper Bound | 1,0253 | |
| | 5% Trimmed Mean | | ,4245 | |
| | Median | | ,2863 | |
| | Variance | | ,288 | |
| | Std. Deviation | | ,53666 | |
| | Minimum | | ,10 | |
| | Maximum | | 1,51 | |
| | Range | | 1,41 | |
| | Interquartile Range | | ,61 | |
| | Skewness | | 1,985 | ,845 |
| | Kurtosis | | 4,159 | 1,741 |
| EST_Perc_ST_W4 | Mean | | 28,3350 | 6,46668 |
| | 95% Confidence Interval for Mean | Lower Bound | 11,7119 | |
| | | Upper Bound | 44,9581 | |
| | 5% Trimmed Mean | | 28,4122 | |
| | Median | | 28,9550 | |
| | Variance | | 250,908 | |
| | Std. Deviation | | 15,84007 | |
| | Minimum | | 10,51 | |
| | Maximum | | 44,77 | |
| | Range | | 34,26 | |
| | Interquartile Range | | 29,30 | |
| | Skewness | | -,046 | ,845 |
| | Kurtosis | | -3,049 | 1,741 |
| EST_Perc_Int_W4 | Mean | | 57,4183 | 6,22269 |
| | 95% Confidence Interval for Mean | Lower Bound | 41,4224 | |
| | | Upper Bound | 73,4143 | |
| | 5% Trimmed Mean | | 56,8704 | |
| | Median | | 53,4450 | |
| | Variance | | 232,331 | |
| | Std. Deviation | | 15,24242 | |
| | Minimum | | 42,39 | |
| | Maximum | | 82,31 | |
| | Range | | 39,92 | |
| | Interquartile Range | | 26,78 | |
| | Skewness | | ,915 | ,845 |
| | Kurtosis | | -,132 | 1,741 |
| EST_Perc_Nec_W4 | Mean | | 14,2483 | 4,82877 |
| | 95% Confidence Interval for Mean | Lower Bound | 1,8356 | |
| | | Upper Bound | 26,6611 | |
| | 5% Trimmed Mean | | 13,7393 | |
| | Median | | 12,6350 | |
| | Variance | | 139,902 | |
| | Std. Deviation | | 11,82801 | |

**Descriptives**

| | | | Statistic | Std. Error |
|---|---|---|---|---|
| | Minimum | | 1,70 | |
| | Maximum | | 35,96 | |
| | Range | | 34,26 | |
| | Interquartile Range | | 14,72 | |
| | Skewness | | 1,420 | ,845 |
| | Kurtosis | | 2,685 | 1,741 |
| EST_GermCells_W4 | Mean | | ,4863 | ,22352 |
| | 95% Confidence Interval for Mean | Lower Bound | -,0883 | |
| | | Upper Bound | 1,0609 | |
| | 5% Trimmed Mean | | ,4546 | |
| | Median | | ,3407 | |
| | Variance | | ,300 | |
| | Std. Deviation | | ,54751 | |
| | Minimum | | ,05 | |
| | Maximum | | 1,50 | |
| | Range | | 1,45 | |
| | Interquartile Range | | ,79 | |
| | Skewness | | 1,541 | ,845 |
| | Kurtosis | | 2,425 | 1,741 |
| EST_Perc_ST_W5 | Mean | | 31,4617 | 7,33765 |
| | 95% Confidence Interval for Mean | Lower Bound | 12,5996 | |
| | | Upper Bound | 50,3237 | |
| | 5% Trimmed Mean | | 31,5941 | |
| | Median | | 36,0500 | |
| | Variance | | 323,047 | |
| | Std. Deviation | | 17,97350 | |
| | Minimum | | 6,23 | |
| | Maximum | | 54,31 | |
| | Range | | 48,08 | |
| | Interquartile Range | | 32,64 | |
| | Skewness | | -,397 | ,845 |
| | Kurtosis | | -1,029 | 1,741 |
| EST_Perc_Int_W5 | Mean | | 58,3767 | 6,68301 |
| | 95% Confidence Interval for Mean | Lower Bound | 41,1974 | |
| | | Upper Bound | 75,5559 | |
| | 5% Trimmed Mean | | 57,9630 | |
| | Median | | 56,2750 | |
| | Variance | | 267,976 | |
| | Std. Deviation | | 16,36996 | |
| | Minimum | | 41,42 | |
| | Maximum | | 82,78 | |
| | Range | | 41,36 | |
| | Interquartile Range | | 31,98 | |
| | Skewness | | ,509 | ,845 |
| | Kurtosis | | -1,068 | 1,741 |
| EST_Perc_Nec_W5 | Mean | | 10,1567 | 4,37774 |
| | 95% Confidence Interval for Mean | Lower Bound | -1,0967 | |
| | | Upper Bound | 21,4100 | |
| | 5% Trimmed Mean | | 9,8107 | |
| | Median | | 4,4550 | |
| | Variance | | 114,988 | |

**Descriptives**

| | | | Statistic | Std. Error |
|---|---|---|---|---|
| | Std. Deviation | | 10,72322 | |
| | Minimum | | 1,42 | |
| | Maximum | | 25,12 | |
| | Range | | 23,70 | |
| | Interquartile Range | | 20,69 | |
| | Skewness | | ,927 | ,845 |
| | Kurtosis | | -1,738 | 1,741 |
| EST_GermCells_W5 | Mean | | ,7355 | ,29008 |
| | 95% Confidence Interval for Mean | Lower Bound | -,0102 | |
| | | Upper Bound | 1,4812 | |
| | 5% Trimmed Mean | | ,7121 | |
| | Median | | ,5757 | |
| | Variance | | ,505 | |
| | Std. Deviation | | ,71055 | |
| | Minimum | | ,00 | |
| | Maximum | | 1,89 | |
| | Range | | 1,89 | |
| | Interquartile Range | | 1,28 | |
| | Skewness | | ,879 | ,845 |
| | Kurtosis | | -,027 | 1,741 |
| EST_Perc_ST_W6 | Mean | | 21,4083 | 6,84577 |
| | 95% Confidence Interval for Mean | Lower Bound | 3,8107 | |
| | | Upper Bound | 39,0059 | |
| | 5% Trimmed Mean | | 20,4826 | |
| | Median | | 15,3050 | |
| | Variance | | 281,187 | |
| | Std. Deviation | | 16,76864 | |
| | Minimum | | 7,33 | |
| | Maximum | | 52,15 | |
| | Range | | 44,82 | |
| | Interquartile Range | | 25,34 | |
| | Skewness | | 1,564 | ,845 |
| | Kurtosis | | 2,218 | 1,741 |
| EST_Perc_Int_W6 | Mean | | 65,2933 | 7,68179 |
| | 95% Confidence Interval for Mean | Lower Bound | 45,5467 | |
| | | Upper Bound | 85,0400 | |
| | 5% Trimmed Mean | | 66,7159 | |
| | Median | | 70,9800 | |
| | Variance | | 354,059 | |
| | Std. Deviation | | 18,81646 | |
| | Minimum | | 27,56 | |
| | Maximum | | 77,42 | |
| | Range | | 49,86 | |
| | Interquartile Range | | 18,60 | |
| | Skewness | | -2,258 | ,845 |
| | Kurtosis | | 5,291 | 1,741 |
| EST_Perc_Nec_W6 | Mean | | 13,2967 | 3,37104 |
| | 95% Confidence Interval for Mean | Lower Bound | 4,6311 | |
| | | Upper Bound | 21,9622 | |
| | 5% Trimmed Mean | | 13,5296 | |
| | Median | | 15,2200 | |

## Descriptives

| | | | Statistic | Std. Error |
|---|---|---|---|---|
| | Variance | | 68,183 | |
| | Std. Deviation | | 8,25733 | |
| | Minimum | | ,48 | |
| | Maximum | | 21,92 | |
| | Range | | 21,44 | |
| | Interquartile Range | | 15,59 | |
| | Skewness | | -,741 | ,845 |
| | Kurtosis | | -,690 | 1,741 |
| EST_GermCells_W6 | Mean | | ,2669 | ,13308 |
| | 95% Confidence Interval for Mean | Lower Bound | -,0752 | |
| | | Upper Bound | ,6090 | |
| | 5% Trimmed Mean | | ,2500 | |
| | Median | | ,1387 | |
| | Variance | | ,106 | |
| | Std. Deviation | | ,32597 | |
| | Minimum | | ,00 | |
| | Maximum | | ,84 | |
| | Range | | ,84 | |
| | Interquartile Range | | ,52 | |
| | Skewness | | 1,309 | ,845 |
| | Kurtosis | | 1,024 | 1,741 |
| ALB_Perc_ST_W2 | Mean | | 40,3360 | 6,44955 |
| | 95% Confidence Interval for Mean | Lower Bound | 22,4292 | |
| | | Upper Bound | 58,2428 | |
| | 5% Trimmed Mean | | 40,3261 | |
| | Median | | 44,0100 | |
| | Variance | | 207,983 | |
| | Std. Deviation | | 14,42163 | |
| | Minimum | | 23,69 | |
| | Maximum | | 57,16 | |
| | Range | | 33,47 | |
| | Interquartile Range | | 27,94 | |
| | Skewness | | -,176 | ,913 |
| | Kurtosis | | -2,389 | 2,000 |
| ALB_Perc_Int_W2 | Mean | | 28,0380 | 7,84729 |
| | 95% Confidence Interval for Mean | Lower Bound | 6,2504 | |
| | | Upper Bound | 49,8256 | |
| | 5% Trimmed Mean | | 28,1411 | |
| | Median | | 28,3500 | |
| | Variance | | 307,900 | |
| | Std. Deviation | | 17,54708 | |
| | Minimum | | 4,88 | |
| | Maximum | | 49,34 | |
| | Range | | 44,46 | |
| | Interquartile Range | | 33,19 | |
| | Skewness | | -,169 | ,913 |
| | Kurtosis | | -1,101 | 2,000 |
| ALB_Perc_Nec_W2 | Mean | | 31,6280 | 8,02770 |
| | 95% Confidence Interval for Mean | Lower Bound | 9,3395 | |
| | | Upper Bound | 53,9165 | |
| | 5% Trimmed Mean | | 31,2850 | |

Descriptives

| | | | Statistic | Std. Error |
|---|---|---|---|---|
| | Median | | 26,9800 | |
| | Variance | | 322,220 | |
| | Std. Deviation | | 17,95049 | |
| | Minimum | | 14,49 | |
| | Maximum | | 54,94 | |
| | Range | | 40,45 | |
| | Interquartile Range | | 34,87 | |
| | Skewness | | ,472 | ,913 |
| | Kurtosis | | -2,274 | 2,000 |
| ALB_GermCells_W2 | Mean | | ,7041 | ,22678 |
| | 95% Confidence Interval for Mean | Lower Bound | ,0744 | |
| | | Upper Bound | 1,3337 | |
| | 5% Trimmed Mean | | ,6822 | |
| | Median | | ,5098 | |
| | Variance | | ,257 | |
| | Std. Deviation | | ,50711 | |
| | Minimum | | ,24 | |
| | Maximum | | 1,56 | |
| | Range | | 1,31 | |
| | Interquartile Range | | ,78 | |
| | Skewness | | 1,620 | ,913 |
| | Kurtosis | | 2,969 | 2,000 |
| ALB_Perc_ST_W3 | Mean | | 18,4050 | 4,66963 |
| | 95% Confidence Interval for Mean | Lower Bound | 6,4013 | |
| | | Upper Bound | 30,4087 | |
| | 5% Trimmed Mean | | 17,9544 | |
| | Median | | 13,9950 | |
| | Variance | | 130,833 | |
| | Std. Deviation | | 11,43822 | |
| | Minimum | | 6,34 | |
| | Maximum | | 38,58 | |
| | Range | | 32,24 | |
| | Interquartile Range | | 16,40 | |
| | Skewness | | 1,271 | ,845 |
| | Kurtosis | | 1,506 | 1,741 |
| ALB_Perc_Int_W3 | Mean | | 46,7550 | 8,32765 |
| | 95% Confidence Interval for Mean | Lower Bound | 25,3481 | |
| | | Upper Bound | 68,1619 | |
| | 5% Trimmed Mean | | 46,6700 | |
| | Median | | 44,9150 | |
| | Variance | | 416,099 | |
| | Std. Deviation | | 20,39850 | |
| | Minimum | | 18,43 | |
| | Maximum | | 76,61 | |
| | Range | | 58,18 | |
| | Interquartile Range | | 32,11 | |
| | Skewness | | ,148 | ,845 |
| | Kurtosis | | -,246 | 1,741 |
| ALB_Perc_Nec_W3 | Mean | | 34,8383 | 7,19451 |
| | 95% Confidence Interval for Mean | Lower Bound | 16,3443 | |
| | | Upper Bound | 53,3324 | |

## Descriptives

| | | | Statistic | Std. Error |
|---|---|---|---|---|
| | 5% Trimmed Mean | | 34,9604 | |
| | Median | | 35,1250 | |
| | Variance | | 310,565 | |
| | Std. Deviation | | 17,62287 | |
| | Minimum | | 9,94 | |
| | Maximum | | 57,54 | |
| | Range | | 47,60 | |
| | Interquartile Range | | 30,04 | |
| | Skewness | | -,160 | ,845 |
| | Kurtosis | | -1,159 | 1,741 |
| ALB_GermCells_W3 | Mean | | ,6488 | ,37471 |
| | 95% Confidence Interval for Mean | Lower Bound | -,3144 | |
| | | Upper Bound | 1,6120 | |
| | 5% Trimmed Mean | | ,5782 | |
| | Median | | ,2837 | |
| | Variance | | ,842 | |
| | Std. Deviation | | ,91785 | |
| | Minimum | | ,07 | |
| | Maximum | | 2,50 | |
| | Range | | 2,42 | |
| | Interquartile Range | | ,84 | |
| | Skewness | | 2,301 | ,845 |
| | Kurtosis | | 5,419 | 1,741 |
| ALB_Perc_ST_W4 | Mean | | 24,1133 | 2,64168 |
| | 95% Confidence Interval for Mean | Lower Bound | 17,3227 | |
| | | Upper Bound | 30,9040 | |
| | 5% Trimmed Mean | | 24,2298 | |
| | Median | | 25,8200 | |
| | Variance | | 41,871 | |
| | Std. Deviation | | 6,47076 | |
| | Minimum | | 14,11 | |
| | Maximum | | 32,02 | |
| | Range | | 17,91 | |
| | Interquartile Range | | 10,88 | |
| | Skewness | | -,609 | ,845 |
| | Kurtosis | | -,354 | 1,741 |
| ALB_Perc_Int_W4 | Mean | | 54,3467 | 7,70383 |
| | 95% Confidence Interval for Mean | Lower Bound | 34,5433 | |
| | | Upper Bound | 74,1500 | |
| | 5% Trimmed Mean | | 54,4791 | |
| | Median | | 52,8100 | |
| | Variance | | 356,094 | |
| | Std. Deviation | | 18,87045 | |
| | Minimum | | 25,50 | |
| | Maximum | | 80,81 | |
| | Range | | 55,31 | |
| | Interquartile Range | | 28,63 | |
| | Skewness | | -,179 | ,845 |
| | Kurtosis | | ,439 | 1,741 |
| ALB_Perc_Nec_W4 | Mean | | 21,5417 | 7,01091 |

## Descriptives

| | | | Statistic | Std. Error |
|---|---|---|---|---|
| | 95% Confidence Interval for Mean | Lower Bound | 3,5196 | |
| | | Upper Bound | 39,5638 | |
| | 5% Trimmed Mean | | 21,3063 | |
| | Median | | 23,8050 | |
| | Variance | | 294,917 | |
| | Std. Deviation | | 17,17314 | |
| | Minimum | | ,00 | |
| | Maximum | | 47,32 | |
| | Range | | 47,32 | |
| | Interquartile Range | | 29,55 | |
| | Skewness | | ,204 | ,845 |
| | Kurtosis | | -,379 | 1,741 |
| ALB_GermCells_W4 | Mean | | ,5977 | ,32036 |
| | 95% Confidence Interval for Mean | Lower Bound | -,2258 | |
| | | Upper Bound | 1,4212 | |
| | 5% Trimmed Mean | | ,5464 | |
| | Median | | ,3594 | |
| | Variance | | ,616 | |
| | Std. Deviation | | ,78472 | |
| | Minimum | | ,00 | |
| | Maximum | | 2,12 | |
| | Range | | 2,12 | |
| | Interquartile Range | | ,99 | |
| | Skewness | | 1,944 | ,845 |
| | Kurtosis | | 4,045 | 1,741 |
| ALB_Perc_ST_W5 | Mean | | 27,1433 | 6,24199 |
| | 95% Confidence Interval for Mean | Lower Bound | 11,0978 | |
| | | Upper Bound | 43,1889 | |
| | 5% Trimmed Mean | | 27,6270 | |
| | Median | | 29,5500 | |
| | Variance | | 233,775 | |
| | Std. Deviation | | 15,28969 | |
| | Minimum | | ,00 | |
| | Maximum | | 45,58 | |
| | Range | | 45,58 | |
| | Interquartile Range | | 19,59 | |
| | Skewness | | -1,112 | ,845 |
| | Kurtosis | | 2,231 | 1,741 |
| ALB_Perc_Int_W5 | Mean | | 40,9667 | 10,77429 |
| | 95% Confidence Interval for Mean | Lower Bound | 13,2705 | |
| | | Upper Bound | 68,6629 | |
| | 5% Trimmed Mean | | 41,4607 | |
| | Median | | 46,1200 | |
| | Variance | | 696,513 | |
| | Std. Deviation | | 26,39152 | |
| | Minimum | | ,00 | |
| | Maximum | | 73,04 | |
| | Range | | 73,04 | |
| | Interquartile Range | | 45,61 | |
| | Skewness | | -,588 | ,845 |
| | Kurtosis | | -,320 | 1,741 |

## Descriptives

| | | | Statistic | Std. Error |
|---|---|---|---|---|
| ALB_Perc_Nec_W5 | Mean | | 31,8917 | 14,94396 |
| | 95% Confidence Interval for Mean | Lower Bound | -6,5230 | |
| | | Upper Bound | 70,3064 | |
| | 5% Trimmed Mean | | 29,8796 | |
| | Median | | 15,9400 | |
| | Variance | | 1339,932 | |
| | Std. Deviation | | 36,60509 | |
| | Minimum | | ,00 | |
| | Maximum | | 100,00 | |
| | Range | | 100,00 | |
| | Interquartile Range | | 49,14 | |
| | Skewness | | 1,662 | ,845 |
| | Kurtosis | | 2,656 | 1,741 |
| ALB_GermCells_W5 | Mean | | ,0805 | ,06731 |
| | 95% Confidence Interval for Mean | Lower Bound | -,0925 | |
| | | Upper Bound | ,2535 | |
| | 5% Trimmed Mean | | ,0664 | |
| | Median | | ,0086 | |
| | Variance | | ,027 | |
| | Std. Deviation | | ,16489 | |
| | Minimum | | ,00 | |
| | Maximum | | ,41 | |
| | Range | | ,41 | |
| | Interquartile Range | | ,14 | |
| | Skewness | | 2,372 | ,845 |
| | Kurtosis | | 5,685 | 1,741 |
| ALB_Perc_ST_W6 | Mean | | 39,6550 | 5,39606 |
| | 95% Confidence Interval for Mean | Lower Bound | 25,7840 | |
| | | Upper Bound | 53,5260 | |
| | 5% Trimmed Mean | | 39,9739 | |
| | Median | | 40,4700 | |
| | Variance | | 174,705 | |
| | Std. Deviation | | 13,21760 | |
| | Minimum | | 17,41 | |
| | Maximum | | 56,16 | |
| | Range | | 38,75 | |
| | Interquartile Range | | 19,98 | |
| | Skewness | | -,767 | ,845 |
| | Kurtosis | | 1,225 | 1,741 |
| ALB_Perc_Int_W6 | Mean | | 42,9017 | 4,13440 |
| | 95% Confidence Interval for Mean | Lower Bound | 32,2738 | |
| | | Upper Bound | 53,5295 | |
| | 5% Trimmed Mean | | 43,2735 | |
| | Median | | 42,3450 | |
| | Variance | | 102,560 | |
| | Std. Deviation | | 10,12718 | |
| | Minimum | | 25,62 | |
| | Maximum | | 53,49 | |
| | Range | | 27,87 | |
| | Interquartile Range | | 15,87 | |
| | Skewness | | -,876 | ,845 |

## Descriptives

| | | | Statistic | Std. Error |
|---|---|---|---|---|
| | Kurtosis | | 1,147 | 1,741 |
| ALB_Perc_Nec_W6 | Mean | | 17,4450 | 2,64608 |
| | 95% Confidence Interval for Mean | Lower Bound | 10,6430 | |
| | | Upper Bound | 24,2470 | |
| | 5% Trimmed Mean | | 17,1800 | |
| | Median | | 17,1850 | |
| | Variance | | 42,011 | |
| | Std. Deviation | | 6,48156 | |
| | Minimum | | 10,56 | |
| | Maximum | | 29,10 | |
| | Range | | 18,54 | |
| | Interquartile Range | | 9,00 | |
| | Skewness | | 1,244 | ,845 |
| | Kurtosis | | 2,215 | 1,741 |
| ALB_GermCells_W6 | Mean | | ,3418 | ,18200 |
| | 95% Confidence Interval for Mean | Lower Bound | -,1261 | |
| | | Upper Bound | ,8097 | |
| | 5% Trimmed Mean | | ,3141 | |
| | Median | | ,1495 | |
| | Variance | | ,199 | |
| | Std. Deviation | | ,44581 | |
| | Minimum | | ,01 | |
| | Maximum | | 1,17 | |
| | Range | | 1,17 | |
| | Interquartile Range | | ,64 | |
| | Skewness | | 1,710 | ,845 |
| | Kurtosis | | 2,656 | 1,741 |
| ALB_EST_Perc_ST_W2 | Mean | | 30,4000 | 6,75947 |
| | 95% Confidence Interval for Mean | Lower Bound | 11,6327 | |
| | | Upper Bound | 49,1673 | |
| | 5% Trimmed Mean | | 30,2539 | |
| | Median | | 31,4600 | |
| | Variance | | 228,452 | |
| | Std. Deviation | | 15,11463 | |
| | Minimum | | 10,91 | |
| | Maximum | | 52,52 | |
| | Range | | 41,61 | |
| | Interquartile Range | | 25,09 | |
| | Skewness | | ,387 | ,913 |
| | Kurtosis | | 1,225 | 2,000 |
| ALB_EST_Perc_Int_W2 | Mean | | 27,1440 | 4,46202 |
| | 95% Confidence Interval for Mean | Lower Bound | 14,7554 | |
| | | Upper Bound | 39,5326 | |
| | 5% Trimmed Mean | | 27,3522 | |
| | Median | | 29,9400 | |
| | Variance | | 99,548 | |
| | Std. Deviation | | 9,97738 | |
| | Minimum | | 12,46 | |
| | Maximum | | 38,08 | |
| | Range | | 25,62 | |
| | Interquartile Range | | 18,04 | |

### Descriptives

| | | | Statistic | Std. Error |
|---|---|---|---|---|
| | Skewness | | -,738 | ,913 |
| | Kurtosis | | -,126 | 2,000 |
| ALB_EST_Perc_Nec_W2 | Mean | | 42,4580 | 8,66909 |
| | 95% Confidence Interval for Mean | Lower Bound | 18,3887 | |
| | | Upper Bound | 66,5273 | |
| | 5% Trimmed Mean | | 42,4950 | |
| | Median | | 42,8800 | |
| | Variance | | 375,766 | |
| | Std. Deviation | | 19,38468 | |
| | Minimum | | 17,54 | |
| | Maximum | | 66,71 | |
| | Range | | 49,17 | |
| | Interquartile Range | | 36,71 | |
| | Skewness | | -,063 | ,913 |
| | Kurtosis | | -1,142 | 2,000 |
| ALB_EST_GermCells_W2 | Mean | | ,7794 | ,38289 |
| | 95% Confidence Interval for Mean | Lower Bound | -,2837 | |
| | | Upper Bound | 1,8425 | |
| | 5% Trimmed Mean | | ,7354 | |
| | Median | | ,4474 | |
| | Variance | | ,733 | |
| | Std. Deviation | | ,85617 | |
| | Minimum | | ,09 | |
| | Maximum | | 2,26 | |
| | Range | | 2,16 | |
| | Interquartile Range | | 1,26 | |
| | Skewness | | 1,858 | ,913 |
| | Kurtosis | | 3,688 | 2,000 |
| ALB_EST_Perc_ST_W3 | Mean | | 33,3283 | 4,63700 |
| | 95% Confidence Interval for Mean | Lower Bound | 21,4085 | |
| | | Upper Bound | 45,2481 | |
| | 5% Trimmed Mean | | 32,8126 | |
| | Median | | 32,0900 | |
| | Variance | | 129,011 | |
| | Std. Deviation | | 11,35829 | |
| | Minimum | | 22,09 | |
| | Maximum | | 53,85 | |
| | Range | | 31,76 | |
| | Interquartile Range | | 16,86 | |
| | Skewness | | 1,314 | ,845 |
| | Kurtosis | | 2,204 | 1,741 |
| ALB_EST_Perc_Int_W3 | Mean | | 43,4133 | 3,00855 |
| | 95% Confidence Interval for Mean | Lower Bound | 35,6796 | |
| | | Upper Bound | 51,1471 | |
| | 5% Trimmed Mean | | 43,6420 | |
| | Median | | 46,2900 | |
| | Variance | | 54,308 | |
| | Std. Deviation | | 7,36941 | |
| | Minimum | | 32,47 | |
| | Maximum | | 50,24 | |
| | Range | | 17,77 | |

**Descriptives**

| | | | Statistic | Std. Error |
|---|---|---|---|---|
| | Interquartile Range | | 14,35 | |
| | Skewness | | -,869 | ,845 |
| | Kurtosis | | -1,311 | 1,741 |
| ALB_EST_Perc_Nec_W3 | Mean | | 23,2583 | 5,27968 |
| | 95% Confidence Interval for Mean | Lower Bound | 9,6865 | |
| | | Upper Bound | 36,8302 | |
| | 5% Trimmed Mean | | 23,8409 | |
| | Median | | 27,2450 | |
| | Variance | | 167,250 | |
| | Std. Deviation | | 12,93252 | |
| | Minimum | | ,00 | |
| | Maximum | | 36,03 | |
| | Range | | 36,03 | |
| | Interquartile Range | | 19,23 | |
| | Skewness | | -1,382 | ,845 |
| | Kurtosis | | 1,892 | 1,741 |
| ALB_EST_GermCells_W3 | Mean | | ,5218 | ,26975 |
| | 95% Confidence Interval for Mean | Lower Bound | -,1716 | |
| | | Upper Bound | 1,2152 | |
| | 5% Trimmed Mean | | ,4796 | |
| | Median | | ,3311 | |
| | Variance | | ,437 | |
| | Std. Deviation | | ,66074 | |
| | Minimum | | ,01 | |
| | Maximum | | 1,79 | |
| | Range | | 1,77 | |
| | Interquartile Range | | ,87 | |
| | Skewness | | 1,849 | ,845 |
| | Kurtosis | | 3,671 | 1,741 |
| ALB_EST_Perc_ST_W4 | Mean | | 14,7800 | 3,35064 |
| | 95% Confidence Interval for Mean | Lower Bound | 6,1669 | |
| | | Upper Bound | 23,3931 | |
| | 5% Trimmed Mean | | 15,2083 | |
| | Median | | 17,2450 | |
| | Variance | | 67,361 | |
| | Std. Deviation | | 8,20736 | |
| | Minimum | | ,00 | |
| | Maximum | | 21,85 | |
| | Range | | 21,85 | |
| | Interquartile Range | | 13,13 | |
| | Skewness | | -1,422 | ,845 |
| | Kurtosis | | 1,814 | 1,741 |
| ALB_EST_Perc_Int_W4 | Mean | | 45,3133 | 9,57449 |
| | 95% Confidence Interval for Mean | Lower Bound | 20,7013 | |
| | | Upper Bound | 69,9254 | |
| | 5% Trimmed Mean | | 46,8076 | |
| | Median | | 52,6550 | |
| | Variance | | 550,026 | |
| | Std. Deviation | | 23,45263 | |
| | Minimum | | ,00 | |
| | Maximum | | 63,73 | |

## Descriptives

| | | | Statistic | Std. Error |
|---|---|---|---|---|
| | Range | | 63,73 | |
| | Interquartile Range | | 27,72 | |
| | Skewness | | -1,910 | ,845 |
| | Kurtosis | | 3,915 | 1,741 |
| ALB_EST_Perc_Nec_W4 | Mean | | 39,9083 | 12,43355 |
| | 95% Confidence Interval for Mean | Lower Bound | 7,9469 | |
| | | Upper Bound | 71,8698 | |
| | 5% Trimmed Mean | | 37,9859 | |
| | Median | | 32,4150 | |
| | Variance | | 927,559 | |
| | Std. Deviation | | 30,45585 | |
| | Minimum | | 14,42 | |
| | Maximum | | 100,00 | |
| | Range | | 85,58 | |
| | Interquartile Range | | 29,42 | |
| | Skewness | | 2,078 | ,845 |
| | Kurtosis | | 4,755 | 1,741 |
| ALB_EST_GermCells_W4 | Mean | | ,6035 | ,37849 |
| | 95% Confidence Interval for Mean | Lower Bound | -,3695 | |
| | | Upper Bound | 1,5764 | |
| | 5% Trimmed Mean | | ,5355 | |
| | Median | | ,2411 | |
| | Variance | | ,860 | |
| | Std. Deviation | | ,92710 | |
| | Minimum | | ,00 | |
| | Maximum | | 2,43 | |
| | Range | | 2,43 | |
| | Interquartile Range | | 1,03 | |
| | Skewness | | 2,106 | ,845 |
| | Kurtosis | | 4,599 | 1,741 |
| ALB_EST_Perc_ST_W5 | Mean | | 26,5650 | 7,05943 |
| | 95% Confidence Interval for Mean | Lower Bound | 8,4182 | |
| | | Upper Bound | 44,7118 | |
| | 5% Trimmed Mean | | 26,7822 | |
| | Median | | 27,4100 | |
| | Variance | | 299,013 | |
| | Std. Deviation | | 17,29199 | |
| | Minimum | | 4,58 | |
| | Maximum | | 44,64 | |
| | Range | | 40,06 | |
| | Interquartile Range | | 34,97 | |
| | Skewness | | -,148 | ,845 |
| | Kurtosis | | -2,286 | 1,741 |
| ALB_EST_Perc_Int_W5 | Mean | | 43,7500 | 8,01675 |
| | 95% Confidence Interval for Mean | Lower Bound | 23,1423 | |
| | | Upper Bound | 64,3577 | |
| | 5% Trimmed Mean | | 43,1506 | |
| | Median | | 43,1300 | |
| | Variance | | 385,609 | |
| | Std. Deviation | | 19,63694 | |
| | Minimum | | 23,45 | |

**Descriptives**

| | | | Statistic | Std. Error |
|---|---|---|---|---|
| | Maximum | | 74,84 | |
| | Range | | 51,39 | |
| | Interquartile Range | | 34,67 | |
| | Skewness | | ,604 | ,845 |
| | Kurtosis | | -,338 | 1,741 |
| ALB_EST_Perc_Nec_W5 | Mean | | 29,6850 | 8,81267 |
| | 95% Confidence Interval for Mean | Lower Bound | 7,0313 | |
| | | Upper Bound | 52,3387 | |
| | 5% Trimmed Mean | | 29,7072 | |
| | Median | | 25,9200 | |
| | Variance | | 465,979 | |
| | Std. Deviation | | 21,58655 | |
| | Minimum | | 1,84 | |
| | Maximum | | 57,13 | |
| | Range | | 55,29 | |
| | Interquartile Range | | 41,57 | |
| | Skewness | | ,197 | ,845 |
| | Kurtosis | | -1,496 | 1,741 |
| ALB_EST_GermCells_W5 | Mean | | ,5292 | ,27358 |
| | 95% Confidence Interval for Mean | Lower Bound | -,1741 | |
| | | Upper Bound | 1,2324 | |
| | 5% Trimmed Mean | | ,4877 | |
| | Median | | ,2856 | |
| | Variance | | ,449 | |
| | Std. Deviation | | ,67012 | |
| | Minimum | | ,00 | |
| | Maximum | | 1,81 | |
| | Range | | 1,81 | |
| | Interquartile Range | | ,85 | |
| | Skewness | | 1,804 | ,845 |
| | Kurtosis | | 3,389 | 1,741 |
| ALB_EST_Perc_ST_W6 | Mean | | 48,6167 | 7,88178 |
| | 95% Confidence Interval for Mean | Lower Bound | 28,3559 | |
| | | Upper Bound | 68,8774 | |
| | 5% Trimmed Mean | | 48,4013 | |
| | Median | | 40,5950 | |
| | Variance | | 372,735 | |
| | Std. Deviation | | 19,30634 | |
| | Minimum | | 26,84 | |
| | Maximum | | 74,27 | |
| | Range | | 47,43 | |
| | Interquartile Range | | 36,20 | |
| | Skewness | | ,648 | ,845 |
| | Kurtosis | | -1,587 | 1,741 |
| ALB_EST_Perc_Int_W6 | Mean | | 34,1100 | 4,04251 |
| | 95% Confidence Interval for Mean | Lower Bound | 23,7184 | |
| | | Upper Bound | 44,5016 | |
| | 5% Trimmed Mean | | 33,8667 | |
| | Median | | 32,9950 | |
| | Variance | | 98,051 | |
| | Std. Deviation | | 9,90209 | |

## Descriptives

| | | | Statistic | Std. Error |
|---|---|---|---|---|
| | Minimum | | 23,60 | |
| | Maximum | | 49,00 | |
| | Range | | 25,40 | |
| | Interquartile Range | | 17,31 | |
| | Skewness | | ,505 | ,845 |
| | Kurtosis | | -1,209 | 1,741 |
| ALB_EST_Perc_Nec_W6 | Mean | | 17,2733 | 4,84170 |
| | 95% Confidence Interval for Mean | Lower Bound | 4,8274 | |
| | | Upper Bound | 29,7193 | |
| | 5% Trimmed Mean | | 17,5104 | |
| | Median | | 21,8650 | |
| | Variance | | 140,652 | |
| | Std. Deviation | | 11,85969 | |
| | Minimum | | ,00 | |
| | Maximum | | 30,28 | |
| | Range | | 30,28 | |
| | Interquartile Range | | 21,59 | |
| | Skewness | | -,716 | ,845 |
| | Kurtosis | | -1,258 | 1,741 |
| ALB_EST_GermCells_W6 | Mean | | ,3553 | ,18232 |
| | 95% Confidence Interval for Mean | Lower Bound | -,1134 | |
| | | Upper Bound | ,8239 | |
| | 5% Trimmed Mean | | ,3303 | |
| | Median | | ,1494 | |
| | Variance | | ,199 | |
| | Std. Deviation | | ,44659 | |
| | Minimum | | ,01 | |
| | Maximum | | 1,15 | |
| | Range | | 1,14 | |
| | Interquartile Range | | ,70 | |
| | Skewness | | 1,467 | ,845 |
| | Kurtosis | | 1,361 | 1,741 |

**Tests of Normality**

| | Kolmogorov-Smirnov[a] | | | Shapiro-Wilk | | |
|---|---|---|---|---|---|---|
| | Statistic | df | Sig. | Statistic | df | Sig. |
| Perc_ST_0 | ,210 | 6 | ,200* | ,920 | 6 | ,507 |
| Perc_Int_0 | ,210 | 6 | ,200* | ,920 | 6 | ,507 |
| GermCells_0 | ,295 | 6 | ,112 | ,743 | 6 | ,017 |
| MEM_Perc_ST_W2 | ,223 | 5 | ,200* | ,961 | 5 | ,817 |
| MEM_Perc_Int_W2 | ,209 | 5 | ,200* | ,924 | 5 | ,557 |
| MEM_Perc_Nec_W2 | ,247 | 5 | ,200* | ,849 | 5 | ,190 |
| MEM_GermCells_W2 | ,261 | 5 | ,200* | ,867 | 5 | ,254 |
| MEM_Perc_ST_W3 | ,168 | 6 | ,200* | ,948 | 6 | ,725 |
| MEM_Perc_Int_W3 | ,179 | 6 | ,200* | ,942 | 6 | ,679 |
| MEM_Perc_Nec_W3 | ,163 | 6 | ,200* | ,932 | 6 | ,598 |
| MEM_GermCells_W3 | ,322 | 6 | ,052 | ,735 | 6 | ,014 |
| MEM_Perc_ST_W4 | ,195 | 6 | ,200* | ,919 | 6 | ,497 |
| MEM_Perc_Int_W4 | ,177 | 6 | ,200* | ,948 | 6 | ,722 |
| MEM_Perc_Nec_W4 | ,148 | 6 | ,200* | ,982 | 6 | ,960 |
| MEM_GermCells_W4 | ,271 | 6 | ,190 | ,822 | 6 | ,091 |
| MEM_Perc_ST_W5 | ,220 | 6 | ,200* | ,951 | 6 | ,747 |
| MEM_Perc_Int_W5 | ,167 | 6 | ,200* | ,975 | 6 | ,927 |
| MEM_Perc_Nec_W5 | ,174 | 6 | ,200* | ,937 | 6 | ,632 |
| MEM_GermCells_W5 | ,274 | 6 | ,181 | ,853 | 6 | ,166 |
| MEM_Perc_ST_W6 | ,167 | 6 | ,200* | ,946 | 6 | ,711 |
| MEM_Perc_Int_W6 | ,186 | 6 | ,200* | ,983 | 6 | ,967 |
| MEM_Perc_Nec_W6 | ,292 | 6 | ,121 | ,893 | 6 | ,336 |
| MEM_GermCells_W6 | ,309 | 6 | ,076 | ,743 | 6 | ,017 |
| KSR_Perc_ST_W2 | ,300 | 5 | ,161 | ,893 | 5 | ,375 |
| KSR_Perc_Int_W2 | ,272 | 5 | ,200* | ,852 | 5 | ,201 |
| KSR_Perc_Nec_W2 | ,200 | 5 | ,200* | ,975 | 5 | ,904 |
| KSR_GermCells_W2 | ,318 | 5 | ,110 | ,838 | 5 | ,159 |
| KSR_Perc_ST_W3 | ,266 | 6 | ,200* | ,821 | 6 | ,091 |
| KSR_Perc_Int_W3 | ,251 | 6 | ,200* | ,849 | 6 | ,156 |
| KSR_Perc_Nec_W3 | ,229 | 6 | ,200* | ,937 | 6 | ,635 |
| KSR_GermCells_W3 | ,331 | 6 | ,039 | ,732 | 6 | ,013 |
| KSR_Perc_ST_W4 | ,232 | 5 | ,200* | ,858 | 5 | ,222 |
| KSR_Perc_Int_W4 | ,168 | 5 | ,200* | ,963 | 5 | ,830 |
| KSR_Perc_Nec_W4 | ,296 | 5 | ,177 | ,818 | 5 | ,113 |
| KSR_GermCells_W4 | ,311 | 5 | ,129 | ,818 | 5 | ,113 |
| KSR_Perc_ST_W5 | ,265 | 6 | ,200* | ,923 | 6 | ,527 |
| KSR_Perc_Int_W5 | ,315 | 6 | ,063 | ,795 | 6 | ,052 |
| KSR_Perc_Nec_W5 | ,296 | 6 | ,109 | ,874 | 6 | ,245 |
| KSR_GermCells_W5 | ,414 | 6 | ,002 | ,617 | 6 | ,001 |
| KSR_Perc_ST_W6 | ,230 | 6 | ,200* | ,885 | 6 | ,293 |
| KSR_Perc_Int_W6 | ,171 | 6 | ,200* | ,987 | 6 | ,981 |
| KSR_Perc_Nec_W6 | ,164 | 6 | ,200* | ,975 | 6 | ,926 |
| KSR_GermCells_W6 | ,431 | 6 | ,001 | ,581 | 6 | ,000 |
| EST_Perc_ST_W2 | ,274 | 4 | . | ,946 | 4 | ,691 |
| EST_Perc_Int_W2 | ,133 | 4 | . | 1,000 | 4 | 1,000 |
| EST_Perc_Nec_W2 | ,199 | 4 | . | ,966 | 4 | ,819 |
| EST_GermCells_W2 | ,255 | 4 | . | ,841 | 4 | ,199 |
| EST_Perc_ST_W3 | ,293 | 6 | ,119 | ,844 | 6 | ,141 |
| EST_Perc_Int_W3 | ,171 | 6 | ,200* | ,979 | 6 | ,947 |

**Tests of Normality**

| | Kolmogorov-Smirnov[a] | | | Shapiro-Wilk | | |
|---|---|---|---|---|---|---|
| | Statistic | df | Sig. | Statistic | df | Sig. |
| EST_Perc_Nec_W3 | ,233 | 6 | ,200* | ,884 | 6 | ,287 |
| EST_GermCells_W3 | ,328 | 6 | ,043 | ,735 | 6 | ,014 |
| EST_Perc_ST_W4 | ,286 | 6 | ,137 | ,811 | 6 | ,073 |
| EST_Perc_Int_W4 | ,199 | 6 | ,200* | ,918 | 6 | ,493 |
| EST_Perc_Nec_W4 | ,295 | 6 | ,111 | ,879 | 6 | ,266 |
| EST_GermCells_W4 | ,227 | 6 | ,200* | ,833 | 6 | ,114 |
| EST_Perc_ST_W5 | ,211 | 6 | ,200* | ,946 | 6 | ,708 |
| EST_Perc_Int_W5 | ,170 | 6 | ,200* | ,932 | 6 | ,595 |
| EST_Perc_Nec_W5 | ,333 | 6 | ,037 | ,764 | 6 | ,027 |
| EST_GermCells_W5 | ,218 | 6 | ,200* | ,928 | 6 | ,568 |
| EST_Perc_ST_W6 | ,295 | 6 | ,112 | ,833 | 6 | ,113 |
| EST_Perc_Int_W6 | ,397 | 6 | ,004 | ,667 | 6 | ,003 |
| EST_Perc_Nec_W6 | ,214 | 6 | ,200* | ,927 | 6 | ,561 |
| EST_GermCells_W6 | ,244 | 6 | ,200* | ,845 | 6 | ,142 |
| ALB_Perc_ST_W2 | ,219 | 5 | ,200* | ,919 | 5 | ,524 |
| ALB_Perc_Int_W2 | ,148 | 5 | ,200* | ,986 | 5 | ,962 |
| ALB_Perc_Nec_W2 | ,205 | 5 | ,200* | ,894 | 5 | ,380 |
| ALB_GermCells_W2 | ,282 | 5 | ,200* | ,845 | 5 | ,181 |
| ALB_Perc_ST_W3 | ,299 | 6 | ,101 | ,877 | 6 | ,254 |
| ALB_Perc_Int_W3 | ,171 | 6 | ,200* | ,984 | 6 | ,971 |
| ALB_Perc_Nec_W3 | ,178 | 6 | ,200* | ,969 | 6 | ,883 |
| ALB_GermCells_W3 | ,383 | 6 | ,006 | ,651 | 6 | ,002 |
| ALB_Perc_ST_W4 | ,188 | 6 | ,200* | ,956 | 6 | ,792 |
| ALB_Perc_Int_W4 | ,186 | 6 | ,200* | ,983 | 6 | ,964 |
| ALB_Perc_Nec_W4 | ,166 | 6 | ,200* | ,958 | 6 | ,807 |
| ALB_GermCells_W4 | ,291 | 6 | ,123 | ,773 | 6 | ,033 |
| ALB_Perc_ST_W5 | ,242 | 6 | ,200* | ,922 | 6 | ,520 |
| ALB_Perc_Int_W5 | ,164 | 6 | ,200* | ,971 | 6 | ,896 |
| ALB_Perc_Nec_W5 | ,316 | 6 | ,062 | ,810 | 6 | ,072 |
| ALB_GermCells_W5 | ,404 | 6 | ,003 | ,587 | 6 | ,000 |
| ALB_Perc_ST_W6 | ,191 | 6 | ,200* | ,960 | 6 | ,820 |
| ALB_Perc_Int_W6 | ,254 | 6 | ,200* | ,888 | 6 | ,308 |
| ALB_Perc_Nec_W6 | ,285 | 6 | ,139 | ,888 | 6 | ,309 |
| ALB_GermCells_W6 | ,313 | 6 | ,068 | ,787 | 6 | ,045 |
| ALB_EST_Perc_ST_W2 | ,236 | 5 | ,200* | ,967 | 5 | ,855 |
| ALB_EST_Perc_Int_W2 | ,210 | 5 | ,200* | ,960 | 5 | ,809 |
| ALB_EST_Perc_Nec_W2 | ,136 | 5 | ,200* | ,988 | 5 | ,971 |
| ALB_EST_GermCells_W2 | ,324 | 5 | ,094 | ,792 | 5 | ,069 |
| ALB_EST_Perc_ST_W3 | ,245 | 6 | ,200* | ,883 | 6 | ,282 |
| ALB_EST_Perc_Int_W3 | ,311 | 6 | ,070 | ,846 | 6 | ,147 |
| ALB_EST_Perc_Nec_W3 | ,244 | 6 | ,200* | ,888 | 6 | ,309 |
| ALB_EST_GermCells_W3 | ,276 | 6 | ,171 | ,792 | 6 | ,050 |
| ALB_EST_Perc_ST_W4 | ,273 | 6 | ,184 | ,854 | 6 | ,170 |
| ALB_EST_Perc_Int_W4 | ,304 | 6 | ,089 | ,779 | 6 | ,038 |
| ALB_EST_Perc_Nec_W4 | ,392 | 6 | ,004 | ,738 | 6 | ,015 |
| ALB_EST_GermCells_W4 | ,319 | 6 | ,057 | ,712 | 6 | ,008 |
| ALB_EST_Perc_ST_W5 | ,195 | 6 | ,200* | ,891 | 6 | ,326 |
| ALB_EST_Perc_Int_W5 | ,171 | 6 | ,200* | ,929 | 6 | ,573 |
| ALB_EST_Perc_Nec_W5 | ,184 | 6 | ,200* | ,943 | 6 | ,685 |

**Tests of Normality**

| | Kolmogorov-Smirnov[a] | | | Shapiro-Wilk | | |
|---|---|---|---|---|---|---|
| | Statistic | df | Sig. | Statistic | df | Sig. |
| ALB_EST_GermCells_W5 | ,252 | 6 | ,200* | ,795 | 6 | ,053 |
| ALB_EST_Perc_ST_W6 | ,309 | 6 | ,075 | ,849 | 6 | ,154 |
| ALB_EST_Perc_Int_W6 | ,226 | 6 | ,200* | ,924 | 6 | ,535 |
| ALB_EST_Perc_Nec_W6 | ,258 | 6 | ,200* | ,892 | 6 | ,327 |
| ALB_EST_GermCells_W6 | ,323 | 6 | ,050 | ,806 | 6 | ,067 |

*. This is a lower bound of the true significance.

a. Lilliefors Significance Correction

```
NPAR TESTS
  /WILCOXON=Perc_ST_0 Perc_ST_0 Perc_ST_0 Perc_ST_0 Perc_ST_0 WITH MEM_Perc_ST_W2 MEM_Perc_ST_
  /MISSING ANALYSIS.
```

## NPar Tests

**Notes**

| | | |
|---|---|---|
| Output Created | | 16-AUG-2017 16:10:21 |
| Comments | | |
| Input | Data | /Users/pmota/Desktop/Organ Culture_treatment and ... |
| | Active Dataset | DataSet1 |
| | Filter | <none> |
| | Weight | <none> |
| | Split File | <none> |
| | N of Rows in Working Data File | 6 |
| Missing Value Handling | Definition of Missing | User-defined missing values are treated as missing. |
| | Cases Used | Statistics for each test are based on all cases with valid data for the variable(s) used in that test. |
| Syntax | | NPAR TESTS /WILCOXON=Perc_ST_0 Perc_ST_0 Perc_ST_0 Perc_ST_0 Perc_ST_0 WITH MEM_Perc_ST_W2 MEM_Perc_ST_W3 MEM_Perc_ST_W4 MEM_Perc_ST_W5 MEM_Perc_ST_W6 (PAIRED) /MISSING ANALYSIS. |
| Resources | Processor Time | 00:00:00,01 |
| | Elapsed Time | 00:00:00,00 |
| | Number of Cases Allowed[a] | 71493 |

a. Based on availability of workspace memory.

```
[DataSet1] /Users/pmota/Desktop/Organ Culture_treatment and week.sav
```

## Wilcoxon Signed Ranks Test

### Ranks

| | | N | Mean Rank | Sum of Ranks |
|---|---|---|---|---|
| MEM_Perc_ST_W2 - Perc_ST_0 | Negative Ranks | 5[a] | 3,00 | 15,00 |
| | Positive Ranks | 0[b] | ,00 | ,00 |
| | Ties | 0[c] | | |
| | Total | 5 | | |
| MEM_Perc_ST_W3 - Perc_ST_0 | Negative Ranks | 6[d] | 3,50 | 21,00 |
| | Positive Ranks | 0[e] | ,00 | ,00 |
| | Ties | 0[f] | | |
| | Total | 6 | | |
| MEM_Perc_ST_W4 - Perc_ST_0 | Negative Ranks | 6[g] | 3,50 | 21,00 |
| | Positive Ranks | 0[h] | ,00 | ,00 |
| | Ties | 0[i] | | |
| | Total | 6 | | |
| MEM_Perc_ST_W5 - Perc_ST_0 | Negative Ranks | 6[j] | 3,50 | 21,00 |
| | Positive Ranks | 0[k] | ,00 | ,00 |
| | Ties | 0[l] | | |
| | Total | 6 | | |
| MEM_Perc_ST_W6 - Perc_ST_0 | Negative Ranks | 6[m] | 3,50 | 21,00 |
| | Positive Ranks | 0[n] | ,00 | ,00 |
| | Ties | 0[o] | | |
| | Total | 6 | | |

a. MEM_Perc_ST_W2 < Perc_ST_0

b. MEM_Perc_ST_W2 > Perc_ST_0

c. MEM_Perc_ST_W2 = Perc_ST_0

d. MEM_Perc_ST_W3 < Perc_ST_0

e. MEM_Perc_ST_W3 > Perc_ST_0

f. MEM_Perc_ST_W3 = Perc_ST_0

g. MEM_Perc_ST_W4 < Perc_ST_0

h. MEM_Perc_ST_W4 > Perc_ST_0

i. MEM_Perc_ST_W4 = Perc_ST_0

j. MEM_Perc_ST_W5 < Perc_ST_0

k. MEM_Perc_ST_W5 > Perc_ST_0

l. MEM_Perc_ST_W5 = Perc_ST_0

m. MEM_Perc_ST_W6 < Perc_ST_0

n. MEM_Perc_ST_W6 > Perc_ST_0

o. MEM_Perc_ST_W6 = Perc_ST_0

### Test Statistics[a]

| | MEM_Perc_ST_W2 - Perc_ST_0 | MEM_Perc_ST_W3 - Perc_ST_0 | MEM_Perc_ST_W4 - Perc_ST_0 | MEM_Perc_ST_W5 - Perc_ST_0 | MEM_Perc_ST_W6 - Perc_ST_0 |
|---|---|---|---|---|---|
| Z | -2,023[b] | -2,201[b] | -2,201[b] | -2,201[b] | -2,201[b] |
| Asymp. Sig. (2-tailed) | ,043 | ,028 | ,028 | ,028 | ,028 |

a. Wilcoxon Signed Ranks Test

b. Based on positive ranks.

```
NPAR TESTS
 /WILCOXON=Perc_ST_0 Perc_ST_0 Perc_ST_0 Perc_ST_0 Perc_ST_0 WITH KSR_Perc_ST_W2 KSR_Perc_ST_W
 /MISSING ANALYSIS.
```

# NPar Tests

**Notes**

| | | |
|---|---|---|
| Output Created | | 16-AUG-2017 16:11:09 |
| Comments | | |
| Input | Data | /Users/pmota/Desktop/Organ Culture_treatment and ... |
| | Active Dataset | DataSet1 |
| | Filter | <none> |
| | Weight | <none> |
| | Split File | <none> |
| | N of Rows in Working Data File | 6 |
| Missing Value Handling | Definition of Missing | User-defined missing values are treated as missing. |
| | Cases Used | Statistics for each test are based on all cases with valid data for the variable(s) used in that test. |
| Syntax | | NPAR TESTS /WILCOXON=Perc_ST_0 Perc_ST_0 Perc_ST_0 Perc_ST_0 Perc_ST_0 WITH KSR_Perc_ST_W2 KSR_Perc_ST_W3 KSR_Perc_ST_W4 KSR_Perc_ST_W5 KSR_Perc_ST_W6 (PAIRED) /MISSING ANALYSIS. |
| Resources | Processor Time | 00:00:00,01 |
| | Elapsed Time | 00:00:00,00 |
| | Number of Cases Allowed[a] | 71493 |

a. Based on availability of workspace memory.

```
[DataSet1] /Users/pmota/Desktop/Organ Culture_treatment and week.sav
```

# Wilcoxon Signed Ranks Test

### Ranks

| | | N | Mean Rank | Sum of Ranks |
|---|---|---|---|---|
| KSR_Perc_ST_W2 - Perc_ST_0 | Negative Ranks | 5[a] | 3,00 | 15,00 |
| | Positive Ranks | 0[b] | ,00 | ,00 |
| | Ties | 0[c] | | |
| | Total | 5 | | |
| KSR_Perc_ST_W3 - Perc_ST_0 | Negative Ranks | 6[d] | 3,50 | 21,00 |
| | Positive Ranks | 0[e] | ,00 | ,00 |
| | Ties | 0[f] | | |
| | Total | 6 | | |
| KSR_Perc_ST_W4 - Perc_ST_0 | Negative Ranks | 5[g] | 3,00 | 15,00 |
| | Positive Ranks | 0[h] | ,00 | ,00 |
| | Ties | 0[i] | | |
| | Total | 5 | | |
| KSR_Perc_ST_W5 - Perc_ST_0 | Negative Ranks | 6[j] | 3,50 | 21,00 |
| | Positive Ranks | 0[k] | ,00 | ,00 |
| | Ties | 0[l] | | |
| | Total | 6 | | |
| KSR_Perc_ST_W6 - Perc_ST_0 | Negative Ranks | 6[m] | 3,50 | 21,00 |
| | Positive Ranks | 0[n] | ,00 | ,00 |
| | Ties | 0[o] | | |
| | Total | 6 | | |

a. KSR_Perc_ST_W2 < Perc_ST_0

b. KSR_Perc_ST_W2 > Perc_ST_0

c. KSR_Perc_ST_W2 = Perc_ST_0

d. KSR_Perc_ST_W3 < Perc_ST_0

e. KSR_Perc_ST_W3 > Perc_ST_0

f. KSR_Perc_ST_W3 = Perc_ST_0

g. KSR_Perc_ST_W4 < Perc_ST_0

h. KSR_Perc_ST_W4 > Perc_ST_0

i. KSR_Perc_ST_W4 = Perc_ST_0

j. KSR_Perc_ST_W5 < Perc_ST_0

k. KSR_Perc_ST_W5 > Perc_ST_0

l. KSR_Perc_ST_W5 = Perc_ST_0

m. KSR_Perc_ST_W6 < Perc_ST_0

n. KSR_Perc_ST_W6 > Perc_ST_0

o. KSR_Perc_ST_W6 = Perc_ST_0

### Test Statistics[a]

| | KSR_Perc_ST_W2 - Perc_ST_0 | KSR_Perc_ST_W3 - Perc_ST_0 | KSR_Perc_ST_W4 - Perc_ST_0 | KSR_Perc_ST_W5 - Perc_ST_0 | KSR_Perc_ST_W6 - Perc_ST_0 |
|---|---|---|---|---|---|
| Z | -2,023[b] | -2,201[b] | -2,023[b] | -2,201[b] | -2,201[b] |
| Asymp. Sig. (2-tailed) | ,043 | ,028 | ,043 | ,028 | ,028 |

a. Wilcoxon Signed Ranks Test

b. Based on positive ranks.

```
NPAR TESTS
 /WILCOXON=Perc_ST_0 Perc_ST_0 Perc_ST_0 Perc_ST_0 Perc_ST_0 WITH EST_Perc_ST_W2 EST_Perc_ST_W
 /MISSING ANALYSIS.
```

# NPar Tests

**Notes**

| | | |
|---|---|---|
| Output Created | | 16-AUG-2017 16:11:44 |
| Comments | | |
| Input | Data | /Users/pmota/Desktop/Organ Culture_treatment and ... |
| | Active Dataset | DataSet1 |
| | Filter | <none> |
| | Weight | <none> |
| | Split File | <none> |
| | N of Rows in Working Data File | 6 |
| Missing Value Handling | Definition of Missing | User-defined missing values are treated as missing. |
| | Cases Used | Statistics for each test are based on all cases with valid data for the variable(s) used in that test. |
| Syntax | | NPAR TESTS /WILCOXON=Perc_ST_0 Perc_ST_0 Perc_ST_0 Perc_ST_0 Perc_ST_0 WITH EST_Perc_ST_W2 EST_Perc_ST_W3 EST_Perc_ST_W4 EST_Perc_ST_W5 EST_Perc_ST_W6 (PAIRED) /MISSING ANALYSIS. |
| Resources | Processor Time | 00:00:00,01 |
| | Elapsed Time | 00:00:00,00 |
| | Number of Cases Allowed[a] | 71493 |

a. Based on availability of workspace memory.

```
[DataSet1] /Users/pmota/Desktop/Organ Culture_treatment and week.sav
```

# Wilcoxon Signed Ranks Test

## Ranks

| | | N | Mean Rank | Sum of Ranks |
|---|---|---|---|---|
| EST_Perc_ST_W2 - Perc_ST_0 | Negative Ranks | 4[a] | 2,50 | 10,00 |
| | Positive Ranks | 0[b] | ,00 | ,00 |
| | Ties | 0[c] | | |
| | Total | 4 | | |
| EST_Perc_ST_W3 - Perc_ST_0 | Negative Ranks | 6[d] | 3,50 | 21,00 |
| | Positive Ranks | 0[e] | ,00 | ,00 |
| | Ties | 0[f] | | |
| | Total | 6 | | |
| EST_Perc_ST_W4 - Perc_ST_0 | Negative Ranks | 6[g] | 3,50 | 21,00 |
| | Positive Ranks | 0[h] | ,00 | ,00 |
| | Ties | 0[i] | | |
| | Total | 6 | | |
| EST_Perc_ST_W5 - Perc_ST_0 | Negative Ranks | 6[j] | 3,50 | 21,00 |
| | Positive Ranks | 0[k] | ,00 | ,00 |
| | Ties | 0[l] | | |
| | Total | 6 | | |
| EST_Perc_ST_W6 - Perc_ST_0 | Negative Ranks | 6[m] | 3,50 | 21,00 |
| | Positive Ranks | 0[n] | ,00 | ,00 |
| | Ties | 0[o] | | |
| | Total | 6 | | |

a. EST_Perc_ST_W2 < Perc_ST_0

b. EST_Perc_ST_W2 > Perc_ST_0

c. EST_Perc_ST_W2 = Perc_ST_0

d. EST_Perc_ST_W3 < Perc_ST_0

e. EST_Perc_ST_W3 > Perc_ST_0

f. EST_Perc_ST_W3 = Perc_ST_0

g. EST_Perc_ST_W4 < Perc_ST_0

h. EST_Perc_ST_W4 > Perc_ST_0

i. EST_Perc_ST_W4 = Perc_ST_0

j. EST_Perc_ST_W5 < Perc_ST_0

k. EST_Perc_ST_W5 > Perc_ST_0

l. EST_Perc_ST_W5 = Perc_ST_0

m. EST_Perc_ST_W6 < Perc_ST_0

n. EST_Perc_ST_W6 > Perc_ST_0

o. EST_Perc_ST_W6 = Perc_ST_0

## Test Statistics[a]

| | EST_Perc_ST_W2 - Perc_ST_0 | EST_Perc_ST_W3 - Perc_ST_0 | EST_Perc_ST_W4 - Perc_ST_0 | EST_Perc_ST_W5 - Perc_ST_0 | EST_Perc_ST_W6 - Perc_ST_0 |
|---|---|---|---|---|---|
| Z | -1,826[b] | -2,201[b] | -2,201[b] | -2,201[b] | -2,201[b] |
| Asymp. Sig. (2-tailed) | ,068 | ,028 | ,028 | ,028 | ,028 |

a. Wilcoxon Signed Ranks Test

b. Based on positive ranks.

```
NPAR TESTS
 /WILCOXON=Perc_ST_0 Perc_ST_0 Perc_ST_0 Perc_ST_0 Perc_ST_0 WITH ALB_Perc_ST_W2 ALB_Perc_ST_W
 /MISSING ANALYSIS.
```

# NPar Tests

**Notes**

| | | |
|---|---|---|
| Output Created | | 16-AUG-2017 16:12:25 |
| Comments | | |
| Input | Data | /Users/pmota/Desktop/Organ Culture_treatment and ... |
| | Active Dataset | DataSet1 |
| | Filter | <none> |
| | Weight | <none> |
| | Split File | <none> |
| | N of Rows in Working Data File | 6 |
| Missing Value Handling | Definition of Missing | User-defined missing values are treated as missing. |
| | Cases Used | Statistics for each test are based on all cases with valid data for the variable(s) used in that test. |
| Syntax | | NPAR TESTS<br><br>/WILCOXON=Perc_ST_0 Perc_ST_0 Perc_ST_0 Perc_ST_0 Perc_ST_0 WITH ALB_Perc_ST_W2 ALB_Perc_ST_W3 ALB_Perc_ST_W4 ALB_Perc_ST_W5 ALB_Perc_ST_W6 (PAIRED)<br>/MISSING ANALYSIS. |
| Resources | Processor Time | 00:00:00,01 |
| | Elapsed Time | 00:00:00,00 |
| | Number of Cases Allowed[a] | 71493 |

a. Based on availability of workspace memory.

```
[DataSet1] /Users/pmota/Desktop/Organ Culture_treatment and week.sav
```

# Wilcoxon Signed Ranks Test

**Ranks**

| | | N | Mean Rank | Sum of Ranks |
|---|---|---|---|---|
| ALB_Perc_ST_W2 - Perc_ST_0 | Negative Ranks | 5[a] | 3,00 | 15,00 |
| | Positive Ranks | 0[b] | ,00 | ,00 |
| | Ties | 0[c] | | |
| | Total | 5 | | |
| ALB_Perc_ST_W3 - Perc_ST_0 | Negative Ranks | 6[d] | 3,50 | 21,00 |
| | Positive Ranks | 0[e] | ,00 | ,00 |
| | Ties | 0[f] | | |
| | Total | 6 | | |
| ALB_Perc_ST_W4 - Perc_ST_0 | Negative Ranks | 6[g] | 3,50 | 21,00 |
| | Positive Ranks | 0[h] | ,00 | ,00 |
| | Ties | 0[i] | | |
| | Total | 6 | | |
| ALB_Perc_ST_W5 - Perc_ST_0 | Negative Ranks | 6[j] | 3,50 | 21,00 |
| | Positive Ranks | 0[k] | ,00 | ,00 |
| | Ties | 0[l] | | |
| | Total | 6 | | |
| ALB_Perc_ST_W6 - Perc_ST_0 | Negative Ranks | 6[m] | 3,50 | 21,00 |
| | Positive Ranks | 0[n] | ,00 | ,00 |
| | Ties | 0[o] | | |
| | Total | 6 | | |

a. ALB_Perc_ST_W2 < Perc_ST_0

b. ALB_Perc_ST_W2 > Perc_ST_0

c. ALB_Perc_ST_W2 = Perc_ST_0

d. ALB_Perc_ST_W3 < Perc_ST_0

e. ALB_Perc_ST_W3 > Perc_ST_0

f. ALB_Perc_ST_W3 = Perc_ST_0

g. ALB_Perc_ST_W4 < Perc_ST_0

h. ALB_Perc_ST_W4 > Perc_ST_0

i. ALB_Perc_ST_W4 = Perc_ST_0

j. ALB_Perc_ST_W5 < Perc_ST_0

k. ALB_Perc_ST_W5 > Perc_ST_0

l. ALB_Perc_ST_W5 = Perc_ST_0

m. ALB_Perc_ST_W6 < Perc_ST_0

n. ALB_Perc_ST_W6 > Perc_ST_0

o. ALB_Perc_ST_W6 = Perc_ST_0

**Test Statistics[a]**

| | ALB_Perc_ST_W2 - Perc_ST_0 | ALB_Perc_ST_W3 - Perc_ST_0 | ALB_Perc_ST_W4 - Perc_ST_0 | ALB_Perc_ST_W5 - Perc_ST_0 | ALB_Perc_ST_W6 - Perc_ST_0 |
|---|---|---|---|---|---|
| Z | -2,023[b] | -2,201[b] | -2,201[b] | -2,201[b] | -2,201[b] |
| Asymp. Sig. (2-tailed) | ,043 | ,028 | ,028 | ,028 | ,028 |

a. Wilcoxon Signed Ranks Test

b. Based on positive ranks.

```
NPAR TESTS
 /WILCOXON=Perc_ST_0 Perc_ST_0 Perc_ST_0 Perc_ST_0 Perc_ST_0 WITH ALB_EST_Perc_ST_W2 ALB_EST_
 /MISSING ANALYSIS.
```

# NPar Tests

**Notes**

| | | |
|---|---|---|
| Output Created | | 16-AUG-2017 16:13:04 |
| Comments | | |
| Input | Data | /Users/pmota/Desktop/Organ Culture_treatment and ... |
| | Active Dataset | DataSet1 |
| | Filter | <none> |
| | Weight | <none> |
| | Split File | <none> |
| | N of Rows in Working Data File | 6 |
| Missing Value Handling | Definition of Missing | User-defined missing values are treated as missing. |
| | Cases Used | Statistics for each test are based on all cases with valid data for the variable(s) used in that test. |
| Syntax | | NPAR TESTS /WILCOXON=Perc_ST_0 Perc_ST_0 Perc_ST_0 Perc_ST_0 Perc_ST_0 WITH ALB_EST_Perc_ST_W2 ALB_EST_Perc_ST_W3 ALB_EST_Perc_ST_W4 ALB_EST_Perc_ST_W5 ALB_EST_Perc_ST_W6 (PAIRED) /MISSING ANALYSIS. |
| Resources | Processor Time | 00:00:00,01 |
| | Elapsed Time | 00:00:00,00 |
| | Number of Cases Allowed[a] | 71493 |

a. Based on availability of workspace memory.

```
[DataSet1] /Users/pmota/Desktop/Organ Culture_treatment and week.sav
```

# Wilcoxon Signed Ranks Test

### Ranks

| | | N | Mean Rank | Sum of Ranks |
|---|---|---|---|---|
| ALB_EST_Perc_ST_W2 - Perc_ST_0 | Negative Ranks | 5[a] | 3,00 | 15,00 |
| | Positive Ranks | 0[b] | ,00 | ,00 |
| | Ties | 0[c] | | |
| | Total | 5 | | |
| ALB_EST_Perc_ST_W3 - Perc_ST_0 | Negative Ranks | 6[d] | 3,50 | 21,00 |
| | Positive Ranks | 0[e] | ,00 | ,00 |
| | Ties | 0[f] | | |
| | Total | 6 | | |
| ALB_EST_Perc_ST_W4 - Perc_ST_0 | Negative Ranks | 6[g] | 3,50 | 21,00 |
| | Positive Ranks | 0[h] | ,00 | ,00 |
| | Ties | 0[i] | | |
| | Total | 6 | | |
| ALB_EST_Perc_ST_W5 - Perc_ST_0 | Negative Ranks | 6[j] | 3,50 | 21,00 |
| | Positive Ranks | 0[k] | ,00 | ,00 |
| | Ties | 0[l] | | |
| | Total | 6 | | |
| ALB_EST_Perc_ST_W6 - Perc_ST_0 | Negative Ranks | 6[m] | 3,50 | 21,00 |
| | Positive Ranks | 0[n] | ,00 | ,00 |
| | Ties | 0[o] | | |
| | Total | 6 | | |

a. ALB_EST_Perc_ST_W2 < Perc_ST_0

b. ALB_EST_Perc_ST_W2 > Perc_ST_0

c. ALB_EST_Perc_ST_W2 = Perc_ST_0

d. ALB_EST_Perc_ST_W3 < Perc_ST_0

e. ALB_EST_Perc_ST_W3 > Perc_ST_0

f. ALB_EST_Perc_ST_W3 = Perc_ST_0

g. ALB_EST_Perc_ST_W4 < Perc_ST_0

h. ALB_EST_Perc_ST_W4 > Perc_ST_0

i. ALB_EST_Perc_ST_W4 = Perc_ST_0

j. ALB_EST_Perc_ST_W5 < Perc_ST_0

k. ALB_EST_Perc_ST_W5 > Perc_ST_0

l. ALB_EST_Perc_ST_W5 = Perc_ST_0

m. ALB_EST_Perc_ST_W6 < Perc_ST_0

n. ALB_EST_Perc_ST_W6 > Perc_ST_0

o. ALB_EST_Perc_ST_W6 = Perc_ST_0

### Test Statistics[a]

| | ALB_EST_Perc_ST_W2 - Perc_ST_0 | ALB_EST_Perc_ST_W3 - Perc_ST_0 | ALB_EST_Perc_ST_W4 - Perc_ST_0 | ALB_EST_Perc_ST_W5 - Perc_ST_0 | ALB_EST_Perc_ST_W6 - Perc_ST_0 |
|---|---|---|---|---|---|
| Z | -2,023[b] | -2,201[b] | -2,201[b] | -2,201[b] | -2,201[b] |
| Asymp. Sig. (2-tailed) | ,043 | ,028 | ,028 | ,028 | ,028 |

a. Wilcoxon Signed Ranks Test

b. Based on positive ranks.

```
NPAR TESTS
 /WILCOXON=MEM_Perc_ST_W2 MEM_Perc_ST_W3 MEM_Perc_ST_W4 MEM_Perc_ST_W5 MEM_Perc_ST_W6 WITH KS
 /MISSING ANALYSIS.
```

# NPar Tests

**Notes**

| | | |
|---|---|---|
| Output Created | | 16-AUG-2017 16:29:49 |
| Comments | | |
| Input | Data | /Users/pmota/Desktop/ Organ Culture_treatment and ... |
| | Active Dataset | DataSet1 |
| | Filter | <none> |
| | Weight | <none> |
| | Split File | <none> |
| | N of Rows in Working Data File | 6 |
| Missing Value Handling | Definition of Missing | User-defined missing values are treated as missing. |
| | Cases Used | Statistics for each test are based on all cases with valid data for the variable(s) used in that test. |
| Syntax | | NPAR TESTS /WILCOXON=MEM_Perc_ST_W2 MEM_Perc_ST_W3 MEM_Perc_ST_W4 MEM_Perc_ST_W5 MEM_Perc_ST_W6 WITH KSR_Perc_ST_W2 KSR_Perc_ST_W3 KSR_Perc_ST_W4 KSR_Perc_ST_W5 KSR_Perc_ST_W6 (PAIRED) /MISSING ANALYSIS. |
| Resources | Processor Time | 00:00:00,01 |
| | Elapsed Time | 00:00:00,00 |
| | Number of Cases Allowed[a] | 52428 |

a. Based on availability of workspace memory.

[DataSet1] /Users/pmota/Desktop/Organ Culture_treatment and week.sav

# Wilcoxon Signed Ranks Test

**Ranks**

| | | N | Mean Rank | Sum of Ranks |
|---|---|---|---|---|
| KSR_Perc_ST_W2 - MEM_Perc_ST_W2 | Negative Ranks | 2[a] | 2,50 | 5,00 |
| | Positive Ranks | 3[b] | 3,33 | 10,00 |
| | Ties | 0[c] | | |
| | Total | 5 | | |
| KSR_Perc_ST_W3 - MEM_Perc_ST_W3 | Negative Ranks | 2[d] | 4,00 | 8,00 |
| | Positive Ranks | 4[e] | 3,25 | 13,00 |
| | Ties | 0[f] | | |
| | Total | 6 | | |
| KSR_Perc_ST_W4 - MEM_Perc_ST_W4 | Negative Ranks | 3[g] | 3,67 | 11,00 |
| | Positive Ranks | 2[h] | 2,00 | 4,00 |
| | Ties | 0[i] | | |
| | Total | 5 | | |
| KSR_Perc_ST_W5 - MEM_Perc_ST_W5 | Negative Ranks | 3[j] | 2,67 | 8,00 |
| | Positive Ranks | 3[k] | 4,33 | 13,00 |
| | Ties | 0[l] | | |
| | Total | 6 | | |
| KSR_Perc_ST_W6 - MEM_Perc_ST_W6 | Negative Ranks | 3[m] | 3,00 | 9,00 |
| | Positive Ranks | 3[n] | 4,00 | 12,00 |
| | Ties | 0[o] | | |
| | Total | 6 | | |

a. KSR_Perc_ST_W2 < MEM_Perc_ST_W2

b. KSR_Perc_ST_W2 > MEM_Perc_ST_W2

c. KSR_Perc_ST_W2 = MEM_Perc_ST_W2

d. KSR_Perc_ST_W3 < MEM_Perc_ST_W3

e. KSR_Perc_ST_W3 > MEM_Perc_ST_W3

f. KSR_Perc_ST_W3 = MEM_Perc_ST_W3

g. KSR_Perc_ST_W4 < MEM_Perc_ST_W4

h. KSR_Perc_ST_W4 > MEM_Perc_ST_W4

i. KSR_Perc_ST_W4 = MEM_Perc_ST_W4

j. KSR_Perc_ST_W5 < MEM_Perc_ST_W5

k. KSR_Perc_ST_W5 > MEM_Perc_ST_W5

l. KSR_Perc_ST_W5 = MEM_Perc_ST_W5

m. KSR_Perc_ST_W6 < MEM_Perc_ST_W6

n. KSR_Perc_ST_W6 > MEM_Perc_ST_W6

o. KSR_Perc_ST_W6 = MEM_Perc_ST_W6

**Test Statistics[a]**

| | KSR_Perc_ST_W2 - MEM_Perc_ST_W2 | KSR_Perc_ST_W3 - MEM_Perc_ST_W3 | KSR_Perc_ST_W4 - MEM_Perc_ST_W4 | KSR_Perc_ST_W5 - MEM_Perc_ST_W5 | KSR_Perc_ST_W6 - MEM_Perc_ST_W6 |
|---|---|---|---|---|---|
| Z | -,674[b] | -,524[b] | -,944[c] | -,524[b] | -,314[b] |
| Asymp. Sig. (2-tailed) | ,500 | ,600 | ,345 | ,600 | ,753 |

a. Wilcoxon Signed Ranks Test

b. Based on negative ranks.

c. Based on positive ranks.

```
NPAR TESTS
 /WILCOXON=MEM_Perc_ST_W2 MEM_Perc_ST_W3 MEM_Perc_ST_W4 MEM_Perc_ST_W5 MEM_Perc_ST_W6 WITH EST
```

```
/MISSING ANALYSIS.
```

# NPar Tests

**Notes**

| | | |
|---|---|---|
| Output Created | | 16-AUG-2017 16:30:48 |
| Comments | | |
| Input | Data | /Users/pmota/Desktop/Organ Culture_treatment and ... |
| | Active Dataset | DataSet1 |
| | Filter | <none> |
| | Weight | <none> |
| | Split File | <none> |
| | N of Rows in Working Data File | 6 |
| Missing Value Handling | Definition of Missing | User-defined missing values are treated as missing. |
| | Cases Used | Statistics for each test are based on all cases with valid data for the variable(s) used in that test. |
| Syntax | | NPAR TESTS /WILCOXON=MEM_Perc_ST_W2 MEM_Perc_ST_W3 MEM_Perc_ST_W4 MEM_Perc_ST_W5 MEM_Perc_ST_W6 WITH EST_Perc_ST_W2 EST_Perc_ST_W3 EST_Perc_ST_W4 EST_Perc_ST_W5 EST_Perc_ST_W6 (PAIRED) /MISSING ANALYSIS. |
| Resources | Processor Time | 00:00:00,01 |
| | Elapsed Time | 00:00:00,00 |
| | Number of Cases Allowed[a] | 52428 |

a. Based on availability of workspace memory.

```
[DataSet1] /Users/pmota/Desktop/Organ Culture_treatment and week.sav
```

# Wilcoxon Signed Ranks Test

**Ranks**

| | | N | Mean Rank | Sum of Ranks |
|---|---|---|---|---|
| EST_Perc_ST_W2 - MEM_Perc_ST_W2 | Negative Ranks | 3[a] | 2,33 | 7,00 |
| | Positive Ranks | 1[b] | 3,00 | 3,00 |
| | Ties | 0[c] | | |
| | Total | 4 | | |
| EST_Perc_ST_W3 - MEM_Perc_ST_W3 | Negative Ranks | 2[d] | 5,00 | 10,00 |
| | Positive Ranks | 4[e] | 2,75 | 11,00 |
| | Ties | 0[f] | | |
| | Total | 6 | | |
| EST_Perc_ST_W4 - MEM_Perc_ST_W4 | Negative Ranks | 3[g] | 2,67 | 8,00 |
| | Positive Ranks | 3[h] | 4,33 | 13,00 |
| | Ties | 0[i] | | |
| | Total | 6 | | |
| EST_Perc_ST_W5 - MEM_Perc_ST_W5 | Negative Ranks | 3[j] | 2,67 | 8,00 |
| | Positive Ranks | 3[k] | 4,33 | 13,00 |
| | Ties | 0[l] | | |
| | Total | 6 | | |
| EST_Perc_ST_W6 - MEM_Perc_ST_W6 | Negative Ranks | 3[m] | 4,00 | 12,00 |
| | Positive Ranks | 3[n] | 3,00 | 9,00 |
| | Ties | 0[o] | | |
| | Total | 6 | | |

a. EST_Perc_ST_W2 < MEM_Perc_ST_W2

b. EST_Perc_ST_W2 > MEM_Perc_ST_W2

c. EST_Perc_ST_W2 = MEM_Perc_ST_W2

d. EST_Perc_ST_W3 < MEM_Perc_ST_W3

e. EST_Perc_ST_W3 > MEM_Perc_ST_W3

f. EST_Perc_ST_W3 = MEM_Perc_ST_W3

g. EST_Perc_ST_W4 < MEM_Perc_ST_W4

h. EST_Perc_ST_W4 > MEM_Perc_ST_W4

i. EST_Perc_ST_W4 = MEM_Perc_ST_W4

j. EST_Perc_ST_W5 < MEM_Perc_ST_W5

k. EST_Perc_ST_W5 > MEM_Perc_ST_W5

l. EST_Perc_ST_W5 = MEM_Perc_ST_W5

m. EST_Perc_ST_W6 < MEM_Perc_ST_W6

n. EST_Perc_ST_W6 > MEM_Perc_ST_W6

o. EST_Perc_ST_W6 = MEM_Perc_ST_W6

**Test Statistics[a]**

| | EST_Perc_ST_W2 - MEM_Perc_ST_W2 | EST_Perc_ST_W3 - MEM_Perc_ST_W3 | EST_Perc_ST_W4 - MEM_Perc_ST_W4 | EST_Perc_ST_W5 - MEM_Perc_ST_W5 | EST_Perc_ST_W6 - MEM_Perc_ST_W6 |
|---|---|---|---|---|---|
| Z | -,730[b] | -,105[c] | -,524[c] | -,524[c] | -,314[b] |
| Asymp. Sig. (2-tailed) | ,465 | ,917 | ,600 | ,600 | ,753 |

a. Wilcoxon Signed Ranks Test

b. Based on positive ranks.

c. Based on negative ranks.

```
NPAR TESTS
 /WILCOXON=MEM_Perc_ST_W2 MEM_Perc_ST_W3 MEM_Perc_ST_W4 MEM_Perc_ST_W5 MEM_Perc_ST_W6 WITH AL
```

```
  /MISSING ANALYSIS.
```

## NPar Tests

**Notes**

| | | |
|---|---|---|
| Output Created | | 16-AUG-2017 16:31:25 |
| Comments | | |
| Input | Data | /Users/pmota/Desktop/Organ Culture_treatment and ... |
| | Active Dataset | DataSet1 |
| | Filter | <none> |
| | Weight | <none> |
| | Split File | <none> |
| | N of Rows in Working Data File | 6 |
| Missing Value Handling | Definition of Missing | User-defined missing values are treated as missing. |
| | Cases Used | Statistics for each test are based on all cases with valid data for the variable(s) used in that test. |
| Syntax | | NPAR TESTS /WILCOXON=MEM_Perc_ST_W2 MEM_Perc_ST_W3 MEM_Perc_ST_W4 MEM_Perc_ST_W5 MEM_Perc_ST_W6 WITH ALB_Perc_ST_W2 ALB_Perc_ST_W3 ALB_Perc_ST_W4 ALB_Perc_ST_W5 ALB_Perc_ST_W6 (PAIRED) /MISSING ANALYSIS. |
| Resources | Processor Time | 00:00:00,01 |
| | Elapsed Time | 00:00:00,00 |
| | Number of Cases Allowed[a] | 52428 |

a. Based on availability of workspace memory.

```
[DataSet1] /Users/pmota/Desktop/Organ Culture_treatment and week.sav
```

## Wilcoxon Signed Ranks Test

**Ranks**

| | | N | Mean Rank | Sum of Ranks |
|---|---|---|---|---|
| ALB_Perc_ST_W2 - MEM_Perc_ST_W2 | Negative Ranks | 2[a] | 3,00 | 6,00 |
| | Positive Ranks | 3[b] | 3,00 | 9,00 |
| | Ties | 0[c] | | |
| | Total | 5 | | |
| ALB_Perc_ST_W3 - MEM_Perc_ST_W3 | Negative Ranks | 4[d] | 3,75 | 15,00 |
| | Positive Ranks | 2[e] | 3,00 | 6,00 |
| | Ties | 0[f] | | |
| | Total | 6 | | |
| ALB_Perc_ST_W4 - MEM_Perc_ST_W4 | Negative Ranks | 4[g] | 3,50 | 14,00 |
| | Positive Ranks | 2[h] | 3,50 | 7,00 |
| | Ties | 0[i] | | |
| | Total | 6 | | |
| ALB_Perc_ST_W5 - MEM_Perc_ST_W5 | Negative Ranks | 3[j] | 3,00 | 9,00 |
| | Positive Ranks | 3[k] | 4,00 | 12,00 |
| | Ties | 0[l] | | |
| | Total | 6 | | |
| ALB_Perc_ST_W6 - MEM_Perc_ST_W6 | Negative Ranks | 1[m] | 2,00 | 2,00 |
| | Positive Ranks | 5[n] | 3,80 | 19,00 |
| | Ties | 0[o] | | |
| | Total | 6 | | |

a. ALB_Perc_ST_W2 < MEM_Perc_ST_W2

b. ALB_Perc_ST_W2 > MEM_Perc_ST_W2

c. ALB_Perc_ST_W2 = MEM_Perc_ST_W2

d. ALB_Perc_ST_W3 < MEM_Perc_ST_W3

e. ALB_Perc_ST_W3 > MEM_Perc_ST_W3

f. ALB_Perc_ST_W3 = MEM_Perc_ST_W3

g. ALB_Perc_ST_W4 < MEM_Perc_ST_W4

h. ALB_Perc_ST_W4 > MEM_Perc_ST_W4

i. ALB_Perc_ST_W4 = MEM_Perc_ST_W4

j. ALB_Perc_ST_W5 < MEM_Perc_ST_W5

k. ALB_Perc_ST_W5 > MEM_Perc_ST_W5

l. ALB_Perc_ST_W5 = MEM_Perc_ST_W5

m. ALB_Perc_ST_W6 < MEM_Perc_ST_W6

n. ALB_Perc_ST_W6 > MEM_Perc_ST_W6

o. ALB_Perc_ST_W6 = MEM_Perc_ST_W6

**Test Statistics[a]**

| | ALB_Perc_ST_W2 - MEM_Perc_ST_W2 | ALB_Perc_ST_W3 - MEM_Perc_ST_W3 | ALB_Perc_ST_W4 - MEM_Perc_ST_W4 | ALB_Perc_ST_W5 - MEM_Perc_ST_W5 | ALB_Perc_ST_W6 - MEM_Perc_ST_W6 |
|---|---|---|---|---|---|
| Z | -,405[b] | -,943[c] | -,734[c] | -,314[b] | -1,782[b] |
| Asymp. Sig. (2-tailed) | ,686 | ,345 | ,463 | ,753 | ,075 |

a. Wilcoxon Signed Ranks Test

b. Based on negative ranks.

c. Based on positive ranks.

```
NPAR TESTS
 /WILCOXON=MEM_Perc_ST_W2 MEM_Perc_ST_W3 MEM_Perc_ST_W4 MEM_Perc_ST_W5 MEM_Perc_ST_W6 WITH AL[illegible]
```

```
/MISSING ANALYSIS.
```

## NPar Tests

**Notes**

| | | |
|---|---|---|
| Output Created | | 16-AUG-2017 16:32:03 |
| Comments | | |
| Input | Data | /Users/pmota/Desktop/Organ Culture_treatment and ... |
| | Active Dataset | DataSet1 |
| | Filter | <none> |
| | Weight | <none> |
| | Split File | <none> |
| | N of Rows in Working Data File | 6 |
| Missing Value Handling | Definition of Missing | User-defined missing values are treated as missing. |
| | Cases Used | Statistics for each test are based on all cases with valid data for the variable(s) used in that test. |
| Syntax | | NPAR TESTS<br><br>/WILCOXON=MEM_Perc_ST_W2 MEM_Perc_ST_W3 MEM_Perc_ST_W4 MEM_Perc_ST_W5 MEM_Perc_ST_W6 WITH ALB_EST_Perc_ST_W2 ALB_EST_Perc_ST_W3 ALB_EST_Perc_ST_W4 ALB_EST_Perc_ST_W5 ALB_EST_Perc_ST_W6 (PAIRED)<br>/MISSING ANALYSIS. |
| Resources | Processor Time | 00:00:00,01 |
| | Elapsed Time | 00:00:00,00 |
| | Number of Cases Allowed[a] | 52428 |

a. Based on availability of workspace memory.

```
[DataSet1] /Users/pmota/Desktop/Organ Culture_treatment and week.sav
```

## Wilcoxon Signed Ranks Test

### Ranks

| | | N | Mean Rank | Sum of Ranks |
|---|---|---|---|---|
| ALB_EST_Perc_ST_W2 - MEM_Perc_ST_W2 | Negative Ranks | 3[a] | 3,33 | 10,00 |
| | Positive Ranks | 2[b] | 2,50 | 5,00 |
| | Ties | 0[c] | | |
| | Total | 5 | | |
| ALB_EST_Perc_ST_W3 - MEM_Perc_ST_W3 | Negative Ranks | 3[d] | 2,67 | 8,00 |
| | Positive Ranks | 3[e] | 4,33 | 13,00 |
| | Ties | 0[f] | | |
| | Total | 6 | | |
| ALB_EST_Perc_ST_W4 - MEM_Perc_ST_W4 | Negative Ranks | 5[g] | 3,80 | 19,00 |
| | Positive Ranks | 1[h] | 2,00 | 2,00 |
| | Ties | 0[i] | | |
| | Total | 6 | | |
| ALB_EST_Perc_ST_W5 - MEM_Perc_ST_W5 | Negative Ranks | 3[j] | 3,67 | 11,00 |
| | Positive Ranks | 3[k] | 3,33 | 10,00 |
| | Ties | 0[l] | | |
| | Total | 6 | | |
| ALB_EST_Perc_ST_W6 - MEM_Perc_ST_W6 | Negative Ranks | 1[m] | 1,00 | 1,00 |
| | Positive Ranks | 5[n] | 4,00 | 20,00 |
| | Ties | 0[o] | | |
| | Total | 6 | | |

a. ALB_EST_Perc_ST_W2 < MEM_Perc_ST_W2

b. ALB_EST_Perc_ST_W2 > MEM_Perc_ST_W2

c. ALB_EST_Perc_ST_W2 = MEM_Perc_ST_W2

d. ALB_EST_Perc_ST_W3 < MEM_Perc_ST_W3

e. ALB_EST_Perc_ST_W3 > MEM_Perc_ST_W3

f. ALB_EST_Perc_ST_W3 = MEM_Perc_ST_W3

g. ALB_EST_Perc_ST_W4 < MEM_Perc_ST_W4

h. ALB_EST_Perc_ST_W4 > MEM_Perc_ST_W4

i. ALB_EST_Perc_ST_W4 = MEM_Perc_ST_W4

j. ALB_EST_Perc_ST_W5 < MEM_Perc_ST_W5

k. ALB_EST_Perc_ST_W5 > MEM_Perc_ST_W5

l. ALB_EST_Perc_ST_W5 = MEM_Perc_ST_W5

m. ALB_EST_Perc_ST_W6 < MEM_Perc_ST_W6

n. ALB_EST_Perc_ST_W6 > MEM_Perc_ST_W6

o. ALB_EST_Perc_ST_W6 = MEM_Perc_ST_W6

### Test Statistics[a]

| | ALB_EST_Perc_ST_W2 - MEM_Perc_ST_W2 | ALB_EST_Perc_ST_W3 - MEM_Perc_ST_W3 | ALB_EST_Perc_ST_W4 - MEM_Perc_ST_W4 | ALB_EST_Perc_ST_W5 - MEM_Perc_ST_W5 | ALB_EST_Perc_ST_W6 - MEM_Perc_ST_W6 |
|---|---|---|---|---|---|
| Z | -,674[b] | -,524[c] | -1,782[b] | -,105[b] | -1,992[c] |
| Asymp. Sig. (2-tailed) | ,500 | ,600 | ,075 | ,917 | ,046 |

a. Wilcoxon Signed Ranks Test

b. Based on positive ranks.

c. Based on negative ranks.

```
NPAR TESTS
  /WILCOXON=Perc_Int_0 Perc_Int_0 Perc_Int_0 Perc_Int_0 Perc_Int_0 WITH MEM_Perc_Int_W2 MEM_Per
  /MISSING ANALYSIS.
```

# NPar Tests

**Notes**

| | | |
|---|---|---|
| Output Created | | 16-AUG-2017 16:18:03 |
| Comments | | |
| Input | Data | /Users/pmota/Desktop/Organ Culture_treatment and ... |
| | Active Dataset | DataSet1 |
| | Filter | <none> |
| | Weight | <none> |
| | Split File | <none> |
| | N of Rows in Working Data File | 6 |
| Missing Value Handling | Definition of Missing | User-defined missing values are treated as missing. |
| | Cases Used | Statistics for each test are based on all cases with valid data for the variable(s) used in that test. |
| Syntax | | NPAR TESTS /WILCOXON=Perc_Int_0 Perc_Int_0 Perc_Int_0 Perc_Int_0 Perc_Int_0 WITH MEM_Perc_Int_W2 MEM_Perc_Int_W3 MEM_Perc_Int_W4 MEM_Perc_Int_W5 MEM_Perc_Int_W6 (PAIRED) /MISSING ANALYSIS. |
| Resources | Processor Time | 00:00:00,01 |
| | Elapsed Time | 00:00:00,00 |
| | Number of Cases Allowed[a] | 71493 |

a. Based on availability of workspace memory.

```
[DataSet1] /Users/pmota/Desktop/Organ Culture_treatment and week.sav
```

# Wilcoxon Signed Ranks Test

**Ranks**

| | | N | Mean Rank | Sum of Ranks |
|---|---|---|---|---|
| MEM_Perc_Int_W2 - Perc_Int_0 | Negative Ranks | 0[a] | ,00 | ,00 |
| | Positive Ranks | 5[b] | 3,00 | 15,00 |
| | Ties | 0[c] | | |
| | Total | 5 | | |
| MEM_Perc_Int_W3 - Perc_Int_0 | Negative Ranks | 0[d] | ,00 | ,00 |
| | Positive Ranks | 6[e] | 3,50 | 21,00 |
| | Ties | 0[f] | | |
| | Total | 6 | | |
| MEM_Perc_Int_W4 - Perc_Int_0 | Negative Ranks | 0[g] | ,00 | ,00 |
| | Positive Ranks | 6[h] | 3,50 | 21,00 |
| | Ties | 0[i] | | |
| | Total | 6 | | |
| MEM_Perc_Int_W5 - Perc_Int_0 | Negative Ranks | 0[j] | ,00 | ,00 |
| | Positive Ranks | 6[k] | 3,50 | 21,00 |
| | Ties | 0[l] | | |
| | Total | 6 | | |
| MEM_Perc_Int_W6 - Perc_Int_0 | Negative Ranks | 0[m] | ,00 | ,00 |
| | Positive Ranks | 6[n] | 3,50 | 21,00 |
| | Ties | 0[o] | | |
| | Total | 6 | | |

a. MEM_Perc_Int_W2 < Perc_Int_0

b. MEM_Perc_Int_W2 > Perc_Int_0

c. MEM_Perc_Int_W2 = Perc_Int_0

d. MEM_Perc_Int_W3 < Perc_Int_0

e. MEM_Perc_Int_W3 > Perc_Int_0

f. MEM_Perc_Int_W3 = Perc_Int_0

g. MEM_Perc_Int_W4 < Perc_Int_0

h. MEM_Perc_Int_W4 > Perc_Int_0

i. MEM_Perc_Int_W4 = Perc_Int_0

j. MEM_Perc_Int_W5 < Perc_Int_0

k. MEM_Perc_Int_W5 > Perc_Int_0

l. MEM_Perc_Int_W5 = Perc_Int_0

m. MEM_Perc_Int_W6 < Perc_Int_0

n. MEM_Perc_Int_W6 > Perc_Int_0

o. MEM_Perc_Int_W6 = Perc_Int_0

**Test Statistics[a]**

| | MEM_Perc_Int_W2 - Perc_Int_0 | MEM_Perc_Int_W3 - Perc_Int_0 | MEM_Perc_Int_W4 - Perc_Int_0 | MEM_Perc_Int_W5 - Perc_Int_0 | MEM_Perc_Int_W6 - Perc_Int_0 |
|---|---|---|---|---|---|
| Z | -2,023[b] | -2,201[b] | -2,201[b] | -2,201[b] | -2,201[b] |
| Asymp. Sig. (2-tailed) | ,043 | ,028 | ,028 | ,028 | ,028 |

a. Wilcoxon Signed Ranks Test

b. Based on negative ranks.

```
NPAR TESTS
 /WILCOXON=Perc_Int_0 Perc_Int_0 Perc_Int_0 Perc_Int_0 Perc_Int_0 WITH KSR_Perc_Int_W2 KSR_Per
 /MISSING ANALYSIS.
```

# NPar Tests

**Notes**

| | | |
|---|---|---|
| Output Created | | 16-AUG-2017 16:19:24 |
| Comments | | |
| Input | Data | /Users/pmota/Desktop/Organ Culture_treatment and ... |
| | Active Dataset | DataSet1 |
| | Filter | <none> |
| | Weight | <none> |
| | Split File | <none> |
| | N of Rows in Working Data File | 6 |
| Missing Value Handling | Definition of Missing | User-defined missing values are treated as missing. |
| | Cases Used | Statistics for each test are based on all cases with valid data for the variable(s) used in that test. |
| Syntax | | NPAR TESTS /WILCOXON=Perc_Int_0 Perc_Int_0 Perc_Int_0 Perc_Int_0 Perc_Int_0 WITH KSR_Perc_Int_W2 KSR_Perc_Int_W3 KSR_Perc_Int_W4 KSR_Perc_Int_W5 KSR_Perc_Int_W6 (PAIRED) /MISSING ANALYSIS. |
| Resources | Processor Time | 00:00:00,01 |
| | Elapsed Time | 00:00:00,00 |
| | Number of Cases Allowed[a] | 71493 |

a. Based on availability of workspace memory.

```
[DataSet1] /Users/pmota/Desktop/Organ Culture_treatment and week.sav
```

# Wilcoxon Signed Ranks Test

**Ranks**

| | | N | Mean Rank | Sum of Ranks |
|---|---|---|---|---|
| KSR_Perc_Int_W2 - Perc_Int_0 | Negative Ranks | 0[a] | ,00 | ,00 |
| | Positive Ranks | 5[b] | 3,00 | 15,00 |
| | Ties | 0[c] | | |
| | Total | 5 | | |
| KSR_Perc_Int_W3 - Perc_Int_0 | Negative Ranks | 0[d] | ,00 | ,00 |
| | Positive Ranks | 6[e] | 3,50 | 21,00 |
| | Ties | 0[f] | | |
| | Total | 6 | | |
| KSR_Perc_Int_W4 - Perc_Int_0 | Negative Ranks | 0[g] | ,00 | ,00 |
| | Positive Ranks | 5[h] | 3,00 | 15,00 |
| | Ties | 0[i] | | |
| | Total | 5 | | |
| KSR_Perc_Int_W5 - Perc_Int_0 | Negative Ranks | 0[j] | ,00 | ,00 |
| | Positive Ranks | 6[k] | 3,50 | 21,00 |
| | Ties | 0[l] | | |
| | Total | 6 | | |
| KSR_Perc_Int_W6 - Perc_Int_0 | Negative Ranks | 0[m] | ,00 | ,00 |
| | Positive Ranks | 6[n] | 3,50 | 21,00 |
| | Ties | 0[o] | | |
| | Total | 6 | | |

a. KSR_Perc_Int_W2 < Perc_Int_0

b. KSR_Perc_Int_W2 > Perc_Int_0

c. KSR_Perc_Int_W2 = Perc_Int_0

d. KSR_Perc_Int_W3 < Perc_Int_0

e. KSR_Perc_Int_W3 > Perc_Int_0

f. KSR_Perc_Int_W3 = Perc_Int_0

g. KSR_Perc_Int_W4 < Perc_Int_0

h. KSR_Perc_Int_W4 > Perc_Int_0

i. KSR_Perc_Int_W4 = Perc_Int_0

j. KSR_Perc_Int_W5 < Perc_Int_0

k. KSR_Perc_Int_W5 > Perc_Int_0

l. KSR_Perc_Int_W5 = Perc_Int_0

m. KSR_Perc_Int_W6 < Perc_Int_0

n. KSR_Perc_Int_W6 > Perc_Int_0

o. KSR_Perc_Int_W6 = Perc_Int_0

**Test Statistics[a]**

| | KSR_Perc_Int_W2 - Perc_Int_0 | KSR_Perc_Int_W3 - Perc_Int_0 | KSR_Perc_Int_W4 - Perc_Int_0 | KSR_Perc_Int_W5 - Perc_Int_0 | KSR_Perc_Int_W6 - Perc_Int_0 |
|---|---|---|---|---|---|
| Z | -2,023[b] | -2,201[b] | -2,023[b] | -2,201[b] | -2,201[b] |
| Asymp. Sig. (2-tailed) | ,043 | ,028 | ,043 | ,028 | ,028 |

a. Wilcoxon Signed Ranks Test

b. Based on negative ranks.

```
NPAR TESTS
 /WILCOXON=Perc_Int_0 Perc_Int_0 Perc_Int_0 Perc_Int_0 Perc_Int_0 WITH EST_Perc_Int_W2 EST_Per
 /MISSING ANALYSIS.
```

# NPar Tests

**Notes**

| | | |
|---|---|---|
| Output Created | | 16-AUG-2017 16:20:16 |
| Comments | | |
| Input | Data | /Users/pmota/Desktop/Organ Culture_treatment and ... |
| | Active Dataset | DataSet1 |
| | Filter | <none> |
| | Weight | <none> |
| | Split File | <none> |
| | N of Rows in Working Data File | 6 |
| Missing Value Handling | Definition of Missing | User-defined missing values are treated as missing. |
| | Cases Used | Statistics for each test are based on all cases with valid data for the variable(s) used in that test. |
| Syntax | | NPAR TESTS /WILCOXON=Perc_Int_0 Perc_Int_0 Perc_Int_0 Perc_Int_0 Perc_Int_0 WITH EST_Perc_Int_W2 EST_Perc_Int_W3 EST_Perc_Int_W4 EST_Perc_Int_W5 EST_Perc_Int_W6 (PAIRED) /MISSING ANALYSIS. |
| Resources | Processor Time | 00:00:00,01 |
| | Elapsed Time | 00:00:00,00 |
| | Number of Cases Allowed[a] | 71493 |

a. Based on availability of workspace memory.

```
[DataSet1] /Users/pmota/Desktop/Organ Culture_treatment and week.sav
```

# Wilcoxon Signed Ranks Test

**Ranks**

| | | N | Mean Rank | Sum of Ranks |
|---|---|---|---|---|
| EST_Perc_Int_W2 - Perc_Int_0 | Negative Ranks | 0[a] | ,00 | ,00 |
| | Positive Ranks | 4[b] | 2,50 | 10,00 |
| | Ties | 0[c] | | |
| | Total | 4 | | |
| EST_Perc_Int_W3 - Perc_Int_0 | Negative Ranks | 0[d] | ,00 | ,00 |
| | Positive Ranks | 6[e] | 3,50 | 21,00 |
| | Ties | 0[f] | | |
| | Total | 6 | | |
| EST_Perc_Int_W4 - Perc_Int_0 | Negative Ranks | 0[g] | ,00 | ,00 |
| | Positive Ranks | 6[h] | 3,50 | 21,00 |
| | Ties | 0[i] | | |
| | Total | 6 | | |
| EST_Perc_Int_W5 - Perc_Int_0 | Negative Ranks | 0[j] | ,00 | ,00 |
| | Positive Ranks | 6[k] | 3,50 | 21,00 |
| | Ties | 0[l] | | |
| | Total | 6 | | |
| EST_Perc_Int_W6 - Perc_Int_0 | Negative Ranks | 0[m] | ,00 | ,00 |
| | Positive Ranks | 6[n] | 3,50 | 21,00 |
| | Ties | 0[o] | | |
| | Total | 6 | | |

a. EST_Perc_Int_W2 < Perc_Int_0

b. EST_Perc_Int_W2 > Perc_Int_0

c. EST_Perc_Int_W2 = Perc_Int_0

d. EST_Perc_Int_W3 < Perc_Int_0

e. EST_Perc_Int_W3 > Perc_Int_0

f. EST_Perc_Int_W3 = Perc_Int_0

g. EST_Perc_Int_W4 < Perc_Int_0

h. EST_Perc_Int_W4 > Perc_Int_0

i. EST_Perc_Int_W4 = Perc_Int_0

j. EST_Perc_Int_W5 < Perc_Int_0

k. EST_Perc_Int_W5 > Perc_Int_0

l. EST_Perc_Int_W5 = Perc_Int_0

m. EST_Perc_Int_W6 < Perc_Int_0

n. EST_Perc_Int_W6 > Perc_Int_0

o. EST_Perc_Int_W6 = Perc_Int_0

**Test Statistics[a]**

| | EST_Perc_Int_W2 - Perc_Int_0 | EST_Perc_Int_W3 - Perc_Int_0 | EST_Perc_Int_W4 - Perc_Int_0 | EST_Perc_Int_W5 - Perc_Int_0 | EST_Perc_Int_W6 - Perc_Int_0 |
|---|---|---|---|---|---|
| Z | -1,826[b] | -2,201[b] | -2,201[b] | -2,201[b] | -2,201[b] |
| Asymp. Sig. (2-tailed) | ,068 | ,028 | ,028 | ,028 | ,028 |

a. Wilcoxon Signed Ranks Test

b. Based on negative ranks.

```
NPAR TESTS
 /WILCOXON=Perc_Int_0 Perc_Int_0 Perc_Int_0 Perc_Int_0 Perc_Int_0 WITH ALB_Perc_Int_W2 ALB_Per
 /MISSING ANALYSIS.
```

# NPar Tests

**Notes**

| | | |
|---|---|---|
| Output Created | | 16-AUG-2017 16:20:48 |
| Comments | | |
| Input | Data | /Users/pmota/Desktop/Organ Culture_treatment and ... |
| | Active Dataset | DataSet1 |
| | Filter | <none> |
| | Weight | <none> |
| | Split File | <none> |
| | N of Rows in Working Data File | 6 |
| Missing Value Handling | Definition of Missing | User-defined missing values are treated as missing. |
| | Cases Used | Statistics for each test are based on all cases with valid data for the variable(s) used in that test. |
| Syntax | | NPAR TESTS /WILCOXON=Perc_Int_0 Perc_Int_0 Perc_Int_0 Perc_Int_0 Perc_Int_0 WITH ALB_Perc_Int_W2 ALB_Perc_Int_W3 ALB_Perc_Int_W4 ALB_Perc_Int_W5 ALB_Perc_Int_W6 (PAIRED) /MISSING ANALYSIS. |
| Resources | Processor Time | 00:00:00,01 |
| | Elapsed Time | 00:00:00,00 |
| | Number of Cases Allowed[a] | 71493 |

a. Based on availability of workspace memory.

```
[DataSet1] /Users/pmota/Desktop/Organ Culture_treatment and week.sav
```

# Wilcoxon Signed Ranks Test

**Ranks**

| | | N | Mean Rank | Sum of Ranks |
|---|---|---|---|---|
| ALB_Perc_Int_W2 - Perc_Int_0 | Negative Ranks | 2[a] | 3,00 | 6,00 |
| | Positive Ranks | 3[b] | 3,00 | 9,00 |
| | Ties | 0[c] | | |
| | Total | 5 | | |
| ALB_Perc_Int_W3 - Perc_Int_0 | Negative Ranks | 1[d] | 1,00 | 1,00 |
| | Positive Ranks | 5[e] | 4,00 | 20,00 |
| | Ties | 0[f] | | |
| | Total | 6 | | |
| ALB_Perc_Int_W4 - Perc_Int_0 | Negative Ranks | 0[g] | ,00 | ,00 |
| | Positive Ranks | 6[h] | 3,50 | 21,00 |
| | Ties | 0[i] | | |
| | Total | 6 | | |
| ALB_Perc_Int_W5 - Perc_Int_0 | Negative Ranks | 2[j] | 2,00 | 4,00 |
| | Positive Ranks | 4[k] | 4,25 | 17,00 |
| | Ties | 0[l] | | |
| | Total | 6 | | |
| ALB_Perc_Int_W6 - Perc_Int_0 | Negative Ranks | 0[m] | ,00 | ,00 |
| | Positive Ranks | 6[n] | 3,50 | 21,00 |
| | Ties | 0[o] | | |
| | Total | 6 | | |

a. ALB_Perc_Int_W2 < Perc_Int_0

b. ALB_Perc_Int_W2 > Perc_Int_0

c. ALB_Perc_Int_W2 = Perc_Int_0

d. ALB_Perc_Int_W3 < Perc_Int_0

e. ALB_Perc_Int_W3 > Perc_Int_0

f. ALB_Perc_Int_W3 = Perc_Int_0

g. ALB_Perc_Int_W4 < Perc_Int_0

h. ALB_Perc_Int_W4 > Perc_Int_0

i. ALB_Perc_Int_W4 = Perc_Int_0

j. ALB_Perc_Int_W5 < Perc_Int_0

k. ALB_Perc_Int_W5 > Perc_Int_0

l. ALB_Perc_Int_W5 = Perc_Int_0

m. ALB_Perc_Int_W6 < Perc_Int_0

n. ALB_Perc_Int_W6 > Perc_Int_0

o. ALB_Perc_Int_W6 = Perc_Int_0

**Test Statistics[a]**

| | ALB_Perc_Int_W2 - Perc_Int_0 | ALB_Perc_Int_W3 - Perc_Int_0 | ALB_Perc_Int_W4 - Perc_Int_0 | ALB_Perc_Int_W5 - Perc_Int_0 | ALB_Perc_Int_W6 - Perc_Int_0 |
|---|---|---|---|---|---|
| Z | -,405[b] | -1,992[b] | -2,201[b] | -1,363[b] | -2,201[b] |
| Asymp. Sig. (2-tailed) | ,686 | ,046 | ,028 | ,173 | ,028 |

a. Wilcoxon Signed Ranks Test

b. Based on negative ranks.

```
NPAR TESTS
 /WILCOXON=Perc_Int_0 Perc_Int_0 Perc_Int_0 Perc_Int_0 Perc_Int_0 WITH ALB_EST_Perc_Int_W2 AL
 /MISSING ANALYSIS.
```

## NPar Tests

**Notes**

| | | |
|---|---|---|
| Output Created | | 16-AUG-2017 16:21:33 |
| Comments | | |
| Input | Data | /Users/pmota/Desktop/Organ Culture_treatment and ... |
| | Active Dataset | DataSet1 |
| | Filter | <none> |
| | Weight | <none> |
| | Split File | <none> |
| | N of Rows in Working Data File | 6 |
| Missing Value Handling | Definition of Missing | User-defined missing values are treated as missing. |
| | Cases Used | Statistics for each test are based on all cases with valid data for the variable(s) used in that test. |
| Syntax | | NPAR TESTS /WILCOXON=Perc_Int_0 Perc_Int_0 Perc_Int_0 Perc_Int_0 Perc_Int_0 WITH ALB_EST_Perc_Int_W2 ALB_EST_Perc_Int_W3 ALB_EST_Perc_Int_W4 ALB_EST_Perc_Int_W5 ALB_EST_Perc_Int_W6 (PAIRED) /MISSING ANALYSIS. |
| Resources | Processor Time | 00:00:00,01 |
| | Elapsed Time | 00:00:01,00 |
| | Number of Cases Allowed[a] | 71493 |

a. Based on availability of workspace memory.

```
[DataSet1] /Users/pmota/Desktop/Organ Culture_treatment and week.sav
```

## Wilcoxon Signed Ranks Test

**Ranks**

| | | N | Mean Rank | Sum of Ranks |
|---|---|---|---|---|
| ALB_EST_Perc_Int_W2 - Perc_Int_0 | Negative Ranks | 1[a] | 5,00 | 5,00 |
| | Positive Ranks | 4[b] | 2,50 | 10,00 |
| | Ties | 0[c] | | |
| | Total | 5 | | |
| ALB_EST_Perc_Int_W3 - Perc_Int_0 | Negative Ranks | 0[d] | ,00 | ,00 |
| | Positive Ranks | 6[e] | 3,50 | 21,00 |
| | Ties | 0[f] | | |
| | Total | 6 | | |
| ALB_EST_Perc_Int_W4 - Perc_Int_0 | Negative Ranks | 1[g] | 3,00 | 3,00 |
| | Positive Ranks | 5[h] | 3,60 | 18,00 |
| | Ties | 0[i] | | |
| | Total | 6 | | |
| ALB_EST_Perc_Int_W5 - Perc_Int_0 | Negative Ranks | 0[j] | ,00 | ,00 |
| | Positive Ranks | 6[k] | 3,50 | 21,00 |
| | Ties | 0[l] | | |
| | Total | 6 | | |
| ALB_EST_Perc_Int_W6 - Perc_Int_0 | Negative Ranks | 0[m] | ,00 | ,00 |
| | Positive Ranks | 6[n] | 3,50 | 21,00 |
| | Ties | 0[o] | | |
| | Total | 6 | | |

a. ALB_EST_Perc_Int_W2 < Perc_Int_0

b. ALB_EST_Perc_Int_W2 > Perc_Int_0

c. ALB_EST_Perc_Int_W2 = Perc_Int_0

d. ALB_EST_Perc_Int_W3 < Perc_Int_0

e. ALB_EST_Perc_Int_W3 > Perc_Int_0

f. ALB_EST_Perc_Int_W3 = Perc_Int_0

g. ALB_EST_Perc_Int_W4 < Perc_Int_0

h. ALB_EST_Perc_Int_W4 > Perc_Int_0

i. ALB_EST_Perc_Int_W4 = Perc_Int_0

j. ALB_EST_Perc_Int_W5 < Perc_Int_0

k. ALB_EST_Perc_Int_W5 > Perc_Int_0

l. ALB_EST_Perc_Int_W5 = Perc_Int_0

m. ALB_EST_Perc_Int_W6 < Perc_Int_0

n. ALB_EST_Perc_Int_W6 > Perc_Int_0

o. ALB_EST_Perc_Int_W6 = Perc_Int_0

**Test Statistics[a]**

| | ALB_EST_Perc_Int_W2 - Perc_Int_0 | ALB_EST_Perc_Int_W3 - Perc_Int_0 | ALB_EST_Perc_Int_W4 - Perc_Int_0 | ALB_EST_Perc_Int_W5 - Perc_Int_0 | ALB_EST_Perc_Int_W6 - Perc_Int_0 |
|---|---|---|---|---|---|
| Z | -,674[b] | -2,201[b] | -1,572[b] | -2,201[b] | -2,201[b] |
| Asymp. Sig. (2-tailed) | ,500 | ,028 | ,116 | ,028 | ,028 |

a. Wilcoxon Signed Ranks Test

b. Based on negative ranks.

```
NPAR TESTS
 /WILCOXON=MEM_Perc_Int_W2 MEM_Perc_Int_W3 MEM_Perc_Int_W4 MEM_Perc_Int_W5 MEM_Perc_Int_W6 WIT
 /MISSING ANALYSIS.
```

# NPar Tests

**Notes**

| | | |
|---|---|---|
| Output Created | | 16-AUG-2017 16:23:50 |
| Comments | | |
| Input | Data | /Users/pmota/Desktop/Organ Culture_treatment and ... |
| | Active Dataset | DataSet1 |
| | Filter | <none> |
| | Weight | <none> |
| | Split File | <none> |
| | N of Rows in Working Data File | 6 |
| Missing Value Handling | Definition of Missing | User-defined missing values are treated as missing. |
| | Cases Used | Statistics for each test are based on all cases with valid data for the variable(s) used in that test. |
| Syntax | | NPAR TESTS /WILCOXON=MEM_Perc_Int_W2 MEM_Perc_Int_W3 MEM_Perc_Int_W4 MEM_Perc_Int_W5 MEM_Perc_Int_W6 WITH KSR_Perc_Int_W2 KSR_Perc_Int_W3 KSR_Perc_Int_W4 KSR_Perc_Int_W5 KSR_Perc_Int_W6 (PAIRED) /MISSING ANALYSIS. |
| Resources | Processor Time | 00:00:00,01 |
| | Elapsed Time | 00:00:00,00 |
| | Number of Cases Allowed[a] | 52428 |

a. Based on availability of workspace memory.

```
[DataSet1] /Users/pmota/Desktop/Organ Culture_treatment and week.sav
```

# Wilcoxon Signed Ranks Test

### Ranks

| | | N | Mean Rank | Sum of Ranks |
|---|---|---|---|---|
| KSR_Perc_Int_W2 - MEM_Perc_Int_W2 | Negative Ranks | 3[a] | 2,33 | 7,00 |
| | Positive Ranks | 2[b] | 4,00 | 8,00 |
| | Ties | 0[c] | | |
| | Total | 5 | | |
| KSR_Perc_Int_W3 - MEM_Perc_Int_W3 | Negative Ranks | 4[d] | 2,50 | 10,00 |
| | Positive Ranks | 2[e] | 5,50 | 11,00 |
| | Ties | 0[f] | | |
| | Total | 6 | | |
| KSR_Perc_Int_W4 - MEM_Perc_Int_W4 | Negative Ranks | 1[g] | 1,00 | 1,00 |
| | Positive Ranks | 4[h] | 3,50 | 14,00 |
| | Ties | 0[i] | | |
| | Total | 5 | | |
| KSR_Perc_Int_W5 - MEM_Perc_Int_W5 | Negative Ranks | 5[j] | 4,00 | 20,00 |
| | Positive Ranks | 1[k] | 1,00 | 1,00 |
| | Ties | 0[l] | | |
| | Total | 6 | | |
| KSR_Perc_Int_W6 - MEM_Perc_Int_W6 | Negative Ranks | 3[m] | 4,00 | 12,00 |
| | Positive Ranks | 3[n] | 3,00 | 9,00 |
| | Ties | 0[o] | | |
| | Total | 6 | | |

a. KSR_Perc_Int_W2 < MEM_Perc_Int_W2

b. KSR_Perc_Int_W2 > MEM_Perc_Int_W2

c. KSR_Perc_Int_W2 = MEM_Perc_Int_W2

d. KSR_Perc_Int_W3 < MEM_Perc_Int_W3

e. KSR_Perc_Int_W3 > MEM_Perc_Int_W3

f. KSR_Perc_Int_W3 = MEM_Perc_Int_W3

g. KSR_Perc_Int_W4 < MEM_Perc_Int_W4

h. KSR_Perc_Int_W4 > MEM_Perc_Int_W4

i. KSR_Perc_Int_W4 = MEM_Perc_Int_W4

j. KSR_Perc_Int_W5 < MEM_Perc_Int_W5

k. KSR_Perc_Int_W5 > MEM_Perc_Int_W5

l. KSR_Perc_Int_W5 = MEM_Perc_Int_W5

m. KSR_Perc_Int_W6 < MEM_Perc_Int_W6

n. KSR_Perc_Int_W6 > MEM_Perc_Int_W6

o. KSR_Perc_Int_W6 = MEM_Perc_Int_W6

### Test Statistics[a]

| | KSR_Perc_Int_W2 - MEM_Perc_Int_W2 | KSR_Perc_Int_W3 - MEM_Perc_Int_W3 | KSR_Perc_Int_W4 - MEM_Perc_Int_W4 | KSR_Perc_Int_W5 - MEM_Perc_Int_W5 | KSR_Perc_Int_W6 - MEM_Perc_Int_W6 |
|---|---|---|---|---|---|
| Z | -,135[b] | -,105[b] | -1,753[b] | -1,992[c] | -,314[c] |
| Asymp. Sig. (2-tailed) | ,893 | ,917 | ,080 | ,046 | ,753 |

a. Wilcoxon Signed Ranks Test

b. Based on negative ranks.

c. Based on positive ranks.

```
NPAR TESTS
 /WILCOXON=MEM_Perc_Int_W2 MEM_Perc_Int_W3 MEM_Perc_Int_W4 MEM_Perc_Int_W5 MEM_Perc_Int_W6 WI
```

/MISSING ANALYSIS.

## NPar Tests

**Notes**

| | | |
|---|---|---|
| Output Created | | 16-AUG-2017 16:24:57 |
| Comments | | |
| Input | Data | /Users/pmota/Desktop/Organ Culture_treatment and ... |
| | Active Dataset | DataSet1 |
| | Filter | <none> |
| | Weight | <none> |
| | Split File | <none> |
| | N of Rows in Working Data File | 6 |
| Missing Value Handling | Definition of Missing | User-defined missing values are treated as missing. |
| | Cases Used | Statistics for each test are based on all cases with valid data for the variable(s) used in that test. |
| Syntax | | NPAR TESTS<br>/WILCOXON=MEM_Perc_Int_W2 MEM_Perc_Int_W3 MEM_Perc_Int_W4 MEM_Perc_Int_W5 MEM_Perc_Int_W6 WITH EST_Perc_Int_W2 EST_Perc_Int_W3 EST_Perc_Int_W4 EST_Perc_Int_W5 EST_Perc_Int_W6 (PAIRED)<br>/MISSING ANALYSIS. |
| Resources | Processor Time | 00:00:00,01 |
| | Elapsed Time | 00:00:00,00 |
| | Number of Cases Allowed[a] | 52428 |

a. Based on availability of workspace memory.

[DataSet1] /Users/pmota/Desktop/Organ Culture_treatment and week.sav

## Wilcoxon Signed Ranks Test

Ranks

| | | N | Mean Rank | Sum of Ranks |
|---|---|---|---|---|
| EST_Perc_Int_W2 - MEM_Perc_Int_W2 | Negative Ranks | 2[a] | 2,50 | 5,00 |
| | Positive Ranks | 2[b] | 2,50 | 5,00 |
| | Ties | 0[c] | | |
| | Total | 4 | | |
| EST_Perc_Int_W3 - MEM_Perc_Int_W3 | Negative Ranks | 1[d] | 6,00 | 6,00 |
| | Positive Ranks | 5[e] | 3,00 | 15,00 |
| | Ties | 0[f] | | |
| | Total | 6 | | |
| EST_Perc_Int_W4 - MEM_Perc_Int_W4 | Negative Ranks | 3[g] | 3,00 | 9,00 |
| | Positive Ranks | 3[h] | 4,00 | 12,00 |
| | Ties | 0[i] | | |
| | Total | 6 | | |
| EST_Perc_Int_W5 - MEM_Perc_Int_W5 | Negative Ranks | 3[j] | 3,67 | 11,00 |
| | Positive Ranks | 3[k] | 3,33 | 10,00 |
| | Ties | 0[l] | | |
| | Total | 6 | | |
| EST_Perc_Int_W6 - MEM_Perc_Int_W6 | Negative Ranks | 3[m] | 3,00 | 9,00 |
| | Positive Ranks | 3[n] | 4,00 | 12,00 |
| | Ties | 0[o] | | |
| | Total | 6 | | |

a. EST_Perc_Int_W2 < MEM_Perc_Int_W2

b. EST_Perc_Int_W2 > MEM_Perc_Int_W2

c. EST_Perc_Int_W2 = MEM_Perc_Int_W2

d. EST_Perc_Int_W3 < MEM_Perc_Int_W3

e. EST_Perc_Int_W3 > MEM_Perc_Int_W3

f. EST_Perc_Int_W3 = MEM_Perc_Int_W3

g. EST_Perc_Int_W4 < MEM_Perc_Int_W4

h. EST_Perc_Int_W4 > MEM_Perc_Int_W4

i. EST_Perc_Int_W4 = MEM_Perc_Int_W4

j. EST_Perc_Int_W5 < MEM_Perc_Int_W5

k. EST_Perc_Int_W5 > MEM_Perc_Int_W5

l. EST_Perc_Int_W5 = MEM_Perc_Int_W5

m. EST_Perc_Int_W6 < MEM_Perc_Int_W6

n. EST_Perc_Int_W6 > MEM_Perc_Int_W6

o. EST_Perc_Int_W6 = MEM_Perc_Int_W6

Test Statistics[a]

| | EST_Perc_Int_W2 - MEM_Perc_Int_W2 | EST_Perc_Int_W3 - MEM_Perc_Int_W3 | EST_Perc_Int_W4 - MEM_Perc_Int_W4 | EST_Perc_Int_W5 - MEM_Perc_Int_W5 | EST_Perc_Int_W6 - MEM_Perc_Int_W6 |
|---|---|---|---|---|---|
| Z | ,000[b] | -,943[c] | -,314[c] | -,105[d] | -,314[c] |
| Asymp. Sig. (2-tailed) | 1,000 | ,345 | ,753 | ,917 | ,753 |

a. Wilcoxon Signed Ranks Test

b. The sum of negative ranks equals the sum of positive ranks.

c. Based on negative ranks.

d. Based on positive ranks.

NPAR TESTS

```
/WILCOXON=MEM_Perc_Int_W2 MEM_Perc_Int_W3 MEM_Perc_Int_W4 MEM_Perc_Int_W5 MEM_Perc_Int_W6 WI
/MISSING ANALYSIS.
```

## NPar Tests

**Notes**

| | | |
|---|---|---|
| Output Created | | 16-AUG-2017 16:25:29 |
| Comments | | |
| Input | Data | /Users/pmota/Desktop/Organ Culture_treatment and ... |
| | Active Dataset | DataSet1 |
| | Filter | <none> |
| | Weight | <none> |
| | Split File | <none> |
| | N of Rows in Working Data File | 6 |
| Missing Value Handling | Definition of Missing | User-defined missing values are treated as missing. |
| | Cases Used | Statistics for each test are based on all cases with valid data for the variable(s) used in that test. |
| Syntax | | NPAR TESTS /WILCOXON=MEM_Perc_Int_W2 MEM_Perc_Int_W3 MEM_Perc_Int_W4 MEM_Perc_Int_W5 MEM_Perc_Int_W6 WITH ALB_Perc_Int_W2 ALB_Perc_Int_W3 ALB_Perc_Int_W4 ALB_Perc_Int_W5 ALB_Perc_Int_W6 (PAIRED) /MISSING ANALYSIS. |
| Resources | Processor Time | 00:00:00,01 |
| | Elapsed Time | 00:00:00,00 |
| | Number of Cases Allowed[a] | 52428 |

a. Based on availability of workspace memory.

```
[DataSet1] /Users/pmota/Desktop/Organ Culture_treatment and week.sav
```

## Wilcoxon Signed Ranks Test

### Ranks

| | | N | Mean Rank | Sum of Ranks |
|---|---|---|---|---|
| ALB_Perc_Int_W2 - MEM_Perc_Int_W2 | Negative Ranks | 4[a] | 3,25 | 13,00 |
| | Positive Ranks | 1[b] | 2,00 | 2,00 |
| | Ties | 0[c] | | |
| | Total | 5 | | |
| ALB_Perc_Int_W3 - MEM_Perc_Int_W3 | Negative Ranks | 3[d] | 3,67 | 11,00 |
| | Positive Ranks | 3[e] | 3,33 | 10,00 |
| | Ties | 0[f] | | |
| | Total | 6 | | |
| ALB_Perc_Int_W4 - MEM_Perc_Int_W4 | Negative Ranks | 3[g] | 3,67 | 11,00 |
| | Positive Ranks | 3[h] | 3,33 | 10,00 |
| | Ties | 0[i] | | |
| | Total | 6 | | |
| ALB_Perc_Int_W5 - MEM_Perc_Int_W5 | Negative Ranks | 5[j] | 3,40 | 17,00 |
| | Positive Ranks | 1[k] | 4,00 | 4,00 |
| | Ties | 0[l] | | |
| | Total | 6 | | |
| ALB_Perc_Int_W6 - MEM_Perc_Int_W6 | Negative Ranks | 4[m] | 4,50 | 18,00 |
| | Positive Ranks | 2[n] | 1,50 | 3,00 |
| | Ties | 0[o] | | |
| | Total | 6 | | |

a. ALB_Perc_Int_W2 < MEM_Perc_Int_W2

b. ALB_Perc_Int_W2 > MEM_Perc_Int_W2

c. ALB_Perc_Int_W2 = MEM_Perc_Int_W2

d. ALB_Perc_Int_W3 < MEM_Perc_Int_W3

e. ALB_Perc_Int_W3 > MEM_Perc_Int_W3

f. ALB_Perc_Int_W3 = MEM_Perc_Int_W3

g. ALB_Perc_Int_W4 < MEM_Perc_Int_W4

h. ALB_Perc_Int_W4 > MEM_Perc_Int_W4

i. ALB_Perc_Int_W4 = MEM_Perc_Int_W4

j. ALB_Perc_Int_W5 < MEM_Perc_Int_W5

k. ALB_Perc_Int_W5 > MEM_Perc_Int_W5

l. ALB_Perc_Int_W5 = MEM_Perc_Int_W5

m. ALB_Perc_Int_W6 < MEM_Perc_Int_W6

n. ALB_Perc_Int_W6 > MEM_Perc_Int_W6

o. ALB_Perc_Int_W6 = MEM_Perc_Int_W6

### Test Statistics[a]

| | ALB_Perc_Int_W2 - MEM_Perc_Int_W2 | ALB_Perc_Int_W3 - MEM_Perc_Int_W3 | ALB_Perc_Int_W4 - MEM_Perc_Int_W4 | ALB_Perc_Int_W5 - MEM_Perc_Int_W5 | ALB_Perc_Int_W6 - MEM_Perc_Int_W6 |
|---|---|---|---|---|---|
| Z | -1,483[b] | -,105[b] | -,105[b] | -1,363[b] | -1,572[b] |
| Asymp. Sig. (2-tailed) | ,138 | ,917 | ,917 | ,173 | ,116 |

a. Wilcoxon Signed Ranks Test

b. Based on positive ranks.

```
NPAR TESTS
 /WILCOXON=MEM_Perc_Int_W2 MEM_Perc_Int_W3 MEM_Perc_Int_W4 MEM_Perc_Int_W5 MEM_Perc_Int_W6 WI
 /MISSING ANALYSIS.
```

# NPar Tests

**Notes**

| | | |
|---|---|---|
| Output Created | | 16-AUG-2017 16:25:58 |
| Comments | | |
| Input | Data | /Users/pmota/Desktop/Organ Culture_treatment and ... |
| | Active Dataset | DataSet1 |
| | Filter | <none> |
| | Weight | <none> |
| | Split File | <none> |
| | N of Rows in Working Data File | 6 |
| Missing Value Handling | Definition of Missing | User-defined missing values are treated as missing. |
| | Cases Used | Statistics for each test are based on all cases with valid data for the variable(s) used in that test. |
| Syntax | | NPAR TESTS /WILCOXON=MEM_Perc_Int_W2 MEM_Perc_Int_W3 MEM_Perc_Int_W4 MEM_Perc_Int_W5 MEM_Perc_Int_W6 WITH ALB_EST_Perc_Int_W2 ALB_EST_Perc_Int_W3 ALB_EST_Perc_Int_W4 ALB_EST_Perc_Int_W5 ALB_EST_Perc_Int_W6 (PAIRED) /MISSING ANALYSIS. |
| Resources | Processor Time | 00:00:00,02 |
| | Elapsed Time | 00:00:00,00 |
| | Number of Cases Allowed[a] | 52428 |

a. Based on availability of workspace memory.

[DataSet1] /Users/pmota/Desktop/Organ Culture_treatment and week.sav

# Wilcoxon Signed Ranks Test

## Ranks

| | | N | Mean Rank | Sum of Ranks |
|---|---|---|---|---|
| ALB_EST_Perc_Int_W2 - MEM_Perc_Int_W2 | Negative Ranks | 4[a] | 3,50 | 14,00 |
| | Positive Ranks | 1[b] | 1,00 | 1,00 |
| | Ties | 0[c] | | |
| | Total | 5 | | |
| ALB_EST_Perc_Int_W3 - MEM_Perc_Int_W3 | Negative Ranks | 3[d] | 3,67 | 11,00 |
| | Positive Ranks | 3[e] | 3,33 | 10,00 |
| | Ties | 0[f] | | |
| | Total | 6 | | |
| ALB_EST_Perc_Int_W4 - MEM_Perc_Int_W4 | Negative Ranks | 4[g] | 3,50 | 14,00 |
| | Positive Ranks | 2[h] | 3,50 | 7,00 |
| | Ties | 0[i] | | |
| | Total | 6 | | |
| ALB_EST_Perc_Int_W5 - MEM_Perc_Int_W5 | Negative Ranks | 3[j] | 5,00 | 15,00 |
| | Positive Ranks | 3[k] | 2,00 | 6,00 |
| | Ties | 0[l] | | |
| | Total | 6 | | |
| ALB_EST_Perc_Int_W6 - MEM_Perc_Int_W6 | Negative Ranks | 6[m] | 3,50 | 21,00 |
| | Positive Ranks | 0[n] | ,00 | ,00 |
| | Ties | 0[o] | | |
| | Total | 6 | | |

a. ALB_EST_Perc_Int_W2 < MEM_Perc_Int_W2

b. ALB_EST_Perc_Int_W2 > MEM_Perc_Int_W2

c. ALB_EST_Perc_Int_W2 = MEM_Perc_Int_W2

d. ALB_EST_Perc_Int_W3 < MEM_Perc_Int_W3

e. ALB_EST_Perc_Int_W3 > MEM_Perc_Int_W3

f. ALB_EST_Perc_Int_W3 = MEM_Perc_Int_W3

g. ALB_EST_Perc_Int_W4 < MEM_Perc_Int_W4

h. ALB_EST_Perc_Int_W4 > MEM_Perc_Int_W4

i. ALB_EST_Perc_Int_W4 = MEM_Perc_Int_W4

j. ALB_EST_Perc_Int_W5 < MEM_Perc_Int_W5

k. ALB_EST_Perc_Int_W5 > MEM_Perc_Int_W5

l. ALB_EST_Perc_Int_W5 = MEM_Perc_Int_W5

m. ALB_EST_Perc_Int_W6 < MEM_Perc_Int_W6

n. ALB_EST_Perc_Int_W6 > MEM_Perc_Int_W6

o. ALB_EST_Perc_Int_W6 = MEM_Perc_Int_W6

## Test Statistics[a]

| | ALB_EST_Perc_Int_W2 - MEM_Perc_Int_W2 | ALB_EST_Perc_Int_W3 - MEM_Perc_Int_W3 | ALB_EST_Perc_Int_W4 - MEM_Perc_Int_W4 | ALB_EST_Perc_Int_W5 - MEM_Perc_Int_W5 | ALB_EST_Perc_Int_W6 - MEM_Perc_Int_W6 |
|---|---|---|---|---|---|
| Z | -1,753[b] | -,105[b] | -,734[b] | -,943[b] | -2,201[b] |
| Asymp. Sig. (2-tailed) | ,080 | ,917 | ,463 | ,345 | ,028 |

a. Wilcoxon Signed Ranks Test

b. Based on positive ranks.

```
NPAR TESTS
  /WILCOXON=MEM_Perc_Nec_W2 MEM_Perc_Nec_W3 MEM_Perc_Nec_W4 MEM_Perc_Nec_W5 MEM_Perc_Nec_W6 WI
  /MISSING ANALYSIS.
```

## NPar Tests

**Notes**

| | | |
|---|---|---|
| Output Created | | 16-AUG-2017 16:39:05 |
| Comments | | |
| Input | Data | /Users/pmota/Desktop/Organ Culture_treatment and ... |
| | Active Dataset | DataSet1 |
| | Filter | <none> |
| | Weight | <none> |
| | Split File | <none> |
| | N of Rows in Working Data File | 6 |
| Missing Value Handling | Definition of Missing | User-defined missing values are treated as missing. |
| | Cases Used | Statistics for each test are based on all cases with valid data for the variable(s) used in that test. |
| Syntax | | NPAR TESTS /WILCOXON=MEM_Perc_Nec_W2 MEM_Perc_Nec_W3 MEM_Perc_Nec_W4 MEM_Perc_Nec_W5 MEM_Perc_Nec_W6 WITH KSR_Perc_Nec_W2 KSR_Perc_Nec_W3 KSR_Perc_Nec_W4 KSR_Perc_Nec_W5 KSR_Perc_Nec_W6 (PAIRED) /MISSING ANALYSIS. |
| Resources | Processor Time | 00:00:00,01 |
| | Elapsed Time | 00:00:00,00 |
| | Number of Cases Allowed[a] | 52428 |

a. Based on availability of workspace memory.

```
[DataSet1] /Users/pmota/Desktop/Organ Culture_treatment and week.sav
```

## Wilcoxon Signed Ranks Test

**Ranks**

| | | N | Mean Rank | Sum of Ranks |
|---|---|---|---|---|
| KSR_Perc_Nec_W2 - MEM_Perc_Nec_W2 | Negative Ranks | 3[a] | 3,67 | 11,00 |
| | Positive Ranks | 2[b] | 2,00 | 4,00 |
| | Ties | 0[c] | | |
| | Total | 5 | | |
| KSR_Perc_Nec_W3 - MEM_Perc_Nec_W3 | Negative Ranks | 6[d] | 3,50 | 21,00 |
| | Positive Ranks | 0[e] | ,00 | ,00 |
| | Ties | 0[f] | | |
| | Total | 6 | | |
| KSR_Perc_Nec_W4 - MEM_Perc_Nec_W4 | Negative Ranks | 4[g] | 2,75 | 11,00 |
| | Positive Ranks | 1[h] | 4,00 | 4,00 |
| | Ties | 0[i] | | |
| | Total | 5 | | |
| KSR_Perc_Nec_W5 - MEM_Perc_Nec_W5 | Negative Ranks | 2[j] | 3,00 | 6,00 |
| | Positive Ranks | 4[k] | 3,75 | 15,00 |
| | Ties | 0[l] | | |
| | Total | 6 | | |
| KSR_Perc_Nec_W6 - MEM_Perc_Nec_W6 | Negative Ranks | 4[m] | 2,50 | 10,00 |
| | Positive Ranks | 2[n] | 5,50 | 11,00 |
| | Ties | 0[o] | | |
| | Total | 6 | | |

a. KSR_Perc_Nec_W2 < MEM_Perc_Nec_W2

b. KSR_Perc_Nec_W2 > MEM_Perc_Nec_W2

c. KSR_Perc_Nec_W2 = MEM_Perc_Nec_W2

d. KSR_Perc_Nec_W3 < MEM_Perc_Nec_W3

e. KSR_Perc_Nec_W3 > MEM_Perc_Nec_W3

f. KSR_Perc_Nec_W3 = MEM_Perc_Nec_W3

g. KSR_Perc_Nec_W4 < MEM_Perc_Nec_W4

h. KSR_Perc_Nec_W4 > MEM_Perc_Nec_W4

i. KSR_Perc_Nec_W4 = MEM_Perc_Nec_W4

j. KSR_Perc_Nec_W5 < MEM_Perc_Nec_W5

k. KSR_Perc_Nec_W5 > MEM_Perc_Nec_W5

l. KSR_Perc_Nec_W5 = MEM_Perc_Nec_W5

m. KSR_Perc_Nec_W6 < MEM_Perc_Nec_W6

n. KSR_Perc_Nec_W6 > MEM_Perc_Nec_W6

o. KSR_Perc_Nec_W6 = MEM_Perc_Nec_W6

**Test Statistics[a]**

| | KSR_Perc_Nec_W2 - MEM_Perc_Nec_W2 | KSR_Perc_Nec_W3 - MEM_Perc_Nec_W3 | KSR_Perc_Nec_W4 - MEM_Perc_Nec_W4 | KSR_Perc_Nec_W5 - MEM_Perc_Nec_W5 | KSR_Perc_Nec_W6 - MEM_Perc_Nec_W6 |
|---|---|---|---|---|---|
| Z | -,944[b] | -2,201[b] | -,944[b] | -,943[c] | -,105[c] |
| Asymp. Sig. (2-tailed) | ,345 | ,028 | ,345 | ,345 | ,917 |

a. Wilcoxon Signed Ranks Test

b. Based on positive ranks.

c. Based on negative ranks.

```
NPAR TESTS
 /WILCOXON=MEM_Perc_Nec_W2 MEM_Perc_Nec_W3 MEM_Perc_Nec_W4 MEM_Perc_Nec_W5 MEM_Perc_Nec_W6 WI[illegible]
```

/MISSING ANALYSIS.

# NPar Tests

**Notes**

| | | |
|---|---|---|
| Output Created | | 16-AUG-2017 16:39:50 |
| Comments | | |
| Input | Data | /Users/pmota/Desktop/Organ Culture_treatment and ... |
| | Active Dataset | DataSet1 |
| | Filter | <none> |
| | Weight | <none> |
| | Split File | <none> |
| | N of Rows in Working Data File | 6 |
| Missing Value Handling | Definition of Missing | User-defined missing values are treated as missing. |
| | Cases Used | Statistics for each test are based on all cases with valid data for the variable(s) used in that test. |
| Syntax | | NPAR TESTS /WILCOXON=MEM_Perc_Nec_W2 MEM_Perc_Nec_W3 MEM_Perc_Nec_W4 MEM_Perc_Nec_W5 MEM_Perc_Nec_W6 WITH EST_Perc_Nec_W2 EST_Perc_Nec_W3 EST_Perc_Nec_W4 EST_Perc_Nec_W5 EST_Perc_Nec_W6 (PAIRED) /MISSING ANALYSIS. |
| Resources | Processor Time | 00:00:00,01 |
| | Elapsed Time | 00:00:00,00 |
| | Number of Cases Allowed[a] | 52428 |

a. Based on availability of workspace memory.

[DataSet1] /Users/pmota/Desktop/Organ Culture_treatment and week.sav

# Wilcoxon Signed Ranks Test

## Ranks

| | | N | Mean Rank | Sum of Ranks |
|---|---|---|---|---|
| EST_Perc_Nec_W2 - MEM_Perc_Nec_W2 | Negative Ranks | 2[a] | 2,00 | 4,00 |
| | Positive Ranks | 2[b] | 3,00 | 6,00 |
| | Ties | 0[c] | | |
| | Total | 4 | | |
| EST_Perc_Nec_W3 - MEM_Perc_Nec_W3 | Negative Ranks | 4[d] | 3,75 | 15,00 |
| | Positive Ranks | 2[e] | 3,00 | 6,00 |
| | Ties | 0[f] | | |
| | Total | 6 | | |
| EST_Perc_Nec_W4 - MEM_Perc_Nec_W4 | Negative Ranks | 3[g] | 4,00 | 12,00 |
| | Positive Ranks | 3[h] | 3,00 | 9,00 |
| | Ties | 0[i] | | |
| | Total | 6 | | |
| EST_Perc_Nec_W5 - MEM_Perc_Nec_W5 | Negative Ranks | 4[j] | 3,50 | 14,00 |
| | Positive Ranks | 2[k] | 3,50 | 7,00 |
| | Ties | 0[l] | | |
| | Total | 6 | | |
| EST_Perc_Nec_W6 - MEM_Perc_Nec_W6 | Negative Ranks | 3[m] | 4,00 | 12,00 |
| | Positive Ranks | 3[n] | 3,00 | 9,00 |
| | Ties | 0[o] | | |
| | Total | 6 | | |

a. EST_Perc_Nec_W2 < MEM_Perc_Nec_W2

b. EST_Perc_Nec_W2 > MEM_Perc_Nec_W2

c. EST_Perc_Nec_W2 = MEM_Perc_Nec_W2

d. EST_Perc_Nec_W3 < MEM_Perc_Nec_W3

e. EST_Perc_Nec_W3 > MEM_Perc_Nec_W3

f. EST_Perc_Nec_W3 = MEM_Perc_Nec_W3

g. EST_Perc_Nec_W4 < MEM_Perc_Nec_W4

h. EST_Perc_Nec_W4 > MEM_Perc_Nec_W4

i. EST_Perc_Nec_W4 = MEM_Perc_Nec_W4

j. EST_Perc_Nec_W5 < MEM_Perc_Nec_W5

k. EST_Perc_Nec_W5 > MEM_Perc_Nec_W5

l. EST_Perc_Nec_W5 = MEM_Perc_Nec_W5

m. EST_Perc_Nec_W6 < MEM_Perc_Nec_W6

n. EST_Perc_Nec_W6 > MEM_Perc_Nec_W6

o. EST_Perc_Nec_W6 = MEM_Perc_Nec_W6

## Test Statistics[a]

| | EST_Perc_Nec_W2 - MEM_Perc_Nec_W2 | EST_Perc_Nec_W3 - MEM_Perc_Nec_W3 | EST_Perc_Nec_W4 - MEM_Perc_Nec_W4 | EST_Perc_Nec_W5 - MEM_Perc_Nec_W5 | EST_Perc_Nec_W6 - MEM_Perc_Nec_W6 |
|---|---|---|---|---|---|
| Z | -,365[b] | -,943[c] | -,314[c] | -,734[c] | -,314[c] |
| Asymp. Sig. (2-tailed) | ,715 | ,345 | ,753 | ,463 | ,753 |

a. Wilcoxon Signed Ranks Test

b. Based on negative ranks.

c. Based on positive ranks.

```
NPAR TESTS
 /WILCOXON=MEM_Perc_Nec_W2 MEM_Perc_Nec_W3 MEM_Perc_Nec_W4 MEM_Perc_Nec_W5 MEM_Perc_Nec_W6 WIT
```

```
/MISSING ANALYSIS.
```

# NPar Tests

**Notes**

| | | |
|---|---|---|
| Output Created | | 16-AUG-2017 16:40:25 |
| Comments | | |
| Input | Data | /Users/pmota/Desktop/Organ Culture_treatment and ... |
| | Active Dataset | DataSet1 |
| | Filter | <none> |
| | Weight | <none> |
| | Split File | <none> |
| | N of Rows in Working Data File | 6 |
| Missing Value Handling | Definition of Missing | User-defined missing values are treated as missing. |
| | Cases Used | Statistics for each test are based on all cases with valid data for the variable(s) used in that test. |
| Syntax | | NPAR TESTS<br><br>/WILCOXON=MEM_Perc_Nec_W2 MEM_Perc_Nec_W3 MEM_Perc_Nec_W4 MEM_Perc_Nec_W5 MEM_Perc_Nec_W6 WITH ALB_Perc_Nec_W2 ALB_Perc_Nec_W3 ALB_Perc_Nec_W4 ALB_Perc_Nec_W5 ALB_Perc_Nec_W6 (PAIRED)<br>/MISSING ANALYSIS. |
| Resources | Processor Time | 00:00:00,01 |
| | Elapsed Time | 00:00:00,00 |
| | Number of Cases Allowed[a] | 52428 |

a. Based on availability of workspace memory.

```
[DataSet1] /Users/pmota/Desktop/Organ Culture_treatment and week.sav
```

# Wilcoxon Signed Ranks Test

**Ranks**

| | | N | Mean Rank | Sum of Ranks |
|---|---|---|---|---|
| ALB_Perc_Nec_W2 - MEM_Perc_Nec_W2 | Negative Ranks | 1[a] | 1,00 | 1,00 |
| | Positive Ranks | 4[b] | 3,50 | 14,00 |
| | Ties | 0[c] | | |
| | Total | 5 | | |
| ALB_Perc_Nec_W3 - MEM_Perc_Nec_W3 | Negative Ranks | 2[d] | 2,00 | 4,00 |
| | Positive Ranks | 4[e] | 4,25 | 17,00 |
| | Ties | 0[f] | | |
| | Total | 6 | | |
| ALB_Perc_Nec_W4 - MEM_Perc_Nec_W4 | Negative Ranks | 2[g] | 4,00 | 8,00 |
| | Positive Ranks | 4[h] | 3,25 | 13,00 |
| | Ties | 0[i] | | |
| | Total | 6 | | |
| ALB_Perc_Nec_W5 - MEM_Perc_Nec_W5 | Negative Ranks | 2[j] | 2,00 | 4,00 |
| | Positive Ranks | 4[k] | 4,25 | 17,00 |
| | Ties | 0[l] | | |
| | Total | 6 | | |
| ALB_Perc_Nec_W6 - MEM_Perc_Nec_W6 | Negative Ranks | 3[m] | 3,00 | 9,00 |
| | Positive Ranks | 3[n] | 4,00 | 12,00 |
| | Ties | 0[o] | | |
| | Total | 6 | | |

a. ALB_Perc_Nec_W2 < MEM_Perc_Nec_W2

b. ALB_Perc_Nec_W2 > MEM_Perc_Nec_W2

c. ALB_Perc_Nec_W2 = MEM_Perc_Nec_W2

d. ALB_Perc_Nec_W3 < MEM_Perc_Nec_W3

e. ALB_Perc_Nec_W3 > MEM_Perc_Nec_W3

f. ALB_Perc_Nec_W3 = MEM_Perc_Nec_W3

g. ALB_Perc_Nec_W4 < MEM_Perc_Nec_W4

h. ALB_Perc_Nec_W4 > MEM_Perc_Nec_W4

i. ALB_Perc_Nec_W4 = MEM_Perc_Nec_W4

j. ALB_Perc_Nec_W5 < MEM_Perc_Nec_W5

k. ALB_Perc_Nec_W5 > MEM_Perc_Nec_W5

l. ALB_Perc_Nec_W5 = MEM_Perc_Nec_W5

m. ALB_Perc_Nec_W6 < MEM_Perc_Nec_W6

n. ALB_Perc_Nec_W6 > MEM_Perc_Nec_W6

o. ALB_Perc_Nec_W6 = MEM_Perc_Nec_W6

**Test Statistics[a]**

| | ALB_Perc_Nec_W2 - MEM_Perc_Nec_W2 | ALB_Perc_Nec_W3 - MEM_Perc_Nec_W3 | ALB_Perc_Nec_W4 - MEM_Perc_Nec_W4 | ALB_Perc_Nec_W5 - MEM_Perc_Nec_W5 | ALB_Perc_Nec_W6 - MEM_Perc_Nec_W6 |
|---|---|---|---|---|---|
| Z | -1,753[b] | -1,363[b] | -,524[b] | -1,363[b] | -,314[b] |
| Asymp. Sig. (2-tailed) | ,080 | ,173 | ,600 | ,173 | ,753 |

a. Wilcoxon Signed Ranks Test

b. Based on negative ranks.

```
NPAR TESTS
 /WILCOXON=MEM_Perc_Nec_W2 MEM_Perc_Nec_W3 MEM_Perc_Nec_W4 MEM_Perc_Nec_W5 MEM_Perc_Nec_W6 WIT
 /MISSING ANALYSIS.
```

# NPar Tests

**Notes**

| | | |
|---|---|---|
| Output Created | | 16-AUG-2017 16:41:06 |
| Comments | | |
| Input | Data | /Users/pmota/Desktop/Organ Culture_treatment and ... |
| | Active Dataset | DataSet1 |
| | Filter | <none> |
| | Weight | <none> |
| | Split File | <none> |
| | N of Rows in Working Data File | 6 |
| Missing Value Handling | Definition of Missing | User-defined missing values are treated as missing. |
| | Cases Used | Statistics for each test are based on all cases with valid data for the variable(s) used in that test. |
| Syntax | | NPAR TESTS<br><br>/WILCOXON=MEM_Perc_Nec_W2 MEM_Perc_Nec_W3 MEM_Perc_Nec_W4 MEM_Perc_Nec_W5 MEM_Perc_Nec_W6 WITH ALB_EST_Perc_Nec_W2 ALB_EST_Perc_Nec_W3 ALB_EST_Perc_Nec_W4 ALB_EST_Perc_Nec_W5 ALB_EST_Perc_Nec_W6 (PAIRED)<br>/MISSING ANALYSIS. |
| Resources | Processor Time | 00:00:00,01 |
| | Elapsed Time | 00:00:00,00 |
| | Number of Cases Allowed[a] | 52428 |

a. Based on availability of workspace memory.

[DataSet1] /Users/pmota/Desktop/Organ Culture_treatment and week.sav

# Wilcoxon Signed Ranks Test

**Ranks**

| | | N | Mean Rank | Sum of Ranks |
|---|---|---|---|---|
| ALB_EST_Perc_Nec_W2 - MEM_Perc_Nec_W2 | Negative Ranks | 0[a] | ,00 | ,00 |
| | Positive Ranks | 5[b] | 3,00 | 15,00 |
| | Ties | 0[c] | | |
| | Total | 5 | | |
| ALB_EST_Perc_Nec_W3 - MEM_Perc_Nec_W3 | Negative Ranks | 2[d] | 5,00 | 10,00 |
| | Positive Ranks | 4[e] | 2,75 | 11,00 |
| | Ties | 0[f] | | |
| | Total | 6 | | |
| ALB_EST_Perc_Nec_W4 - MEM_Perc_Nec_W4 | Negative Ranks | 1[g] | 2,00 | 2,00 |
| | Positive Ranks | 5[h] | 3,80 | 19,00 |
| | Ties | 0[i] | | |
| | Total | 6 | | |
| ALB_EST_Perc_Nec_W5 - MEM_Perc_Nec_W5 | Negative Ranks | 1[j] | 2,00 | 2,00 |
| | Positive Ranks | 5[k] | 3,80 | 19,00 |
| | Ties | 0[l] | | |
| | Total | 6 | | |
| ALB_EST_Perc_Nec_W6 - MEM_Perc_Nec_W6 | Negative Ranks | 1[m] | 5,00 | 5,00 |
| | Positive Ranks | 5[n] | 3,20 | 16,00 |
| | Ties | 0[o] | | |
| | Total | 6 | | |

a. ALB_EST_Perc_Nec_W2 < MEM_Perc_Nec_W2

b. ALB_EST_Perc_Nec_W2 > MEM_Perc_Nec_W2

c. ALB_EST_Perc_Nec_W2 = MEM_Perc_Nec_W2

d. ALB_EST_Perc_Nec_W3 < MEM_Perc_Nec_W3

e. ALB_EST_Perc_Nec_W3 > MEM_Perc_Nec_W3

f. ALB_EST_Perc_Nec_W3 = MEM_Perc_Nec_W3

g. ALB_EST_Perc_Nec_W4 < MEM_Perc_Nec_W4

h. ALB_EST_Perc_Nec_W4 > MEM_Perc_Nec_W4

i. ALB_EST_Perc_Nec_W4 = MEM_Perc_Nec_W4

j. ALB_EST_Perc_Nec_W5 < MEM_Perc_Nec_W5

k. ALB_EST_Perc_Nec_W5 > MEM_Perc_Nec_W5

l. ALB_EST_Perc_Nec_W5 = MEM_Perc_Nec_W5

m. ALB_EST_Perc_Nec_W6 < MEM_Perc_Nec_W6

n. ALB_EST_Perc_Nec_W6 > MEM_Perc_Nec_W6

o. ALB_EST_Perc_Nec_W6 = MEM_Perc_Nec_W6

**Test Statistics[a]**

| | ALB_EST_Perc_Nec_W2 - MEM_Perc_Nec_W2 | ALB_EST_Perc_Nec_W3 - MEM_Perc_Nec_W3 | ALB_EST_Perc_Nec_W4 - MEM_Perc_Nec_W4 | ALB_EST_Perc_Nec_W5 - MEM_Perc_Nec_W5 | ALB_EST_Perc_Nec_W6 - MEM_Perc_Nec_W6 |
|---|---|---|---|---|---|
| Z | -2,023[b] | -,105[b] | -1,782[b] | -1,782[b] | -1,153[b] |
| Asymp. Sig. (2-tailed) | ,043 | ,917 | ,075 | ,075 | ,249 |

a. Wilcoxon Signed Ranks Test

b. Based on negative ranks.

```
NPAR TESTS
  /WILCOXON=GermCells_0 GermCells_0 GermCells_0 GermCells_0 GermCells_0 WITH MEM_GermCells_W2
  /MISSING ANALYSIS.
```

# NPar Tests

**Notes**

| | | |
|---|---|---|
| Output Created | | 16-AUG-2017 14:26:38 |
| Comments | | |
| Input | Data | /Users/pmota/Desktop/Organ Culture_treatment and ... |
| | Active Dataset | DataSet1 |
| | Filter | <none> |
| | Weight | <none> |
| | Split File | <none> |
| | N of Rows in Working Data File | 6 |
| Missing Value Handling | Definition of Missing | User-defined missing values are treated as missing. |
| | Cases Used | Statistics for each test are based on all cases with valid data for the variable(s) used in that test. |
| Syntax | | NPAR TESTS /WILCOXON=GermCells_0 GermCells_0 GermCells_0 GermCells_0 GermCells_0 WITH MEM_GermCells_W2 MEM_GermCells_W3 MEM_GermCells_W4 MEM_GermCells_W5 MEM_GermCells_W6 (PAIRED) /MISSING ANALYSIS. |
| Resources | Processor Time | 00:00:00,01 |
| | Elapsed Time | 00:00:00,00 |
| | Number of Cases Allowed[a] | 71493 |

a. Based on availability of workspace memory.

```
[DataSet1] /Users/pmota/Desktop/Organ Culture_treatment and week.sav
```

# Wilcoxon Signed Ranks Test

**Ranks**

| | | N | Mean Rank | Sum of Ranks |
|---|---|---|---|---|
| MEM_GermCells_W2 - GermCells_0 | Negative Ranks | 4[a] | 2,50 | 10,00 |
| | Positive Ranks | 1[b] | 5,00 | 5,00 |
| | Ties | 0[c] | | |
| | Total | 5 | | |
| MEM_GermCells_W3 - GermCells_0 | Negative Ranks | 5[d] | 3,20 | 16,00 |
| | Positive Ranks | 1[e] | 5,00 | 5,00 |
| | Ties | 0[f] | | |
| | Total | 6 | | |
| MEM_GermCells_W4 - GermCells_0 | Negative Ranks | 4[g] | 3,75 | 15,00 |
| | Positive Ranks | 2[h] | 3,00 | 6,00 |
| | Ties | 0[i] | | |
| | Total | 6 | | |
| MEM_GermCells_W5 - GermCells_0 | Negative Ranks | 4[j] | 4,25 | 17,00 |
| | Positive Ranks | 2[k] | 2,00 | 4,00 |
| | Ties | 0[l] | | |
| | Total | 6 | | |
| MEM_GermCells_W6 - GermCells_0 | Negative Ranks | 6[m] | 3,50 | 21,00 |
| | Positive Ranks | 0[n] | ,00 | ,00 |
| | Ties | 0[o] | | |
| | Total | 6 | | |

a. MEM_GermCells_W2 < GermCells_0

b. MEM_GermCells_W2 > GermCells_0

c. MEM_GermCells_W2 = GermCells_0

d. MEM_GermCells_W3 < GermCells_0

e. MEM_GermCells_W3 > GermCells_0

f. MEM_GermCells_W3 = GermCells_0

g. MEM_GermCells_W4 < GermCells_0

h. MEM_GermCells_W4 > GermCells_0

i. MEM_GermCells_W4 = GermCells_0

j. MEM_GermCells_W5 < GermCells_0

k. MEM_GermCells_W5 > GermCells_0

l. MEM_GermCells_W5 = GermCells_0

m. MEM_GermCells_W6 < GermCells_0

n. MEM_GermCells_W6 > GermCells_0

o. MEM_GermCells_W6 = GermCells_0

**Test Statistics[a]**

| | MEM_GermCells_W2 - GermCells_0 | MEM_GermCells_W3 - GermCells_0 | MEM_GermCells_W4 - GermCells_0 | MEM_GermCells_W5 - GermCells_0 | MEM_GermCells_W6 - GermCells_0 |
|---|---|---|---|---|---|
| Z | -,674[b] | -1,153[b] | -,943[b] | -1,363[b] | -2,201[b] |
| Asymp. Sig. (2-tailed) | ,500 | ,249 | ,345 | ,173 | ,028 |

a. Wilcoxon Signed Ranks Test

b. Based on positive ranks.

```
NPAR TESTS
  /WILCOXON=GermCells_0 GermCells_0 GermCells_0 GermCells_0 GermCells_0 WITH KSR_GermCells_W2
  /MISSING ANALYSIS.
```

# NPar Tests

**Notes**

| | | |
|---|---|---|
| Output Created | | 16-AUG-2017 14:27:41 |
| Comments | | |
| Input | Data | /Users/pmota/Desktop/Organ Culture_treatment and ... |
| | Active Dataset | DataSet1 |
| | Filter | <none> |
| | Weight | <none> |
| | Split File | <none> |
| | N of Rows in Working Data File | 6 |
| Missing Value Handling | Definition of Missing | User-defined missing values are treated as missing. |
| | Cases Used | Statistics for each test are based on all cases with valid data for the variable(s) used in that test. |
| Syntax | | NPAR TESTS<br><br>/WILCOXON=GermCells_0 GermCells_0 GermCells_0 GermCells_0 GermCells_0 WITH KSR_GermCells_W2 KSR_GermCells_W3 KSR_GermCells_W4 KSR_GermCells_W5 KSR_GermCells_W6 (PAIRED)<br>/MISSING ANALYSIS. |
| Resources | Processor Time | 00:00:00,01 |
| | Elapsed Time | 00:00:00,00 |
| | Number of Cases Allowed[a] | 71493 |

a. Based on availability of workspace memory.

```
[DataSet1] /Users/pmota/Desktop/Organ Culture_treatment and week.sav
```

# Wilcoxon Signed Ranks Test

**Ranks**

| | | N | Mean Rank | Sum of Ranks |
|---|---|---|---|---|
| KSR_GermCells_W2 - GermCells_0 | Negative Ranks | 4[a] | 3,50 | 14,00 |
| | Positive Ranks | 1[b] | 1,00 | 1,00 |
| | Ties | 0[c] | | |
| | Total | 5 | | |
| KSR_GermCells_W3 - GermCells_0 | Negative Ranks | 5[d] | 4,00 | 20,00 |
| | Positive Ranks | 1[e] | 1,00 | 1,00 |
| | Ties | 0[f] | | |
| | Total | 6 | | |
| KSR_GermCells_W4 - GermCells_0 | Negative Ranks | 4[g] | 3,50 | 14,00 |
| | Positive Ranks | 1[h] | 1,00 | 1,00 |
| | Ties | 0[i] | | |
| | Total | 5 | | |
| KSR_GermCells_W5 - GermCells_0 | Negative Ranks | 6[j] | 3,50 | 21,00 |
| | Positive Ranks | 0[k] | ,00 | ,00 |
| | Ties | 0[l] | | |
| | Total | 6 | | |
| KSR_GermCells_W6 - GermCells_0 | Negative Ranks | 5[m] | 4,00 | 20,00 |
| | Positive Ranks | 1[n] | 1,00 | 1,00 |
| | Ties | 0[o] | | |
| | Total | 6 | | |

a. KSR_GermCells_W2 < GermCells_0

b. KSR_GermCells_W2 > GermCells_0

c. KSR_GermCells_W2 = GermCells_0

d. KSR_GermCells_W3 < GermCells_0

e. KSR_GermCells_W3 > GermCells_0

f. KSR_GermCells_W3 = GermCells_0

g. KSR_GermCells_W4 < GermCells_0

h. KSR_GermCells_W4 > GermCells_0

i. KSR_GermCells_W4 = GermCells_0

j. KSR_GermCells_W5 < GermCells_0

k. KSR_GermCells_W5 > GermCells_0

l. KSR_GermCells_W5 = GermCells_0

m. KSR_GermCells_W6 < GermCells_0

n. KSR_GermCells_W6 > GermCells_0

o. KSR_GermCells_W6 = GermCells_0

**Test Statistics[a]**

| | KSR_GermCells_W2 - GermCells_0 | KSR_GermCells_W3 - GermCells_0 | KSR_GermCells_W4 - GermCells_0 | KSR_GermCells_W5 - GermCells_0 | KSR_GermCells_W6 - GermCells_0 |
|---|---|---|---|---|---|
| Z | -1,753[b] | -1,992[b] | -1,753[b] | -2,201[b] | -1,992[b] |
| Asymp. Sig. (2-tailed) | ,080 | ,046 | ,080 | ,028 | ,046 |

a. Wilcoxon Signed Ranks Test

b. Based on positive ranks.

```
NPAR TESTS
 /WILCOXON=GermCells_0 GermCells_0 GermCells_0 GermCells_0 GermCells_0 WITH EST_GermCells_W2
 /MISSING ANALYSIS.
```

# NPar Tests

**Notes**

| | | |
|---|---|---|
| Output Created | | 16-AUG-2017 14:28:41 |
| Comments | | |
| Input | Data | /Users/pmota/Desktop/Organ Culture_treatment and ... |
| | Active Dataset | DataSet1 |
| | Filter | <none> |
| | Weight | <none> |
| | Split File | <none> |
| | N of Rows in Working Data File | 6 |
| Missing Value Handling | Definition of Missing | User-defined missing values are treated as missing. |
| | Cases Used | Statistics for each test are based on all cases with valid data for the variable(s) used in that test. |
| Syntax | | NPAR TESTS<br><br>/WILCOXON=GermCells_0 GermCells_0 GermCells_0 GermCells_0 GermCells_0 WITH EST_GermCells_W2 EST_GermCells_W3 EST_GermCells_W4 EST_GermCells_W5 EST_GermCells_W6 (PAIRED)<br>/MISSING ANALYSIS. |
| Resources | Processor Time | 00:00:00,01 |
| | Elapsed Time | 00:00:00,00 |
| | Number of Cases Allowed[a] | 71493 |

a. Based on availability of workspace memory.

```
[DataSet1] /Users/pmota/Desktop/Organ Culture_treatment and week.sav
```

# Wilcoxon Signed Ranks Test

**Ranks**

| | | N | Mean Rank | Sum of Ranks |
|---|---|---|---|---|
| EST_GermCells_W2 - GermCells_0 | Negative Ranks | 2[a] | 2,50 | 5,00 |
| | Positive Ranks | 2[b] | 2,50 | 5,00 |
| | Ties | 0[c] | | |
| | Total | 4 | | |
| EST_GermCells_W3 - GermCells_0 | Negative Ranks | 5[d] | 3,20 | 16,00 |
| | Positive Ranks | 1[e] | 5,00 | 5,00 |
| | Ties | 0[f] | | |
| | Total | 6 | | |
| EST_GermCells_W4 - GermCells_0 | Negative Ranks | 4[g] | 3,75 | 15,00 |
| | Positive Ranks | 2[h] | 3,00 | 6,00 |
| | Ties | 0[i] | | |
| | Total | 6 | | |
| EST_GermCells_W5 - GermCells_0 | Negative Ranks | 4[j] | 2,75 | 11,00 |
| | Positive Ranks | 2[k] | 5,00 | 10,00 |
| | Ties | 0[l] | | |
| | Total | 6 | | |
| EST_GermCells_W6 - GermCells_0 | Negative Ranks | 5[m] | 3,80 | 19,00 |
| | Positive Ranks | 1[n] | 2,00 | 2,00 |
| | Ties | 0[o] | | |
| | Total | 6 | | |

a. EST_GermCells_W2 < GermCells_0

b. EST_GermCells_W2 > GermCells_0

c. EST_GermCells_W2 = GermCells_0

d. EST_GermCells_W3 < GermCells_0

e. EST_GermCells_W3 > GermCells_0

f. EST_GermCells_W3 = GermCells_0

g. EST_GermCells_W4 < GermCells_0

h. EST_GermCells_W4 > GermCells_0

i. EST_GermCells_W4 = GermCells_0

j. EST_GermCells_W5 < GermCells_0

k. EST_GermCells_W5 > GermCells_0

l. EST_GermCells_W5 = GermCells_0

m. EST_GermCells_W6 < GermCells_0

n. EST_GermCells_W6 > GermCells_0

o. EST_GermCells_W6 = GermCells_0

**Test Statistics[a]**

| | EST_GermCells_W2 - GermCells_0 | EST_GermCells_W3 - GermCells_0 | EST_GermCells_W4 - GermCells_0 | EST_GermCells_W5 - GermCells_0 | EST_GermCells_W6 - GermCells_0 |
|---|---|---|---|---|---|
| Z | ,000[b] | -1,153[c] | -,943[c] | -,105[c] | -1,782[c] |
| Asymp. Sig. (2-tailed) | 1,000 | ,249 | ,345 | ,917 | ,075 |

a. Wilcoxon Signed Ranks Test

b. The sum of negative ranks equals the sum of positive ranks.

c. Based on positive ranks.

```
NPAR TESTS
 /WILCOXON=GermCells_0 GermCells_0 GermCells_0 GermCells_0 GermCells_0 WITH ALB_GermCells_W2 /
 /MISSING ANALYSIS.
```

# NPar Tests

**Notes**

| | | |
|---|---|---|
| Output Created | | 16-AUG-2017 14:29:44 |
| Comments | | |
| Input | Data | /Users/pmota/Desktop/Organ Culture_treatment and ... |
| | Active Dataset | DataSet1 |
| | Filter | <none> |
| | Weight | <none> |
| | Split File | <none> |
| | N of Rows in Working Data File | 6 |
| Missing Value Handling | Definition of Missing | User-defined missing values are treated as missing. |
| | Cases Used | Statistics for each test are based on all cases with valid data for the variable(s) used in that test. |
| Syntax | | NPAR TESTS /WILCOXON=GermCells_0 GermCells_0 GermCells_0 GermCells_0 GermCells_0 WITH ALB_GermCells_W2 ALB_GermCells_W3 ALB_GermCells_W4 ALB_GermCells_W5 ALB_GermCells_W6 (PAIRED) /MISSING ANALYSIS. |
| Resources | Processor Time | 00:00:00,01 |
| | Elapsed Time | 00:00:00,00 |
| | Number of Cases Allowed[a] | 71493 |

a. Based on availability of workspace memory.

[DataSet1] /Users/pmota/Desktop/Organ Culture_treatment and week.sav

# Wilcoxon Signed Ranks Test

### Ranks

| | | N | Mean Rank | Sum of Ranks |
|---|---|---|---|---|
| ALB_GermCells_W2 - GermCells_0 | Negative Ranks | 4[a] | 2,50 | 10,00 |
| | Positive Ranks | 1[b] | 5,00 | 5,00 |
| | Ties | 0[c] | | |
| | Total | 5 | | |
| ALB_GermCells_W3 - GermCells_0 | Negative Ranks | 5[d] | 3,00 | 15,00 |
| | Positive Ranks | 1[e] | 6,00 | 6,00 |
| | Ties | 0[f] | | |
| | Total | 6 | | |
| ALB_GermCells_W4 - GermCells_0 | Negative Ranks | 5[g] | 3,00 | 15,00 |
| | Positive Ranks | 1[h] | 6,00 | 6,00 |
| | Ties | 0[i] | | |
| | Total | 6 | | |
| ALB_GermCells_W5 - GermCells_0 | Negative Ranks | 6[j] | 3,50 | 21,00 |
| | Positive Ranks | 0[k] | ,00 | ,00 |
| | Ties | 0[l] | | |
| | Total | 6 | | |
| ALB_GermCells_W6 - GermCells_0 | Negative Ranks | 5[m] | 3,60 | 18,00 |
| | Positive Ranks | 1[n] | 3,00 | 3,00 |
| | Ties | 0[o] | | |
| | Total | 6 | | |

a. ALB_GermCells_W2 < GermCells_0

b. ALB_GermCells_W2 > GermCells_0

c. ALB_GermCells_W2 = GermCells_0

d. ALB_GermCells_W3 < GermCells_0

e. ALB_GermCells_W3 > GermCells_0

f. ALB_GermCells_W3 = GermCells_0

g. ALB_GermCells_W4 < GermCells_0

h. ALB_GermCells_W4 > GermCells_0

i. ALB_GermCells_W4 = GermCells_0

j. ALB_GermCells_W5 < GermCells_0

k. ALB_GermCells_W5 > GermCells_0

l. ALB_GermCells_W5 = GermCells_0

m. ALB_GermCells_W6 < GermCells_0

n. ALB_GermCells_W6 > GermCells_0

o. ALB_GermCells_W6 = GermCells_0

### Test Statistics[a]

| | ALB_GermCells_W2 - GermCells_0 | ALB_GermCells_W3 - GermCells_0 | ALB_GermCells_W4 - GermCells_0 | ALB_GermCells_W5 - GermCells_0 | ALB_GermCells_W6 - GermCells_0 |
|---|---|---|---|---|---|
| Z | -,674[b] | -,943[b] | -,943[b] | -2,201[b] | -1,572[b] |
| Asymp. Sig. (2-tailed) | ,500 | ,345 | ,345 | ,028 | ,116 |

a. Wilcoxon Signed Ranks Test

b. Based on positive ranks.

```
NPAR TESTS
 /WILCOXON=GermCells_0 GermCells_0 GermCells_0 GermCells_0 GermCells_0 WITH ALB_EST_GermCells_
 /MISSING ANALYSIS.
```

# NPar Tests

**Notes**

| | | |
|---|---|---|
| Output Created | | 16-AUG-2017 15:20:50 |
| Comments | | |
| Input | Data | /Users/pmota/Desktop/Organ Culture_treatment and ... |
| | Active Dataset | DataSet1 |
| | Filter | <none> |
| | Weight | <none> |
| | Split File | <none> |
| | N of Rows in Working Data File | 6 |
| Missing Value Handling | Definition of Missing | User-defined missing values are treated as missing. |
| | Cases Used | Statistics for each test are based on all cases with valid data for the variable(s) used in that test. |
| Syntax | | NPAR TESTS<br><br>/WILCOXON=GermCells_0 GermCells_0 GermCells_0 GermCells_0 GermCells_0 WITH ALB_EST_GermCells_W2 ALB_EST_GermCells_W3 ALB_EST_GermCells_W4 ALB_EST_GermCells_W5 ALB_EST_GermCells_W6 (PAIRED)<br>/MISSING ANALYSIS. |
| Resources | Processor Time | 00:00:00,02 |
| | Elapsed Time | 00:00:01,00 |
| | Number of Cases Allowed[a] | 71493 |

a. Based on availability of workspace memory.

```
[DataSet1] /Users/pmota/Desktop/Organ Culture_treatment and week.sav
```

# Wilcoxon Signed Ranks Test

## Ranks

| | | N | Mean Rank | Sum of Ranks |
|---|---|---|---|---|
| ALB_EST_GermCells_W2 - GermCells_0 | Negative Ranks | 4[a] | 2,50 | 10,00 |
| | Positive Ranks | 1[b] | 5,00 | 5,00 |
| | Ties | 0[c] | | |
| | Total | 5 | | |
| ALB_EST_GermCells_W3 - GermCells_0 | Negative Ranks | 5[d] | 3,00 | 15,00 |
| | Positive Ranks | 1[e] | 6,00 | 6,00 |
| | Ties | 0[f] | | |
| | Total | 6 | | |
| ALB_EST_GermCells_W4 - GermCells_0 | Negative Ranks | 5[g] | 3,00 | 15,00 |
| | Positive Ranks | 1[h] | 6,00 | 6,00 |
| | Ties | 0[i] | | |
| | Total | 6 | | |
| ALB_EST_GermCells_W5 - GermCells_0 | Negative Ranks | 5[j] | 3,00 | 15,00 |
| | Positive Ranks | 1[k] | 6,00 | 6,00 |
| | Ties | 0[l] | | |
| | Total | 6 | | |
| ALB_EST_GermCells_W6 - GermCells_0 | Negative Ranks | 5[m] | 3,60 | 18,00 |
| | Positive Ranks | 1[n] | 3,00 | 3,00 |
| | Ties | 0[o] | | |
| | Total | 6 | | |

a. ALB_EST_GermCells_W2 < GermCells_0

b. ALB_EST_GermCells_W2 > GermCells_0

c. ALB_EST_GermCells_W2 = GermCells_0

d. ALB_EST_GermCells_W3 < GermCells_0

e. ALB_EST_GermCells_W3 > GermCells_0

f. ALB_EST_GermCells_W3 = GermCells_0

g. ALB_EST_GermCells_W4 < GermCells_0

h. ALB_EST_GermCells_W4 > GermCells_0

i. ALB_EST_GermCells_W4 = GermCells_0

j. ALB_EST_GermCells_W5 < GermCells_0

k. ALB_EST_GermCells_W5 > GermCells_0

l. ALB_EST_GermCells_W5 = GermCells_0

m. ALB_EST_GermCells_W6 < GermCells_0

n. ALB_EST_GermCells_W6 > GermCells_0

o. ALB_EST_GermCells_W6 = GermCells_0

## Test Statistics[a]

| | ALB_EST_GermCells_W2 - GermCells_0 | ALB_EST_GermCells_W3 - GermCells_0 | ALB_EST_GermCells_W4 - GermCells_0 | ALB_EST_GermCells_W5 - GermCells_0 | ALB_EST_GermCells_W6 - GermCells_0 |
|---|---|---|---|---|---|
| Z | -,674[b] | -,943[b] | -,943[b] | -,943[b] | -1,572[b] |
| Asymp. Sig. (2-tailed) | ,500 | ,345 | ,345 | ,345 | ,116 |

a. Wilcoxon Signed Ranks Test

b. Based on positive ranks.

```
NPAR TESTS
 /WILCOXON=MEM_GermCells_W2 MEM_GermCells_W3 MEM_GermCells_W4 MEM_GermCells_W5 MEM_GermCells_W
 /MISSING ANALYSIS.
```

# NPar Tests

**Notes**

| | | |
|---|---|---|
| Output Created | | 16-AUG-2017 16:35:00 |
| Comments | | |
| Input | Data | /Users/pmota/Desktop/Organ Culture_treatment and ... |
| | Active Dataset | DataSet1 |
| | Filter | <none> |
| | Weight | <none> |
| | Split File | <none> |
| | N of Rows in Working Data File | 6 |
| Missing Value Handling | Definition of Missing | User-defined missing values are treated as missing. |
| | Cases Used | Statistics for each test are based on all cases with valid data for the variable(s) used in that test. |
| Syntax | | NPAR TESTS<br><br>/WILCOXON=MEM_GermCells_W2 MEM_GermCells_W3 MEM_GermCells_W4 MEM_GermCells_W5 MEM_GermCells_W6 WITH KSR_GermCells_W2 KSR_GermCells_W3 KSR_GermCells_W4 KSR_GermCells_W5 KSR_GermCells_W6 (PAIRED)<br>/MISSING ANALYSIS. |
| Resources | Processor Time | 00:00:00,01 |
| | Elapsed Time | 00:00:00,00 |
| | Number of Cases Allowed[a] | 52428 |

a. Based on availability of workspace memory.

```
[DataSet1] /Users/pmota/Desktop/Organ Culture_treatment and week.sav
```

# Wilcoxon Signed Ranks Test

**Ranks**

| | | N | Mean Rank | Sum of Ranks |
|---|---|---|---|---|
| KSR_GermCells_W2 - MEM_GermCells_W2 | Negative Ranks | 5[a] | 3,00 | 15,00 |
| | Positive Ranks | 0[b] | ,00 | ,00 |
| | Ties | 0[c] | | |
| | Total | 5 | | |
| KSR_GermCells_W3 - MEM_GermCells_W3 | Negative Ranks | 6[d] | 3,50 | 21,00 |
| | Positive Ranks | 0[e] | ,00 | ,00 |
| | Ties | 0[f] | | |
| | Total | 6 | | |
| KSR_GermCells_W4 - MEM_GermCells_W4 | Negative Ranks | 5[g] | 3,00 | 15,00 |
| | Positive Ranks | 0[h] | ,00 | ,00 |
| | Ties | 0[i] | | |
| | Total | 5 | | |
| KSR_GermCells_W5 - MEM_GermCells_W5 | Negative Ranks | 6[j] | 3,50 | 21,00 |
| | Positive Ranks | 0[k] | ,00 | ,00 |
| | Ties | 0[l] | | |
| | Total | 6 | | |
| KSR_GermCells_W6 - MEM_GermCells_W6 | Negative Ranks | 3[m] | 2,67 | 8,00 |
| | Positive Ranks | 2[n] | 3,50 | 7,00 |
| | Ties | 1[o] | | |
| | Total | 6 | | |

a. KSR_GermCells_W2 < MEM_GermCells_W2

b. KSR_GermCells_W2 > MEM_GermCells_W2

c. KSR_GermCells_W2 = MEM_GermCells_W2

d. KSR_GermCells_W3 < MEM_GermCells_W3

e. KSR_GermCells_W3 > MEM_GermCells_W3

f. KSR_GermCells_W3 = MEM_GermCells_W3

g. KSR_GermCells_W4 < MEM_GermCells_W4

h. KSR_GermCells_W4 > MEM_GermCells_W4

i. KSR_GermCells_W4 = MEM_GermCells_W4

j. KSR_GermCells_W5 < MEM_GermCells_W5

k. KSR_GermCells_W5 > MEM_GermCells_W5

l. KSR_GermCells_W5 = MEM_GermCells_W5

m. KSR_GermCells_W6 < MEM_GermCells_W6

n. KSR_GermCells_W6 > MEM_GermCells_W6

o. KSR_GermCells_W6 = MEM_GermCells_W6

**Test Statistics[a]**

| | KSR_GermCells_W2 - MEM_GermCells_W2 | KSR_GermCells_W3 - MEM_GermCells_W3 | KSR_GermCells_W4 - MEM_GermCells_W4 | KSR_GermCells_W5 - MEM_GermCells_W5 | KSR_GermCells_W6 - MEM_GermCells_W6 |
|---|---|---|---|---|---|
| Z | -2,023[b] | -2,201[b] | -2,023[b] | -2,201[b] | -,135[b] |
| Asymp. Sig. (2-tailed) | ,043 | ,028 | ,043 | ,028 | ,893 |

a. Wilcoxon Signed Ranks Test

b. Based on positive ranks.

```
NPAR TESTS
 /WILCOXON=MEM_GermCells_W2 MEM_GermCells_W3 MEM_GermCells_W4 MEM_GermCells_W5 MEM_GermCells_W
 /MISSING ANALYSIS.
```

# NPar Tests

**Notes**

| | | |
|---|---|---|
| Output Created | | 16-AUG-2017 16:36:07 |
| Comments | | |
| Input | Data | /Users/pmota/Desktop/Organ Culture_treatment and ... |
| | Active Dataset | DataSet1 |
| | Filter | <none> |
| | Weight | <none> |
| | Split File | <none> |
| | N of Rows in Working Data File | 6 |
| Missing Value Handling | Definition of Missing | User-defined missing values are treated as missing. |
| | Cases Used | Statistics for each test are based on all cases with valid data for the variable(s) used in that test. |
| Syntax | | NPAR TESTS /WILCOXON=MEM_GermCells_W2 MEM_GermCells_W3 MEM_GermCells_W4 MEM_GermCells_W5 MEM_GermCells_W6 WITH EST_GermCells_W2 EST_GermCells_W3 EST_GermCells_W4 EST_GermCells_W5 EST_GermCells_W6 (PAIRED) /MISSING ANALYSIS. |
| Resources | Processor Time | 00:00:00,01 |
| | Elapsed Time | 00:00:00,00 |
| | Number of Cases Allowed[a] | 52428 |

a. Based on availability of workspace memory.

[DataSet1] /Users/pmota/Desktop/Organ Culture_treatment and week.sav

# Wilcoxon Signed Ranks Test

**Ranks**

| | | N | Mean Rank | Sum of Ranks |
|---|---|---|---|---|
| EST_GermCells_W2 - MEM_GermCells_W2 | Negative Ranks | 1[a] | 1,00 | 1,00 |
| | Positive Ranks | 3[b] | 3,00 | 9,00 |
| | Ties | 0[c] | | |
| | Total | 4 | | |
| EST_GermCells_W3 - MEM_GermCells_W3 | Negative Ranks | 3[d] | 3,67 | 11,00 |
| | Positive Ranks | 3[e] | 3,33 | 10,00 |
| | Ties | 0[f] | | |
| | Total | 6 | | |
| EST_GermCells_W4 - MEM_GermCells_W4 | Negative Ranks | 3[g] | 4,67 | 14,00 |
| | Positive Ranks | 3[h] | 2,33 | 7,00 |
| | Ties | 0[i] | | |
| | Total | 6 | | |
| EST_GermCells_W5 - MEM_GermCells_W5 | Negative Ranks | 2[j] | 1,50 | 3,00 |
| | Positive Ranks | 4[k] | 4,50 | 18,00 |
| | Ties | 0[l] | | |
| | Total | 6 | | |
| EST_GermCells_W6 - MEM_GermCells_W6 | Negative Ranks | 1[m] | 3,00 | 3,00 |
| | Positive Ranks | 4[n] | 3,00 | 12,00 |
| | Ties | 1[o] | | |
| | Total | 6 | | |

a. EST_GermCells_W2 < MEM_GermCells_W2

b. EST_GermCells_W2 > MEM_GermCells_W2

c. EST_GermCells_W2 = MEM_GermCells_W2

d. EST_GermCells_W3 < MEM_GermCells_W3

e. EST_GermCells_W3 > MEM_GermCells_W3

f. EST_GermCells_W3 = MEM_GermCells_W3

g. EST_GermCells_W4 < MEM_GermCells_W4

h. EST_GermCells_W4 > MEM_GermCells_W4

i. EST_GermCells_W4 = MEM_GermCells_W4

j. EST_GermCells_W5 < MEM_GermCells_W5

k. EST_GermCells_W5 > MEM_GermCells_W5

l. EST_GermCells_W5 = MEM_GermCells_W5

m. EST_GermCells_W6 < MEM_GermCells_W6

n. EST_GermCells_W6 > MEM_GermCells_W6

o. EST_GermCells_W6 = MEM_GermCells_W6

**Test Statistics[a]**

| | EST_GermCells_W2 - MEM_GermCells_W2 | EST_GermCells_W3 - MEM_GermCells_W3 | EST_GermCells_W4 - MEM_GermCells_W4 | EST_GermCells_W5 - MEM_GermCells_W5 | EST_GermCells_W6 - MEM_GermCells_W6 |
|---|---|---|---|---|---|
| Z | -1,461[b] | -,105[c] | -,734[c] | -1,572[b] | -1,214[b] |
| Asymp. Sig. (2-tailed) | ,144 | ,917 | ,463 | ,116 | ,225 |

a. Wilcoxon Signed Ranks Test

b. Based on negative ranks.

c. Based on positive ranks.

```
NPAR TESTS
 /WILCOXON=MEM_GermCells_W2 MEM_GermCells_W3 MEM_GermCells_W4 MEM_GermCells_W5 MEM_GermCells_W
```

/MISSING ANALYSIS.

# NPar Tests

**Notes**

| | | |
|---|---|---|
| Output Created | | 16-AUG-2017 16:36:42 |
| Comments | | |
| Input | Data | /Users/pmota/Desktop/Organ Culture_treatment and ... |
| | Active Dataset | DataSet1 |
| | Filter | <none> |
| | Weight | <none> |
| | Split File | <none> |
| | N of Rows in Working Data File | 6 |
| Missing Value Handling | Definition of Missing | User-defined missing values are treated as missing. |
| | Cases Used | Statistics for each test are based on all cases with valid data for the variable(s) used in that test. |
| Syntax | | NPAR TESTS /WILCOXON=MEM_GermCells_W2 MEM_GermCells_W3 MEM_GermCells_W4 MEM_GermCells_W5 MEM_GermCells_W6 WITH ALB_GermCells_W2 ALB_GermCells_W3 ALB_GermCells_W4 ALB_GermCells_W5 ALB_GermCells_W6 (PAIRED) /MISSING ANALYSIS. |
| Resources | Processor Time | 00:00:00,01 |
| | Elapsed Time | 00:00:00,00 |
| | Number of Cases Allowed[a] | 52428 |

a. Based on availability of workspace memory.

[DataSet1] /Users/pmota/Desktop/Organ Culture_treatment and week.sav

# Wilcoxon Signed Ranks Test

**Ranks**

| | | N | Mean Rank | Sum of Ranks |
|---|---|---|---|---|
| ALB_GermCells_W2 - MEM_GermCells_W2 | Negative Ranks | 0[a] | ,00 | ,00 |
| | Positive Ranks | 5[b] | 3,00 | 15,00 |
| | Ties | 0[c] | | |
| | Total | 5 | | |
| ALB_GermCells_W3 - MEM_GermCells_W3 | Negative Ranks | 2[d] | 3,00 | 6,00 |
| | Positive Ranks | 4[e] | 3,75 | 15,00 |
| | Ties | 0[f] | | |
| | Total | 6 | | |
| ALB_GermCells_W4 - MEM_GermCells_W4 | Negative Ranks | 2[g] | 4,00 | 8,00 |
| | Positive Ranks | 4[h] | 3,25 | 13,00 |
| | Ties | 0[i] | | |
| | Total | 6 | | |
| ALB_GermCells_W5 - MEM_GermCells_W5 | Negative Ranks | 5[j] | 3,60 | 18,00 |
| | Positive Ranks | 1[k] | 3,00 | 3,00 |
| | Ties | 0[l] | | |
| | Total | 6 | | |
| ALB_GermCells_W6 - MEM_GermCells_W6 | Negative Ranks | 1[m] | 4,00 | 4,00 |
| | Positive Ranks | 5[n] | 3,40 | 17,00 |
| | Ties | 0[o] | | |
| | Total | 6 | | |

a. ALB_GermCells_W2 < MEM_GermCells_W2

b. ALB_GermCells_W2 > MEM_GermCells_W2

c. ALB_GermCells_W2 = MEM_GermCells_W2

d. ALB_GermCells_W3 < MEM_GermCells_W3

e. ALB_GermCells_W3 > MEM_GermCells_W3

f. ALB_GermCells_W3 = MEM_GermCells_W3

g. ALB_GermCells_W4 < MEM_GermCells_W4

h. ALB_GermCells_W4 > MEM_GermCells_W4

i. ALB_GermCells_W4 = MEM_GermCells_W4

j. ALB_GermCells_W5 < MEM_GermCells_W5

k. ALB_GermCells_W5 > MEM_GermCells_W5

l. ALB_GermCells_W5 = MEM_GermCells_W5

m. ALB_GermCells_W6 < MEM_GermCells_W6

n. ALB_GermCells_W6 > MEM_GermCells_W6

o. ALB_GermCells_W6 = MEM_GermCells_W6

**Test Statistics[a]**

| | ALB_GermCells_W2 - MEM_GermCells_W2 | ALB_GermCells_W3 - MEM_GermCells_W3 | ALB_GermCells_W4 - MEM_GermCells_W4 | ALB_GermCells_W5 - MEM_GermCells_W5 | ALB_GermCells_W6 - MEM_GermCells_W6 |
|---|---|---|---|---|---|
| Z | -2,023[b] | -,943[b] | -,524[b] | -1,572[c] | -1,363[b] |
| Asymp. Sig. (2-tailed) | ,043 | ,345 | ,600 | ,116 | ,173 |

a. Wilcoxon Signed Ranks Test

b. Based on negative ranks.

c. Based on positive ranks.

```
NPAR TESTS
 /WILCOXON=MEM_GermCells_W2 MEM_GermCells_W3 MEM_GermCells_W4 MEM_GermCells_W5 MEM_GermCells_W
```

```
/MISSING ANALYSIS.
```

# NPar Tests

**Notes**

| | | |
|---|---|---|
| Output Created | | 16-AUG-2017 16:37:29 |
| Comments | | |
| Input | Data | /Users/pmota/Desktop/Organ Culture_treatment and ... |
| | Active Dataset | DataSet1 |
| | Filter | <none> |
| | Weight | <none> |
| | Split File | <none> |
| | N of Rows in Working Data File | 6 |
| Missing Value Handling | Definition of Missing | User-defined missing values are treated as missing. |
| | Cases Used | Statistics for each test are based on all cases with valid data for the variable(s) used in that test. |
| Syntax | | NPAR TESTS /WILCOXON=MEM_GermCells_W2 MEM_GermCells_W3 MEM_GermCells_W4 MEM_GermCells_W5 MEM_GermCells_W6 WITH ALB_EST_GermCells_W2 ALB_EST_GermCells_W3 ALB_EST_GermCells_W4 ALB_EST_GermCells_W5 ALB_EST_GermCells_W6 (PAIRED) /MISSING ANALYSIS. |
| Resources | Processor Time | 00:00:00,01 |
| | Elapsed Time | 00:00:00,00 |
| | Number of Cases Allowed[a] | 52428 |

a. Based on availability of workspace memory.

```
[DataSet1] /Users/pmota/Desktop/Organ Culture_treatment and week.sav
```

# Wilcoxon Signed Ranks Test

## Ranks

| | | N | Mean Rank | Sum of Ranks |
|---|---|---|---|---|
| ALB_EST_GermCells_W2 - MEM_GermCells_W2 | Negative Ranks | 2[a] | 1,50 | 3,00 |
| | Positive Ranks | 3[b] | 4,00 | 12,00 |
| | Ties | 0[c] | | |
| | Total | 5 | | |
| ALB_EST_GermCells_W3 - MEM_GermCells_W3 | Negative Ranks | 1[d] | 3,00 | 3,00 |
| | Positive Ranks | 4[e] | 3,00 | 12,00 |
| | Ties | 1[f] | | |
| | Total | 6 | | |
| ALB_EST_GermCells_W4 - MEM_GermCells_W4 | Negative Ranks | 2[g] | 4,00 | 8,00 |
| | Positive Ranks | 4[h] | 3,25 | 13,00 |
| | Ties | 0[i] | | |
| | Total | 6 | | |
| ALB_EST_GermCells_W5 - MEM_GermCells_W5 | Negative Ranks | 3[j] | 3,00 | 9,00 |
| | Positive Ranks | 3[k] | 4,00 | 12,00 |
| | Ties | 0[l] | | |
| | Total | 6 | | |
| ALB_EST_GermCells_W6 - MEM_GermCells_W6 | Negative Ranks | 1[m] | 4,00 | 4,00 |
| | Positive Ranks | 5[n] | 3,40 | 17,00 |
| | Ties | 0[o] | | |
| | Total | 6 | | |

a. ALB_EST_GermCells_W2 < MEM_GermCells_W2

b. ALB_EST_GermCells_W2 > MEM_GermCells_W2

c. ALB_EST_GermCells_W2 = MEM_GermCells_W2

d. ALB_EST_GermCells_W3 < MEM_GermCells_W3

e. ALB_EST_GermCells_W3 > MEM_GermCells_W3

f. ALB_EST_GermCells_W3 = MEM_GermCells_W3

g. ALB_EST_GermCells_W4 < MEM_GermCells_W4

h. ALB_EST_GermCells_W4 > MEM_GermCells_W4

i. ALB_EST_GermCells_W4 = MEM_GermCells_W4

j. ALB_EST_GermCells_W5 < MEM_GermCells_W5

k. ALB_EST_GermCells_W5 > MEM_GermCells_W5

l. ALB_EST_GermCells_W5 = MEM_GermCells_W5

m. ALB_EST_GermCells_W6 < MEM_GermCells_W6

n. ALB_EST_GermCells_W6 > MEM_GermCells_W6

o. ALB_EST_GermCells_W6 = MEM_GermCells_W6

## Test Statistics[a]

| | ALB_EST_GermCells_W2 - MEM_GermCells_W2 | ALB_EST_GermCells_W3 - MEM_GermCells_W3 | ALB_EST_GermCells_W4 - MEM_GermCells_W4 | ALB_EST_GermCells_W5 - MEM_GermCells_W5 | ALB_EST_GermCells_W6 - MEM_GermCells_W6 |
|---|---|---|---|---|---|
| Z | -1,214[b] | -1,214[b] | -,524[b] | -,314[b] | -1,363[b] |
| Asymp. Sig. (2-tailed) | ,225 | ,225 | ,600 | ,753 | ,173 |

a. Wilcoxon Signed Ranks Test

b. Based on negative ranks.
