# Supplementary material for: Can we induce spermatogenesis in the domestic cat using an in vitro tissue culture approach?
Source: PLoS One. 2018 Feb 7;13(2):e0191912. doi: 10.1371/journal.pone.0191912 (PMC5802888; doi:10.1371/journal.pone.0191912)
Supplement: S2 File — Histology, categorization and graphical representation (Panel A, B and C, respectively) of organ culture of B6 and 129 mouse strains testicular tissue using the protocol of Sato and colleagues [5]. (PDF) [file pone.0191912.s002.pdf]

A

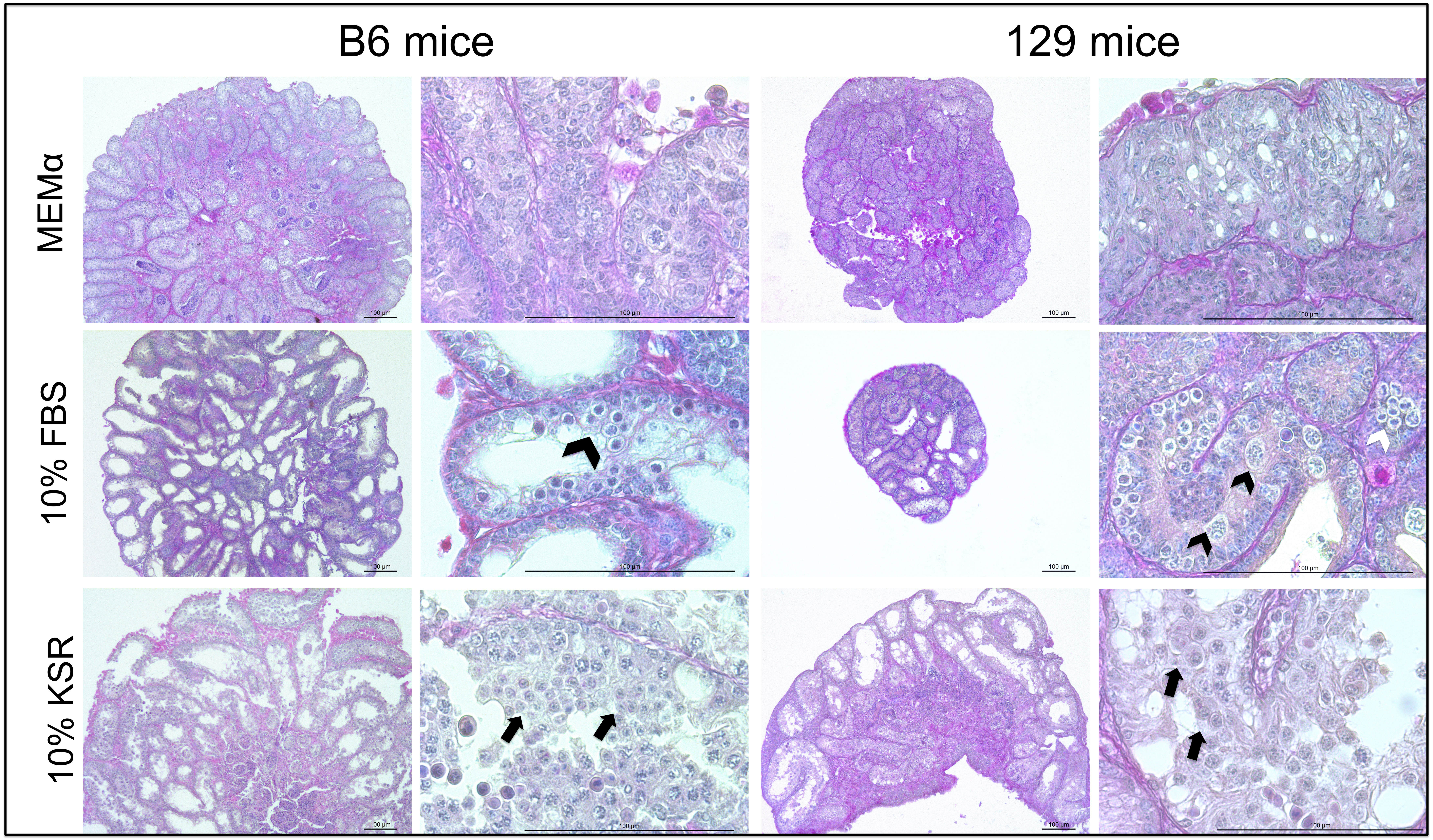

Panel A: Representative images of H&PAS stained fragments of mice testicular tissue cultured for 21 days (B6 mice) or for 28 days (129 mice) in the different types of media tested. Late spermatocytes (black arrowed) and meiotic figures (white arrowed) are the more developed germ cells present in the fragments cultured with FBS, while rounds spermatids (arrow) are observed in the KSR supplemented culture. Images at 100x and 630x magnification. Scale bar: 100µm.

B

**Ranking of Spermatogenic development**	
Sertoli cell only	**1**
Atrophy	**2**
ST without lumen	**3**
ST with lumen but without spermatogenic development	**4**
ST with early primary spermatocytes	**5**
ST with late primary spermatocytes	**6**
ST with many late primary spermatocytes	**7**
ST with meiosis figure or secondary spermatocytes	**8**
ST with round spermatids	**9**
ST with elongating spermatids	**10**

C

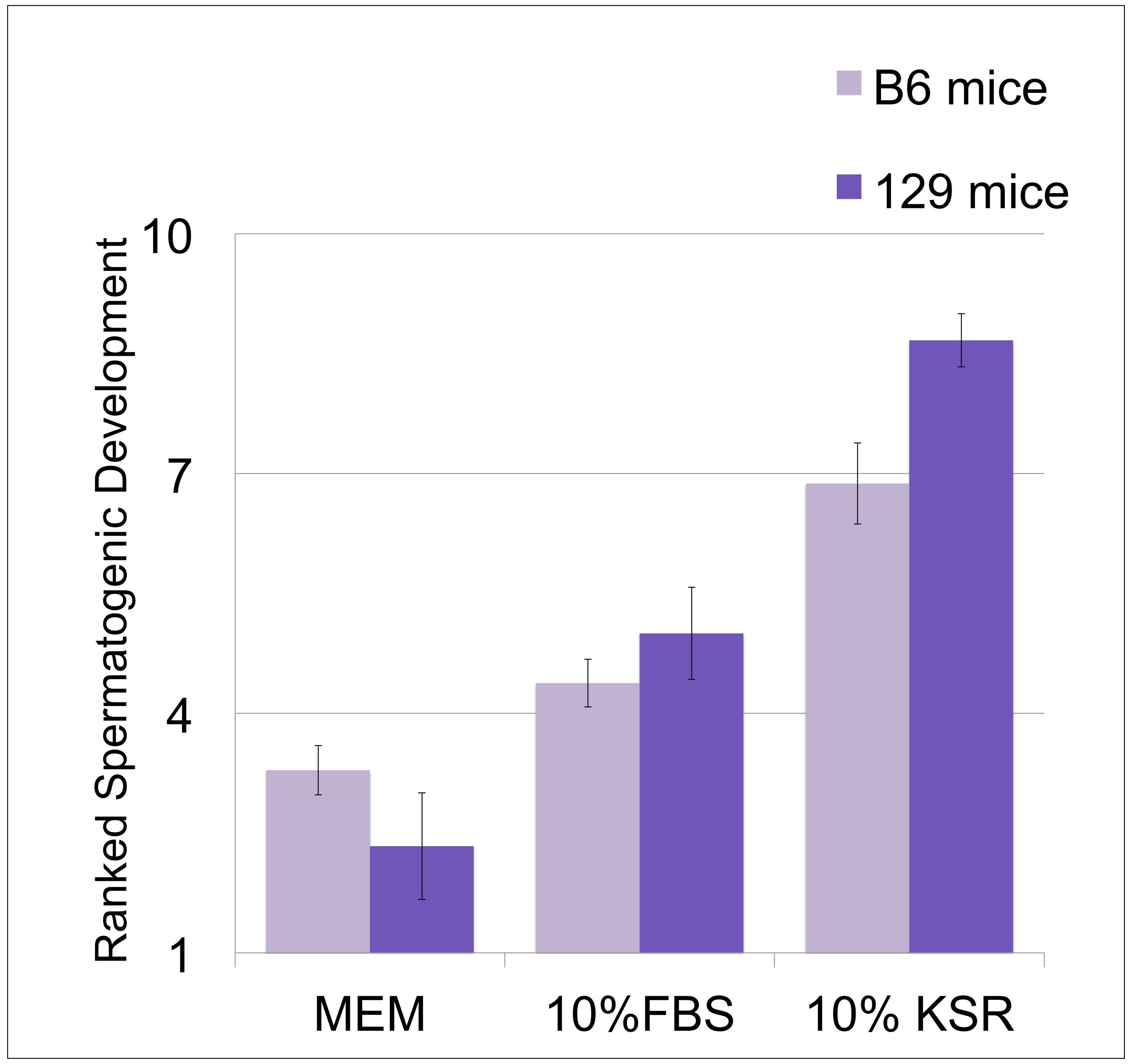

Panel B: Table describing the ranking used to transform histological descriptive observations into the numerical data used to assess development of mice testicular tissue. ST – Seminiferous tubules.

Panel C: Illustrative graph of the ranked development (mean ± sem) of B6 and 129 mice testicular tissue in the different mediums used.
